# Supplementary material for: Direct alkylation of N,N-dialkyl benzamides with methyl sulfides under transition metal-free conditions
Source: Commun Chem. 2021 Sep 27;4:138. doi: 10.1038/s42004-021-00575-2 (PMC9814863; doi:10.1038/s42004-021-00575-2)
Supplement: Supplementary file 1 — Supplementary Information [file 42004_2021_575_MOESM1_ESM.pdf]

Supplementary Information

Direct alkylation of *N,N*-dialkyl benzamides with methyl sulfides under transition metal-free conditions

*Can-Can Bao[1], Hui-Zhen Du[1], Yan-Long Luo[1] and Bing-Tao Guan[2]**

[1]College of Chemistry, Nankai University, Tianjin 300071, China

[2]Department of chemistry, Fudan University, 2005 Songhu Road, Shanghai 200438, China

E-mail: bguan@fudan.edu.cn

Table of Contents

1. Supplementary Methods S2

2. Control Experiments S5

3. General Information S15

4. General Procedures and Analytical Data of Compound **3** and **5** S16

5. NMR Spectra S38

6. Supplementary References S94

1. Supplementary Methods

Supplementary Table 1 Alkylation of *N,N*-diisopropyl benzamide with different amount of LDA[a]

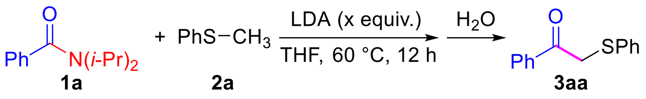

entry	LDA	**3aa** yield (%)[b]
1	64.3 mg (1.2 equiv.)	94
2	58.9 mg (1.1 equiv.)	94
3	53.6 mg (1.0 equiv.)	91
4	42.8 mg (0.8 equiv.)	70
5	32.1 mg (0.6 equiv.)	52
6	21.4 mg (0.4 equiv.)	36
7	10.7 mg (0.2 equiv.)	16

[a]Conditions: bezamide **1a** (0.50 mmol), thioanisole **2a** (1.00 mmol, 2.0 equiv.), LDA, THF (1.0 mL), 60 ℃, 12 h. LDA: lithium diisopropylamide. [b]NMR yields with 2-methyloxynaphthalene as an internal standard.

Supplementary Table 2 Alkylation of *N,N*-diisopropyl benzamide with different LDAs[a]

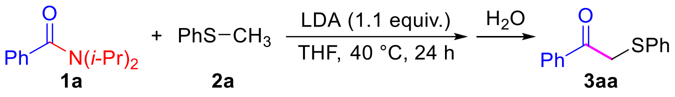

entry	source of LDA	**3aa** yield (%)[b]
1	solid, synthesized according to literature	98
2	2.0 M solution in THF/n-heptane/ethylbenzene (J&K Scientific)	95
3	2.0 M solution in THF/n-heptane/ethylbenzene (Shanghai Macklin Biochemical Co., Ltd)	95
4	1.0 M solution in THF/n-heptane/ethylbenzene (Sigma-Aldrich)	98
5	2.0 M solution in heptane/THF/ethylbenzene (Shanghai Aladdin Biochemical Technology Co., Ltd)	97

[a]Conditions: bezamide **1a** (0.50 mmol), thioanisole **2a** (0.70 mmol, 1.4 equiv.), LDA (0.55 mmol, 1.1 equiv.), THF (1.0 mL), 40 ℃, 24 h. LDA: lithium diisopropylamide. [b]NMR yields with 2-methyloxynaphthalene as an internal standard.

Supplementary Table 3 Alkylation of *N,N*-diisopropyl benzamide in different solvents[a]

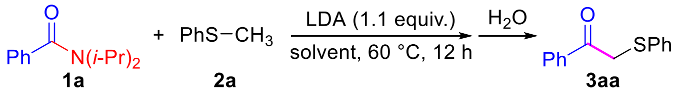

entry	solvent	**3aa** yield (%)[b]
1	THF	94
2	Et_2O	82
3[c]	CPME	80
4	*t*-BuOMe	78
5	hexane	90

[a]Conditions: bezamide **1a** (0.50 mmol), thioanisole **2a** (1.00 mmol, 2.0 equiv.), LDA (0.55 mmol, 1.1 equiv.), solvent (1.0 mL), 60 ℃, 12 h. LDA: lithium diisopropylamide. [b]NMR yields with 2-methyloxynaphthalene as an internal standard. [c]CPME: cyclopentyl methyl ether.

Supplementary Table 4 Additional substrates in the alkylation of benzamides[a]

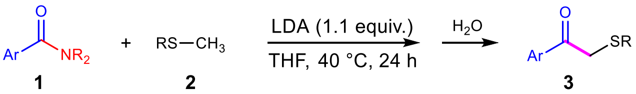

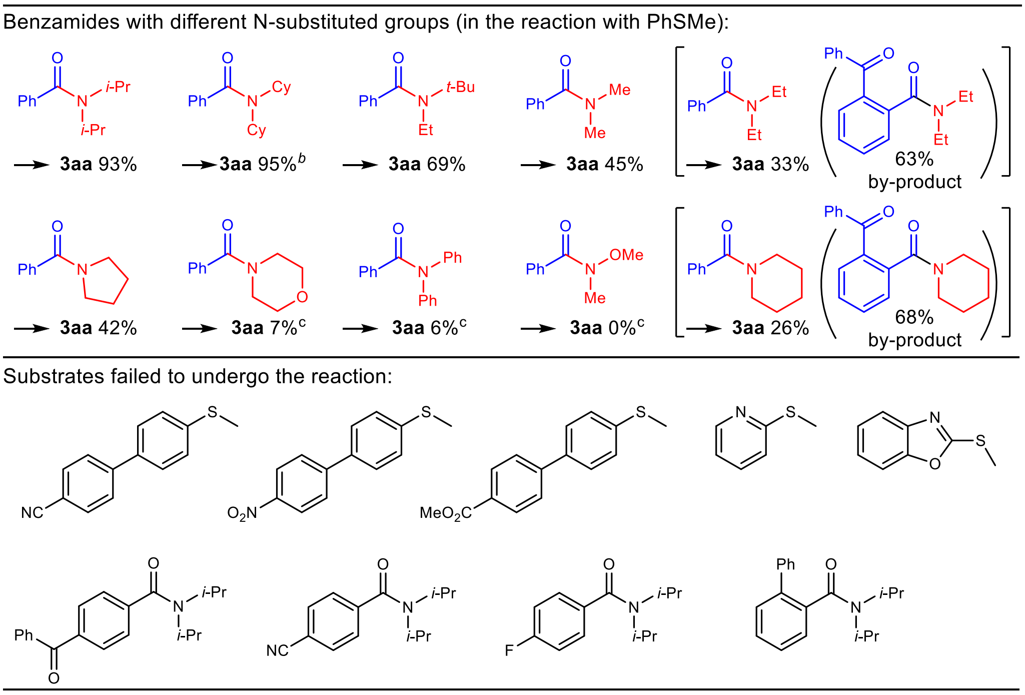

[a]Conditions: amide **1** (0.5 mmol), **2** (0.7 mmol, 1.4 equiv.), LDA (0.55 mmol, 1.1 equiv.), THF (1 mL), 40 ℃, 24 h, isolated yield. [b]Cy: cyclohexyl. [c]NMR yield with 2-methyloxynaphthalene as an internal standard.

Note: 1. Tertiary benzemides were not stable in absence of sulfides under LDA conditions. 2. Benzamides and methyl sulfides with sterically bulky or strong coordination groups failed to undergo the reaction.

Supplementary Table 5 Alkylation of *N,N*-diisopropyl benzamide with isopropyl methyl sulfide[a]

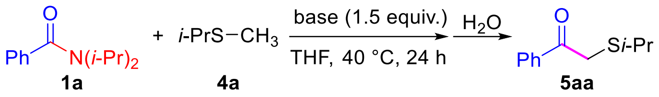

entry	base	**1a** conv. (%)[b]	**5aa** yield (%)[b]
1	LiHMDS	< 5	0
2	KHMDS	< 5	0
3	LiTMP	> 95	80
4	LDA	82	82
5	*n*-BuLi[c]	> 95	10[d]
6	$LiCH_2TMS$	> 95	24
7[e]	LDA	93	92 (85)

[a]Conditions: bezamide **1a** (0.50 mmol), thioanisole **4a** (0.70 mmol, 1.4 equiv.), base (0.75 mmol, 1.5 equiv.), THF (1.0 mL), 40 ℃, 24 h. HMDS: bis(trimethylsilyl)amide; LDA: lithium diisopropylamide; LiTMP: lithium 2,2,6,6-tetramethylpiperidide. [b]NMR yields and conversions with 1,3,5-trimethoxybenzene as an internal standard, isolated yield in parenthesis. [c]1.6 M in hexane. [d]Butyl phenyl ketone and 5-phenylnonan-5-ol were obtained as by-products in 24% and 36% yields, respectively. [e]LDA (0.75 mmol, 1.5 equiv.), **4a** (1.25 mmol, 2.5 equiv.) were used.

Supplementary Table 6 Side-products analysis with *n*-BuLi as base[a]

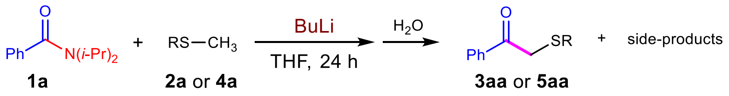

sulfide	product	side-products		
H_3C−SPh **2a**	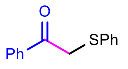 21%	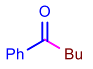 30%	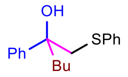 4%	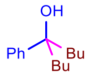 14%
H_3C−S^iPr **4a**	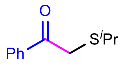 10%	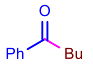 24%	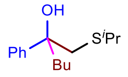 4%	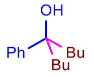 36%

[a]Conditions: for sulfide **2a**: bezamide **1a** (0.50 mmol), thioanisole **2a** (1.00 mmol, 2.0 equiv.), BuLi (0.60 mmol, 1.2 equiv.), THF (1.0 mL), 60 °C, 24 h. GC yields with dodecane as an internal standard.; for sulfide **4a**: bezamide **1a** (0.50 mmol), thioanisole **4a** (0.70 mmol, 1.4 equiv.), BuLi (0.75 mmol, 1.5 equiv.), THF (1.0 mL), 40 °C, 24 h. GC yields with dodecane as an internal standard.

The alkylation of *N*, *N*-diisopropylbenzamide with methyl sulfides using *n*-BuLi as base consumed most reactant but only afforded the ketone product in low yield (Table 1, entry 5 and Supplementary Table 5, entry 5). To clarify the side-products, we detected the identifiable compounds in these reactions and provided the GC yields (with dodecane as an internal standard). Apart for the desired ketone products, butyl phenyl ketones and their over addition product tertiary alcohol products were also obtained.

2. Control Experiments

2.1 Capture of enolate intermediate

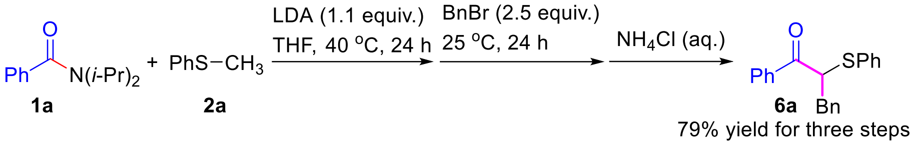

To a 25 mL Schlenk tube equipped with a Teflon septum and magnetic stir bar were added benzamide **1a** (102.7 mg, 0.50 mmol), methyl phenyl sulfide **2a** (86.9 mg, 82 µL, 0.70 mmol, 1.4 equiv.), THF (1.0 mL) and LDA (58.9 mg, 0.55 mmol, 1.1 equiv., solid). The tube was sealed and stirred at 40 °C for 24 hours. The reaction mixture was then cooled to room temperature. BnBr (213.8 mg, 148 µL, 1.25 mmol, 2.5 equiv.) was added to the reaction mixture under nitrogen atmosphere. After stirring at 25 °C for another 24 hours, the reaction mixture was quenched by adding 10 mL of H_2O and then diluted with ethyl acetate (EtOAc, 30 mL). After separation, the aqueous solution was extracted thrice with EtOAc (30 mL x 3) and the combined organic layer was washed with brine (10 mL), dried over anhydrous Na_2SO_4. After filtration and concentration by rotary evaporation, the residue was purified by silica gel column chromatography (PE/EtOAc = 50/1), affording the desired product **6a** (119.7 mg, 0.38 mmol, 79% yield for three steps) as a colorless solid.

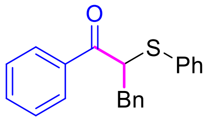

6a

1,3-Diphenyl-2-(phenylthio)propan-1-one (6a).[1] Colorless solid, 125.4 mg, 79% yield, mp: 78-81 ℃; ^{1}H NMR (400 MHz, $CDCl_3$): δ 7.82 (d, J = 7.6 Hz, 2H), 7.52-7.46 (m, 1H), 7.39-7.34 (m, 2H), 7.31-7.13 (m, 10H), 4.69 (dd, J = 8.4, 6.0 Hz, 1H), 3.40 (dd, J = 14.0, 8.4 Hz, 1H), 3.13 (dd, J = 14.0, 6.0 Hz, 1H); ^{13}C NMR (101 MHz, $CDCl_3$) δ 195.3, 138.8, 136.3, 134.7, 133.1, 132.1, 129.4, 129.1, 128.9, 128.64, 128.63, 128.60, 126.7, 53.0, 37.4; IR (KBr, cm^{-1}) 3071, 3059, 3022, 2964, 2919, 1668, 1593, 1579, 1493, 1447, 1438, 1432, 1362, 1246, 1180, 1171, 947, 747, 701, 691, 683, 659, 628, 515.

2.2 Parallel Intermolecular Competitive Reaction

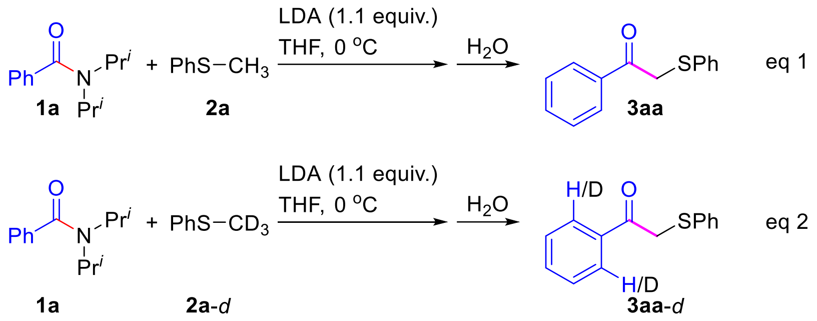

k_H/k_D = 0.4467/0.1419 = 3.2

To a 25 mL Schlenk tube equipped with a Teflon septum and magnetic stir bar were added benzamide **1a** (41.0 mg, 0.20 mmol), methyl sulfide **2a** (34.8 mg, 0.28 mmol, 1.4 equiv.), *n*-dodecane (internal standard, 15.5 mg) and THF (4 mL). The reaction tube was sealed and placed in ice-water bath. Then a mixture of LDA (23.6 mg, 0.22 mmol, 1.1 equiv., solid) and THF (1 mL) was added to the reaction mixture under nitrogen atmosphere. The tube was then sealed and continuously stirred in ice-water bath. Data were sampled and analyzed by GC for every 5 minutes. The reaction of deuterated methyl sulfide **2a**-*d* was carried out and monitored similarly.

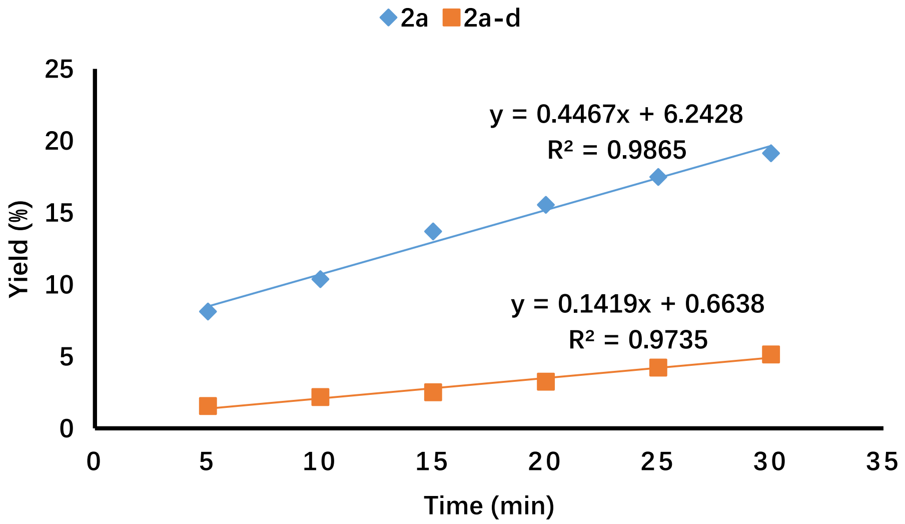

Supplementary Figure 1. Plot of yield (%) versus time (s) for kinetic isotope effect experiment with methyl sulfide **2a** and deuterated methyl sulfide **2a**-*d*.

2.3 Intramolecular Competitive Reaction

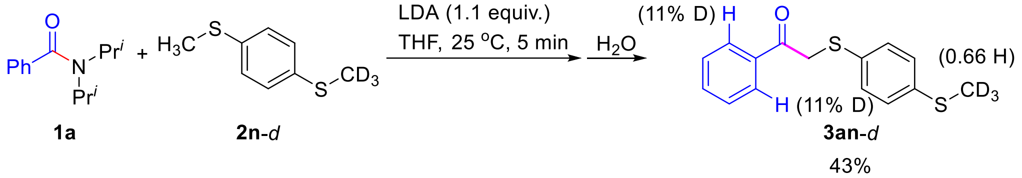

To a 25 mL Schlenk tube equipped with a Teflon septum and magnetic stir bar were added benzamide **1a** (61.6 mg, 0.30 mmol), methyl sulfide **2n**-*d* (72.9 mg, 0.42 mmol, 1.4 equiv.), THF (5.0 mL) and LDA (35.3 mg, 0.33 mmol, 1.1 equiv., solid). The tube was sealed and stirred at 25 °C for 5 min. The mixture was then quenched by adding five drops of H_2O and then diluted with ethyl acetate (EtOAc, 30 mL). The resulting mixture was dried over anhydrous Na_2SO_4. After filtration and concentration by rotary evaporation, the residue was purified by silica gel column chromatography (PE/EtOAc = 50/1) to afford the desired product **3an**-*d* (36.0 mg, 0.13 mmol, 43% yield) as a colorless oil.

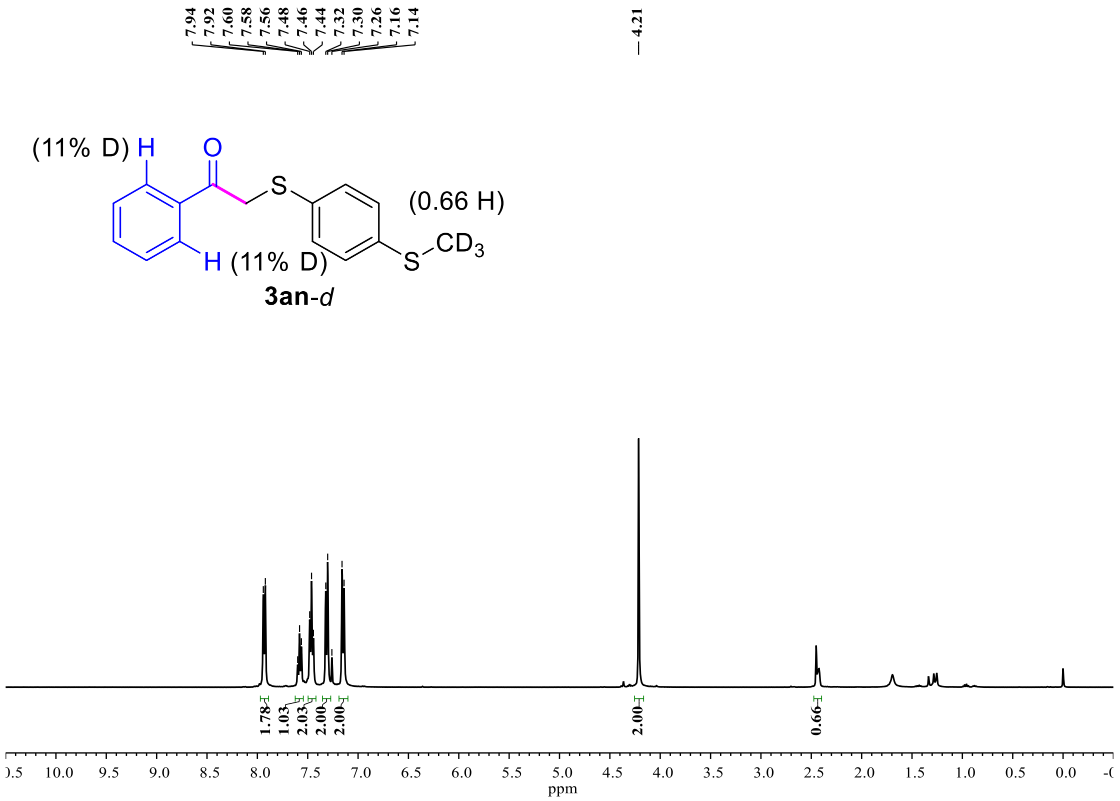

Supplementary Figure 2. ^{1}H NMR of **3an**-*d*.

2.4 Deuterium Scrambling Reaction 1

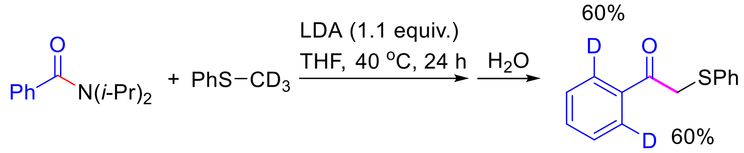

To a 25 mL Schlenk tube equipped with a Teflon septum and magnetic stir bar were added benzamide **1a** (102.7 mg, 0.50 mmol), deuterated methyl phenyl sulfide **2a-***d* (89.1 mg, 0.70 mmol, 1.4 equiv.), THF (1.0 mL) and LDA (58.9 mg, 0.55 mmol, 1.1 equiv., solid). The tube was sealed and stirred at 40 °C for 24 hours. The mixture was then cooled to room temperature, quenched by adding five drops of H_2O and then diluted with ethyl acetate (EtOAc, 30 mL). The resulting mixture was dried over anhydrous Na_2SO_4. After filtration and concentration by rotary evaporation, the residue was purified by silica gel column chromatography (PE/EtOAc = 50/1) to afford the desired product **3aa-***d* (86.7 mg, 0.38 mmol, 76% yield) as a colorless oil. No tertiary alcohol product was detected in the crude reaction mixture by ^{1}H NMR.

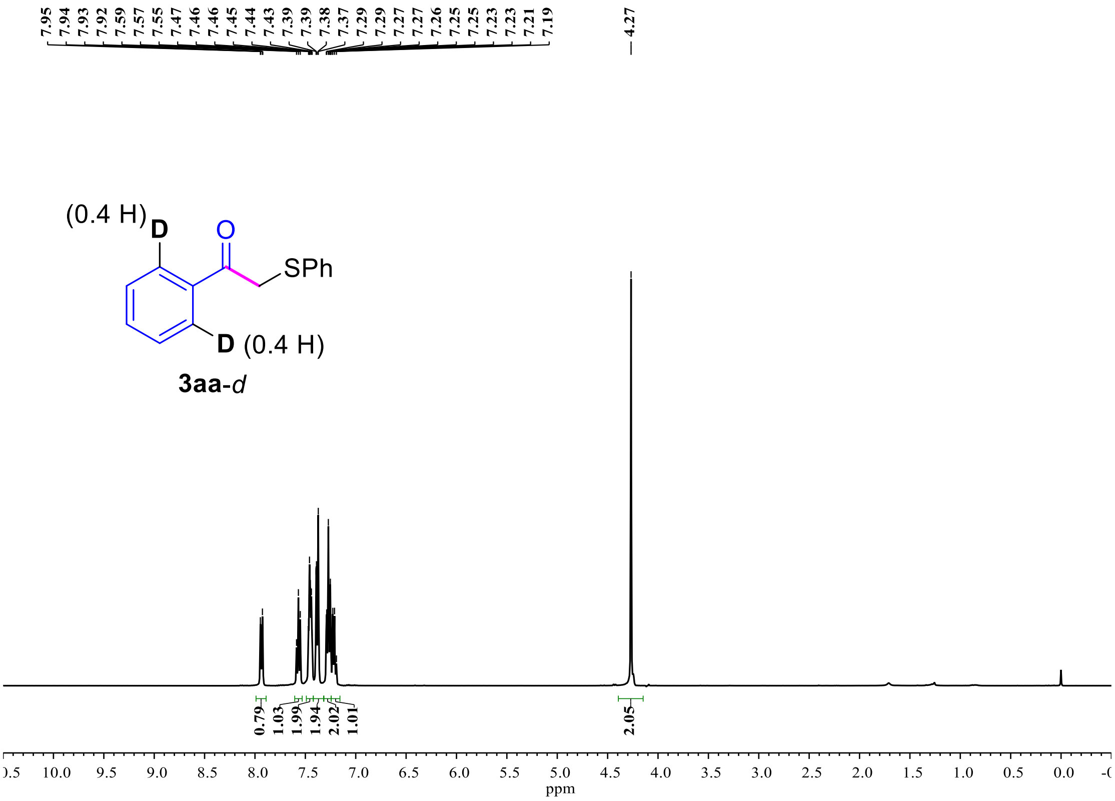

Supplementary Figure 3. ^{1}H NMR of **3aa-***d*.

2.5 Deuterium Scrambling Reaction 2

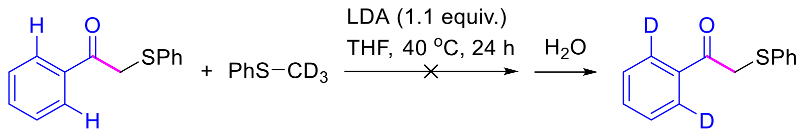

3aa **2a**-*d* **3aa**-*d*

To a 25 mL Schlenk tube equipped with a Teflon septum and magnetic stir bar were added non-deuterated ketone **3aa** (112.8 mg, 0.49 mmol), deuterated methyl phenyl sulfide **2a**-*d* (89.1 mg, 0.70 mmol, 1.4 equiv.), THF (1.0 mL) and LDA (58.9 mg, 0.55 mmol, 1.1 equiv., solid). The tube was sealed and stirred at 40 °C for 24 hours. The mixture was then cooled to room temperature, quenched by adding five drops of H_2O and then diluted with ethyl acetate (EtOAc, 30 mL). The resulting mixture was dried over anhydrous Na_2SO_4. After filtration and concentration by rotary evaporation, the residue was purified by silica gel column chromatography (PE/EtOAc = 50/1) to isolated the compound of ketone (38.8 mg, 0.21 mmol, 42% recovered) as a colorless oil. ^{1}H NMR analysis of the isolated ketone revealed that no deuterium atoms were incorporated in the isolated ketone. The above result indicated the C-H/C-D exchange in our reaction occurs before the C-C bond formation process.

2.6 Deprotonation Reaction of Sulfide

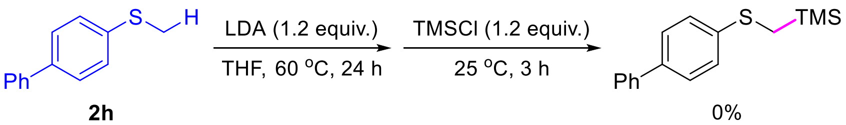

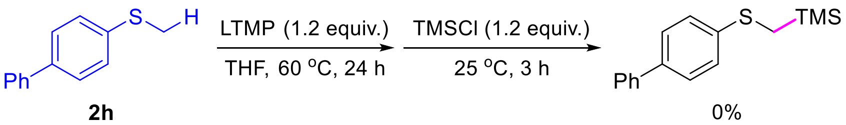

To a 25 mL Schlenk tube equipped with a Teflon septum and magnetic stir bar were added sulfide **2h** (100.1 mg, 0.50 mmol), THF (1.0 mL) and LDA (58.9 mg, 0.55 mmol, 1.1 equiv., solid). The tube was sealed and stirred at 60 °C for 24 hours. The mixture was then cooled to room temperature, and TMSCl (65.2 mg, 0.60 mmol, 1.2 equiv.) was added to the reaction mixture under an atmosphere of N_2. The reaction tube was placed at 25 °C and stirred for 3 h. Then, the resulting solution was quenched by adding five drops of H_2O and then diluted with ethyl acetate. The resulting mixture was dried over anhydrous Na_2SO_4 and an internal standard of 1,3,5-trimethoxybenzene was added. ^{1}H NMR analysis of the crude reaction mixture indicated that no TMS substituted sulfide was detected and 94% of **2h** was recovered. This is the case for the reaction with LiTMP. The above outcomes suggest that the direct deprotonation reaction of thioanisole by LDA and LiTMP are unsuccessful.

2.7 The Reaction Between *ortho*-Lithium Benzamide Complex and Thioanisole.

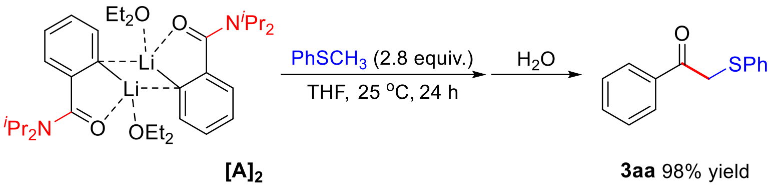

We prepared the ortho-lithiated amides **[A]2** according reported procedure.[2] Treating **[A]2** with 2.8 equiv. of thioanisole afforded ketone product **3aa** with 98% yield. The above outcome suggests intermediate **A**, as proposed, could be involved in the reaction pathway.

2.8 Kinetic Experiments

2.8.1 Kinetic studies: variable benzamide **1a** (from 0.076 M - 0.125 M).

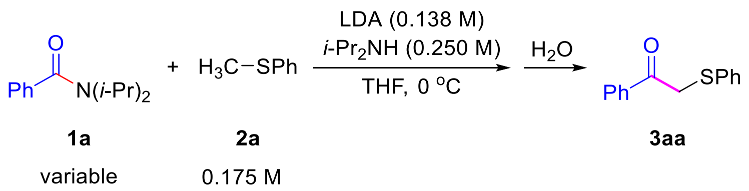

Reaction condition: [**2a**] = 0.175 M, [LDA] = 0.138 M, [*i*-Pr$_2$NH] = 0.250 M, 0 °C.

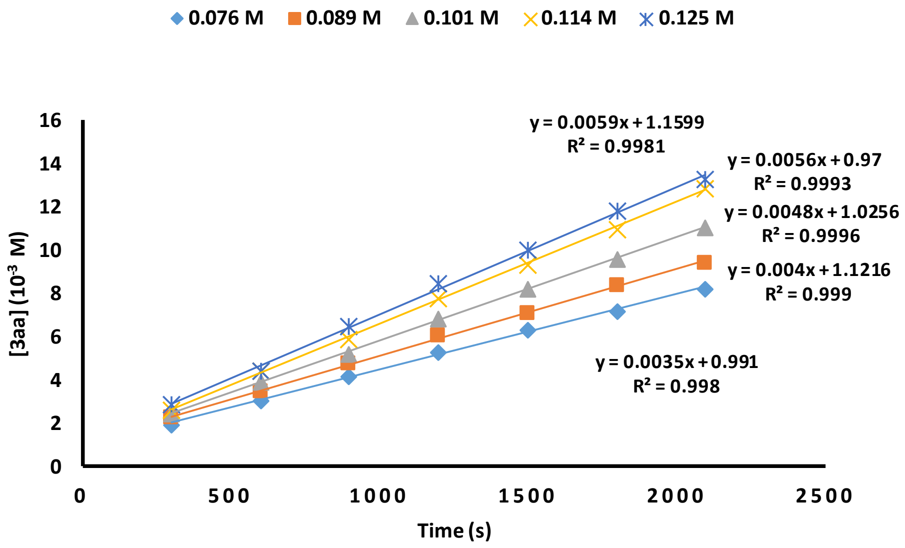

Supplementary Figure 4. Initial reaction rates with various concentrations of **1a**

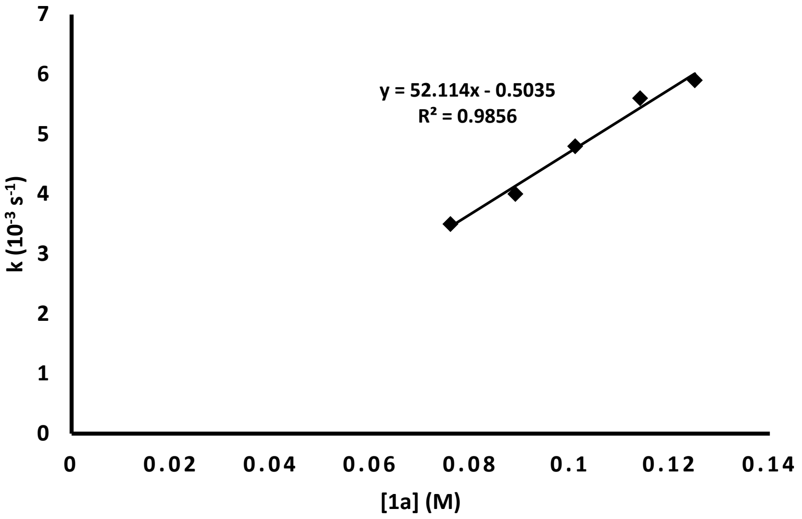

Supplementary Figure 5. Effect of concentration of **1a** on the initial rate

2.8.2 Kinetic studies: variable thioanisole **2a** (from 0.077 M - 0.174 M).

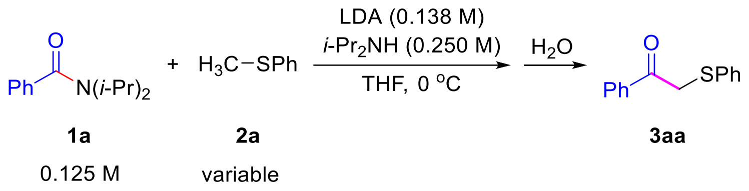

Reaction condition: [**1a**] = 0.125 M, [LDA] = 0.138 M, [*i*-Pr$_2$NH] = 0.250 M, 0 °C.

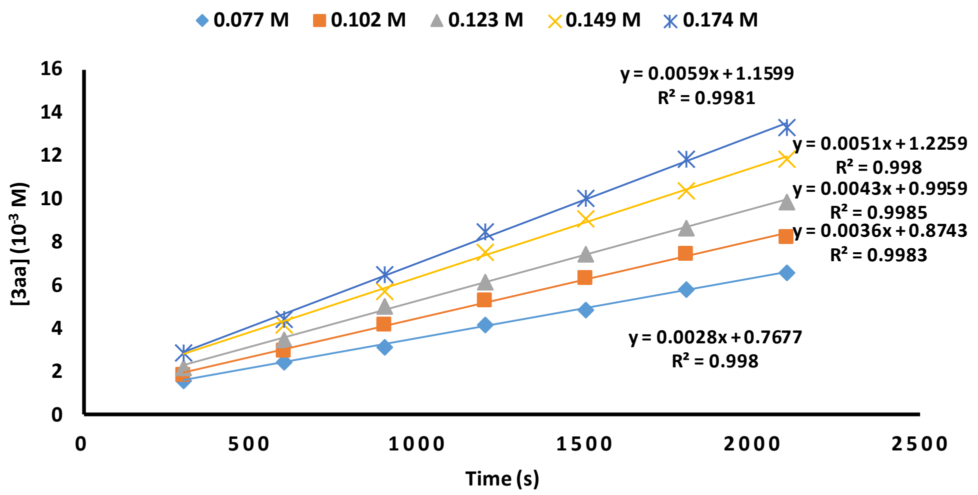

Supplementary Figure 6. Initial reaction rates with various concentrations of **2a**

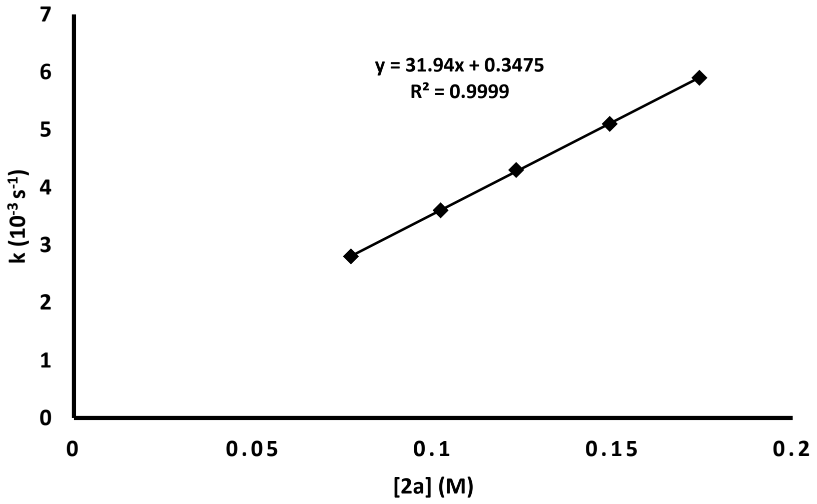

Supplementary Figure 7. Effect of concentration of **2a** on the initial rate

2.8.3 Kinetic studies: variable LDA (from 0.066 M - 0.163 M).

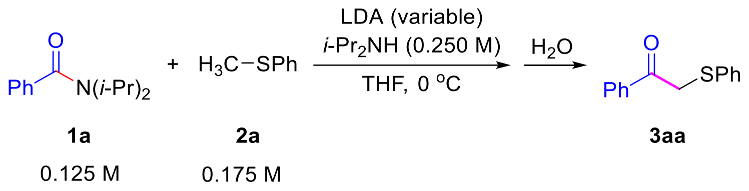

Reaction condition: [**1a**] = 0.125 M, [**2a**] = 0.175 M, [*i*-Pr$_2$NH] = 0.250 M, 0 °C.

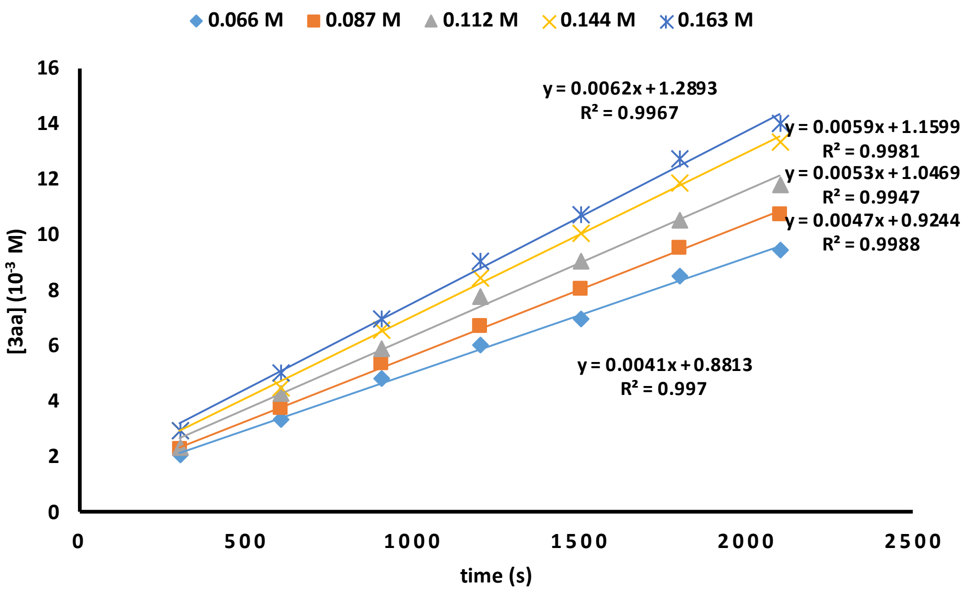

Supplementary Figure 8. Initial reaction rates with various concentrations of LDA

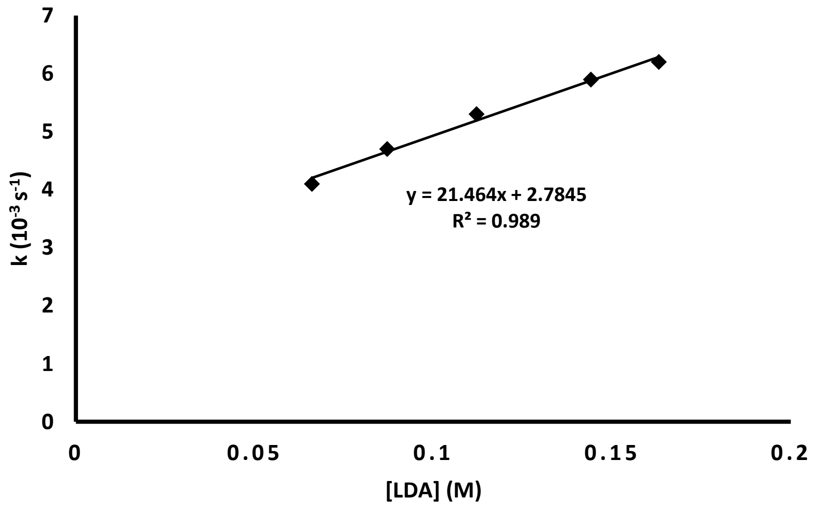

Supplementary Figure 9. Effect of concentration of LDA on the initial rate

2.8.4 Kinetic studies: variable *i*-Pr_2NH (from 0.125 M - 0.375 M).

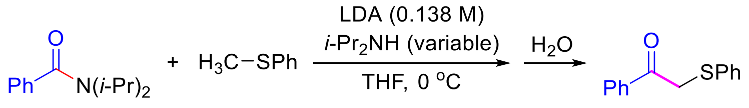

Reaction condition: [**1a**] = 0.125 M, [**2a**] = 0.175 M, [LDA] = 0.138 M, 0 °C.

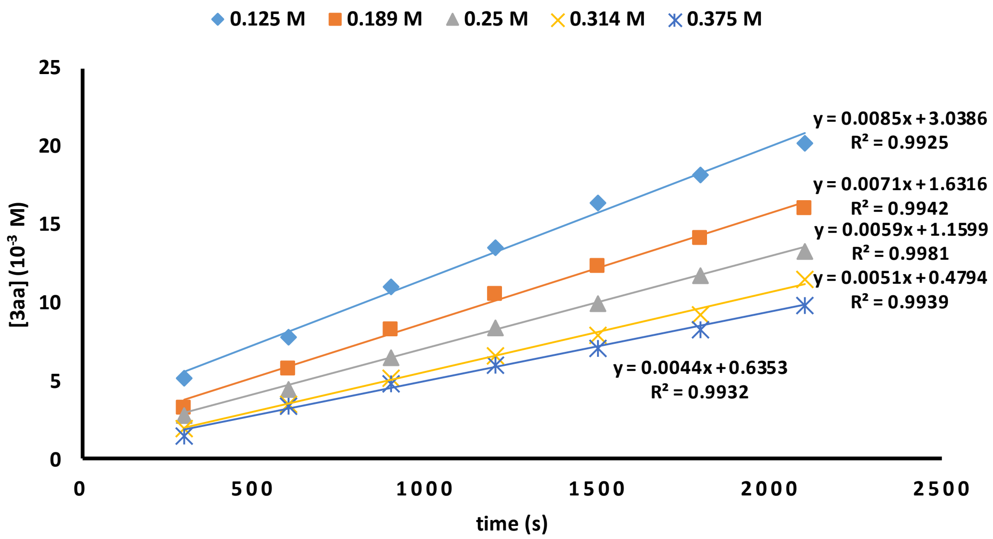

Supplementary Figure 10. Initial reaction rates with various concentrations of *i*-Pr_2NH

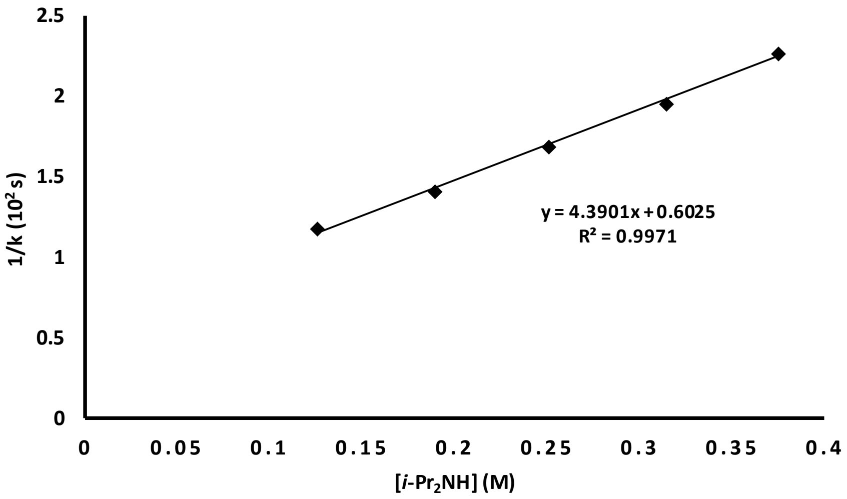

Supplementary Figure 11. Effect of concentration of *i*-Pr_2NH on the initial rate

3. General Information

All manipulations of air- and moisture-sensitive compounds were performed under a nitrogen atmosphere by use of standard Schlenk techniques or in a glovebox. THF, Et2O, t-BuOMe, CPME and hexane, were dried by distillation over sodium/benzophenone. Lithium diisopropylamide (LDA) was synthesized according to literature[3] and kept under -30 ºC in a glove box in its solid state after removing the solvents in vacuum. Several commercial LDAs (in THF/n-heptane/ethylbenzene) were used directly as received (Supplementary Table 4). Sulfides **2a**, **2e**, **2g**, **4a-c** and **4e-h** purchased from Alfa Aesar, TCI (Shanghai) Development Co., Ltd., Shanghai Aladdin Biochemical Technology Co., Ltd. and Bidepharm were used as received. Sulfides **2b**, **2c**, **2h**, **2i** and **2l** were prepared from aryl bromides according to the literature.[4] Sulfides **2d**, **2f**, **2j**, **2k**, **2m** and **2n** were synthesized from thiophenols or phenols according to the literature.[5] Sulfides **4d** and **4i-p** were prepared form alkyl halides or their analogues according to the literature.[6] Benzamides **1a**, **1b**, **1e**, **1f**, **1h**, **1j**, **1k**, **1l** and **lm** were synthesized form aroyl chlorides according to the literature.[7] Benzamides **1c**, **1d**, **1g** and **1i** were prepared from carboxylic acids according to the literature.[8]

TLC were performed on silica gel Huanghai HSGF254 plates and visualized by UV fluorescence (λ_{max} = 254 nm) and aqueous solution of $KMnO_4$. Silica gel (200-300 mesh) was purchased from Qingdao Haiyang Chemical Co., Ltd., China. ^{1}H NMR, ^{13}C NMR were recorded on a Bruker ASCEND400 instrument in $CDCl_3$ (400 MHz for ^{1}H, 101 MHz for ^{13}C). Chemical shifts (δ) are recorded in ppm relative to the residual solvent signals ($CDCl_3$: δ = 7.26 for ^{1}H, δ = 77.16 for ^{13}C). Data were reported as follows: chemical shift in ppm (δ), multiplicity (s = singlet, d = doublet, t = triplet, q = quartet, m = multiplet), coupling constant (Hz), integration. Gas chromatography (GC) data were collected from SHIMADZU GAS CHROMATOGRAPH GC-2014 AOC-20i. High resolution mass spectra (HRMS) were recorded on a Varian 7.0T FTMS with Varian QFT-ESI or Agilent GCQTOF 7200 (EI). Melting point were collected with melting point apparatus (RY-1A) made by Tianjin Analysis Instrument Factory.

4. General Procedures and Analytical Data of Compound 3 and 5

4.1 A general procedure for the alkylation of benzamide with PhSMe

To a 25 mL Schlenk tube equipped with a Teflon septum and magnetic stir bar were added benzamide **1a** (102.7 mg, 0.50 mmol), methyl phenyl sulfide **2a** (86.9 mg, 82 μL, 0.70 mmol, 1.4 equiv.), THF (1.0 mL) and LDA (58.9 mg, 0.55 mmol, 1.1 equiv., solid). The tube was sealed and stirred at 40 °C for 24 hours. The mixture was then cooled to room temperature, quenched by adding five drops of H_2O and then diluted with ethyl acetate (EtOAc, 30 mL). The resulting mixture was dried over anhydrous Na_2SO_4. After filtration and concentration by rotary evaporation, the residue was purified by silica gel column chromatography (PE/EtOAc = 50/1) to afford the desired product **3aa** (106.2 mg, 0.46 mmol, 93% yield) as a colorless oil.

4.2 A general procedure for the alkylation of benzamide with *i*-PrSMe

To a 25 mL Schlenk tube equipped with a Teflon septum and magnetic stir bar were added benzamide **1a** (102.7 mg, 0.50 mmol), isopropyl methyl sulfide **4a** (112.7 mg, 136 μL, 1.25 mmol, 2.5 equiv.), THF (1.0 mL) and LDA (80.3 mg, 0.75 mmol, 1.5 equiv., solid). The tube was sealed and stirred at 40 °C for 24 hours. The mixture was then cooled to room temperature, quenched by adding five drops of H_2O and then diluted with ethyl acetate (EtOAc, 30 mL). The resulting mixture was dried over anhydrous Na_2SO_4. After filtration and concentration by rotary evaporation, the residue was purified by silica gel column chromatography (PE/EtOAc = 50/1) to afford the desired product **5aa** (82.6 mg, 0.43 mmol, 85% yield) as a colorless oil.

4.3 A gram-scale process for the alkylation of benzamide with thioanisole

To a 50 mL Schlenk tube equipped with a Teflon septum and magnetic stir bar were added benzamide **1a** (1.03 g, 5.0 mmol), methyl phenyl sulfide **2a** (0.87 g, 825 μL, 7.0 mmol, 1.4 equiv.), THF (10 mL) and LDA (589.2 mg, 5.5 mmol, 1.1 equiv., solid). The tube was sealed and stirred at 40 °C for 24 hours. The mixture was then cooled to room temperature, quenched by saturated NH_4Cl aqueous solution (10 mL) and then diluted

with ethyl acetate (EtOAc, 50 mL). After separation, the aqueous solution was extracted thrice with EtOAc (50 mL x 3). The combined organic layer was washed with brine (50 mL), and dried over anhydrous Na_2SO_4. After filtration and concentration by rotary evaporation, the mixture was purified by silica gel column chromatography (PE/EtOAc = 50/1) to afford the desired product **3aa** (1.06 g, 4.6 mmol, 92% yield) as a colorless oil.

4.4 Analytical Data of Compound 3 and 5

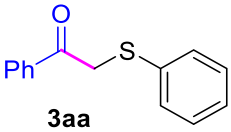

3aa

1-Phenyl-2-(phenylthio)ethan-1-one (3aa).[9] Colorless oil, 106.4 mg, 93% yield; ^{1}H NMR (400 MHz, $CDCl_3$) δ 7.98-7.89 (m, 2H), 7.59-7.53 (m, 1H), 7.47-7.41 (m, 2H), 7.41-7.34 (m, 2H), 7.30-7.23 (m, 2H), 7.23-7.17 (m, 1H), 4.26 (s, 2H); ^{13}C NMR (101 MHz, $CDCl_3$) δ 194.1, 135.4, 134.8, 133.6, 130.5, 129.1, 128.74, 128.73, 127.1, 41.2; IR (liquid film, cm^{-1}) 3060, 3004, 2926, 1679, 1597, 1580, 1480, 1449, 1439, 1276, 1199, 1068, 975, 745, 688.

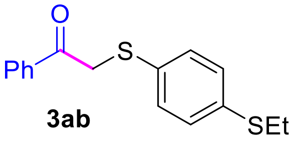

3ab

2-((4-(Ethylthio)phenyl)thio)-1-phenylethan-1-one (3ab). Pale pink oil, 138.8 mg, 96% yield; ^{1}H NMR (400 MHz, $CDCl_3$) δ 8.01-7.87 (m, 2H), 7.58 (t, J = 7.2 Hz, 1H), 7.50-7.42 (m, 2H), 7.30 (d, J = 8.4 Hz, 2H), 7.21 (d, J = 8.4 Hz, 2H), 4.24 (s, 2H), 2.92 (q, J = 7.2 Hz, 2H), 1.30 (t, J = 7.2 Hz, 3H); ^{13}C NMR (101 MHz, $CDCl_3$) δ 194.1, 136.4, 135.5, 133.6, 131.8, 131.6, 129.3, 128.8, 41.6, 27.6, 14.4; IR (liquid film, cm^{-1}) 3061, 2972, 2929, 2892, 2872, 1683, 1676, 1596, 1580, 1479, 1447, 1393, 1319, 1290, 1266, 1198, 1109, 998, 802, 749, 684, 640; HRMS (EI): m/z calcd. for $C_{16}H_{16}OS_2$ $[M]^+$: 288.0637, found: 288.0637.

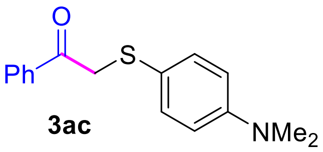

3ac

2-((4-(Dimethylamino)phenyl)thio)-1-phenylethan-1-one (3ac). Pale yellow oil, 122.9 mg, 91% yield; ^{1}H NMR (400 MHz, $CDCl_3$) δ 8.00-7.88 (m, 2H), 7.56 (t, *J* = 7.2 Hz, 1H), 7.49-7.41 (m, 2H), 7.30 (d, *J* = 8.0 Hz, 2H), 6.62 (d, *J* = 8.0 Hz, 2H), 4.07 (s, 2H), 2.95 (s, 6H); ^{13}C NMR (101 MHz, $CDCl_3$) δ 194.7, 135.7, 135.3, 133.3, 128.9, 128.7, 112.9, 43.6, 40.5; IR (liquid film, cm^{-1}) 3061, 2894, 2810, 1676, 1595, 1506, 1447, 1359, 1276, 1226, 1195, 1130, 1100, 1014, 946, 813, 723, 689; HRMS (EI): *m*/*z* calcd. for $C_{16}H_{17}NOS$ $[M]^+$: 271.1025, found: 271.1021.

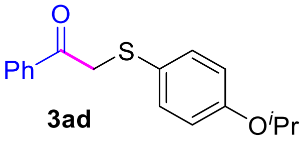

3ad

2-((4-Isopropoxyphenyl)thio)-1-phenylethan-1-one (3ad). Colorless oil, 125.2 mg, 86% yield; ^{1}H NMR (400 MHz, $CDCl_3$) δ 7.96-7.87 (m, 2H), 7.60-7.54 (m, 1H), 7.48-7.41 (m, 2H), 7.38-7.30 (m, 2H), 6.82-6.76 (m, 2H), 4.56-4.47 (m, 1H), 4.14 (s, 2H), 1.32 (d, *J* = 5.6 Hz, 6H); ^{13}C NMR (101 MHz, $CDCl_3$) δ 194.6, 158.3, 135.7, 134.8, 133.5, 128.9, 128.8, 124.3, 116.6, 70.1, 43.0, 22.1; IR (liquid film, cm^{-1}) 3063, 2977, 2933, 1679, 1592, 1490, 1449, 1385, 1281, 1245, 1182, 1117, 1103, 1016, 1001, 952, 828, 688; HRMS (ESI): *m*/*z* calcd. for $C_{17}H_{18}NaO_2S$ $[M + Na]^+$: 309.0920, found: 309.0925.

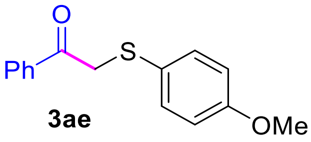

3ae

2-((4-Methoxyphenyl)thio)-1-phenylethan-1-one (3ae).[9] Colorless oil, 118.5 mg, 92% yield; ^{1}H NMR (400 MHz, $CDCl_3$) δ 7.92 (d, *J* = 7.6 Hz, 2H), 7.57 (t, *J* = 7.6 Hz, 1H), 7.51-7.42 (m, 2H), 7.36 (d, *J* = 8.8 Hz, 2H), 6.82 (d, *J* = 8.8 Hz, 2H), 4.13 (s, 2H), 3.78 (s, 3H); ^{13}C NMR (101 MHz, $CDCl_3$) δ 194.5, 159.9, 135.6, 134.8, 133.5, 128.9, 128.8, 124.7, 114.8, 55.5, 42.9; IR (liquid film, cm^{-1}) 3064, 3004, 2959, 2939, 2837, 1678,

1592, 1493, 1462, 1449, 1288, 1248, 1173, 1104, 1029, 975, 827, 688.

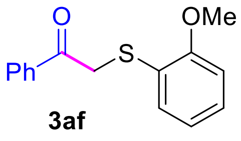

3af

2-((2-Methoxyphenyl)thio)-1-phenylethan-1-one (3af).[10] Colorless oil, 105.4 mg, 82% yield; ^{1}H NMR (400 MHz, $CDCl_3$) δ 7.85 (d, J = 7.6 Hz, 2H), 7.50-7.43 (m, 1H), 7.40-7.30 (m, 2H), 7.26 (d, J = 7.6 Hz, 1H), 7.18-7.14 (m, 1H), 6.85-6.69 (m, 2H), 4.14 (s, 2H), 3.75 (s, 3H); ^{13}C NMR (101 MHz, $CDCl_3$) δ 194.6, 158.4, 135.7, 133.4, 132.7, 129.2, 128.71, 128.66, 122.1, 121.1, 110.8, 55.8, 39.6; IR (liquid film, cm^{-1}) 3060, 2964, 2938, 2837, 1687, 1597, 1579, 1474, 1464, 1450, 1271, 1239, 1096, 1059, 1021, 803, 741, 713, 688.

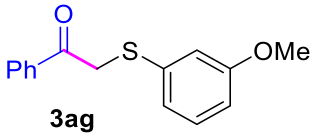

3ag

2-((3-Methoxyphenyl)thio)-1-phenylethan-1-one (3ag).[10] Colorless oil, 123.0 mg, 93% yield; ^{1}H NMR (400 MHz, $CDCl_3$) δ 8.03-7.86 (m, 2H), 7.62-7.54 (m, 1H), 7.52-7.39 (m, 2H), 7.23-7.14 (m, 1H), 7.05-6.86 (m, 2H), 6.86-6.65 (m, 1H), 4.29 (s, 2H), 3.77 (s, 3H); ^{13}C NMR (101 MHz, $CDCl_3$) δ 194.2, 159.9, 136.2, 135.5, 133.6, 130.0, 128.81, 128.79, 122.4, 115.5, 113.0, 55.4, 41.1; IR (liquid film, cm^{-1}) 3063, 3004, 2937, 2835, 1682, 1590, 1576, 1479, 1449, 1283, 1248, 1232, 1039, 974, 860, 804, 777, 687.

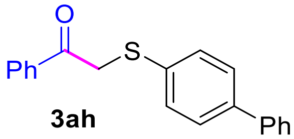

3ah

2-([1,1'-Biphenyl]-4-ylthio)-1-phenylethan-1-one (3ah). Colorless solid, 131.3 mg, 86% yield, mp: 104-107 ℃; ^{1}H NMR (400 MHz, $CDCl_3$) δ 8.04-7.92 (m, 2H), 7.62-7.42 (m, 11H), 7.38-7.33 (m, 1H), 4.32 (s, 2H); ^{13}C NMR (101 MHz, $CDCl_3$) δ 194.2, 140.4, 140.2, 135.5, 133.9, 133.6, 131.0, 128.9, 128.8, 127.8, 127.6, 127.1, 41.3; IR (KBr, cm^{-1}) 3070, 3055, 2993, 2979, 1671, 1596, 1579, 1478, 1446, 1277, 1137, 1090, 1013, 1003, 938, 826, 764, 758, 737, 724, 712, 701, 686, 647, 554; HRMS (ESI): *m*/*z*

calcd. for $C_{20}H_{16}NaOS$ $[M + Na]^+$: 327.0814, found: 327.0819.

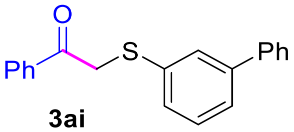

3ai

2-([1,1'-Biphenyl]-3-ylthio)-1-phenylethan-1-one (3ai). Colorless oil, 128.4 mg, 83% yield; ^{1}H NMR (400 MHz, $CDCl_3$) δ 8.00-7.93 (m, 2H), 7.62-7.53 (m, 4H), 7.49-7.41 (m, 5H), 7.39-7.32 (m, 3H), 4.34 (s, 2H); ^{13}C NMR (101 MHz, $CDCl_3$) δ 194.2, 142.2, 140.4, 135.5, 135.4, 133.6, 129.5, 129.22, 129.15, 128.9, 128.8, 127.7, 127.2, 126.1, 41.2; IR (liquid film, cm^{-1}) 3059, 3030, 2916, 2849, 1679, 1596, 1589, 1564, 1467, 1449, 1399, 1318, 1276, 1198, 1182, 1076, 1001, 991, 975, 756, 728, 698, 645, 614; HRMS (ESI): *m/z* calcd. for $C_{20}H_{16}NaOS$ $[M + Na]^+$: 327.0814, found: 327.0820.

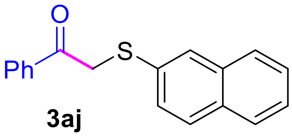

3aj

2-(Naphthalen-2-ylthio)-1-phenylethan-1-one (3aj).[9] Colorless oil, 126.4 mg, 92% yield; ^{1}H NMR (400 MHz, $CDCl_3$) δ 8.01-7.94 (m, 2H), 7.84 (s, 1H), 7.81-7.71 (m, 3H), 7.61-7.56 (m, 1H), 7.53-7.41 (m, 5H), 4.38 (s, 2H); ^{13}C NMR (101 MHz, $CDCl_3$) δ 194.2, 135.5, 133.8, 133.7, 132.32, 132.29, 129.0, 128.84, 128.83, 128.80, 128.1, 127.8, 127.5, 126.7, 126.3, 41.3; IR (liquid film, cm^{-1}) 3081, 3052, 2943, 2902, 1687, 1586, 1501, 1447, 1372, 1337, 1287, 1195, 1076, 991, 859, 815, 783, 767, 746, 738, 688, 648, 554.

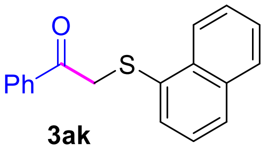

3ak

2-(Naphthalen-1-ylthio)-1-phenylethan-1-one (3ak). Colorless oil, 130.9 mg, 93% yield; ^{1}H NMR (400 MHz, $CDCl_3$) δ 8.43 (d, J = 8.4 Hz, 1H), 7.92-7.84 (m, 3H), 7.79 (d, J = 8.4 Hz, 1H), 7.65 (dd, J = 7.2, 0.8 Hz, 1H), 7.58-50 (m, 3H), 7.45-7.36 (m, 3H), 4.30 (s, 2H); ^{13}C NMR (101 MHz, $CDCl_3$) δ 194.3, 135.6, 134.1, 133.5, 133.4, 131.7, 131.3, 128.9, 128.82, 128.78, 128.7, 126.9, 126.5, 125.7, 125.2, 41.8; IR (liquid film,

cm^{-1}) 3054, 2963, 2927, 2893, 1679, 1591, 1561, 1448, 1383, 1317, 1289, 977, 783, 769, 749, 682, 665; HRMS (ESI): *m*/*z* calcd. for $C_{18}H_{14}NaOS$ [M + Na]$^+$: 301.0658, found: 301.0662.

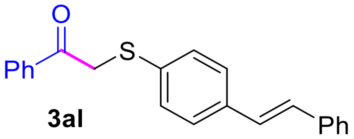

3al

(E)-1-Phenyl-2-((4-styrylphenyl)thio)ethan-1-one (3al). Colorless solid, 150.5 mg, 93% yield, mp: 118-120 ℃; ^{1}H NMR (400 MHz, $CDCl_3$) δ 8.03-7.89 (m, 2H), 7.61-7.55 (m, 1H), 7.52-7.40 (m, 6H), 7.39-7.33 (m, 4H), 7.28-7.23 (m, 1H), 7.10-7.02 (m, 2H), 4.28 (s, 2H); ^{13}C NMR (101 MHz, $CDCl_3$) δ 194.1, 137.2, 136.4, 135.5, 134.0, 133.7, 130.8, 129.2, 128.8, 127.9, 127.2, 126.7, 41.3; IR (KBr, cm^{-1}) 3073, 3052, 3019, 2923, 2880, 2852, 1675, 1596, 1588, 1580, 1495, 1447, 1394, 1280, 1186, 1090, 970, 807, 726, 710, 688, 530; HRMS (ESI): *m*/*z* calcd. for $C_{22}H_{18}NaOS$ [M + Na]$^+$: 353.0971, found: 353.0975.

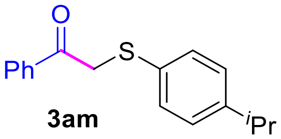

3am

2-((4-Isopropylphenyl)thio)-1-phenylethan-1-one (3am). Colorless oil, 127.5 mg, 93% yield; ^{1}H NMR (400 MHz, $CDCl_3$) δ 8.05-7.83 (m, 2H), 7.61-7.53 (m, 1H), 7.50-7.40 (m, 2H), 7.40-7.28 (m, 2H), 7.22-7.08 (m, 2H), 4.24 (s, 2H), 2.98-2.78 (m, 1H), 1.23 (d, J = 6.8 Hz, 6H); ^{13}C NMR (101 MHz, $CDCl_3$) δ 194.4, 148.5, 135.6, 133.5, 131.5, 131.4, 128.8, 128.7, 127.4, 41.9, 33.8, 24.0; IR (liquid film, cm^{-1}) 3063, 2961, 2928, 2870, 1682, 1597, 1493, 1449, 1407, 1275, 1101, 1070, 1073, 1015, 1001, 974, 825, 715, 687; HRMS (ESI): *m*/*z* calcd. for $C_{17}H_{18}NaOS$ [M + Na]$^+$: 293.0971, found: 293.0975.

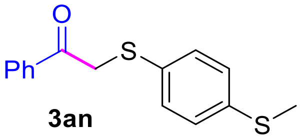

3an

2-((4-(Methylthio)phenyl)thio)-1-phenylethan-1-one (3an). Colorless solid, 89.5 mg,

65% yield, mp: 69-70 ℃; ^{1}H NMR (400 MHz, $CDCl_3$) δ 7.98-7.87 (m, 2H), 7.58 (t, *J* = 7.2 Hz, 1H), 7.49-7.42 (m, 2H), 7.31 (d, *J* = 8.0 Hz, 2H), 7.15 (d, *J* = 8.0 Hz, 2H), 4.21 (s, 2H), 2.44 (s, 3H); ^{13}C NMR (101 MHz, $CDCl_3$) δ 194.1, 138.4, 135.4, 133.6, 131.9, 130.7, 128.8, 127.0, 41.8, 15.8; IR (KBr, cm^{-1}) 3326, 3067, 2995, 2951, 2918, 1671, 1596, 1577, 1494, 1430, 1390, 1278, 1185, 1107, 1016, 1008, 967, 802, 747, 727, 686, 655, 560; HRMS (ESI): *m*/*z* calcd. for $C_{15}H_{14}NaOS_2$ $[M + Na]^+$: 297.0378, found: 297.0382.

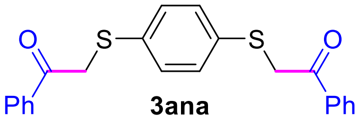

3ana

2,2'-(1,4-Phenylenebis(sulfanediyl))bis(1-phenylethan-1-one) (3ana). Colorless solid, 105.7 mg, 93% yield, mp: 125-126 ℃; ^{1}H NMR (400 MHz, $CDCl_3$) δ 7.99-7.88 (m, 4H), 7.58 (t, *J* = 7.2 Hz, 2H), 7.50-7.44 (m, 4H), 7.34-7.26 (m, 4H), 4.26 (s, 4H); ^{13}C NMR (101 MHz, $CDCl_3$) δ 194.0, 135.4, 134.0, 133.7, 131.0, 128.9, 128.8, 41.2; IR (KBr, cm^{-1}) 3067, 3058, 2921, 2901, 2850, 1676, 1596, 1579, 1481, 1447, 1389, 1321, 1279, 1192, 1110, 1009, 998, 801, 753, 685, 640, 557; HRMS (ESI): *m*/*z* calcd. for $C_{22}H_{18}NaO_2S_2$ $[M + Na]^+$: 401.0640, found: 401.0642.

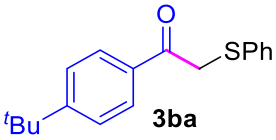

3ba

1-(4-(*Tert*-butyl)phenyl)-2-(phenylthio)ethan-1-one (3ba). Colorless oil, 132.3 mg, 95% yield; ^{1}H NMR (400 MHz, $CDCl_3$) δ 7.92-7.86 (m, 2H), 7.49-7.45 (m, 2H), 7.43-7.34 (m, 2H), 7.30-7.24 (m, 2H), 7.24-7.16 (m, 1H), 4.26 (s, 2H), 1.34 (s, 9H); ^{13}C NMR (101 MHz, $CDCl_3$) δ 193.8, 157.4, 135.1, 132.9, 130.4, 129.1, 128.8, 127.1, 125.8, 41.2, 35.3, 31.2; IR (liquid film, cm^{-1}) 3059, 2964, 2905, 2869, 1675, 1604, 1478, 1439, 1281, 1108, 1069, 999, 978, 745, 691; HRMS (ESI): *m*/*z* calcd. for $C_{18}H_{21}OS$ $[M + H]^+$: 285.1308, found: 285.1305.

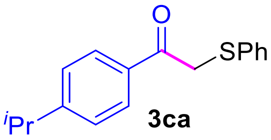

3ca

1-(4-Isopropylphenyl)-2-(phenylthio)ethan-1-one (3ca). Colorless oil, 127.3 mg, 99% yield; ^{1}H NMR (400 MHz, $CDCl_3$) δ 7.93-7.84 (m, 2H), 7.44-7.33 (m, 2H), 7.33-7.25 (m, 4H), 7.25-7.15 (m, 1H), 4.26 (s, 2H), 3.03-2.90 (m, 1H), 1.27 (d, J = 6.8 Hz, 6H); ^{13}C NMR (101 MHz, $CDCl_3$) δ 193.8, 155.2, 135.1, 133.3, 130.4, 129.2, 129.1, 127.1, 126.9, 41.2, 34.4, 23.8; IR (liquid film, cm^{-1}) 3059, 2962, 2928, 1675, 1605, 1439, 1418, 1310, 1279, 1056, 1012, 999, 743, 691; HRMS (ESI): m/z calcd. for $C_{17}H_{19}OS$ [M + H]$^+$: 271.1151, found: 271.1151.

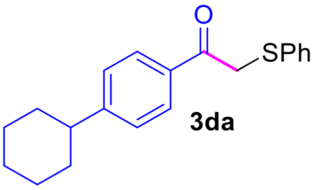

3da

1-(4-Cyclohexylphenyl)-2-(phenylthio)ethan-1-one (3da). Colorless solid, 120.3 mg, 78% yield, mp: 52-54 ℃; ^{1}H NMR (400 MHz, $CDCl_3$) δ 7.91-7.82 (m, 2H), 7.43-7.33 (m, 2H), 7.30-7.24 (m, 4H), 7.23-7.15 (m, 1H), 4.25 (s, 2H), 2.62-2.49 (m, 1H), 1.90-1.80 (m, 4H), 1.79-1.72 (m, 1H), 1.47-1.33 (m, 4H), 1.31-1.20 (m, 1H); ^{13}C NMR (101 MHz, $CDCl_3$) δ 193.8, 154.4, 135.1, 133.3, 130.4, 129.1, 129.0, 127.3, 127.1, 44.8, 41.2, 34.2, 26.8, 26.1; IR (KBr, cm^{-1}) 3058, 2925, 2851, 1686, 1603, 1584, 1481, 1451, 1369, 1293, 1213, 1189, 1175, 1089, 988, 820, 757, 731, 690, 567; HRMS (EI): m/z calcd. for $C_{20}H_{22}OS$ [M]$^+$: 310.1386, found: 310.1387.

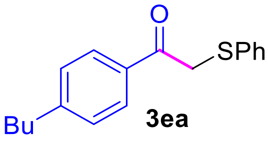

3ea

1-(4-Butylphenyl)-2-(phenylthio)ethan-1-one (3ea). Colorless oil, 102.1 mg, 72% yield; ^{1}H NMR (400 MHz, $CDCl_3$) δ 7.91-7.82 (m, 2H), 7.45-7.32 (m, 2H), 7.32-7.14 (m, 5H), 4.26 (s, 2H), 2.67 (t, J = 8.0 Hz, 2H), 1.65-1.57 (m, 2H), 1.40-1.30 (m, 2H), 0.93 (t, J = 7.6 Hz, 3H); ^{13}C NMR (101 MHz, $CDCl_3$) δ 193.9, 149.5, 135.1, 133.2, 130.5, 129.2, 129.0, 128.9, 127.1, 41.3, 35.9, 33.3, 22.4, 14.0; IR (liquid film, cm^{-1})

3059, 2956, 2930, 2859, 1675, 1605, 1466, 1439, 1416, 1280, 1179, 1069, 977, 744, 691; HRMS (ESI): *m*/*z* calcd. for $C_{18}H_{21}OS$ $[M + H]^+$: 285.1308, found: 285.1302.

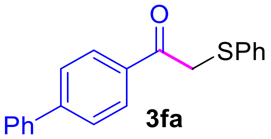

3fa

1-([1,1'-Biphenyl]-4-yl)-2-(phenylthio)ethan-1-one (3fa). Colorless solid, 136.2 mg, 89% yield, mp: 89-92 ℃; ^{1}H NMR (400 MHz, $CDCl_3$) δ 8.04-7.98 (m, 2H), 7.70-7.65 (m, 2H), 7.64-7.60 (m, 2H), 7.50-7.44 (m, 2H), 7.44-7.37 (m, 3H), 7.33-7.25 (m, 2H), 7.25-7.20 (m, 1H), 4.29 (s, 2H); ^{13}C NMR (101 MHz, $CDCl_3$) δ 193.8, 146.2, 139.8, 134.9, 134.1, 130.6, 129.4, 129.2, 129.1, 128.5, 127.40, 127.38, 127.2, 41.3; IR (KBr, cm^{-1}) 3054, 2946, 2923, 2905, 1686, 1601, 1580, 1481, 1437, 1402, 1386, 1204, 1192, 1129, 981, 846, 764, 696, 689; HRMS (ESI): *m*/*z* calcd. for $C_{20}H_{16}NaOS$ $[M + Na]^+$: 327.0814, found: 327.0820.

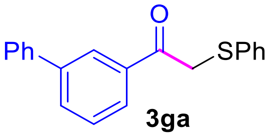

3ga

1-([1,1'-Biphenyl]-3-yl)-2-(phenylthio)ethan-1-one (3ga). Colorless oil, 130.1 mg, 89% yield; ^{1}H NMR (400 MHz, $CDCl_3$) δ 8.17-8.11 (m, 1H), 7.94-7.88 (m, 1H), 7.83-7.78 (m, 1H), 7.60-7.56 (m, 2H), 7.55-7.51 (m, 1H), 7.49-7.44 (m, 2H), 7.43-7.35 (m, 3H), 7.31-7.20 (m, 3H), 4.31 (s, 2H); ^{13}C NMR (101 MHz, $CDCl_3$) δ 194.1, 141.9, 140.0, 135.9, 134.8, 132.2, 130.7, 129.23, 129.19, 129.0, 128.0, 127.6, 127.5, 127.3, 41.4; IR (liquid film, cm^{-1}) 3059, 3032, 2926, 1682, 1583, 1478, 1453, 1439, 1301, 1074, 1025, 983, 745, 697; HRMS (ESI): *m*/*z* calcd. for $C_{20}H_{16}NaOS$ $[M + Na]^+$: 327.0814, found: 327.0819.

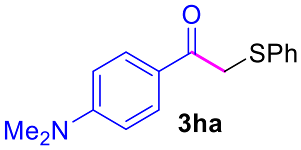

3ha

1-(4-(Dimethylamino)phenyl)-2-(phenylthio)ethan-1-one (3ha). Pale yellow oil, 116.0 mg, 85% yield; ^{1}H NMR (400 MHz, $CDCl_3$) δ 7.94-7.82 (m, 2H), 7.49-7.34 (m,

2H), 7.32-7.25 (m, 2H), 7.24-7.16 (m, 1H), 6.68-6.61 (m, 2H), 4.23 (s, 2H), 3.06 (s, 6H); ^{13}C NMR (101 MHz, $CDCl_3$) δ 192.2, 153.7, 135.9, 131.1, 130.0, 129.0, 126.7, 123.3, 110.7, 40.7, 40.1; IR (liquid film, cm^{-1}) 3056, 2908, 2817, 1655, 1597, 1549, 1530, 1481, 1438, 1374, 1322, 1293, 1231, 1191, 1122, 1024, 945, 820, 743, 691; HRMS (EI): *m*/*z* calcd. for $C_{16}H_{17}NOS$ $[M]^+$: 271.1025, found: 271.1024.

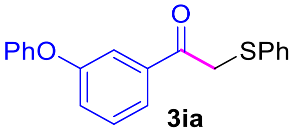

3ia

1-(3-Phenoxyphenyl)-2-(phenylthio)ethan-1-one (3ia). Colorless oil, 160.2 mg, 82% yield; ^{1}H NMR (400 MHz, $CDCl_3$) δ 7.69-7.62 (m, 1H), 7.59-7.54 (m, 1H), 7.44-7.33 (m, 5H), 7.30-7.19 (m, 4H), 7.18-7.12 (m, 1H), 7.08-6.95 (m, 2H), 4.22 (s, 2H); ^{13}C NMR (101 MHz, $CDCl_3$) δ 193.6, 158.0, 156.6, 137.3, 134.7, 130.8, 130.2, 130.1, 129.2, 127.4, 124.1, 123.7, 123.5, 119.3, 118.6, 41.4; IR (liquid film, cm^{-1}) 3063, 2916, 1682, 1581, 1489, 1456, 1439, 1273, 1236, 1164, 1071, 1024, 912, 894, 745, 691; HRMS (ESI): *m*/*z* calcd. for $C_{20}H_{16}NaO_2S$ $[M + Na]^+$: 343.0763, found: 343.0768.

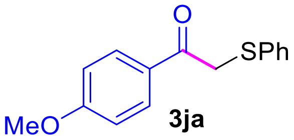

3ja

1-(4-Methoxyphenyl)-2-(phenylthio)ethan-1-one (3ja).[9] Colorless oil, 121.2 mg, 91% yield; ^{1}H NMR (400 MHz, $CDCl_3$) δ 7.98-7.88 (m, 2H), 7.46-7.34 (m, 2H), 7.30-7.24 (m, 2H), 7.24-7.14 (m, 1H), 6.95-6.89 (m, 2H), 4.23 (s, 2H), 3.86 (s, 3H); ^{13}C NMR (101 MHz, $CDCl_3$) δ 192.8, 163.9, 135.2, 131.2, 130.4, 129.2, 128.5, 127.1, 114.0, 55.6, 41.1; IR (liquid film, cm^{-1}) 3052, 3015, 2978, 2934, 2905, 1659, 1603, 1574, 1509, 1421, 1313, 1265, 1202, 1181, 1024, 994, 830, 818, 741, 690.

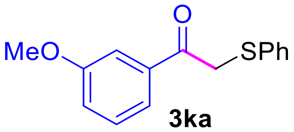

3ka

1-(3-Methoxyphenyl)-2-(phenylthio)ethan-1-one (3ka). Colorless oil, 115.5 mg, 90% yield; ^{1}H NMR (400 MHz, $CDCl_3$) δ 7.56-7.45 (m, 2H), 7.44-7.34 (m, 3H), 7.31-7.21

(m, 3H), 7.16-7.10 (m, 1H), 4.27 (s, 2H), 3.84 (s, 3H); ^{13}C NMR (101 MHz, $CDCl_3$) δ 194.0, 160.0, 136.9, 134.9, 130.7, 129.8, 129.2, 127.3, 121.4, 120.2, 113.0, 55.6, 41.4; IR (liquid film, cm^{-1}) 3074, 3060, 3004, 2960, 2940, 2836, 1679, 1597, 1582, 1487, 1465, 1438, 1279, 1222, 1069, 1024, 789, 744, 690; HRMS (ESI): *m*/*z* calcd. for $C_{15}H_{14}NaO_2S$ [M + Na]$^+$: 281.0607, found: 281.0611.

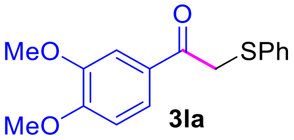

3la

1-(3,4-Dimethoxyphenyl)-2-(phenylthio)ethan-1-one (3la). Colorless oil, 136.3 mg, 92% yield; ^{1}H NMR (400 MHz, $CDCl_3$) δ 7.56 (dd, *J* = 8.4, 2.0 Hz, 1H), 7.50 (d, *J* = 2.0 Hz, 1H), 7.45-7.34 (m, 2H), 7.33-7.25 (m, 2H), 7.25-7.15 (m, 1H), 6.87 (d, *J* = 8.4 Hz, 1H), 4.24 (s, 2H), 3.94 (s, 3H), 3.90 (s, 3H); ^{13}C NMR (101 MHz, $CDCl_3$) δ 192.9, 153.7, 149.2, 135.2, 130.4, 129.1, 128.6, 127.1, 123.6, 110.8, 110.1, 56.2, 56.1, 40.8; IR (liquid film, cm^{-1}) 3078, 3059, 3004, 2961, 2936, 2839, 1668, 1594, 1585, 1515, 1464, 1296, 1273, 1151, 1022, 745, 692; HRMS (ESI): *m*/*z* calcd. for $C_{16}H_{16}NaO_3S$ [M + Na]$^+$: 311.0712, found: 311.0718.

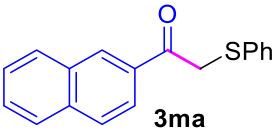

3ma

1-(Naphthalen-2-yl)-2-(phenylthio)ethan-1-one (3ma). Colorless solid, 113.7 mg, 83% yield, mp: 69-72 ℃; ^{1}H NMR (400 MHz, $CDCl_3$) δ 8.41 (s, 1H), 7.99 (dd, *J* = 8.8, 2.0 Hz, 1H), 7.91-7.83 (m, 3H), 7.61-7.57 (m, 1H), 7.55-7.50 (m, 1H), 7.45-7.37 (m, 2H), 7.31-7.18 (m, 3H), 4.38 (s, 2H); ^{13}C NMR (101 MHz, $CDCl_3$) δ 194.2, 135.8, 134.9, 132.8, 132.5, 130.73, 130.68, 129.7, 129.2, 128.8, 128.7, 127.9, 127.3, 127.0, 124.3, 41.4; IR (KBr, cm^{-1}) 3055, 2925, 1697, 1670, 1626, 1470, 1437, 1284, 1138, 1116, 1022, 957, 825, 781, 742, 689; HRMS (ESI): *m*/*z* calcd. for $C_{18}H_{14}NaOS$ [M + Na]$^+$: 301.0658, found: 301.0660.

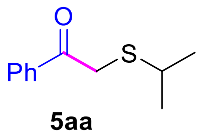

5aa

2-(Isopropylthio)-1-phenylethan-1-one (5aa).[11] Colorless oil, 82.0 mg, 85% yield; ^{1}H NMR (400 MHz, $CDCl_3$) δ 8.03 -7.92 (m, 2H), 7.60-7.53 (m, 1H), 7.50-7.42 (m, 2H), 3.83 (s, 2H), 3.07-2.90 (m, 1H), 1.27 (d, J = 6.8 Hz, 6H); ^{13}C NMR (101 MHz, $CDCl_3$) δ 195.1, 135.4, 133.4, 128.9, 128.7, 36.4, 35.6, 23.0; IR (liquid film, cm^{-1}) 3062, 2962, 2925, 2866, 1677, 1598, 1580, 1530, 1449, 1384, 1367, 1307, 1278, 1247, 1068, 1015, 971, 726, 688.

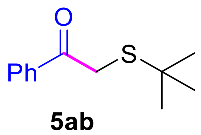

5ab

2-(*Tert*-butylthio)-1-phenylethan-1-one (5ab). Colorless oil, 92.7 mg, 89% yield; ^{1}H NMR (400 MHz, $CDCl_3$) δ 7.95 (d, J = 8.0 Hz, 2H), 7.62-7.51 (m, 1H), 7.51-7.36 (m, 2H), 3.88 (s, 2H), 1.35 (s, 9H); ^{13}C NMR (101 MHz, $CDCl_3$) δ 196.4, 135.7, 133.4, 128.9, 128.7, 43.8, 35.8, 30.8; IR (liquid film, cm^{-1}) 3063, 2963, 2923, 2898, 1678, 1597, 1449, 1364, 1276, 1219, 1165, 1111, 1014, 1001, 971, 719, 688; HRMS (ESI): *m*/*z* calcd. for $C_{12}H_{16}NaOS$ [M + Na]$^{+}$: 231.0814, found: 231.0820.

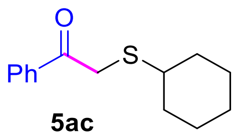

5ac

2-(Cyclohexylthio)-1-phenylethan-1-one (5ac). Colorless oil, 93.1 mg, 79% yield; ^{1}H NMR (400 MHz, $CDCl_3$) δ 7.97 (d, J = 7.6 Hz, 2H), 7.61-7.53 (m, 1H), 7.53-7.40 (m, 2H), 3.82 (s, 2H), 2.82-2.65 (m, 1H), 2.05-1.93 (m, 2H), 1.78-1.70 (m, 2H), 1.44-1.13 (m, 6H); ^{13}C NMR (101 MHz, $CDCl_3$) δ 195.3, 135.4, 133.4, 128.9, 128.7, 44.0, 35.8, 33.2, 26.0, 25.9; IR (liquid film, cm^{-1}) 3063, 2929, 2852, 1678, 1597, 1579, 1449, 1278, 1179, 1099, 1071, 1017, 999, 971, 887, 845, 817, 720, 687; HRMS (ESI): *m*/*z* calcd. for $C_{14}H_{18}NaOS$ [M + Na]$^{+}$: 257.0971, found: 257.0975.

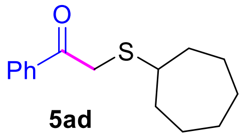

5ad

2-(Cycloheptylthio)-1-phenylethan-1-one (5ad). Colorless oil, 94.5 mg, 76% yield; ^{1}H NMR (400 MHz, $CDCl_3$) δ 8.04-7.91 (m, 2H), 7.56 (tt, J = 7.2, 1.6 Hz, 1H), 7.50-7.40 (m, 2H), 3.79 (s, 2H), 3.03-2.81 (m, 1H), 2.05-1.95 (m, 2H), 1.72-1.63 (m, 2H), 1.61-1.40 (m, 8H); ^{13}C NMR (101 MHz, $CDCl_3$) δ 195.1, 135.4, 133.3, 128.9, 128.7, 45.5, 36.6, 34.5, 28.4, 25.8; IR (liquid film, cm^{-1}) 3061, 2926, 2854, 1675, 1598, 1580, 1448, 1315, 1276, 1197, 1181, 1159, 1071, 1014, 972, 804, 726, 688; HRMS (EI): *m*/*z* calcd. for $C_{15}H_{20}OS$ $[M]^+$: 248.1229, found: 248.1228.

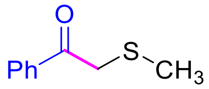

5ae

2-(Methylthio)-1-phenylethan-1-one (5ae).[12] Colorless oil, 58.2 mg, 70% yield; ^{1}H NMR (400 MHz, $CDCl_3$) δ 8.01-7.94 (m, 2H), 7.57 (tt, J = 7.2, 1.6 Hz, 1H), 7.50-7.43 (m, 2H), 3.76 (s, 2H), 2.14 (s, 3H); ^{13}C NMR (101 MHz, $CDCl_3$) δ 194.2, 135.3, 133.4, 128.9, 128.8, 39.2, 16.0; IR (liquid film, cm^{-1}) 3062, 2981, 2920, 1674, 1597, 1580, 1492, 1449, 1317, 1279, 1199, 1182, 1073, 1019, 1001, 979, 808, 729, 704, 687, 646.

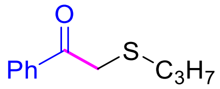

5af

1-Phenyl-2-(propylthio)ethan-1-one (5af).[9] Colorless oil, 65.4 mg, 67% yield; ^{1}H NMR (400 MHz, $CDCl_3$) δ 8.01-7.92 (m, 2H), 7.59-7.52 (m, 1H), 7.50-7.42 (m, 2H), 3.77 (s, 2H), 2.53 (t, J = 7.3 Hz, 2H), 1.66-1.56 (m, 2H), 0.96 (t, J = 7.6 Hz, 3H); ^{13}C NMR (101 MHz, $CDCl_3$) δ 194.7, 135.3, 133.4, 128.9, 128.7, 37.1, 34.4, 22.4, 13.4; IR (liquid film, cm^{-1}) 3061, 2963, 2931, 2872, 1675, 1598, 1580, 1448, 1417, 1377, 1315, 1277, 1197, 1182, 1138, 1075, 1014, 974, 806, 728, 689, 647, 563.

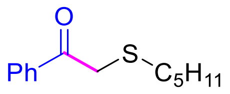

5ag

2-(Pentylthio)-1-phenylethan-1-one (5ag). Colorless oil, 70.3 mg, 65% yield; ^{1}H NMR (400 MHz, $CDCl_3$) δ 8.02-7.93 (m, 2H), 7.60-7.54 (m, 1H), 7.51-7.43 (m, 2H), 3.78 (s, 2H), 2.56 (t, J = 7.2 Hz, 2H), 1.63-1.55 (m, 2H), 1.38-1.26 (m, 4H), 0.88 (t, J = 6.4 Hz, 3H); ^{13}C NMR (101 MHz, $CDCl_3$) δ 194.7, 135.4, 133.4, 128.9, 128.8, 37.3, 32.5, 31.0, 28.8, 22.4, 14.1; IR (liquid film, cm^{-1}) 3061, 2956, 2928, 2871, 2858, 1675, 1598, 1580, 1466, 1448, 1417, 1315, 1277, 1197, 1182, 1137, 1075, 1015, 805, 749, 727, 689, 647, 563; HRMS (ESI): m/z calcd. for $C_{13}H_{18}NaOS$ $[M]^{+}$: 245.0971, found: 245.0975.

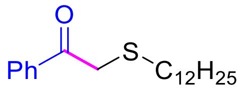

5ah

2-(Dodecylthio)-1-phenylethan-1-one (5ah).[9] Colorless oil, 122.1 mg, 76% yield; ^{1}H NMR (400 MHz, $CDCl_3$): δ 7.97 (d, J = 7.6 Hz, 2H), 7.61-7.53 (m, 1H), 7.50-7.42 (m, 2H), 3.77 (s, 2H), 2.55 (t, J = 7.6 Hz, 2H), 1.62-1.54 (m, 2H), 1.36-1.23 (m, 18H), 0.88 (t, J = 6.8 Hz, 3H); ^{13}C NMR (101 MHz, $CDCl_3$) δ 194.7, 135.4, 133.4, 128.9, 128.8, 37.2, 32.5, 32.0, 29.8, 29.7, 29.6, 29.5, 29.3, 29.1, 28.9, 22.8, 14.2; IR (KBr, cm^{-1}) 3061, 2954, 2923, 2852, 1673, 1597, 1580, 1490, 1467, 1450, 1378, 1275, 1176, 1096, 1076, 978, 832, 720, 685, 655.

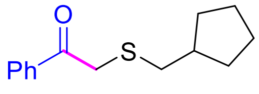

5ai

2-((Cyclopentylmethyl)thio)-1-phenylethan-1-one (5ai). Colorless oil, 73.5 mg, 63% yield; ^{1}H NMR (400 MHz, $CDCl_3$) δ 8.03-7.92 (m, 2H), 7.61-7.53 (m, 1H), 7.50-7.41 (m, 2H), 3.78 (s, 2H), 2.57 (d, J = 7.6 Hz, 2H), 2.14-1.99 (m, 1H), 1.84-1.75 (m, 2H), 1.63-1.47 (m, 4H), 1.26-1.16 (m, 2H); ^{13}C NMR (101 MHz, $CDCl_3$) δ 194.7, 135.4, 133.4, 128.9, 128.8, 39.4, 38.7, 37.7, 32.4, 25.3; IR (liquid film, cm^{-1}) 3062, 2924, 2851,

1675, 1598, 1580, 1492, 1448, 1415, 1316, 1278, 1182, 1070, 1014, 974, 928, 893, 806, 719, 688, 646; HRMS (EI): *m/z* calcd. for $C_{14}H_{18}OS$ $[M]^+$: 234.1073, found: 234.1070.

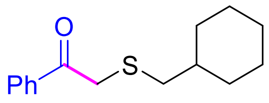

5aj

2-((Cyclohexylmethyl)thio)-1-phenylethan-1-one (5aj). Colorless oil, 87.7 mg, 71% yield; ^{1}H NMR (400 MHz, $CDCl_3$) δ 8.06-7.88 (m, 2H), 7.62-7.51 (m, 1H), 7.51-7.41 (m, 2H), 3.75 (s, 2H), 2.45 (d, *J* = 6.8 Hz, 2H), 1.86-1.77 (m, 2H), 1.72-1.61 (m, 3H), 1.54-1.42 (m, 1H), 1.26-1.06 (m, 3H), 0.98-0.86 (m, 2H); ^{13}C NMR (101 MHz, $CDCl_3$) δ 194.7, 135.4, 133.4, 128.9, 128.7, 39.9, 37.7, 37.4, 32.8, 26.4, 26.1; IR (liquid film, cm^{-1}) 3062, 2924, 2851, 1675, 1598, 1580, 1492, 1448, 1415, 1316, 1278, 1182, 1070, 1014, 974, 928, 893, 806, 719, 688, 646; HRMS (EI): *m/z* calcd. for $C_{15}H_{20}OS$ $[M]^+$: 248.1229, found: 248.1223.

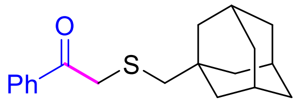

5ak

2-((((3r,5r,7r)-Adamantan-1-yl)methyl)thio)-1-phenylethan-1-one (5ak). Colorless oil, 95.1 mg, 63% yield; ^{1}H NMR (400 MHz, $CDCl_3$) δ 8.06-7.89 (m, 2H), 7.61-7.53 (m, 1H), 7.53-7.40 (m, 2H), 3.75 (s, 2H), 2.40 (s, 2H), 1.98-1.91 (m, 3H), 1.63 (dd, *J* = 31.6, 12 Hz, 6H), 1.53 (d, *J* = 2.8 Hz, 6H); ^{13}C NMR (101 MHz, $CDCl_3$) δ 194.8, 135.4, 133.3, 128.9, 128.7, 47.4, 41.8, 39.0, 36.9, 34.1, 28.6; IR (liquid film, cm^{-1}) 3061, 2902, 2846, 1675, 1598, 1580, 1449, 1360, 1344, 1316, 1276, 1196, 1181, 1099, 1015, 976, 725, 688; HRMS (EI): *m/z* calcd. for $C_{19}H_{24}OS$ $[M]^+$: 300.1542, found: 300.1546.

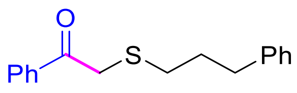

5al

1-Phenyl-2-((3-phenylpropyl)thio)ethan-1-one (5al).[13] Colorless oil, 92.6 mg, 69% yield; ^{1}H NMR (400 MHz, $CDCl_3$) δ 8.02-7.89 (m, 2H), 7.57 (t, *J* = 7.6 Hz, 1H), 7.50-7.42 (m, 2H), 7.30-7.22 (m, 2H), 7.20-7.09 (m, 3H), 3.78 (s, 2H), 2.68 (t, *J* = 7.6 Hz,

2H), 2.58 (t, $J = 7.2$ Hz, 2H), 1.91 (tt, $J = 7.6, 7.2$ Hz, 2H); ^{13}C NMR (101 MHz, $CDCl_3$) δ 194.6, 141.4, 135.3, 133.4, 128.9, 128.8, 128.6, 128.5, 126.0, 37.2, 34.8, 31.8, 30.6; IR (liquid film, cm^{-1}) 3061, 3026, 2927, 2855, 1675, 1598, 1580, 1496, 1449, 1416, 1277, 1198, 1181, 1075, 1015, 974, 746, 700, 688.

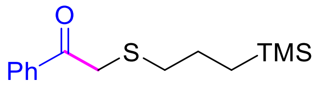

5am

1-Phenyl-2-((3-(trimethylsilyl)propyl)thio)ethan-1-one (5am). Colorless oil, 94.5 mg, 70% yield; ^{1}H NMR (400 MHz, $CDCl_3$) δ 8.04-7.91 (m, 2H), 7.62-7.52 (m, 1H), 7.51-7.41 (m, 2H), 3.77 (s, 2H), 2.57 (t, $J = 7.2$ Hz, 2H), 1.64-1.52 (m, 2H), 0.61-0.51 (m, 2H), -0.03 (s, 9H); ^{13}C NMR (101 MHz, $CDCl_3$) δ 194.7, 135.3, 133.4, 128.9, 128.8, 37.1, 36.1, 23.9, 16.3, -1.6; IR (liquid film, cm^{-1}) 3063, 2952, 2921, 1676, 1598, 1580, 1492, 1449, 1415, 1306, 1277, 1248, 1415, 1306, 1277, 1248, 1197, 1181, 1016, 1001, 975, 863, 837, 727, 689, 646; HRMS (EI): *m*/*z* calcd. for $C_{14}H_{22}OSSi$ $[M]^+$: 266.1155, found: 266.1158.

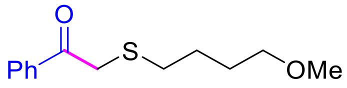

5an

2-((4-Methoxybutyl)thio)-1-phenylethan-1-one (5an). Colorless oil, 74.6 mg, 63% yield; ^{1}H NMR (400 MHz, $CDCl_3$) δ 7.98 (d, $J = 7.2$ Hz, 2H), 7.58 (t, $J = 7.2$ Hz, 1H), 7.52-7.42 (m, 2H), 3.79 (s, 2H), 3.36 (t, $J = 6.0$ Hz, 2H), 3.31 (s, 3H), 2.59 (t, $J = 6.8$ Hz, 2H), 1.70-1.61 (m, 4H); ^{13}C NMR (101 MHz, $CDCl_3$) δ 194.6, 135.3, 133.4, 128.8, 128.7, 72.2, 58.6, 37.1, 32.1, 28.7, 25.7; IR (liquid film, cm^{-1}) 3060, 2930, 2866, 2827, 1675, 1630, 1598, 1580, 1448, 1418, 1388, 1315, 1277, 1198, 1117, 1014, 885, 806, 728, 689; HRMS (EI): *m*/*z* calcd. for $C_{13}H_{18}O_2S$ $[M]^+$: 238.1022, found: 238.1024.

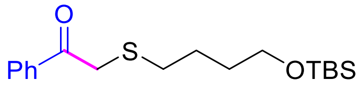

5ao

2-((4-((*Tert*-butyldimethylsilyl)oxy)butyl)thio)-1-phenylethan-1-one (5ao).

Colorless oil, 105.7 mg, 62% yield; ^{1}H NMR (400 MHz, $CDCl_3$) δ 8.01-7.91 (m, 2H), 7.56 (tt, *J* = 7.2, 2.0 Hz, 1H), 7.51-7.39 (m, 2H), 3.77 (s, 2H), 3.59 (t, *J* = 6.4 Hz, 2H), 2.58 (t, *J* = 7.2 Hz, 2H), 1.69-1.54 (m, 4H), 0.91-0.84 (m, 9H), 0.03 (s, 6H); ^{13}C NMR (101 MHz, $CDCl_3$) δ 194.6, 135.3, 133.4, 128.9, 128.7, 62.6, 37.1, 32.3, 31.9, 26.0, 25.4, 18.4, -5.2; IR (liquid film, cm^{-1}) 3060, 2953, 2929,2857, 1676, 1598, 1581, 1472, 1449, 1388, 1277, 1256, 1102, 1014, 837, 776, 727, 688; HRMS (EI): *m*/*z* calcd. For $C_{18}H_{30}O_2SSi$ $[M]^+$: 338.1730, found: 338.1735.

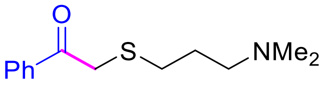

5ap

2-((3-(Dimethylamino)propyl)thio)-1-phenylethan-1-one (5ap). Colorless oil, 98.3 mg, 83% yield; ^{1}H NMR (400 MHz, $CDCl_3$) δ 8.02-7.89 (m, 2H), 7.59-7.50 (m, 1H), 7.50-7.39 (m, 2H), 3.78 (s, 2H), 3.58 (t, *J* = 7.2 Hz, 2H), 2.30 (t, *J* = 7.2 Hz, 2H), 2.18 (s, 6H), 1.74 (tt, *J* = 7.2, 7.2 Hz, 2H); ^{13}C NMR (101 MHz, $CDCl_3$) δ 194.5, 135.3, 133.4, 128.8, 128.7, 58.4, 45.5, 37.2, 30.3, 27.1; IR (liquid film, cm^{-1}) 3060, 2940, 2858, 2816, 2767, 1675, 1598, 1580, 1448, 1377, 1277, 1199, 1042, 1013, 727, 689; HRMS (EI): *m*/*z* calcd. For $C_{13}H_{19}NOS$ $[M]^+$: 237.1182, found: 237.1185.

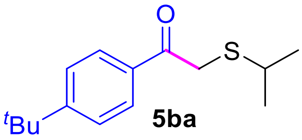

5ba

1-(4-(*Tert*-butyl)phenyl)-2-(isopropylthio)ethan-1-one (5ba). Colorless oil, 100.9 mg, 81% yield; ^{1}H NMR (400 MHz, $CDCl_3$) δ 7.92 (d, *J* = 8.4 Hz, 2H), 7.48 (d, *J* = 8.4 Hz, 2H), 3.81 (s, 2H), 3.10-2.87 (m, 1H), 1.34 (s, 9H), 1.28 (d, *J* = 6.4 Hz, 6H); ^{13}C NMR (101 MHz, $CDCl_3$) δ 194.8, 157.2, 132.8, 128.9, 125.7, 36.3, 35.6, 35.3, 31.2, 23.1; IR (liquid film, cm^{-1}) 3060, 2940, 2858, 2816, 2767, 1675, 1598, 1580, 1448, 1377, 1277, 1199, 1042, 1013, 727, 689; HRMS (EI): *m*/*z* calcd. For $C_{15}H_{22}OS$ $[M]^+$: 250.1386, found: 250.1385.

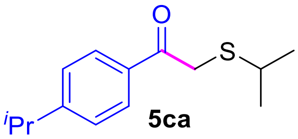

1-(4-Isopropylphenyl)-2-(isopropylthio)ethan-1-one (5ca). Colorless oil, 86.6 mg, 73% yield; ^{1}H NMR (400 MHz, $CDCl_3$) δ 7.91 (d, J = 8.4 Hz, 2H), 7.31 (d, J = 8.4 Hz, 2H), 3.80 (s, 2H), 3.13-2.84 (m, 2H), 1.30-1.24 (m, 12H); ^{13}C NMR (101 MHz, $CDCl_3$) δ 194.8, 154.9, 133.3, 129.1, 126.8, 36.3, 35.6, 34.4, 23.7, 23.0; IR (liquid film, cm^{-1}) 3049, 3030, 2962, 2928, 2869, 1674, 1606, 1569, 1463, 1419, 1384, 1365, 1310, 1279, 1186, 1055, 1011, 974, 851, 826, 781; HRMS (EI): *m*/*z* calcd. For $C_{14}H_{20}OS$ $[M]^+$: 236.1229, found: 236.1229.

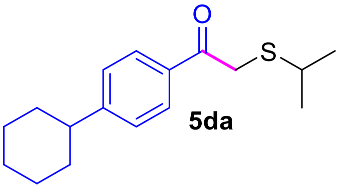

1-(4-Cyclohexylphenyl)-2-(isopropylthio)ethan-1-one (5da). Colorless oil, 108.4 mg, 79% yield; ^{1}H NMR (400 MHz, $CDCl_3$) δ 7.90 (d, J = 8.4 Hz, 2H), 7.29 (d, J = 8.4 Hz, 2H), 3.80 (s, 2H), 3.14-2.88 (m, 1H), 2.63-2.51 (m, 1H), 1.93-1.81 (m, 4H), 1.79-1.71 (m, 1H), 1.49-1.34 (m, 4H), 1.33-1.20 (m, 7H); ^{13}C NMR (101 MHz, $CDCl_3$) δ 194.8, 154.1, 133.2, 129.1, 127.2, 44.8, 36.3, 35.6, 34.2, 26.8, 26.1, 23.1; IR (liquid film, cm^{-1}) 3030, 2926, 2852, 1672, 1605, 1569, 1449, 1419, 1383, 1366, 1310, 1279, 1247, 1185, 1155, 1056, 1026, 1011, 999, 844, 775, 714, 657; HRMS (EI): *m*/*z* calcd. For $C_{17}H_{24}OS$ $[M]^+$: 276.1542, found: 276.1546.

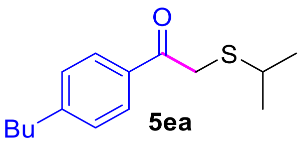

1-(4-Butylphenyl)-2-(isopropylthio)ethan-1-one (5ea). Colorless oil, 43.5 mg, 36% yield; ^{1}H NMR (400 MHz, $CDCl_3$) δ 8.00-7.80 (m, 2H), 7.35-7.21 (m, 2H), 3.81 (s, 2H), 3.08-2.90 (m, 1H), 2.67 (t, J = 8.0 Hz, 2H), 1.66-1.57 (m, 2H), 1.41-1.32 (m, 2H), 1.28 (d, J = 6.8 Hz, 6H), 0.93 (t, J = 7.4 Hz, 3H); ^{13}C NMR (101 MHz, $CDCl_3$) δ 194.9, 149.2, 133.1, 129.0, 128.8, 36.3, 35.8, 35.6, 33.3, 23.1, 22.5, 14.0; IR (liquid film, cm-

^{1}) 3030, 2926, 2852, 1672, 1605, 1569, 1449, 1419, 1383, 1366, 1311, 1279, 1185, 1155, 1056, 1026, 1011, 999, 844, 775, 714, 657; HRMS (EI): *m*/*z* calcd. For $C_{15}H_{22}OS$ $[M]^+$: 250.1386, found: 250.1390.

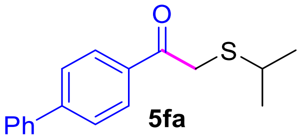

5fa

1-([1,1'-Biphenyl]-4-yl)-2-(isopropylthio)ethan-1-one (5fa). Colorless solid, 107.2 mg, 80% yield, mp: 66-68 ℃; ^{1}H NMR (400 MHz, $CDCl_3$) δ 8.06 (d, *J* = 8.4 Hz, 2H), 7.71-7.67 (m, 2H), 7.66-7.61 (m, 2H), 7.50-7.44 (m, 2H), 7.43-7.37 (m, 1H), 3.86 (s, 2H), 3.11-2.93 (m, 1H), 1.31 (d, *J* = 6.8 Hz, 6H); ^{13}C NMR (101 MHz, $CDCl_3$) δ 194.7, 146.0, 139.9, 134.1, 129.5, 129.1, 128.4, 127.37, 127.37, 36.4, 35.6, 23.1; IR (KBr, cm^{-1}) 3059, 2979, 2965, 2936, 2870, 1671, 1603, 1581, 1486, 1449, 1418, 1361, 1315, 1289, 1273, 1247, 1182, 1156, 1056, 1027, 1004, 856, 755, 692, 680, 569; HRMS (EI): *m*/*z* calcd. For $C_{17}H_{18}OS$ $[M]^+$: 270.1073, found: 270.1075.

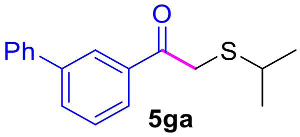

5ga

1-([1,1'-Biphenyl]-3-yl)-2-(isopropylthio)ethan-1-one (5ga). Colorless oil, 125.7 mg, 93% yield; ^{1}H NMR (400 MHz, $CDCl_3$) δ 8.21 (s, 1H), 7.99-7.92 (m, 1H), 7.83-7.77 (m, 1H), 7.65-7.60 (m, 2H), 7.55 (t, *J* = 8.0 Hz, 1H), 7.50-7.44 (m, 2H), 7.43-7.35 (m, 1H), 3.88 (s, 2H), 3.17-2.87 (m, 1H), 1.30 (d, *J* = 6.8 Hz, 6H); ^{13}C NMR (101 MHz, $CDCl_3$) δ 195.1, 141.9, 140.2, 135.9, 132.1, 129.2, 129.1, 128.0, 127.7, 127.6, 127.3, 36.5, 35.7, 23.1; IR (liquid film, cm^{-1}) 3061, 3032, 2961, 2925, 2866, 1675, 1598, 1584, 1478, 1453, 1421, 1383, 1307, 1245, 1179, 1156, 1053, 1033, 1015, 905, 813, 752, 698, 614; HRMS (EI): *m*/*z* calcd. For $C_{17}H_{18}OS$ $[M]^+$: 270.1073, found: 270.1073.

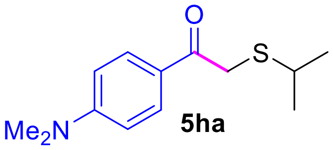

5ha

1-(4-(Dimethylamino)phenyl)-2-(isopropylthio)ethan-1-one (5ha). Colorless oil,

105.1 mg, 89% yield; ^{1}H NMR (400 MHz, $CDCl_3$) δ 7.87 (d, J = 9.2 Hz, 2H), 6.63 (d, J = 9.2 Hz, 2H), 3.74 (s, 2H), 3.16-2.93 (m, 7H), 1.26 (d, J = 6.8 Hz, 6H); ^{13}C NMR (101 MHz, $CDCl_3$) δ 193.5, 153.5, 131.1, 123.2, 110.7, 40.1, 36.0, 35.5, 23.1; IR (liquid film, cm^{-1}) 3061, 3032, 2961, 2925, 2866, 1675, 1598, 1584, 1478, 1453, 1421, 1366, 1307, 1245, 1179, 1156, 1053, 1033, 1015, 905, 813, 752, 698, 614; HRMS (EI): m/z calcd. For $C_{13}H_{19}NOS$ $[M]^+$: 237.1182, found: 237.1184.

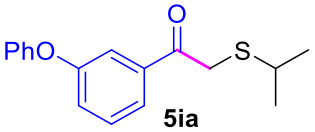

5ia

2-(Isopropylthio)-1-(3-phenoxyphenyl)ethan-1-one (5ia). Pale pink oil, 97.8 mg, 68% yield; ^{1}H NMR (400 MHz, $CDCl_3$) δ 7.69 (d, J = 7.6 Hz, 1H), 7.61 (s, 1H), 7.45-7.40 (m, 1H), 7.39-7.33 (m, 2H), 7.24-7.18 (m, 1H), 7.18-7.11 (m, 1H), 7.09-6.97 (m, 2H), 3.78 (s, 2H), 3.09-2.85 (m, 1H), 1.27 (d, J = 6.8 Hz, 6H); ^{13}C NMR (101 MHz, $CDCl_3$) δ 194.5, 157.9, 156.6, 137.2, 130.11, 130.08, 124.0, 123.6, 123.5, 119.3, 118.6, 36.5, 35.6, 23.0; IR (liquid film, cm^{-1}) 3061, 3032, 2961, 2925, 2866, 1675, 1598, 1584, 1478, 1453, 1421, 1366, 1307, 1245, 1179, 1156, 1053, 1033, 1015, 905, 813, 752, 698, 614; HRMS (EI): m/z calcd. For $C_{17}H_{18}O_2S$ $[M]^+$: 286.1022, found: 286.1027.

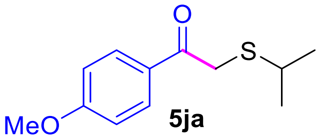

5ja

2-(Isopropylthio)-1-(4-methoxyphenyl)ethan-1-one (5ja). Colorless oil, 93.2 mg, 82% yield; ^{1}H NMR (400 MHz, $CDCl_3$) δ 7.95 (d, J = 8.8 Hz, 2H), 6.93 (d, J = 8.8 Hz, 2H), 3.86 (s, 3H), 3.77 (s, 2H), 3.20-2.84 (m, 1H), 1.27 (d, J = 6.8 Hz, 6H); ^{13}C NMR (101 MHz, $CDCl_3$) δ 193.9, 163.7, 131.2, 128.4, 113.9, 55.6, 36.2, 35.6, 23.1; IR (liquid film, cm^{-1}) 3061, 3032, 2961, 2925, 2866, 1675, 1598, 1584, 1478, 1453, 1421, 1383, 1366, 1307, 1245, 1179, 1156, 1137, 1053, 1033, 1015, 752, 698, 614; HRMS (EI): m/z calcd. For $C_{12}H_{16}O_2S$ $[M]^+$: 224.0866, found: 224.0865.

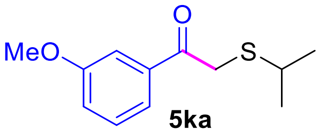

5ka

2-(Isopropylthio)-1-(3-methoxyphenyl)ethan-1-one (5ka). Colorless oil, 89.5 mg, 79% yield; ^{1}H NMR (400 MHz, $CDCl_3$) δ 7.58-7.51 (m, 1H), 7.50 (s, 1H), 7.40-7.32 (m, 1H), 7.15-7.06 (m, 1H), 3.84 (s, 3H), 3.81 (s, 2H), 3.06-2.90 (m, 1H), 1.27 (d, *J* = 6.8 Hz, 6H); ^{13}C NMR (101 MHz, $CDCl_3$) δ 194.9, 159.9, 136.8, 129.7, 121.5, 119.9, 113.1, 55.5, 36.5, 35.6, 23.0; IR (liquid film, cm^{-1}) 3061, 3032, 2961, 2925, 2866, 1675, 1598, 1584, 1478, 1453, 1421, 1383, 1366, 1307, 1245, 1179, 1156, 1137, 1053, 1033, 1015, 999.9, 905, 752, 698, 614; HRMS (EI): *m*/*z* calcd. For $C_{12}H_{16}O_2S$ [M]$^+$: 224.0866, found: 224.0865.

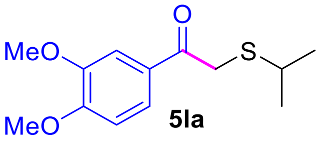

5la

1-(3,4-Dimethoxyphenyl)-2-(isopropylthio)ethan-1-one (5la). Colorless oil, 97.7 mg, 78% yield; ^{1}H NMR (400 MHz, $CDCl_3$) δ 7.58 (dd, *J* = 8.4, 2.0 Hz, 1H), 7.53 (d, *J* = 2.0 Hz, 1H), 6.87 (d, *J* = 8.4 Hz, 1H), 3.93 (s, 3H), 3.92 (s, 3H), 3.77 (s, 2H), 3.06-2.92 (m, 1H), 1.27 (d, *J* = 6.8 Hz, 6H); ^{13}C NMR (101 MHz, $CDCl_3$) δ 194.0, 153.3, 149.2, 128.5, 123.7, 110.8, 110.1, 56.2, 56.1, 36.1, 35.7, 23.1; IR (liquid film, cm^{-1}) 3061, 3032, 2961, 2925, 2866, 1675, 1598, 1584, 1478, 1453, 1421, 1383, 1366, 1307, 1245, 1179, 1156, 1137, 1033, 1015, 905, 813, 752, 698, 614; HRMS (EI): *m*/*z* calcd. For $C_{13}H_{18}O_3S$ [M]$^+$: 254.0971, found: 254.0976.

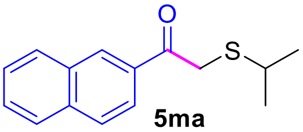

5ma

1-(3,4-Dimethoxyphenyl)-2-(isopropylthio)ethan-1-one (5la). Pale pink oil, 84.8 mg, 70% yield; ^{1}H NMR (400 MHz, $CDCl_3$) δ 8.51 (s, 1H), 8.03 (d, *J* = 8.0 Hz, 1H), 7.96 (d, *J* = 8.0 Hz, 1H), 7.92-7.84 (m, 2H), 7.65-7.49 (m, 2H), 3.95 (s, 2H), 3.22-2.89 (m, 1H), 1.30 (d, *J* = 6.8 Hz, 6H); ^{13}C NMR (101 MHz, $CDCl_3$) δ 195.1, 135.7, 132.7, 132.6,

130.7, 129.8, 128.7, 128.6, 127.9, 126.9, 124.5, 36.5, 35.7, 23.1; IR (liquid film, cm^{-1}) 3061, 3032, 2961, 2925, 2866, 1675, 1598, 1584, 1478, 1453, 1421, 1383, 1307, 1245, 1179, 1156, 1053, 1033, 1015, 999.9, 752, 698, 614; HRMS (EI): *m*/*z* calcd. For $C_{15}H_{16}OS$ $[M]^+$: 244.0916, found: 244.0915.

5. NMR Spectra

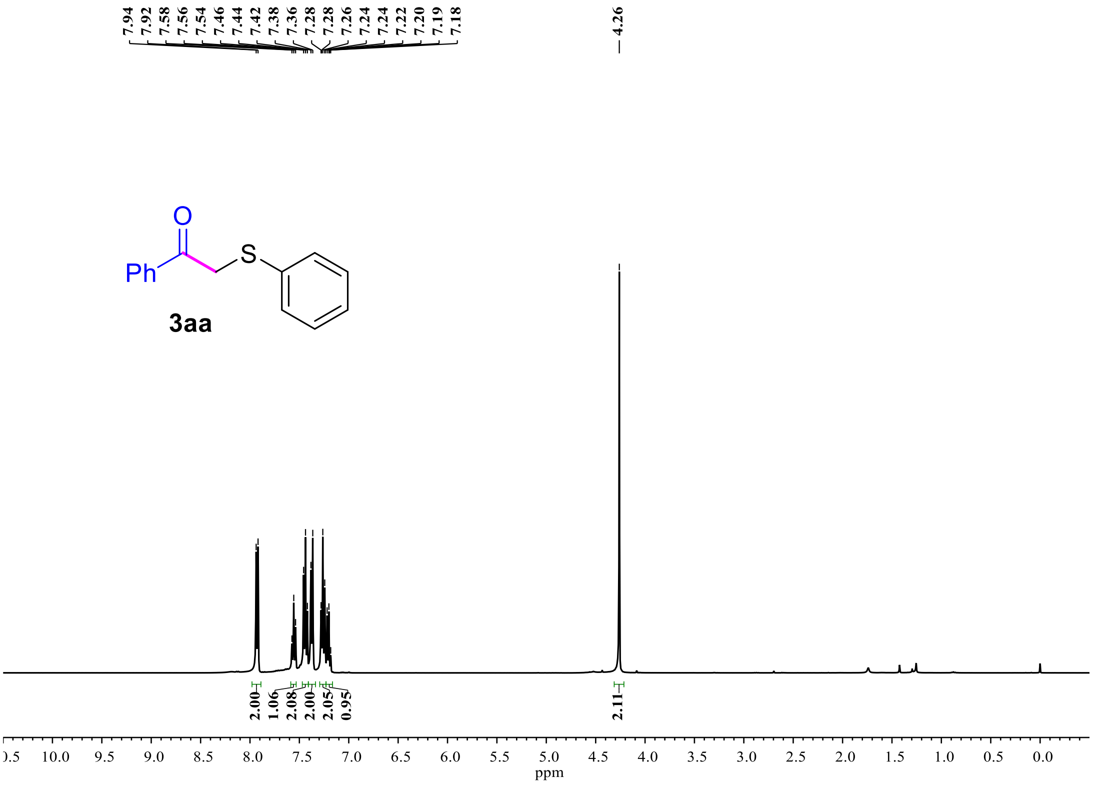

Supplementary Figure 12. ^{1}H NMR of **3aa**.

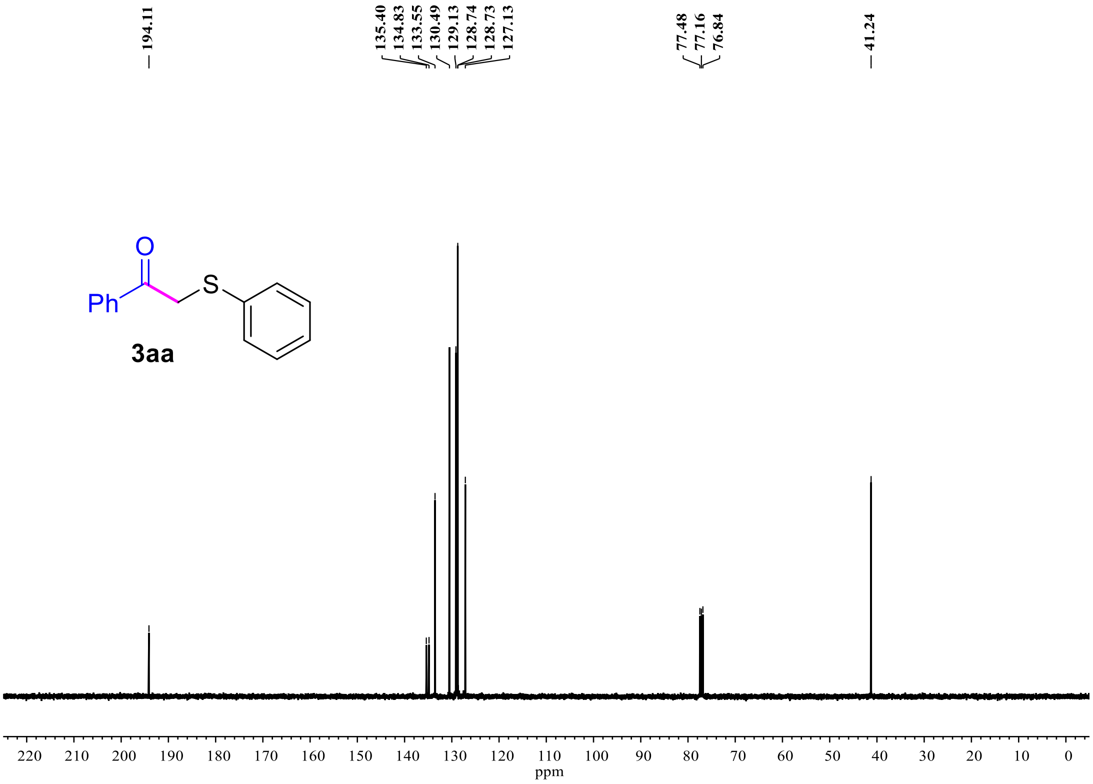

Supplementary Figure 13. ^{13}C NMR of **3aa**.

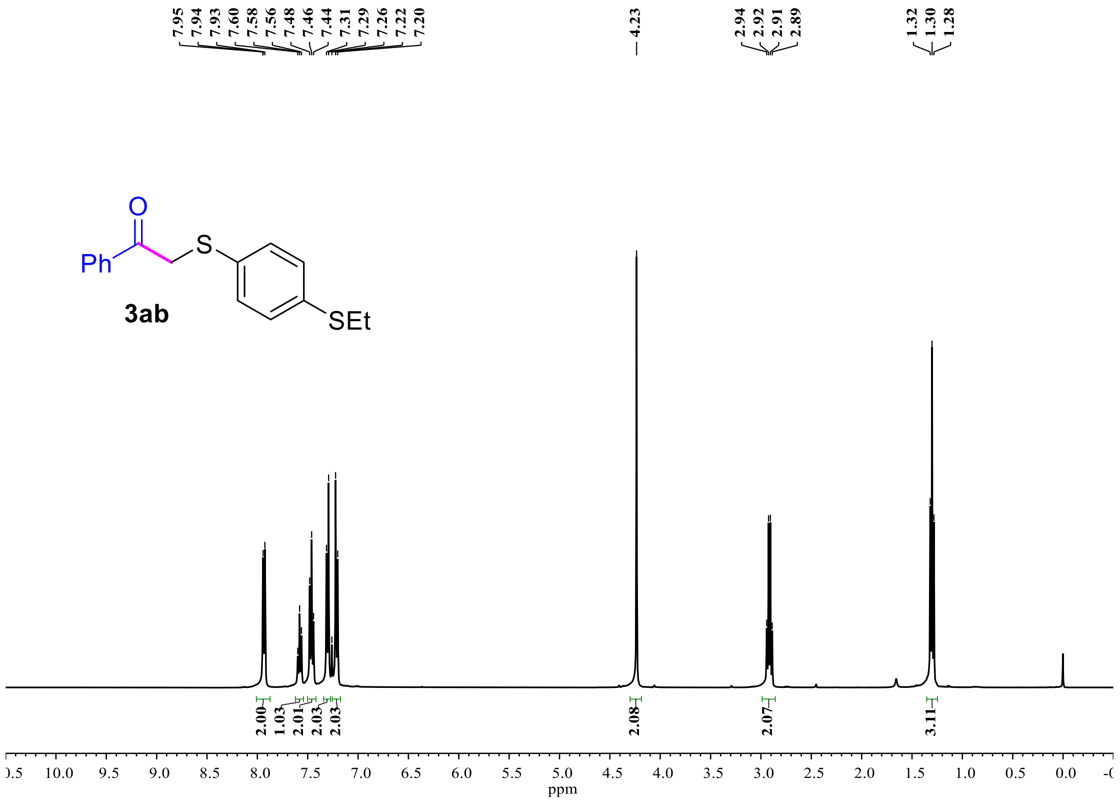

Supplementary Figure 14. ^{1}H NMR of **3ab**.

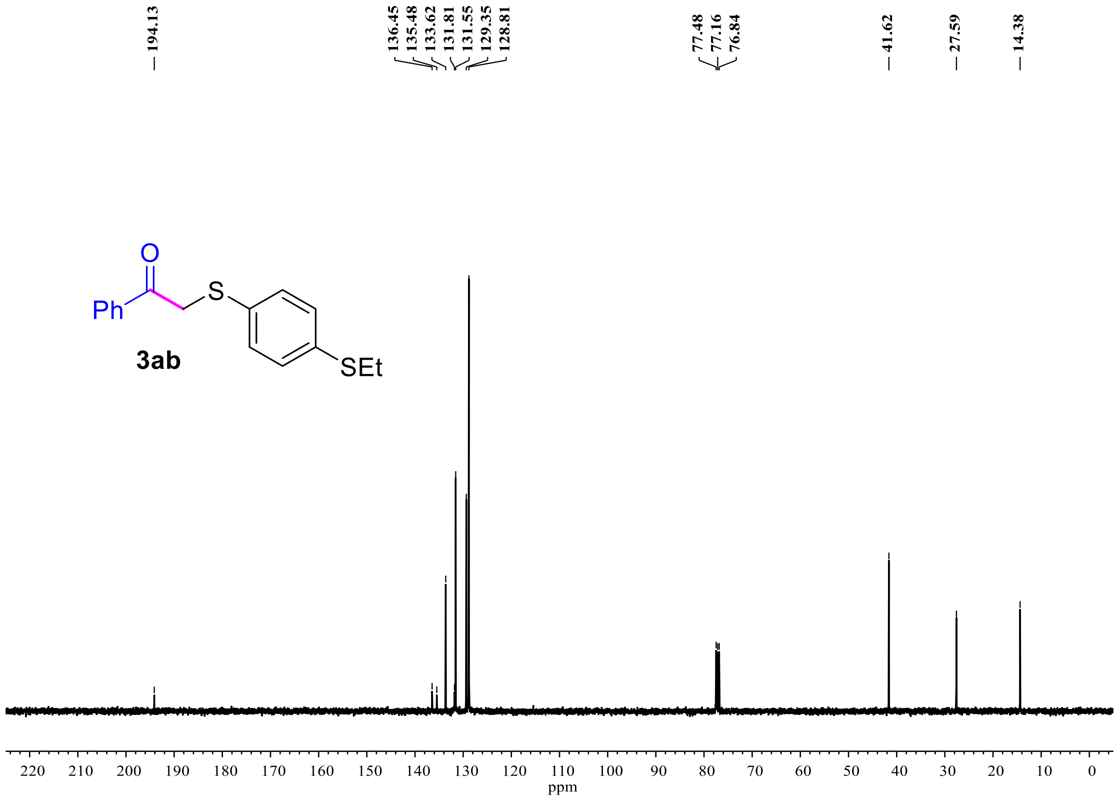

Supplementary Figure 15. ^{13}C NMR of **3ab**.

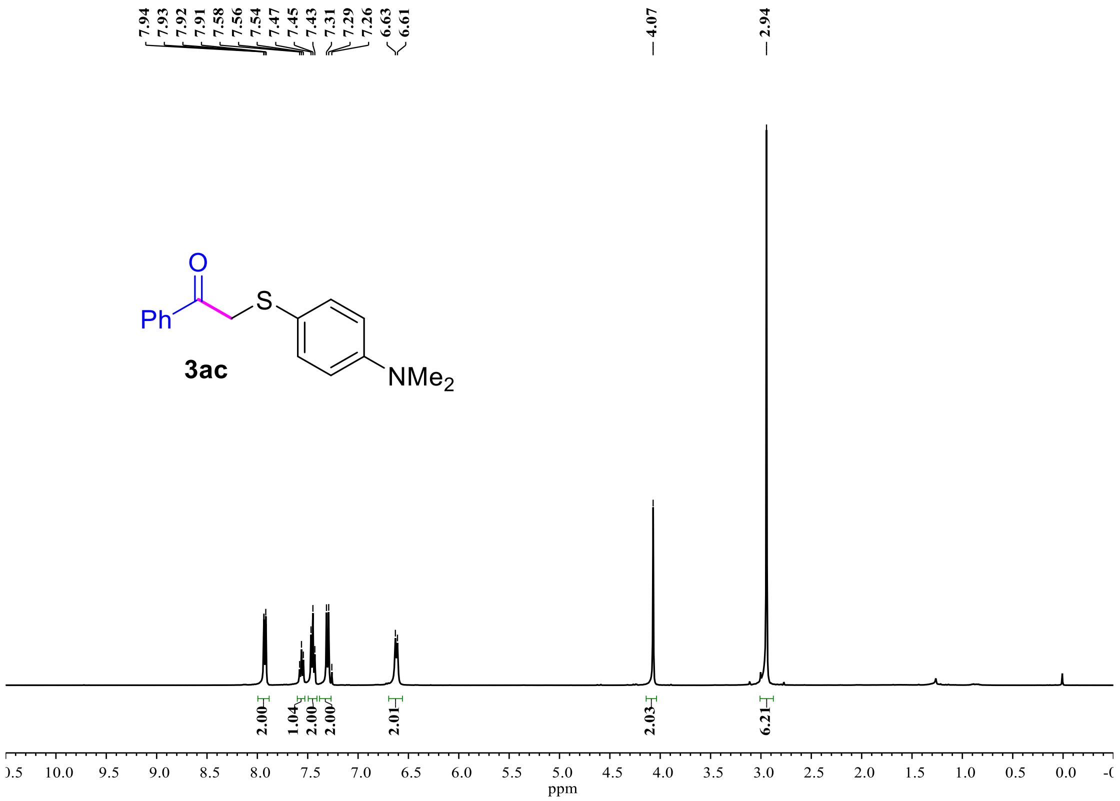

Supplementary Figure 16. ^{1}H NMR of **3ac**.

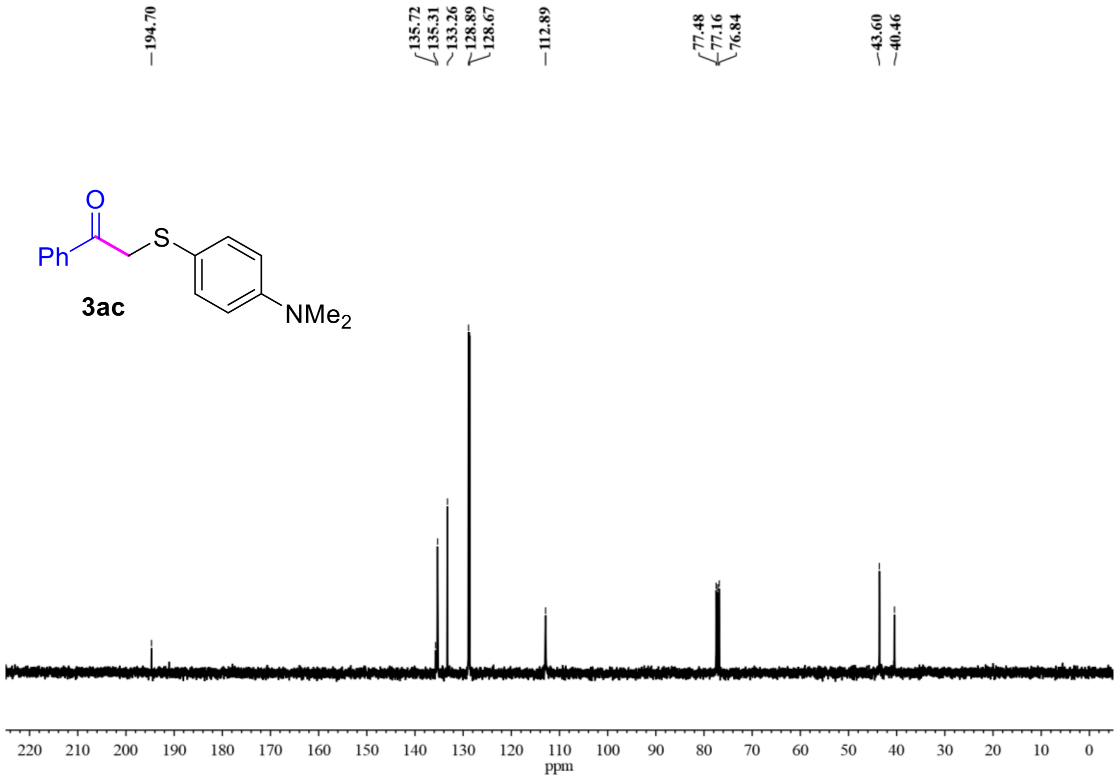

Supplementary Figure 17. ^{13}C NMR of **3ac**.

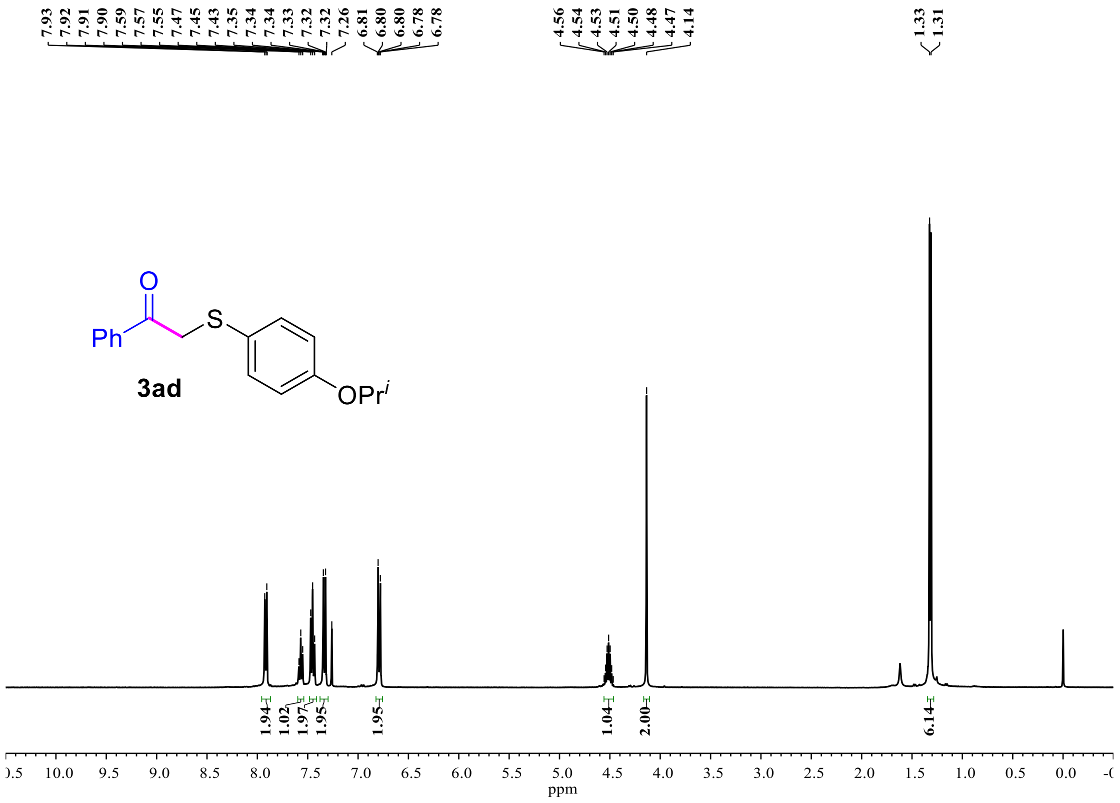

Supplementary Figure 18. ^{1}H NMR of **3ad**.

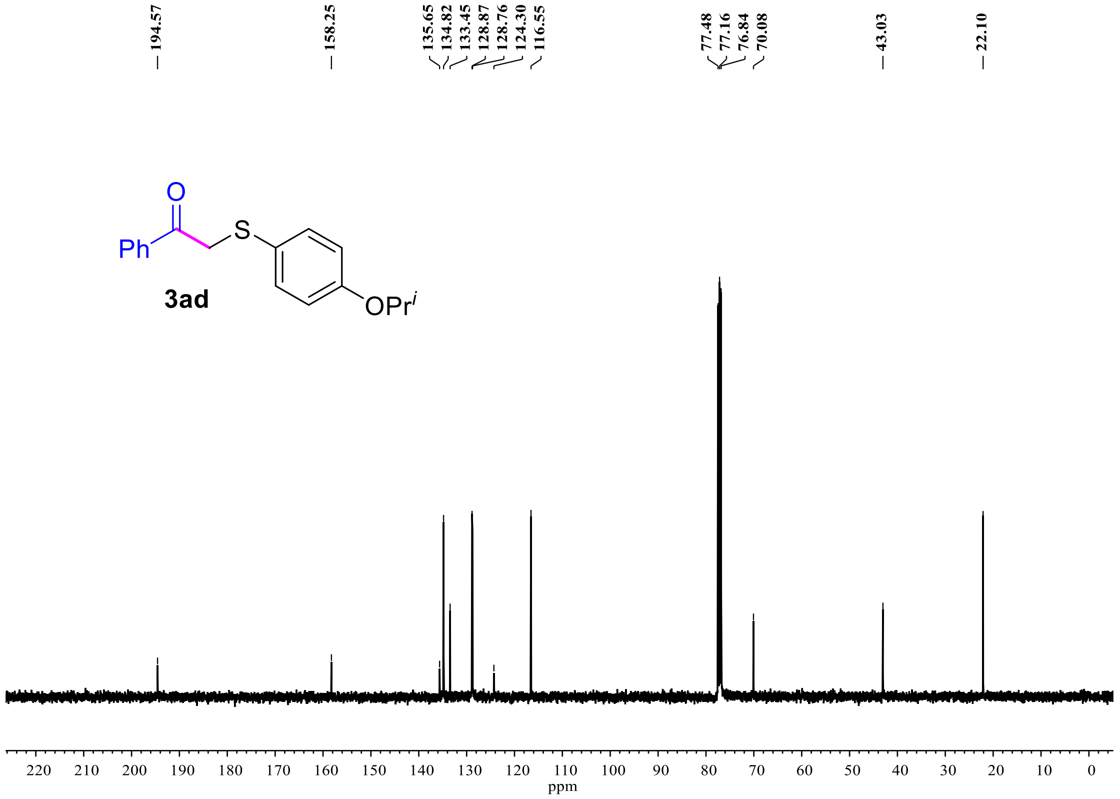

Supplementary Figure 19. ^{13}C NMR of **3ad**.

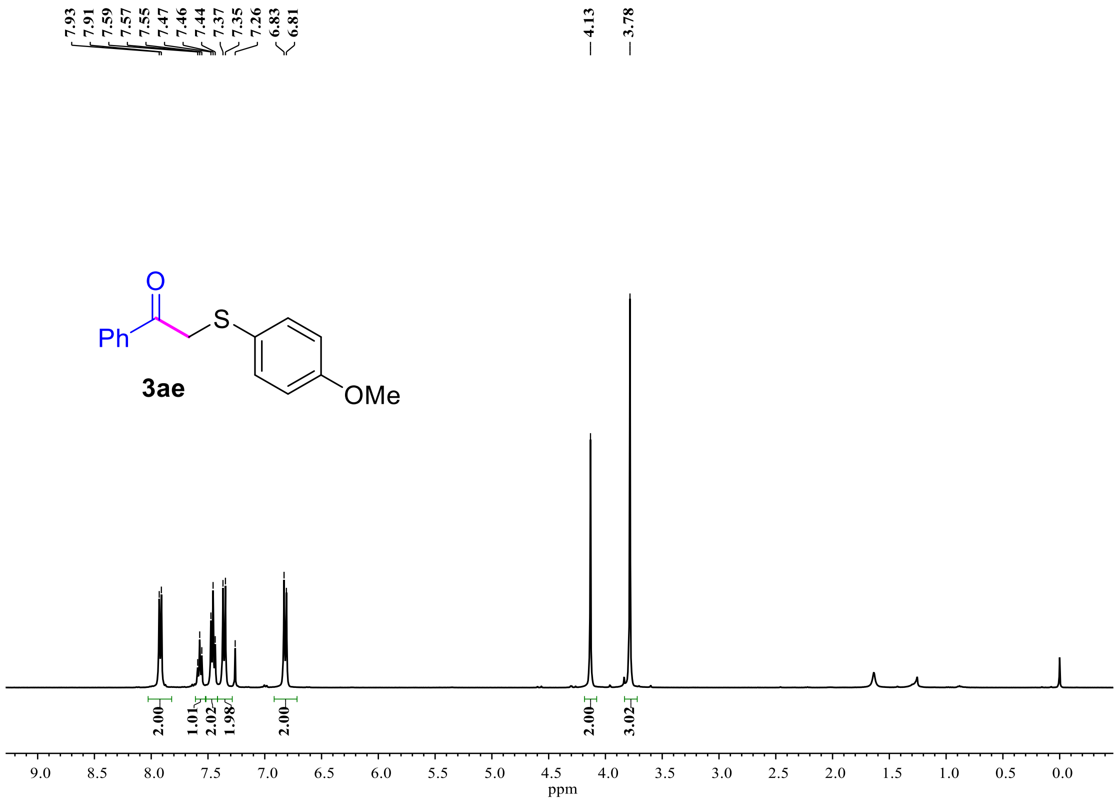

Supplementary Figure 20. ^{1}H NMR of **3ae**.

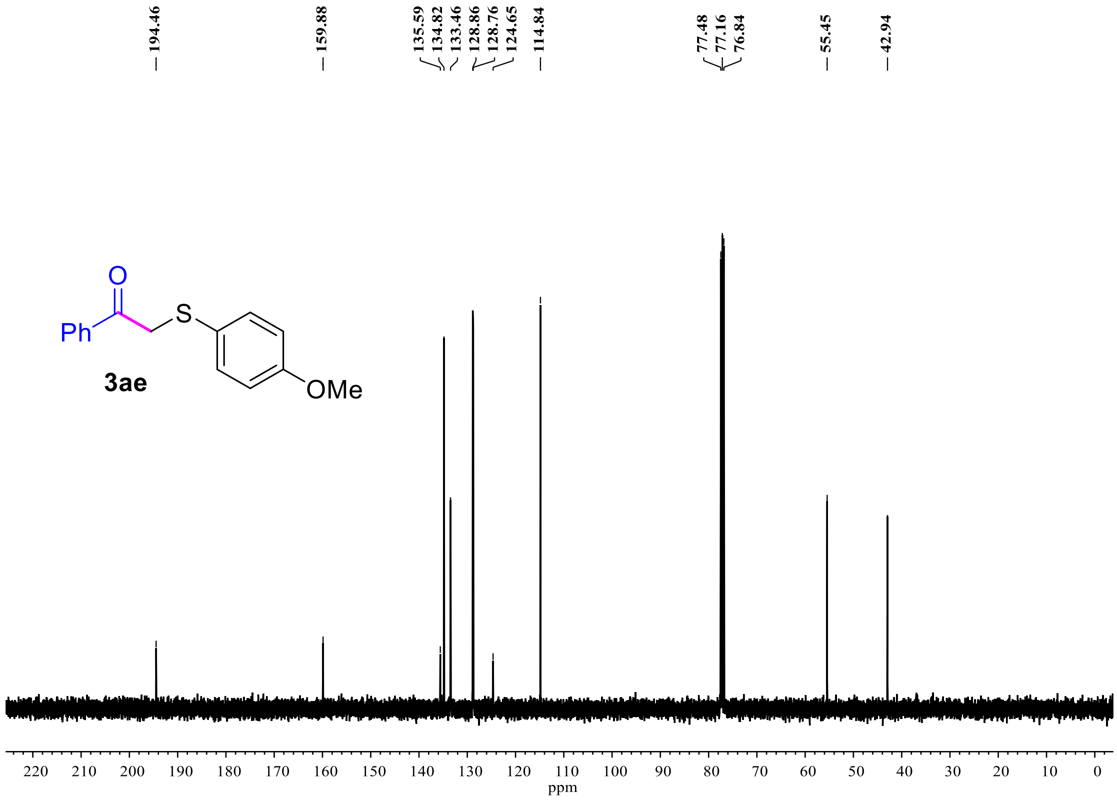

Supplementary Figure 21. ^{13}C NMR of **3ae**.

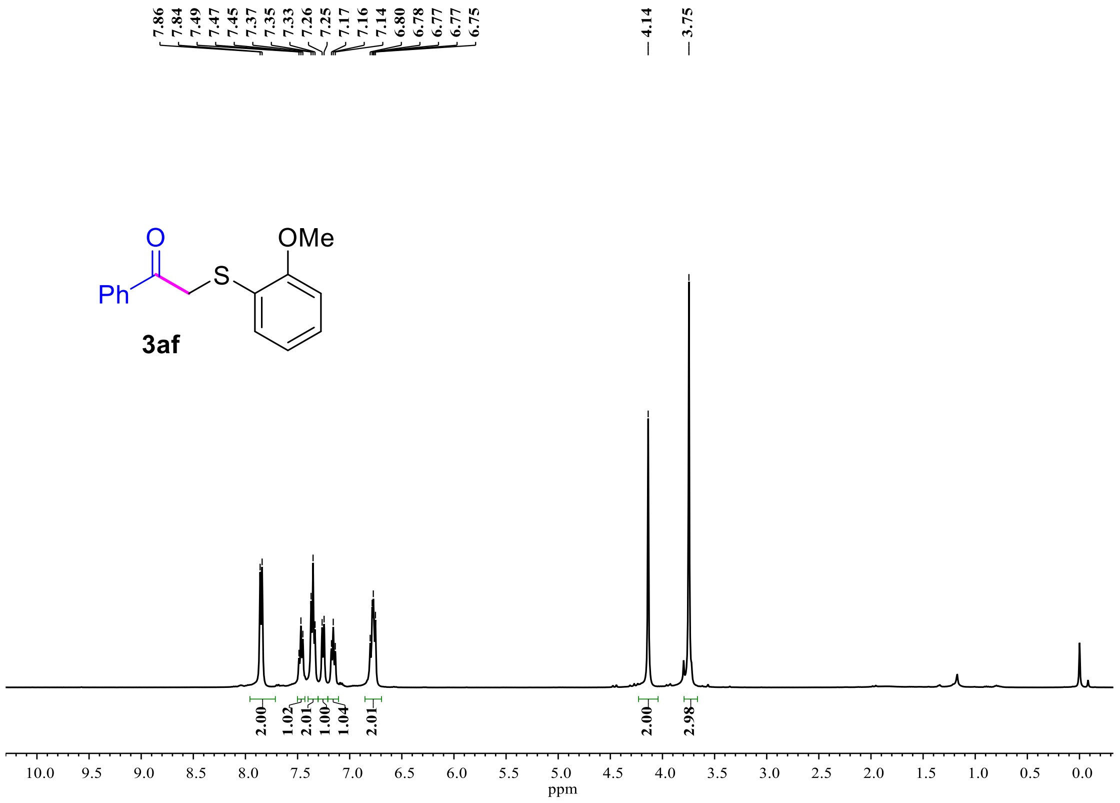

Supplementary Figure 22. ^{1}H NMR of **3af**.

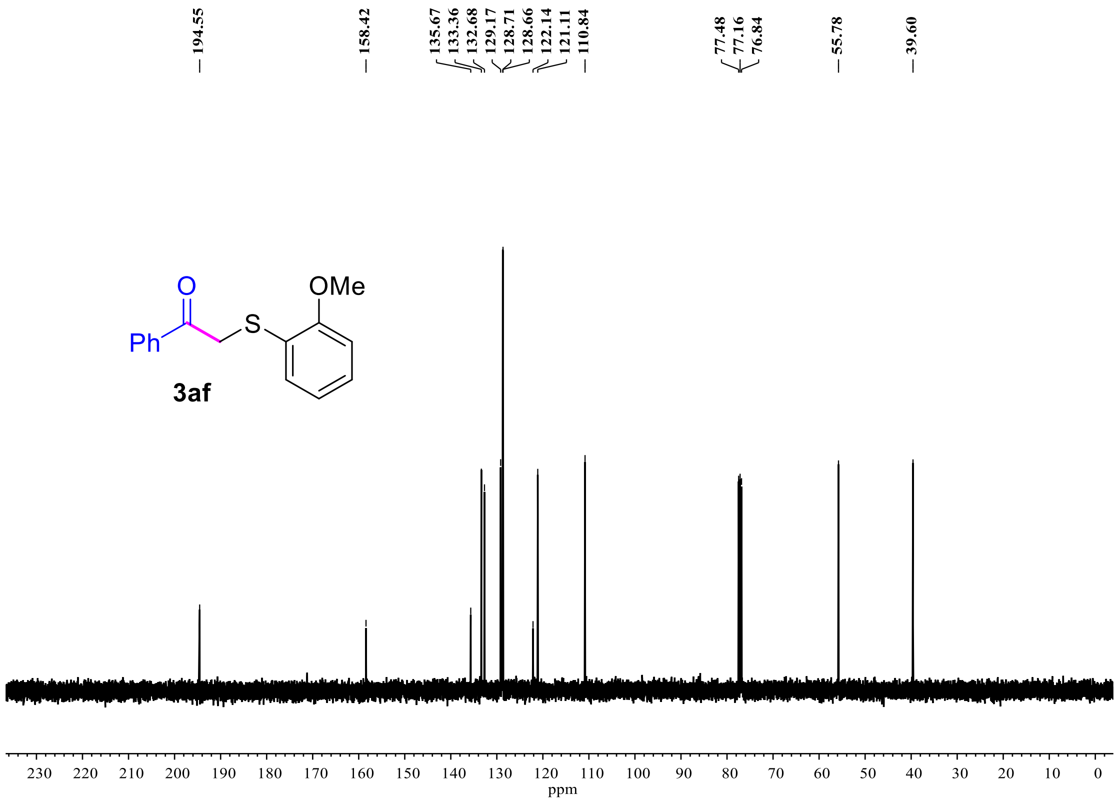

Supplementary Figure 23. ^{13}C NMR of **3af**.

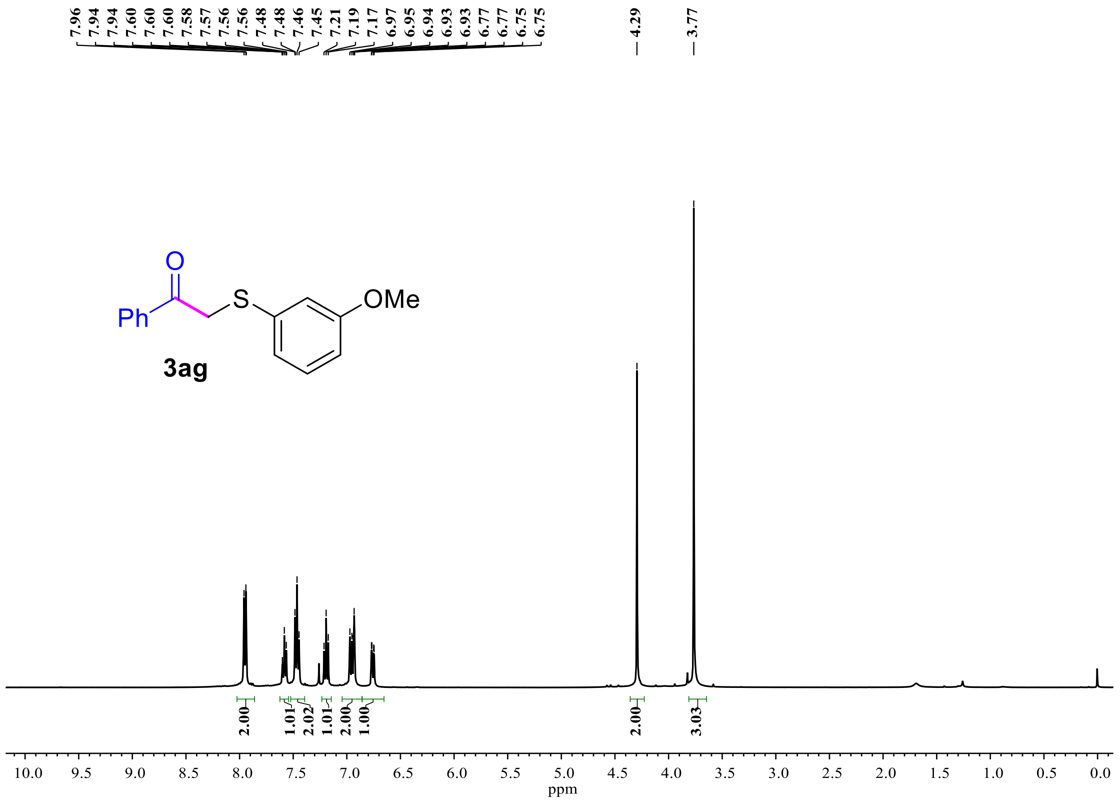

Supplementary Figure 24. ^{1}H NMR of **3ag**.

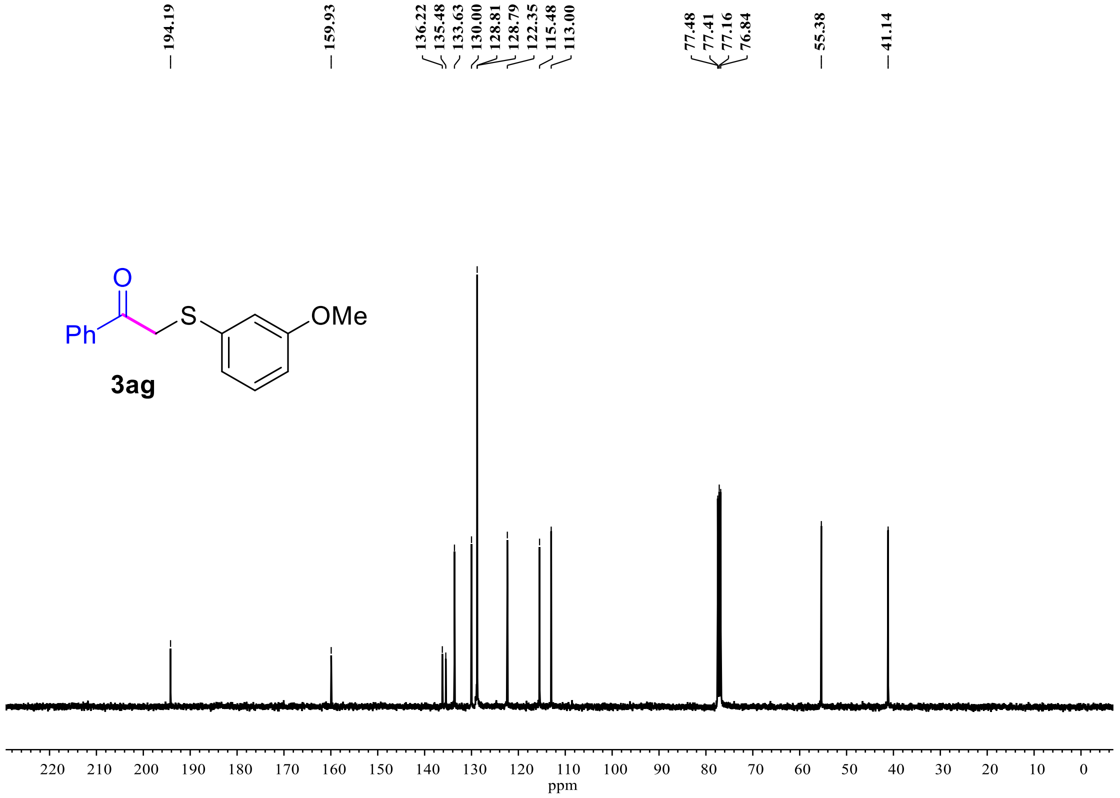

Supplementary Figure 25. ^{13}C NMR of **3ag**.

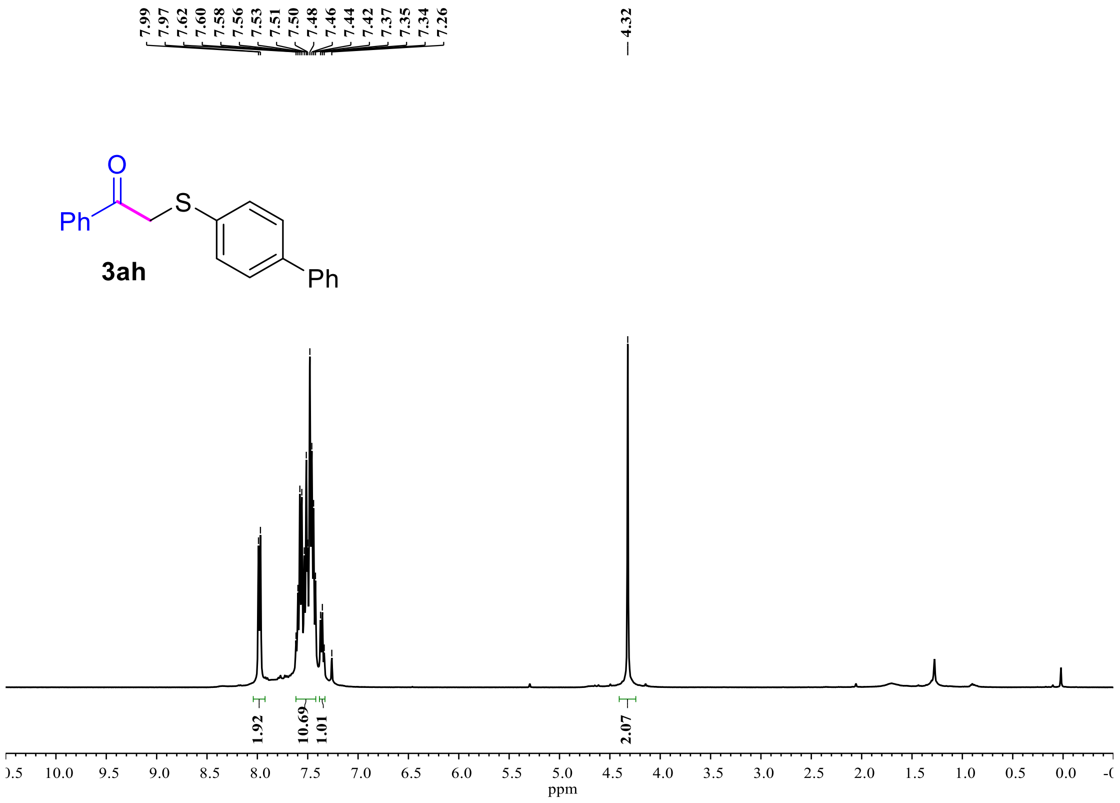

Supplementary Figure 26. ^{1}H NMR of **3ah**.

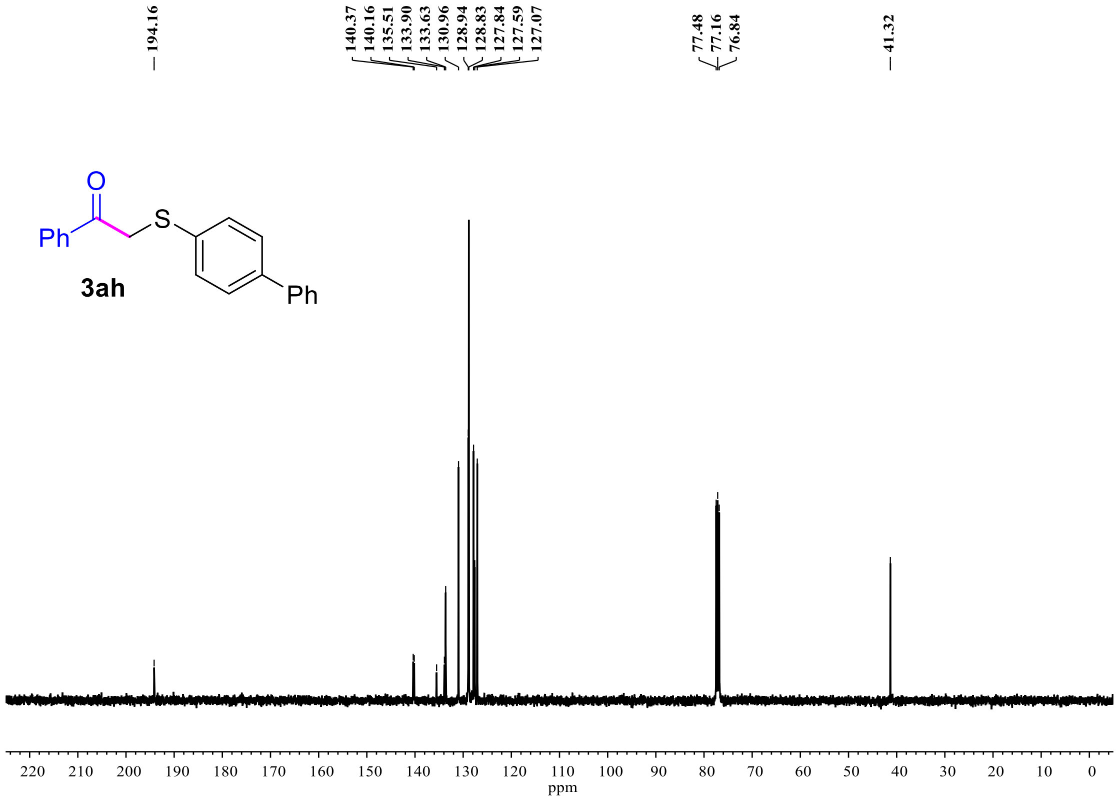

Supplementary Figure 27. ^{13}C NMR of **3ah**.

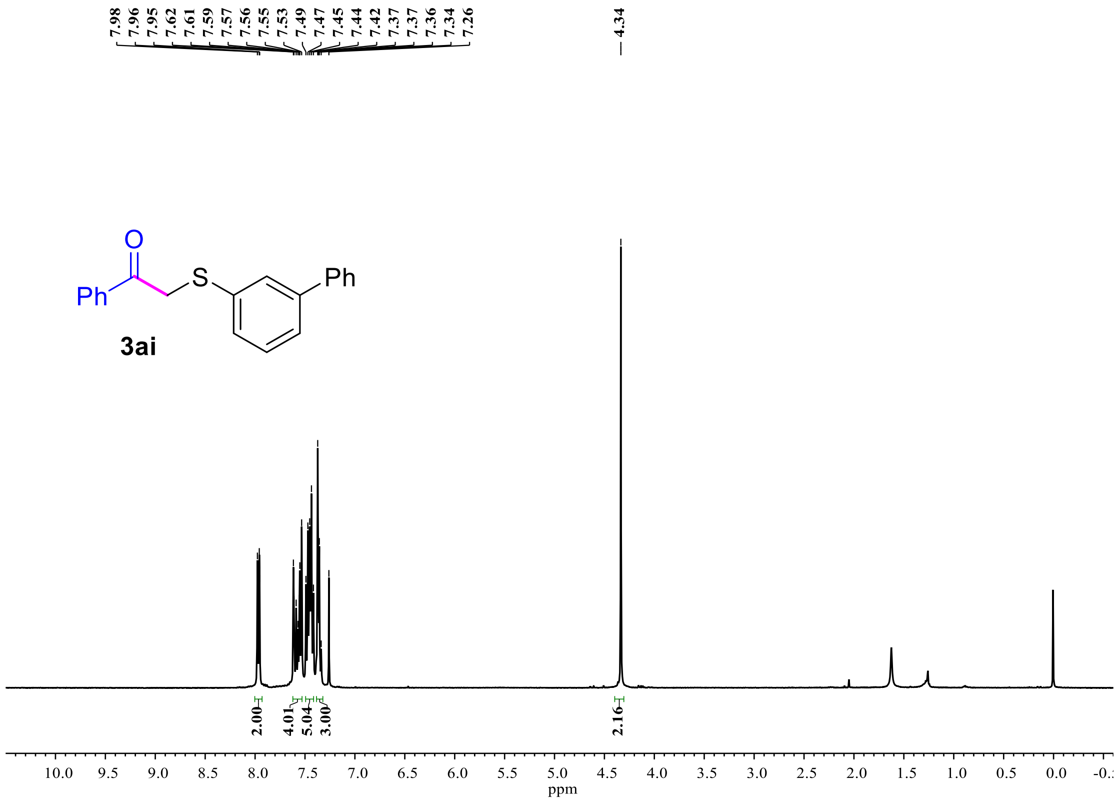

Supplementary Figure 28. ^{1}H NMR of **3ai**.

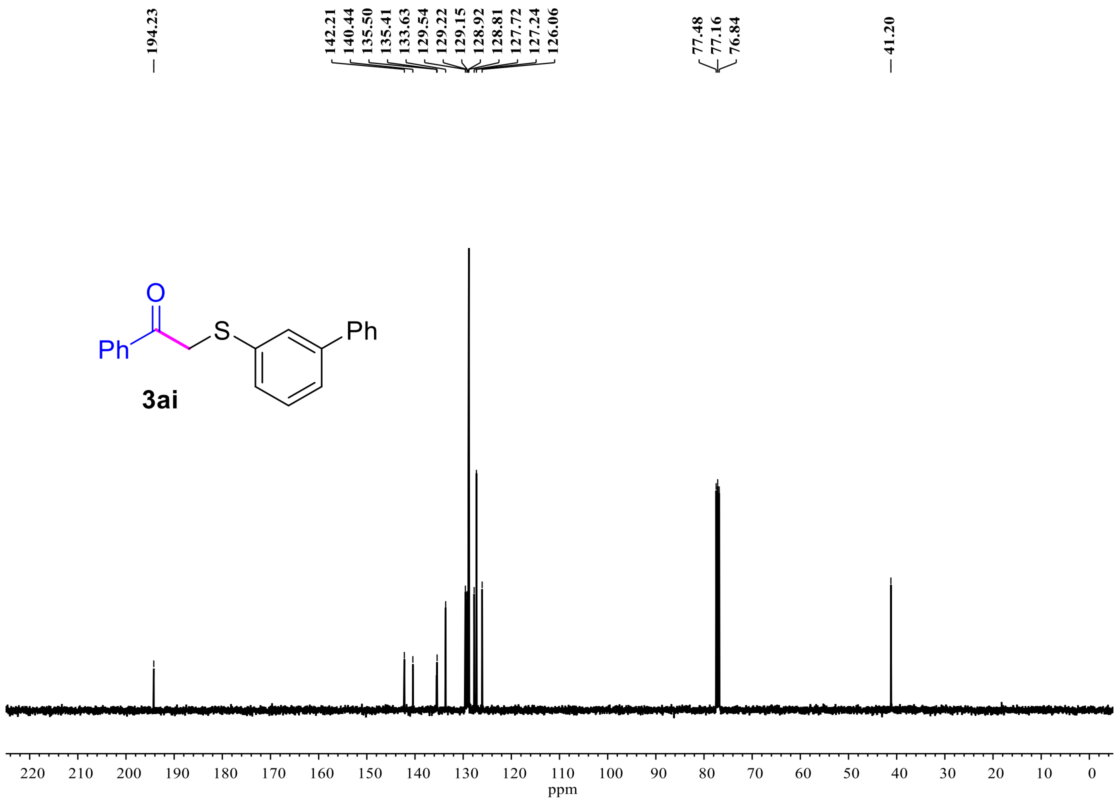

Supplementary Figure 29. ^{13}C NMR of **3ai**.

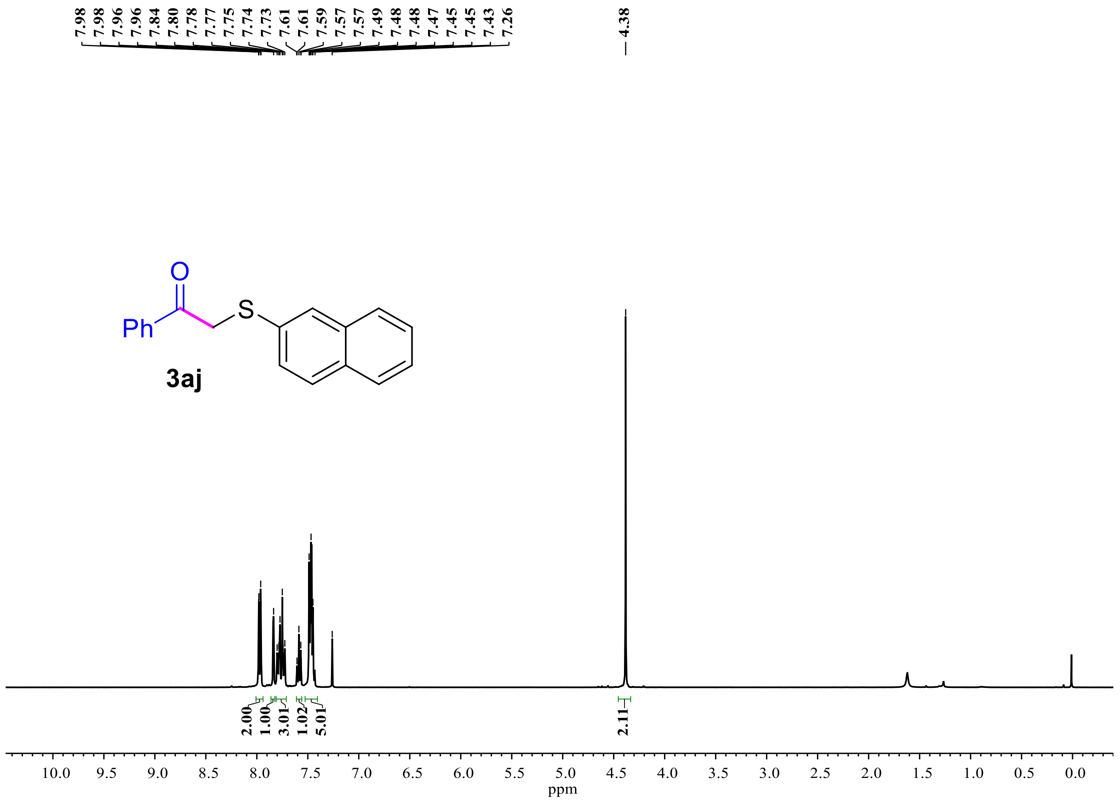

Supplementary Figure 30. ^{1}H NMR of **3aj**.

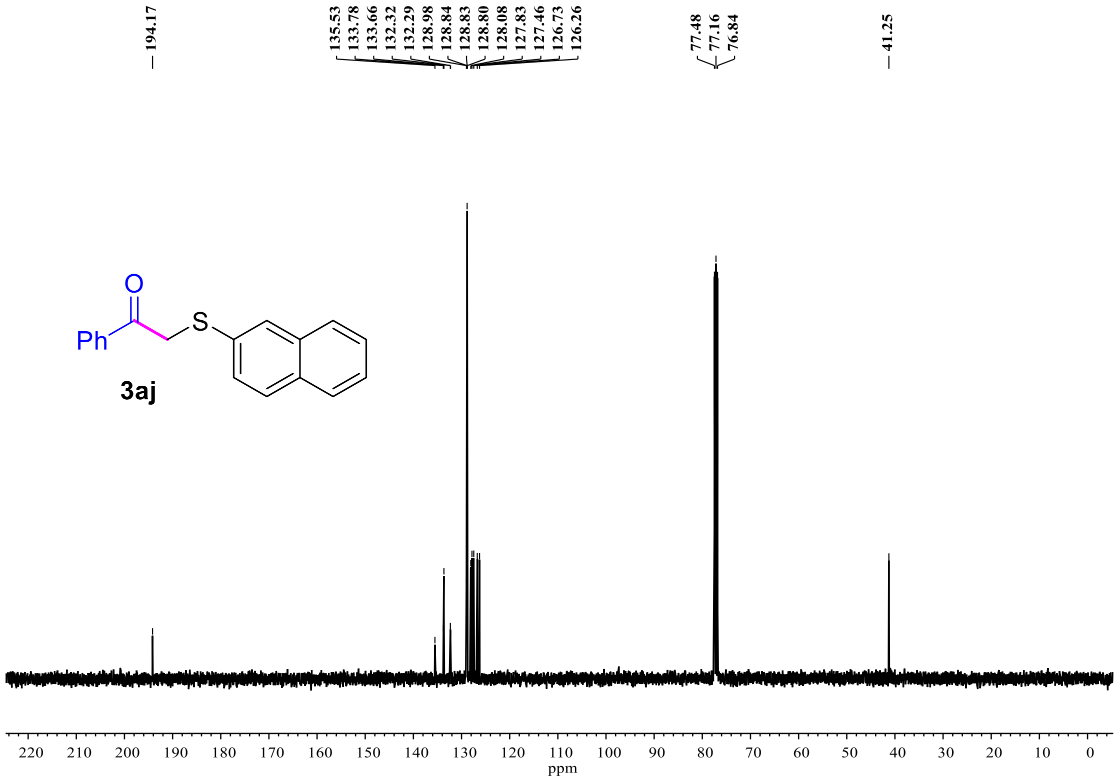

Supplementary Figure 31. ^{13}C NMR of **3aj**.

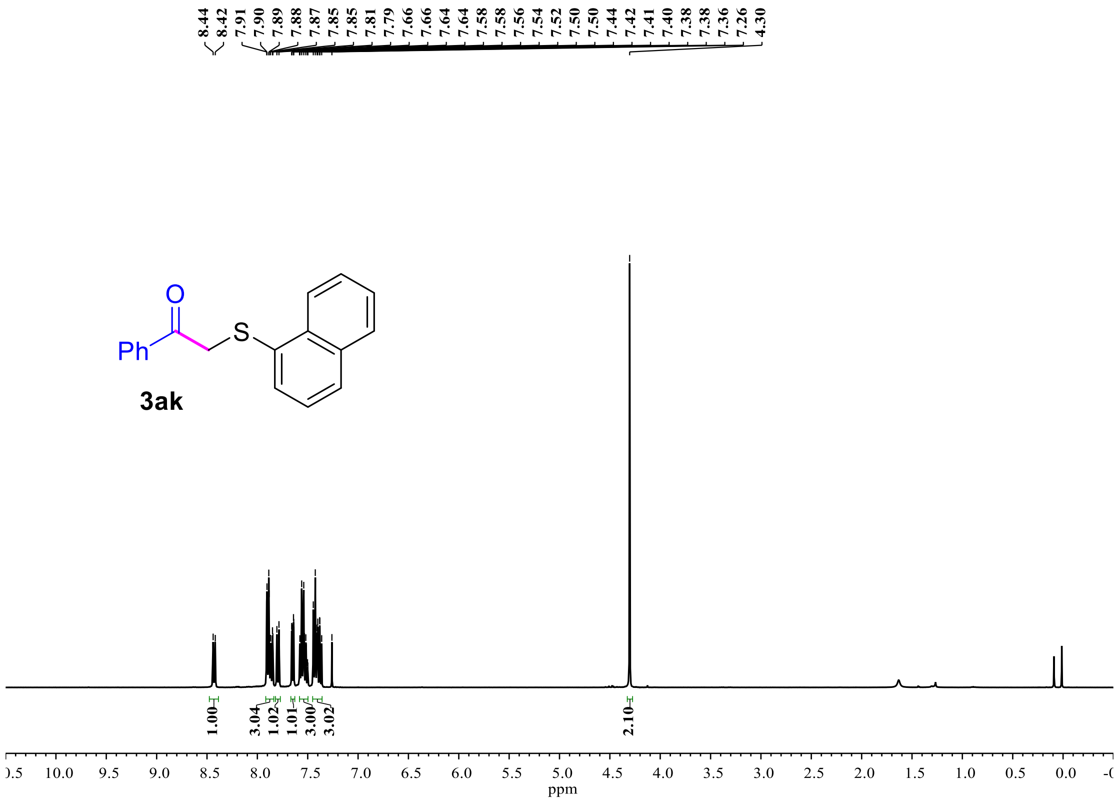

Supplementary Figure 32. ^{1}H NMR of **3ak**.

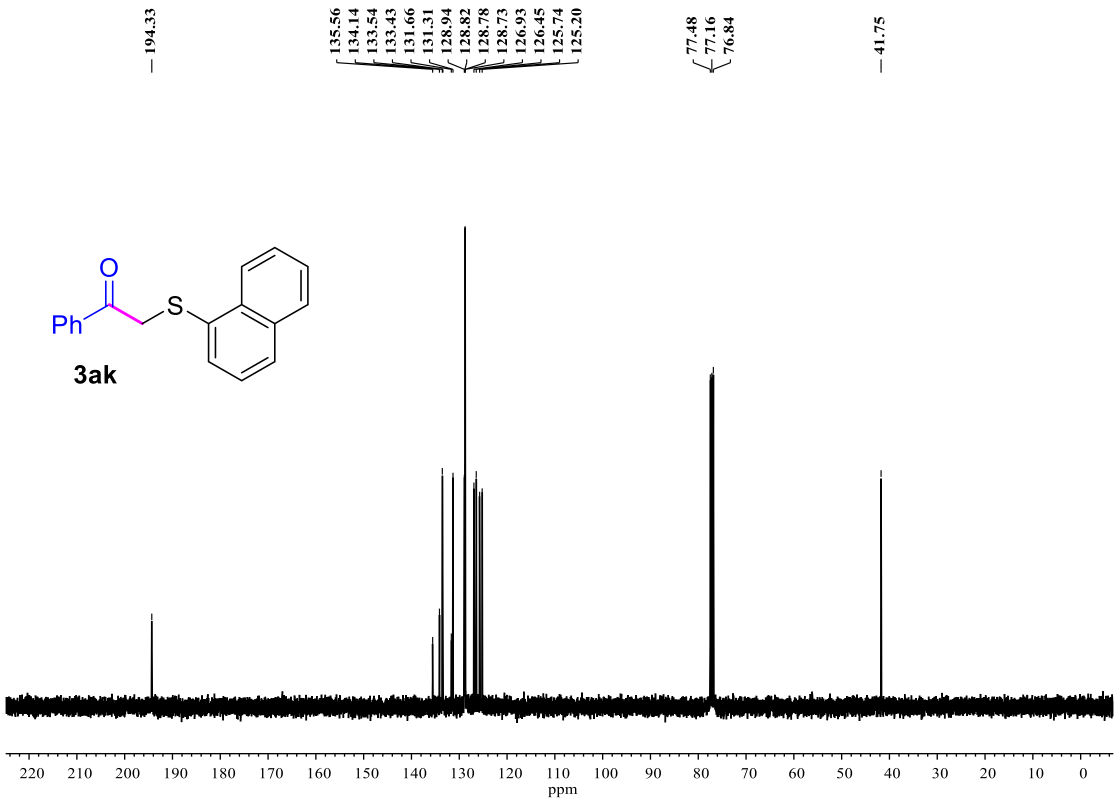

Supplementary Figure 33. ^{13}C NMR of **3ak**.

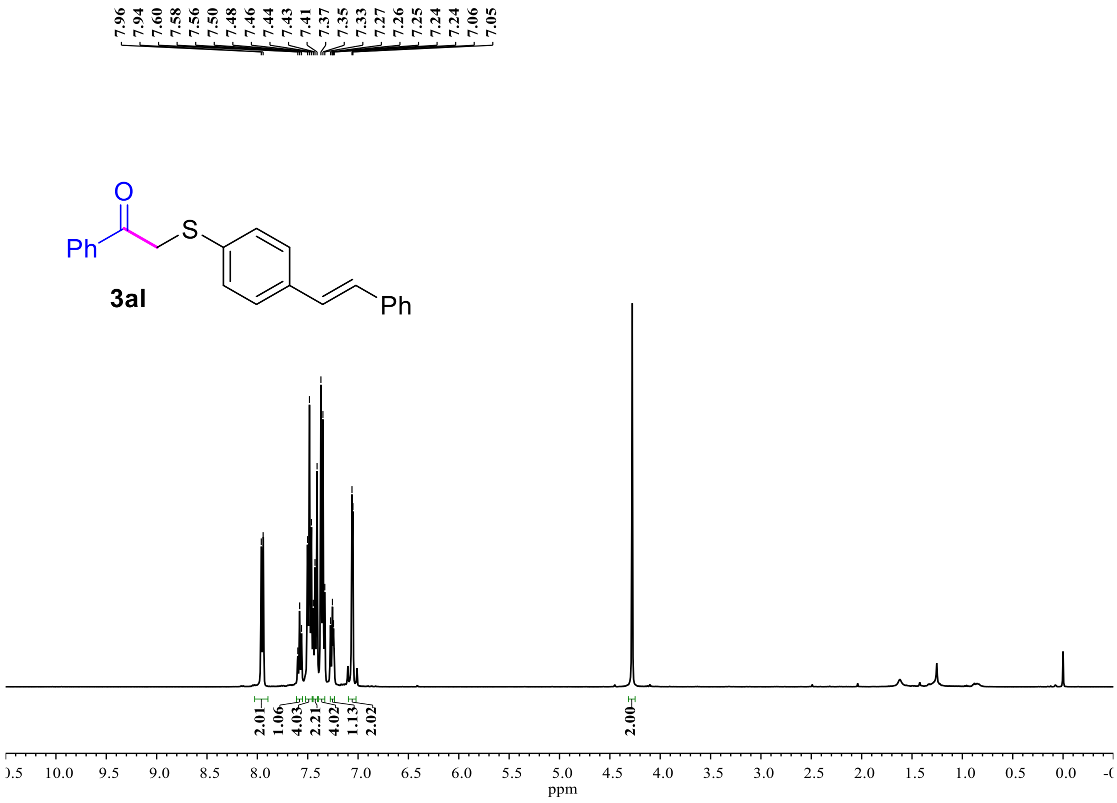

Supplementary Figure 34. ^{1}H NMR of **3al**.

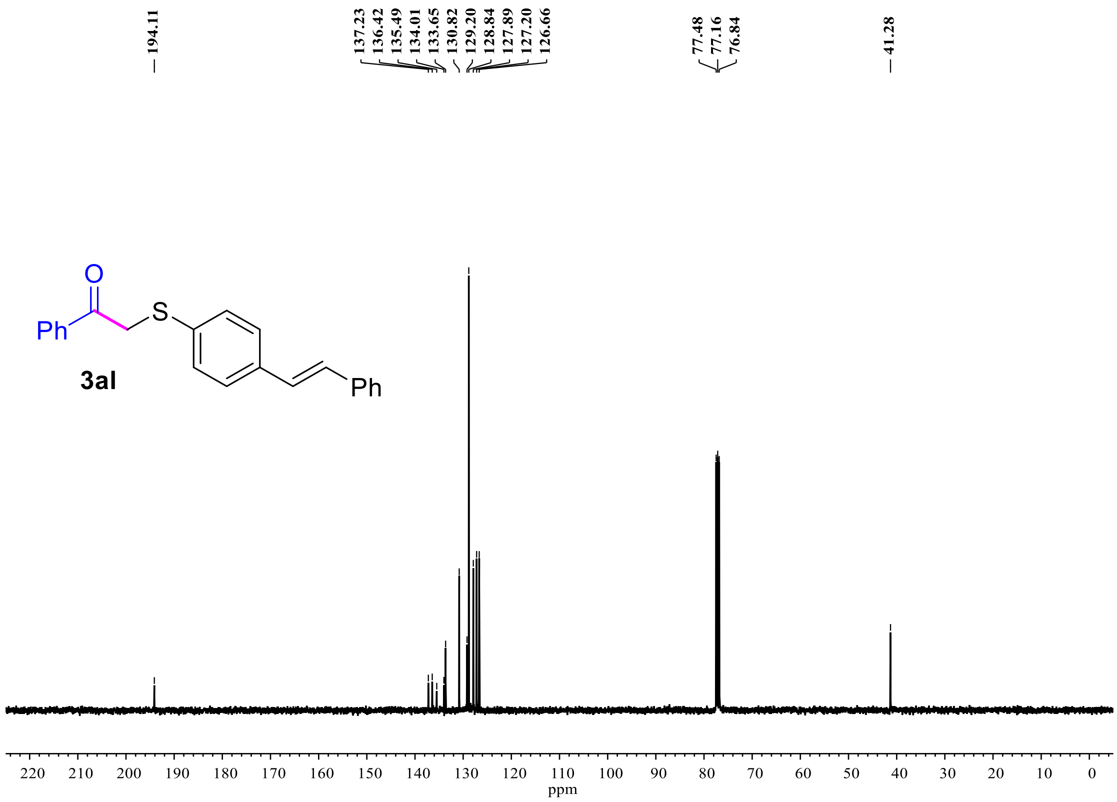

Supplementary Figure 35. ^{13}C NMR of **3al**.

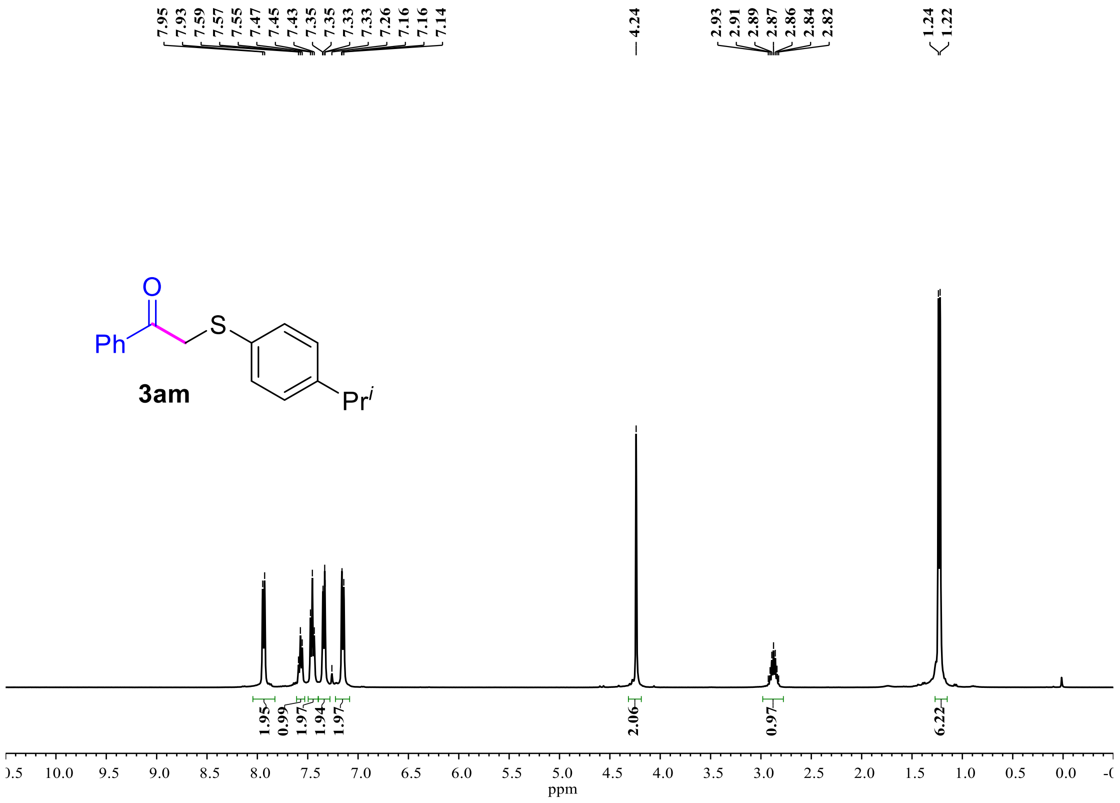

Supplementary Figure 36. ^{1}H NMR of **3am**.

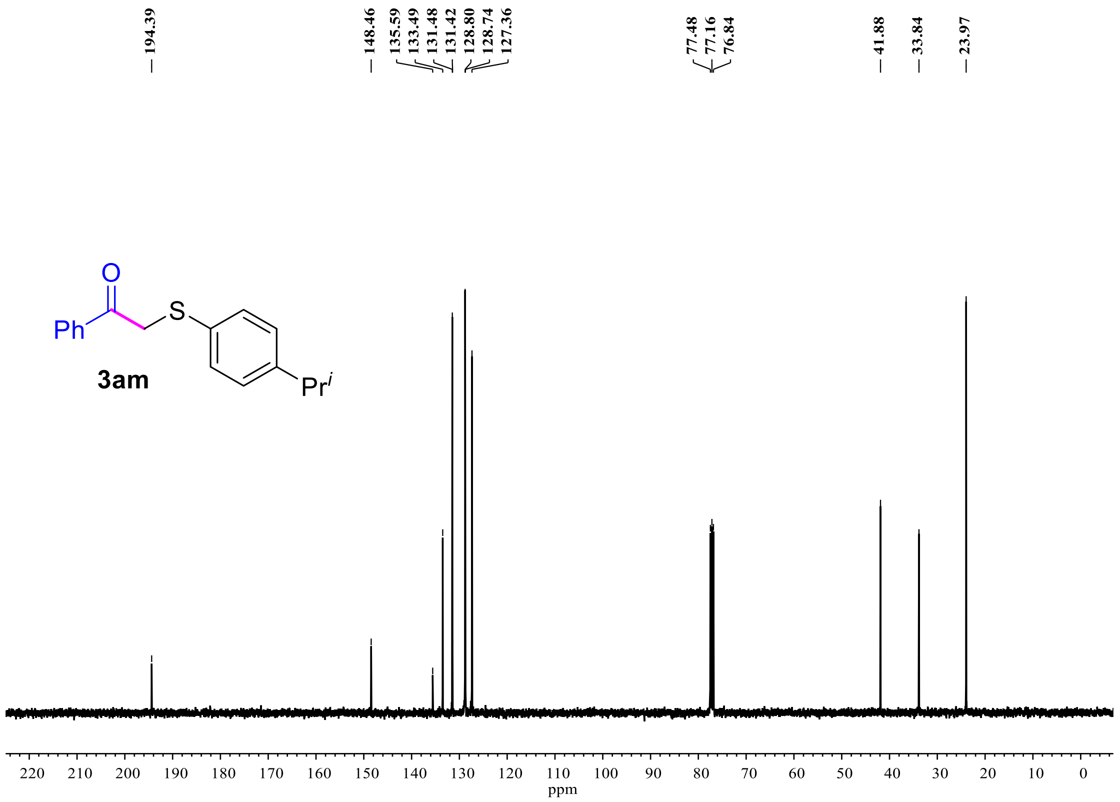

Supplementary Figure 37. ^{13}C NMR of **3am**.

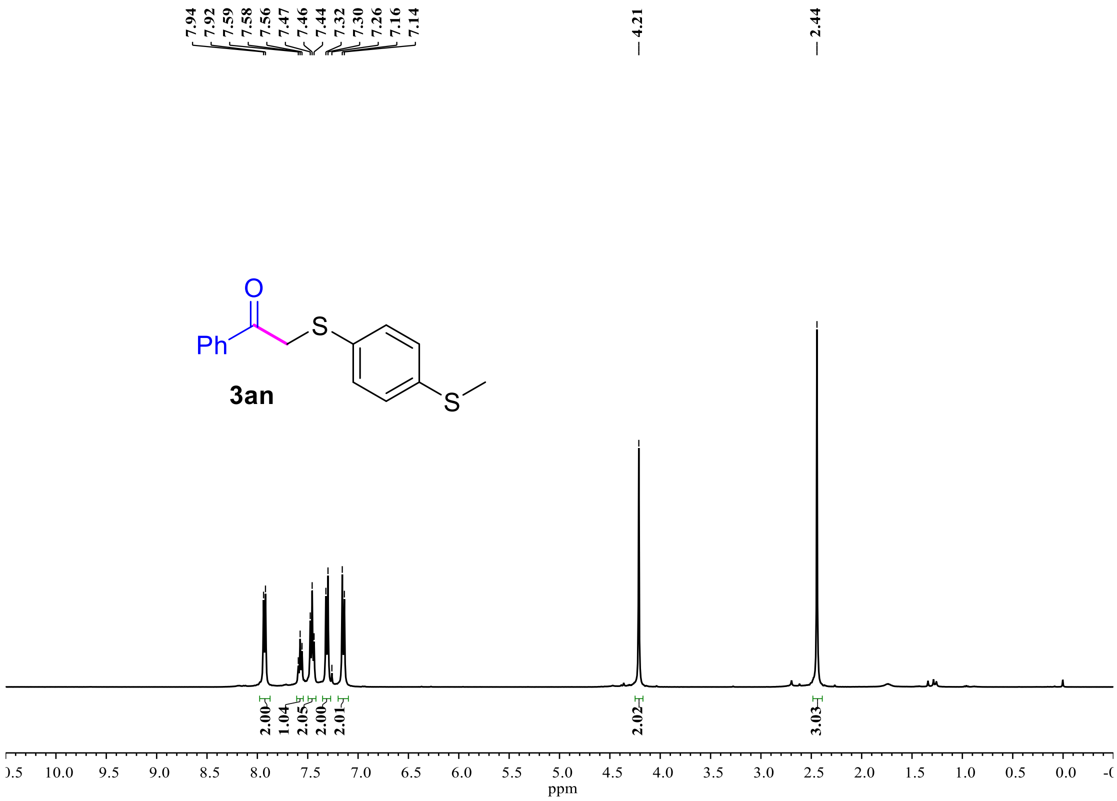

Supplementary Figure 38. ^{1}H NMR of **3an**.

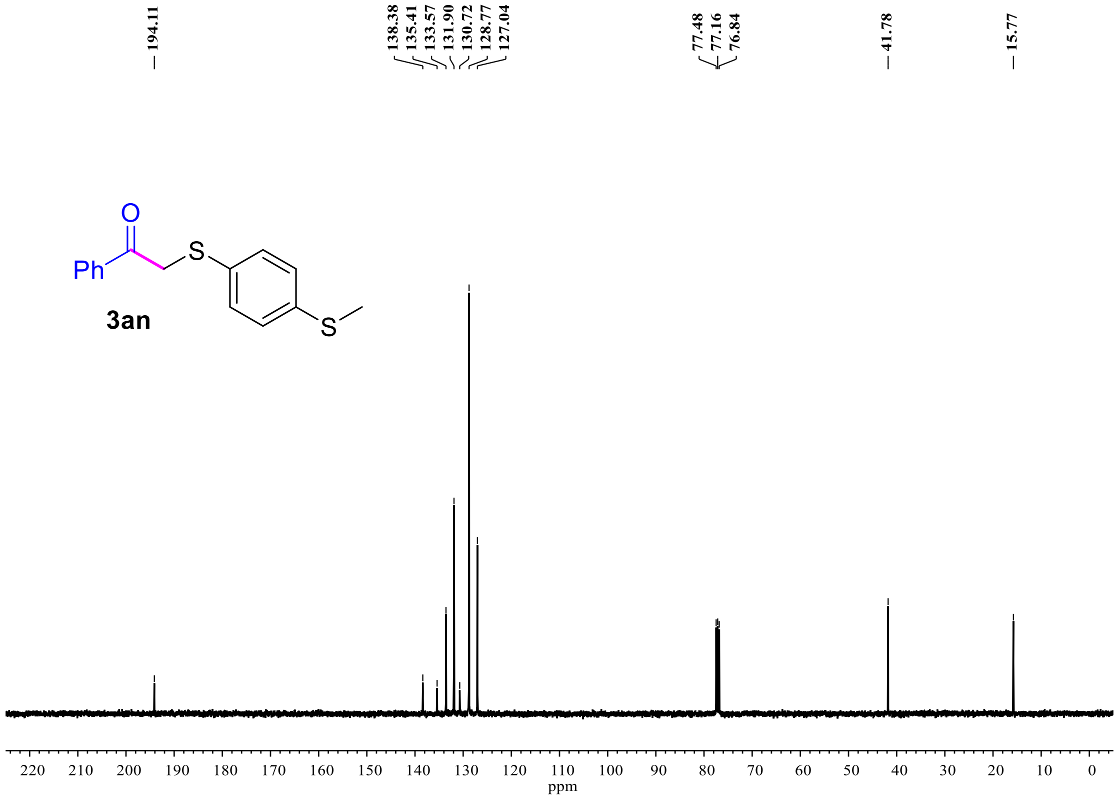

Supplementary Figure 39. ^{13}C NMR of **3an**.

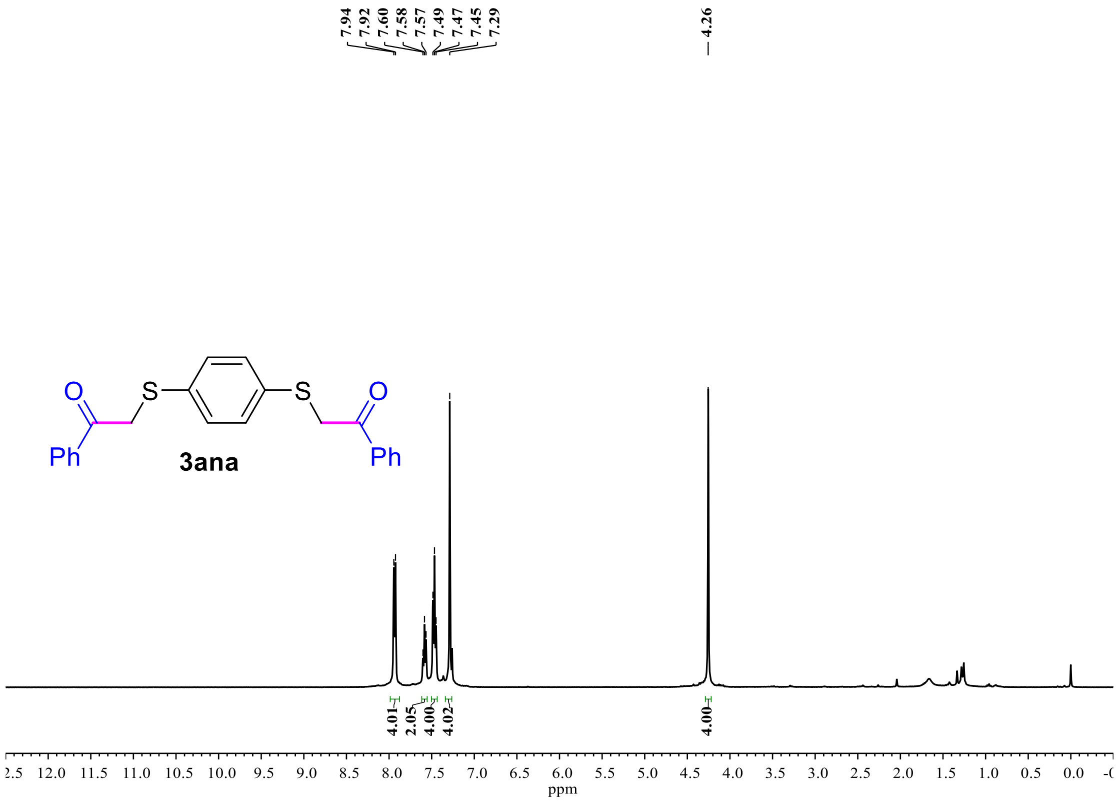

Supplementary Figure 40. ^{1}H NMR of **3ana**.

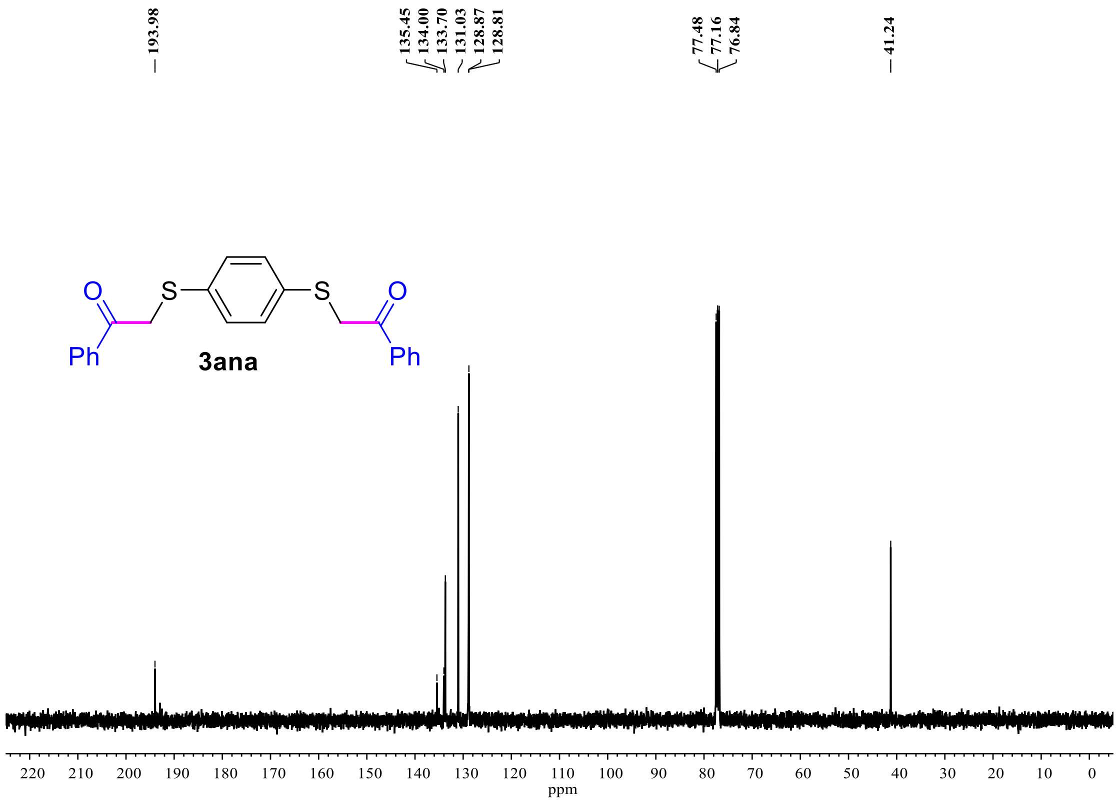

Supplementary Figure 41. ^{13}C NMR of **3ana**.

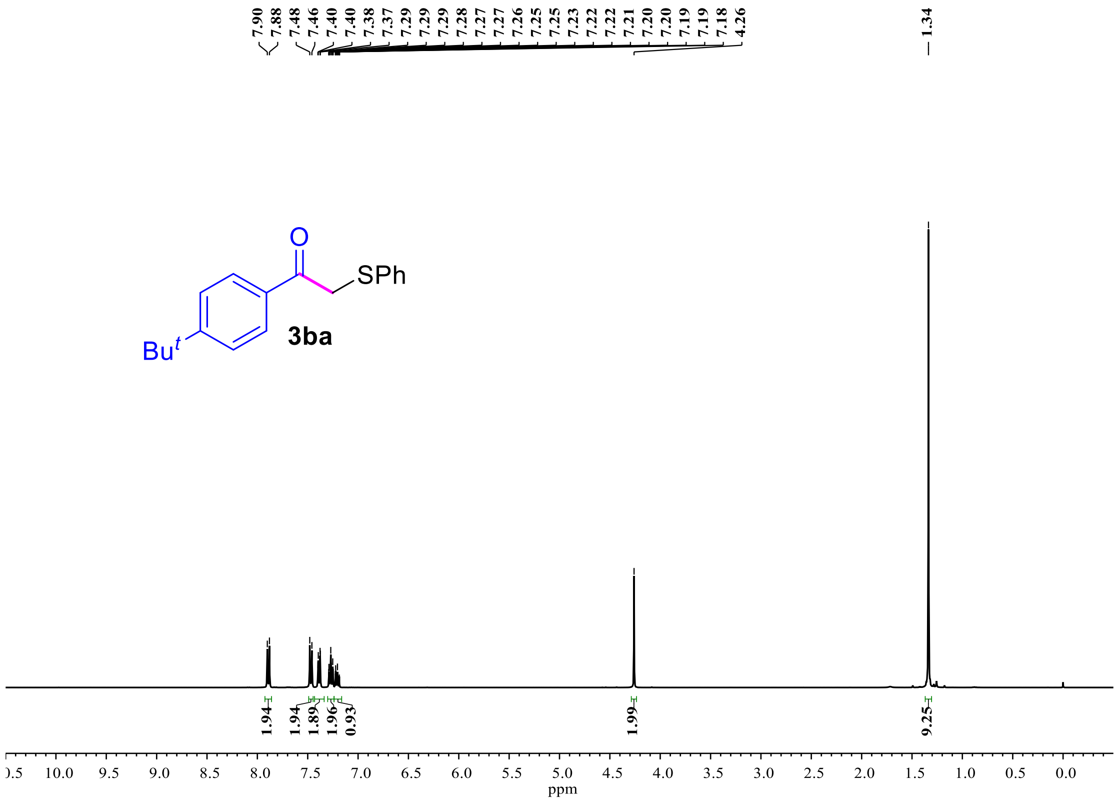

Supplementary Figure 42. ^{1}H NMR of **3ba**.

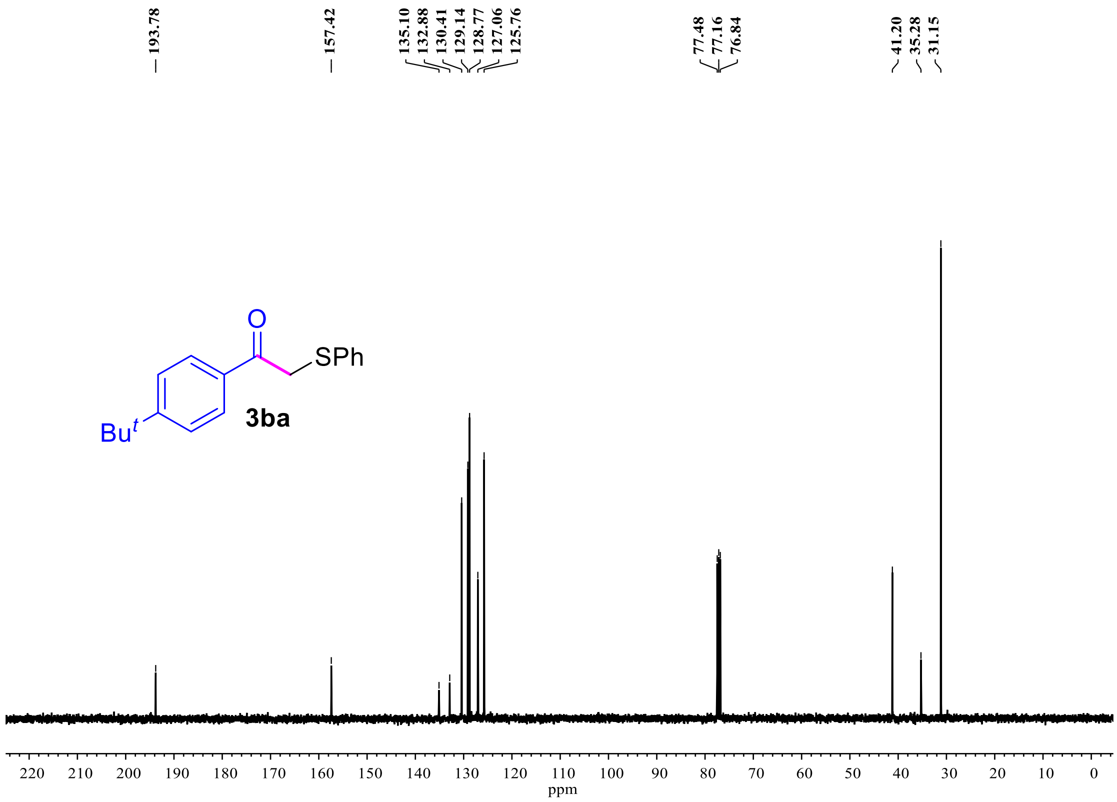

Supplementary Figure 43. ^{13}C NMR of **3ba**.

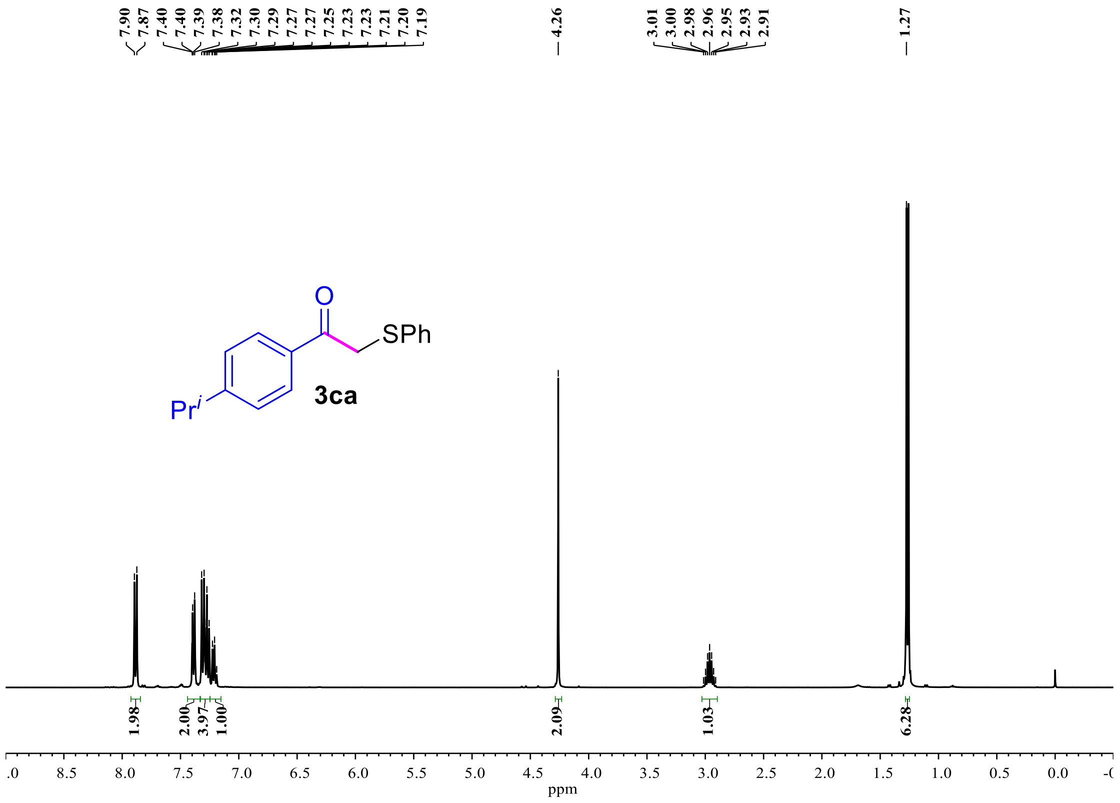

Supplementary Figure 44. ^{1}H NMR of **3ca**.

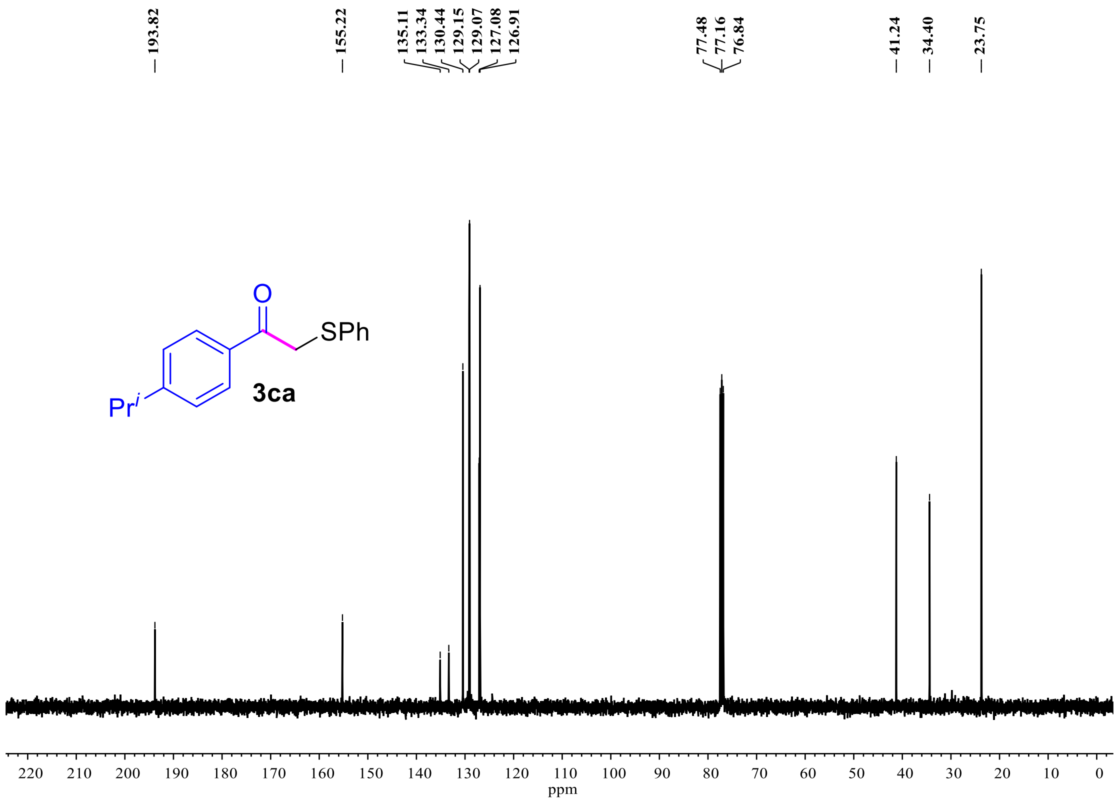

Supplementary Figure 45. ^{13}C NMR of **3ca**.

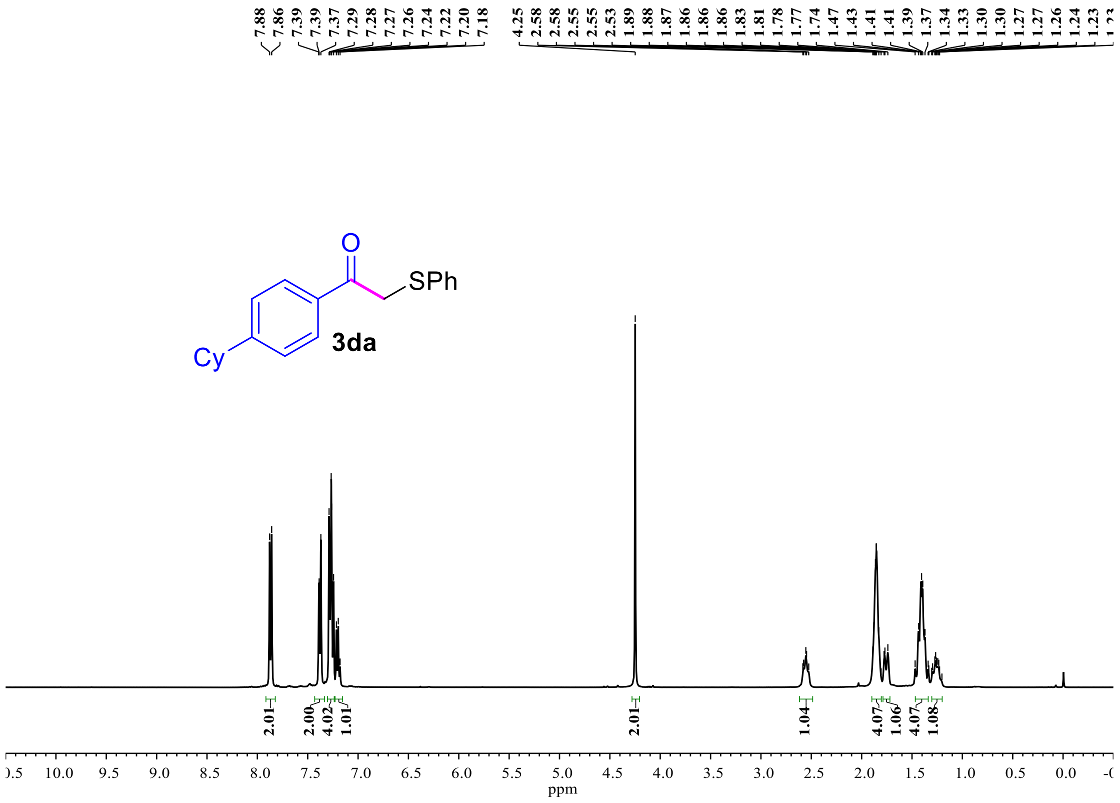

Supplementary Figure 46. ^{1}H NMR of **3da**.

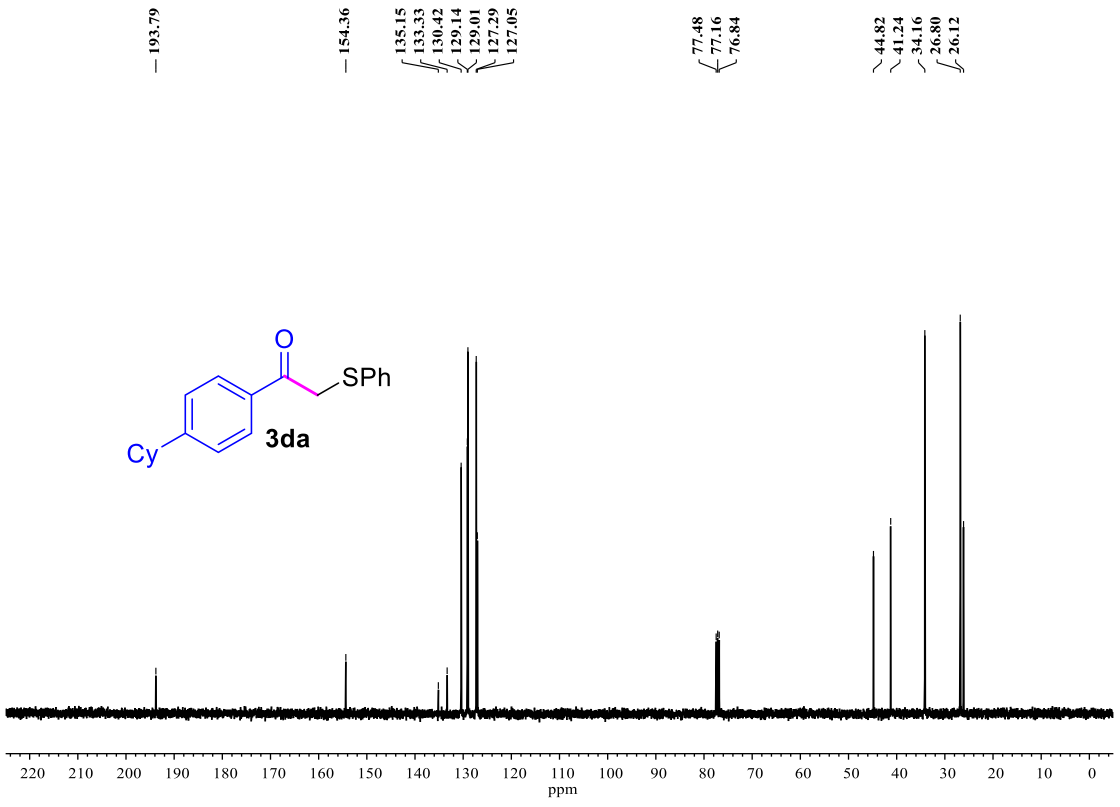

Supplementary Figure 47. ^{13}C NMR of **3da**.

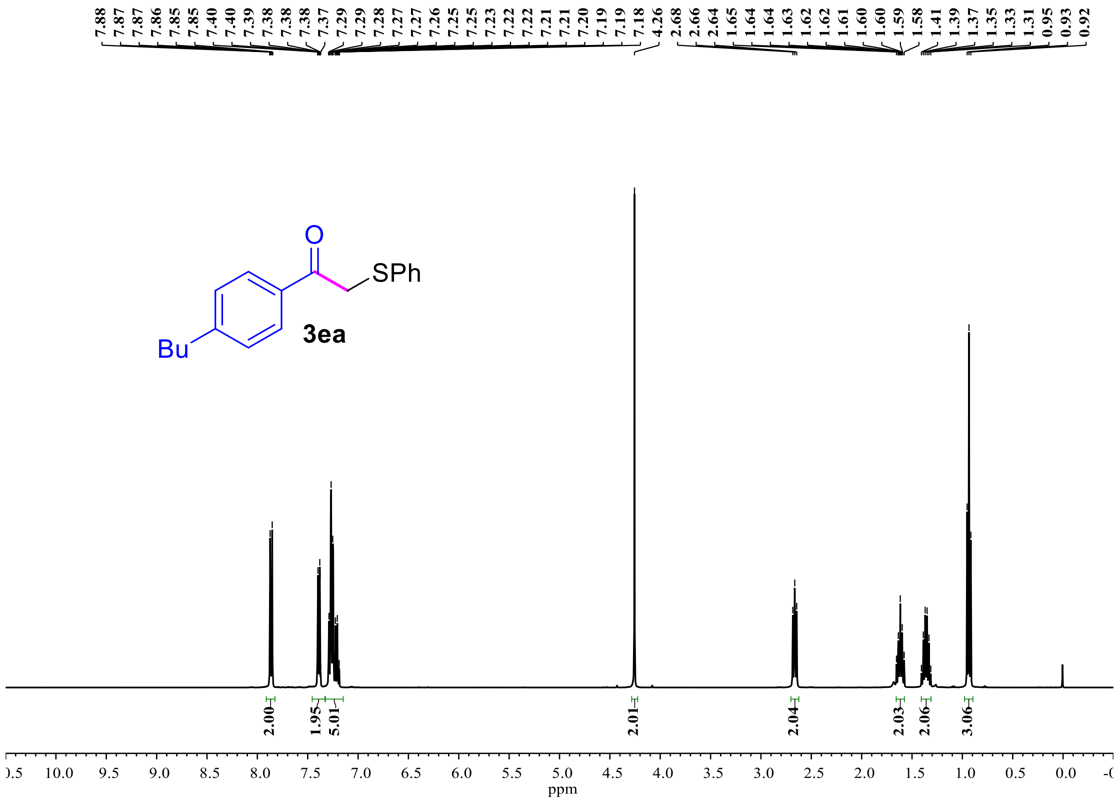

Supplementary Figure 48. ^{1}H NMR of **3ea**.

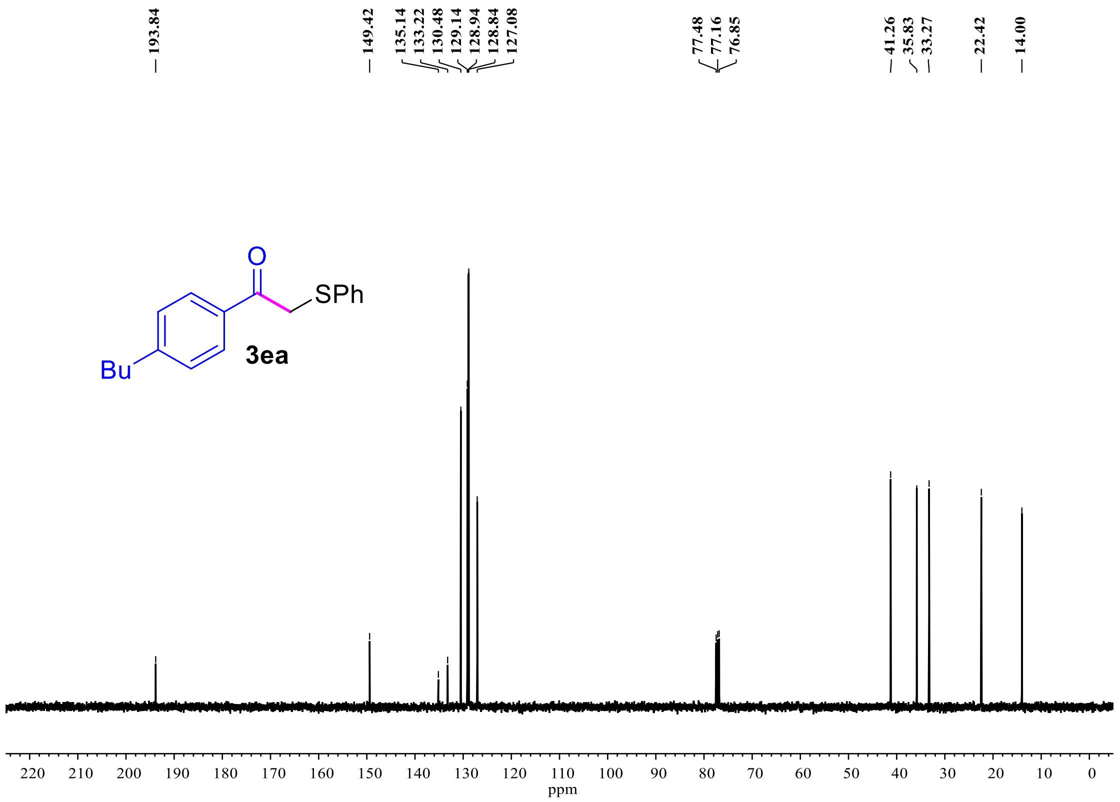

Supplementary Figure 49. ^{13}C NMR of **3ea**.

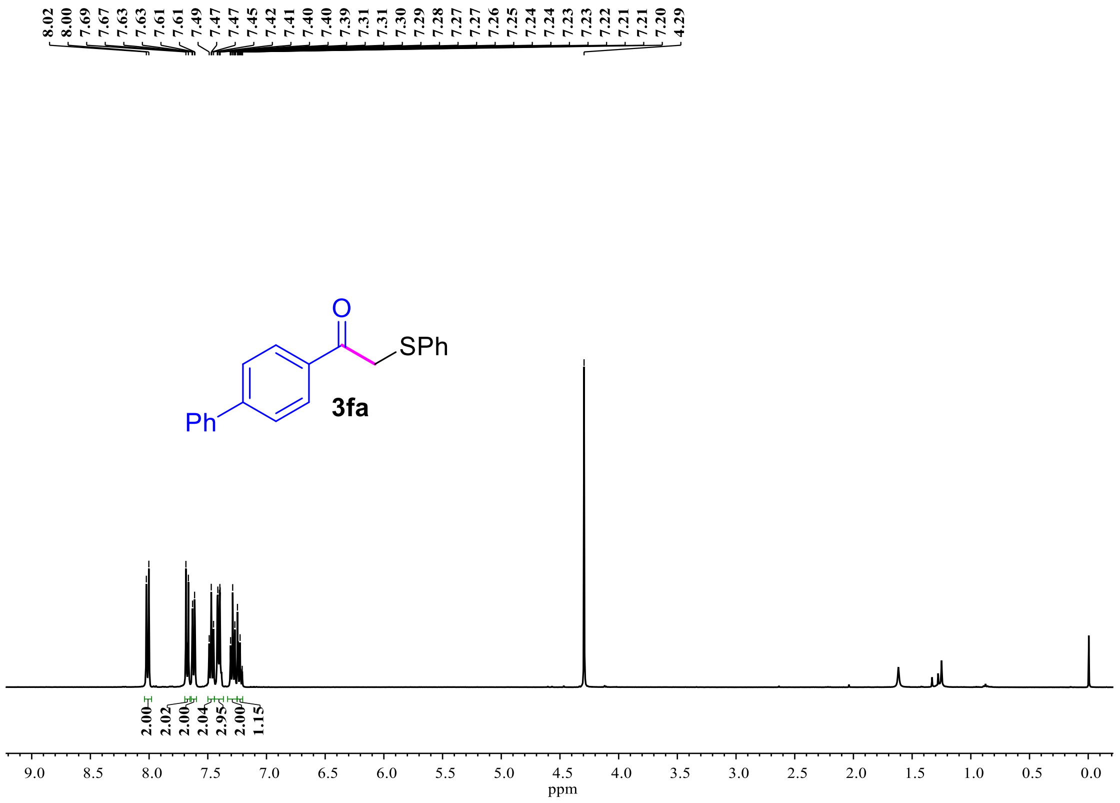

Supplementary Figure 50. ^{1}H NMR of **3fa**.

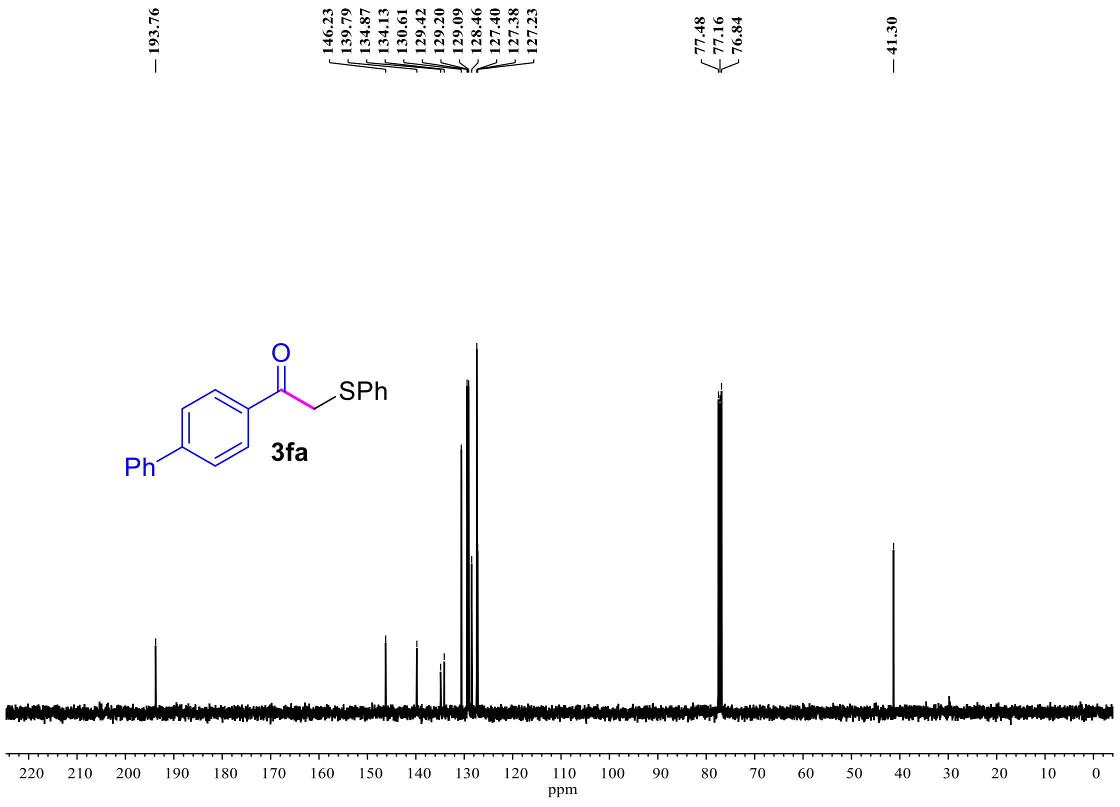

Supplementary Figure 51. ^{13}C NMR of **3fa**.

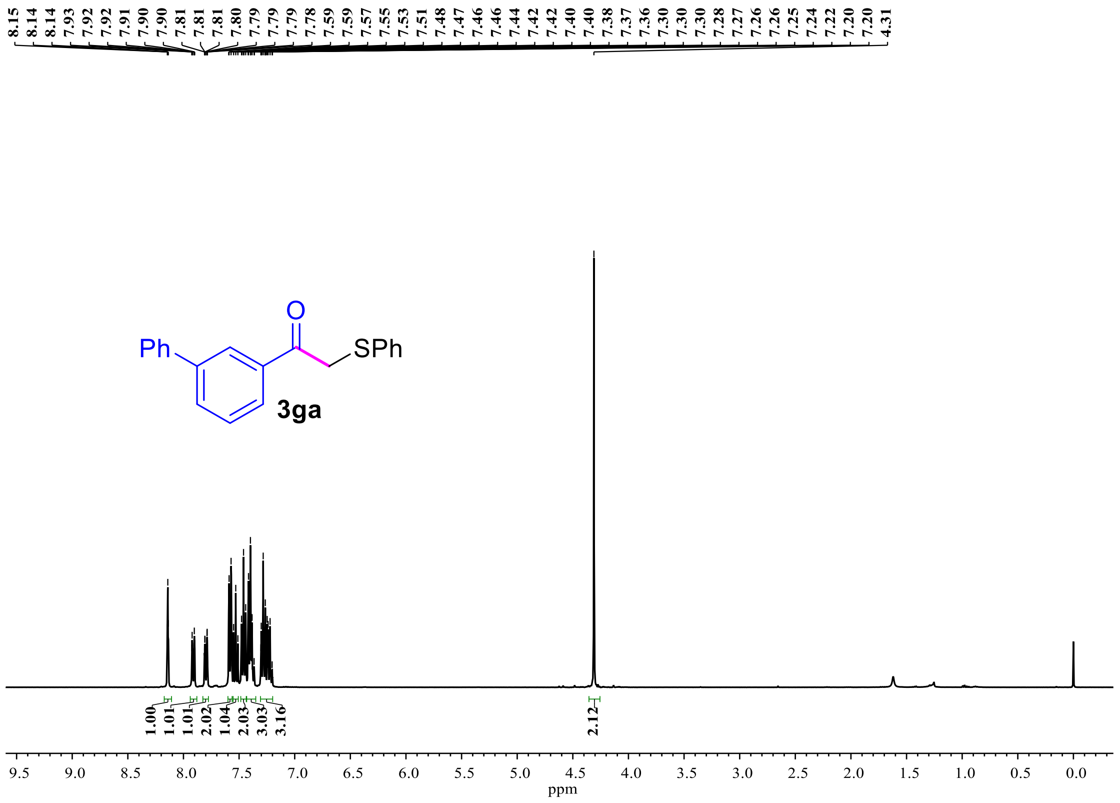

Supplementary Figure 52. ^{1}H NMR of **3ga**.

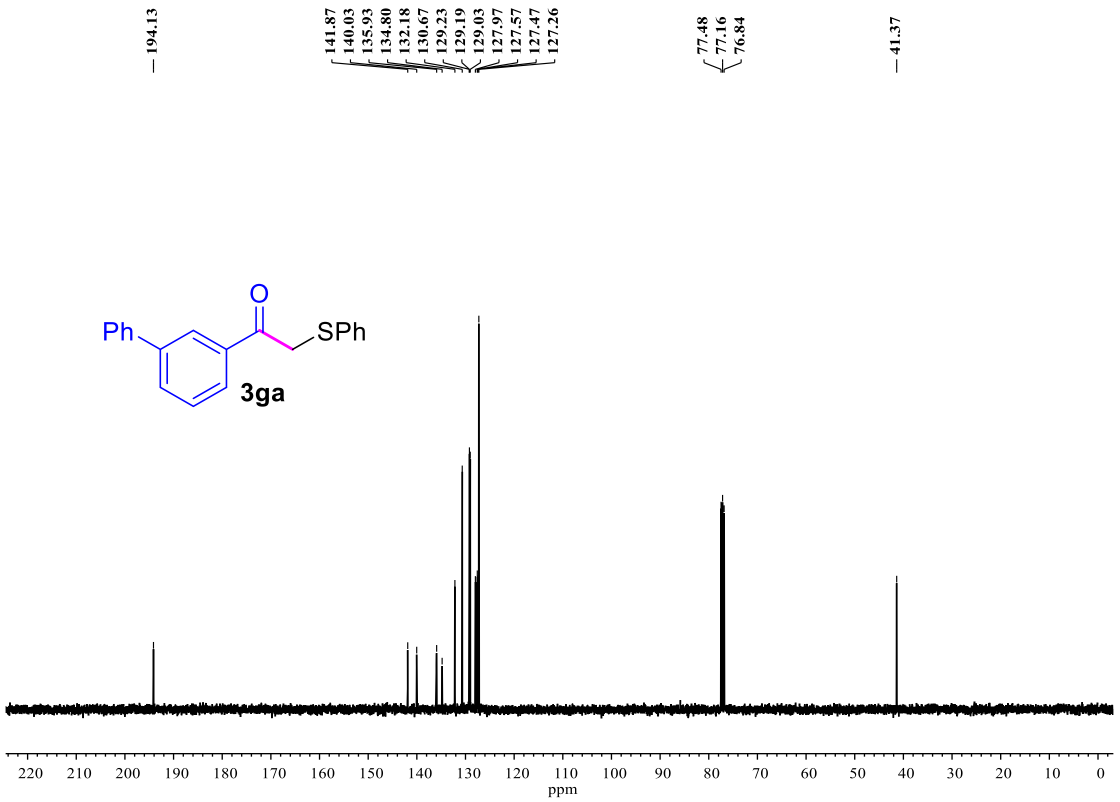

Supplementary Figure 53. ^{13}C NMR of **3ga**.

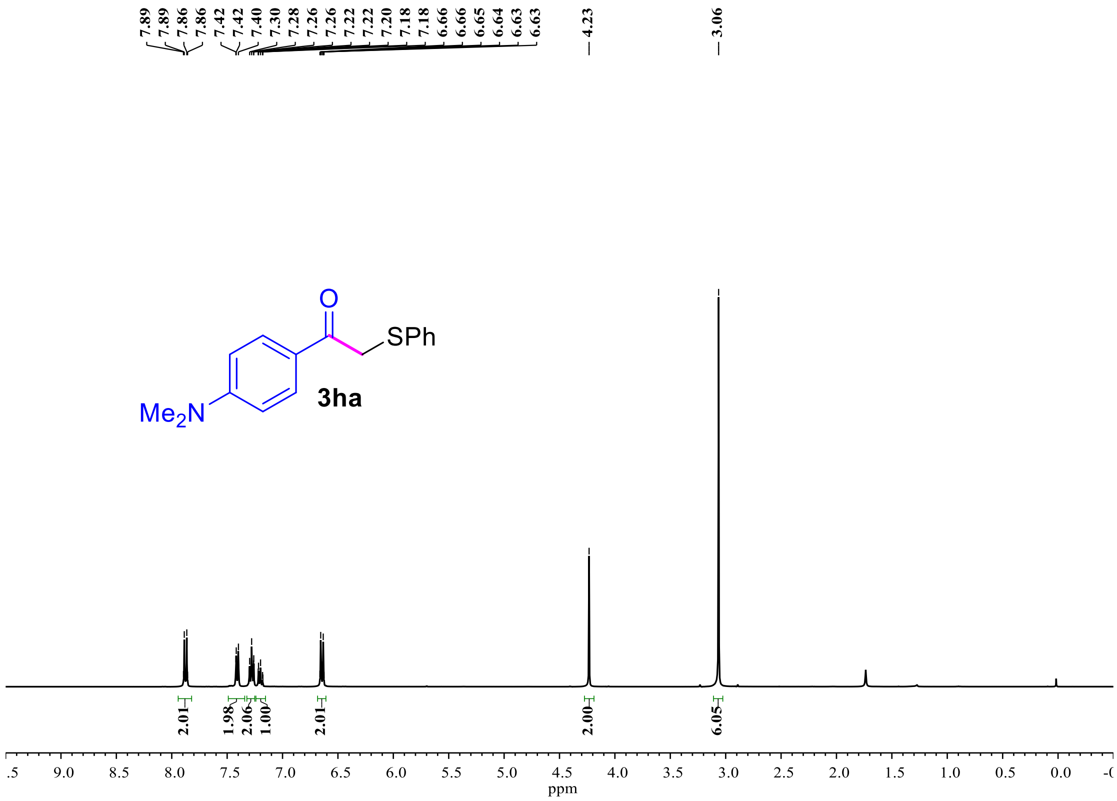

Supplementary Figure 54. ^{1}H NMR of **3ha**.

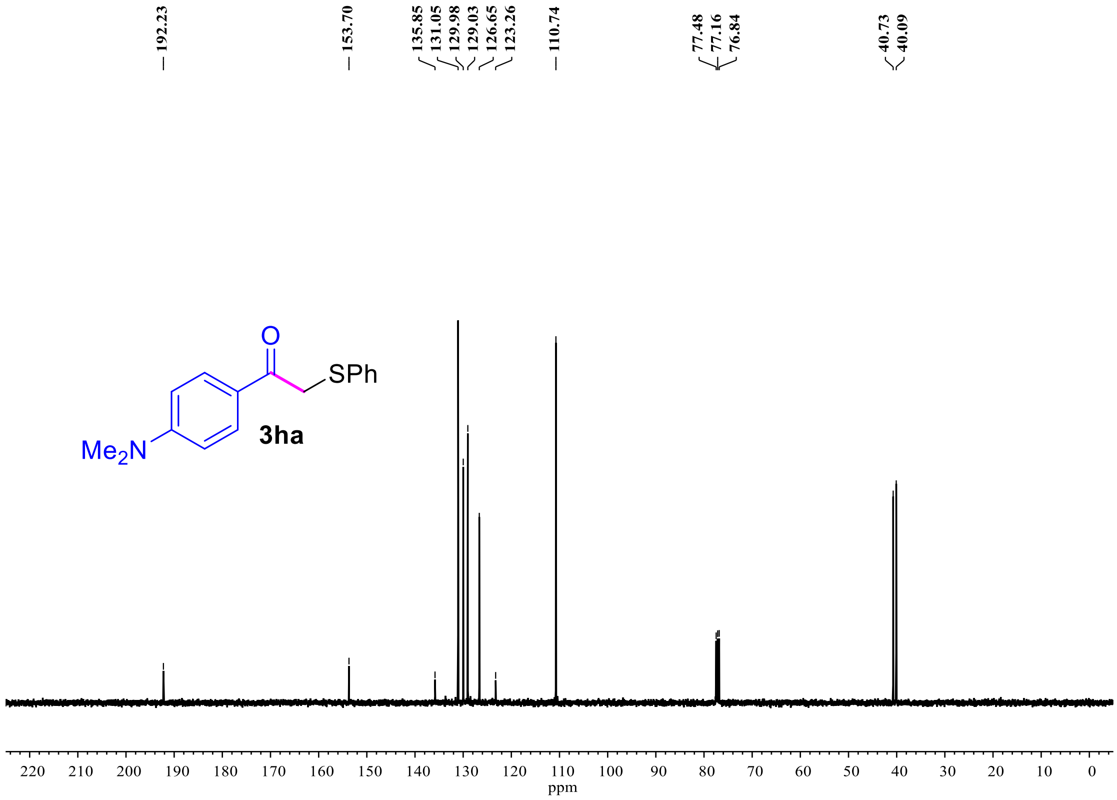

Supplementary Figure 55. ^{13}C NMR of **3ha**.

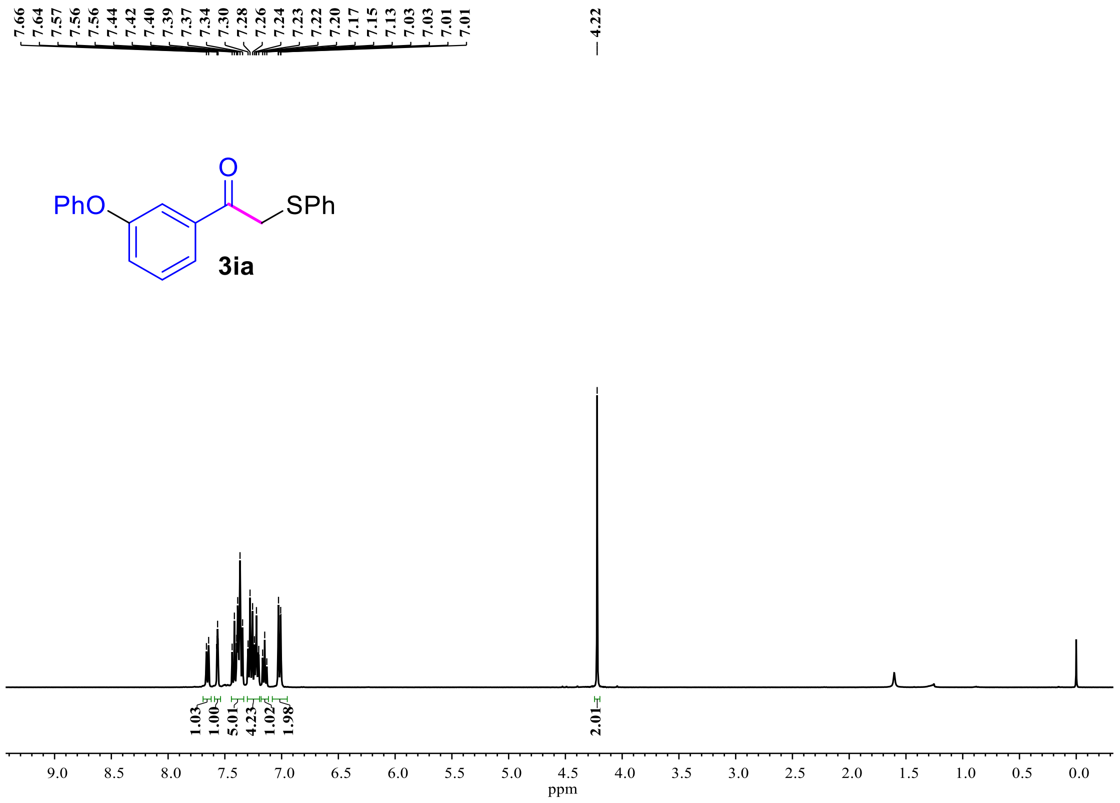

Supplementary Figure 56. ^{1}H NMR of **3ia**.

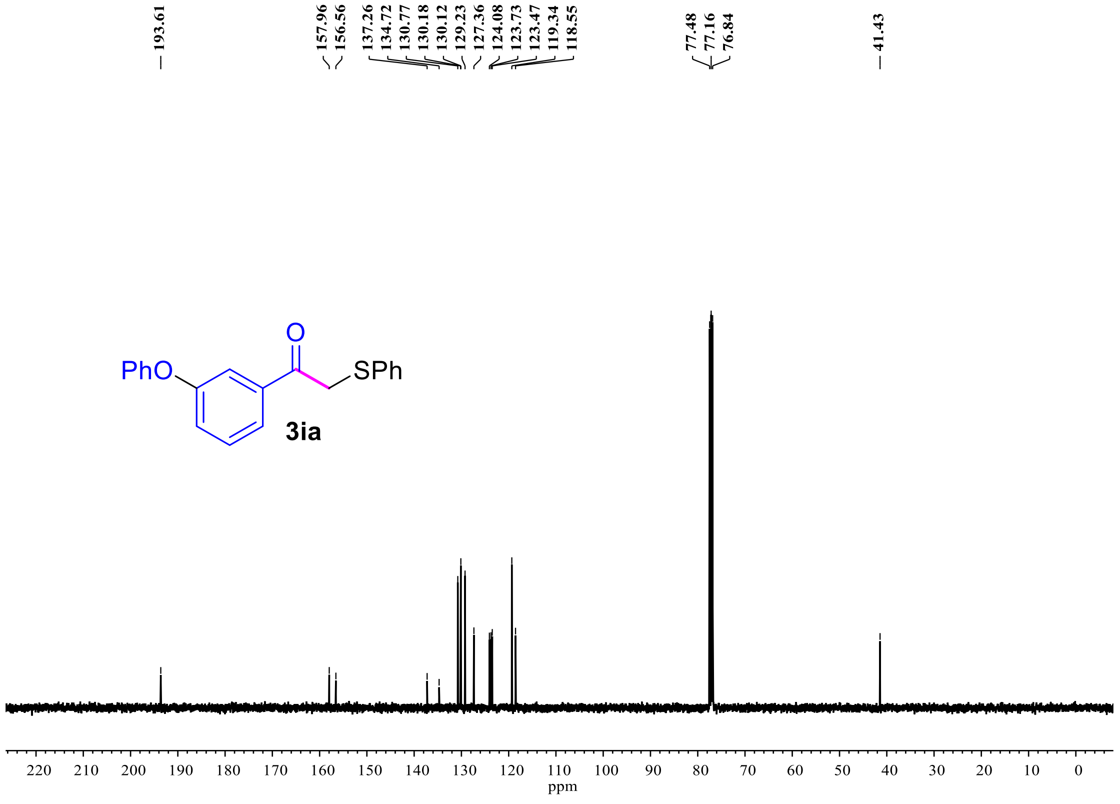

Supplementary Figure 57. ^{13}C NMR of **3ia**.

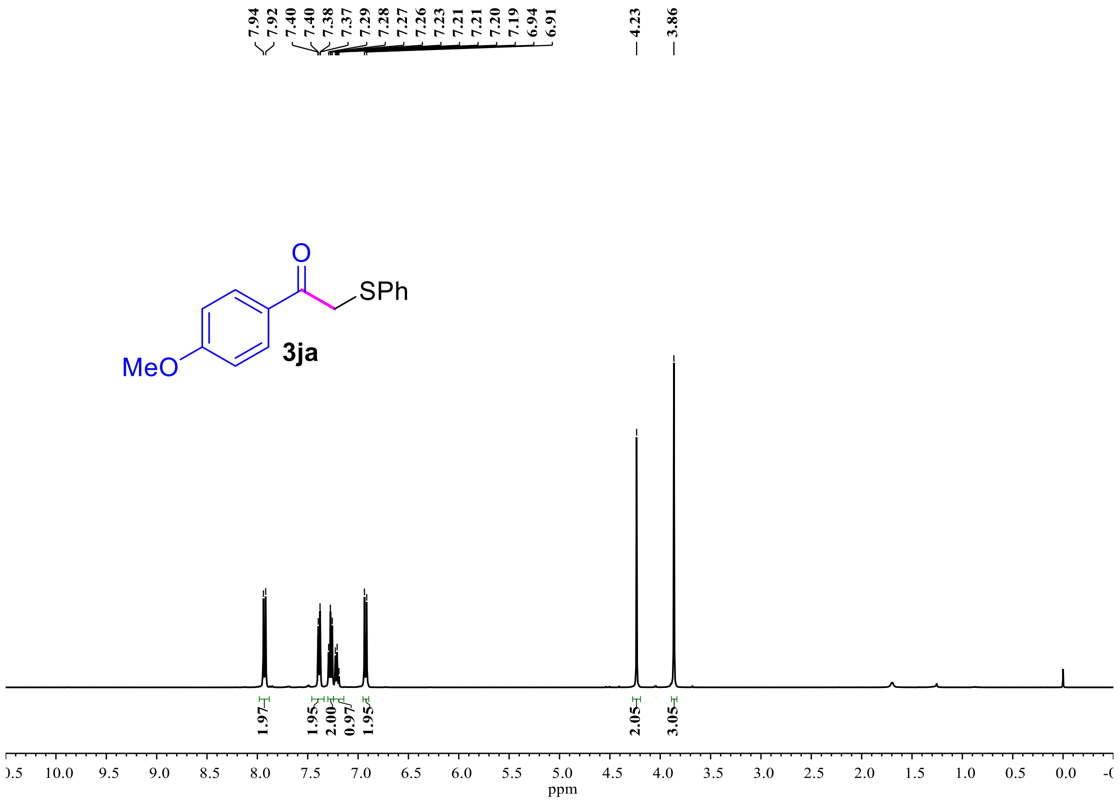

Supplementary Figure 58. ^{1}H NMR of **3ja**.

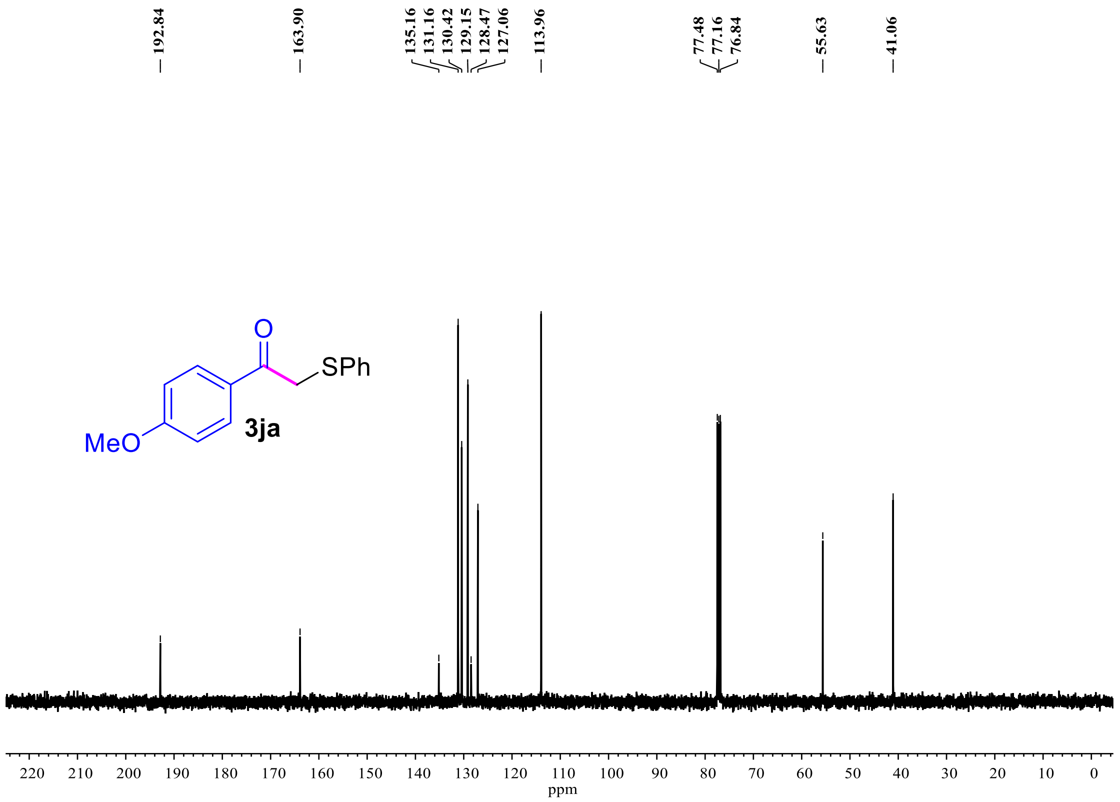

Supplementary Figure 59. ^{13}C NMR of **3ja**.

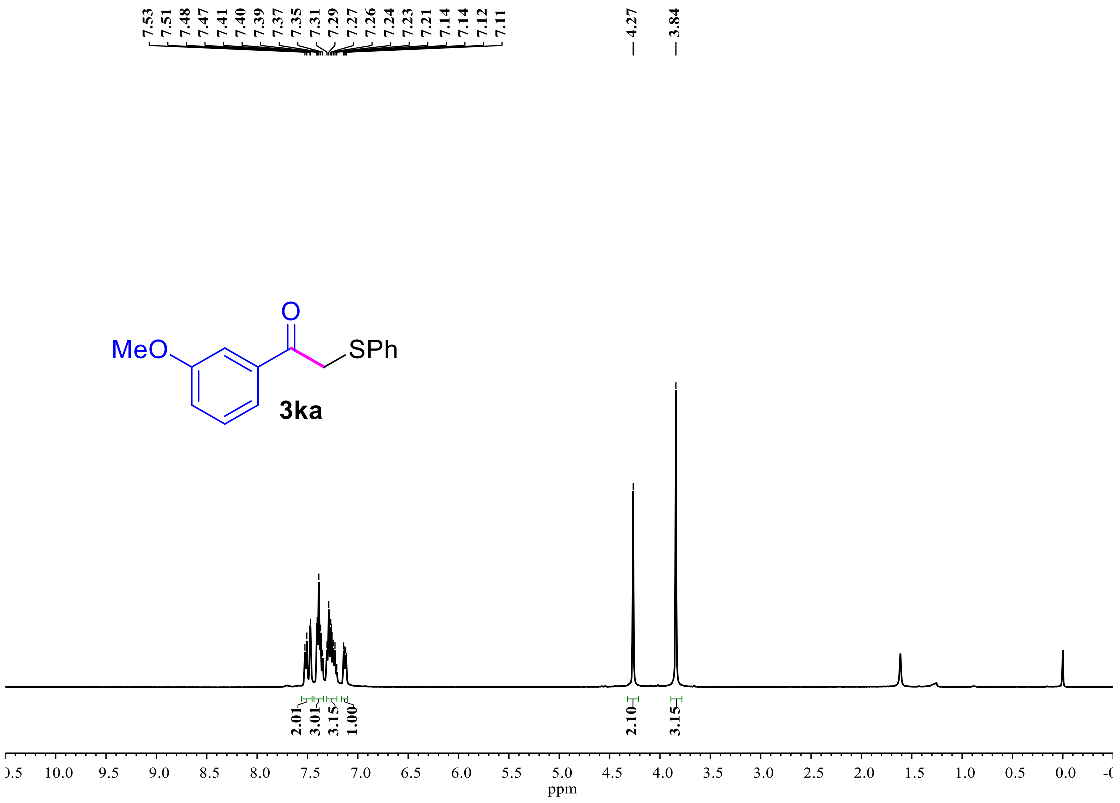

Supplementary Figure 60. ^{1}H NMR of **3ka**.

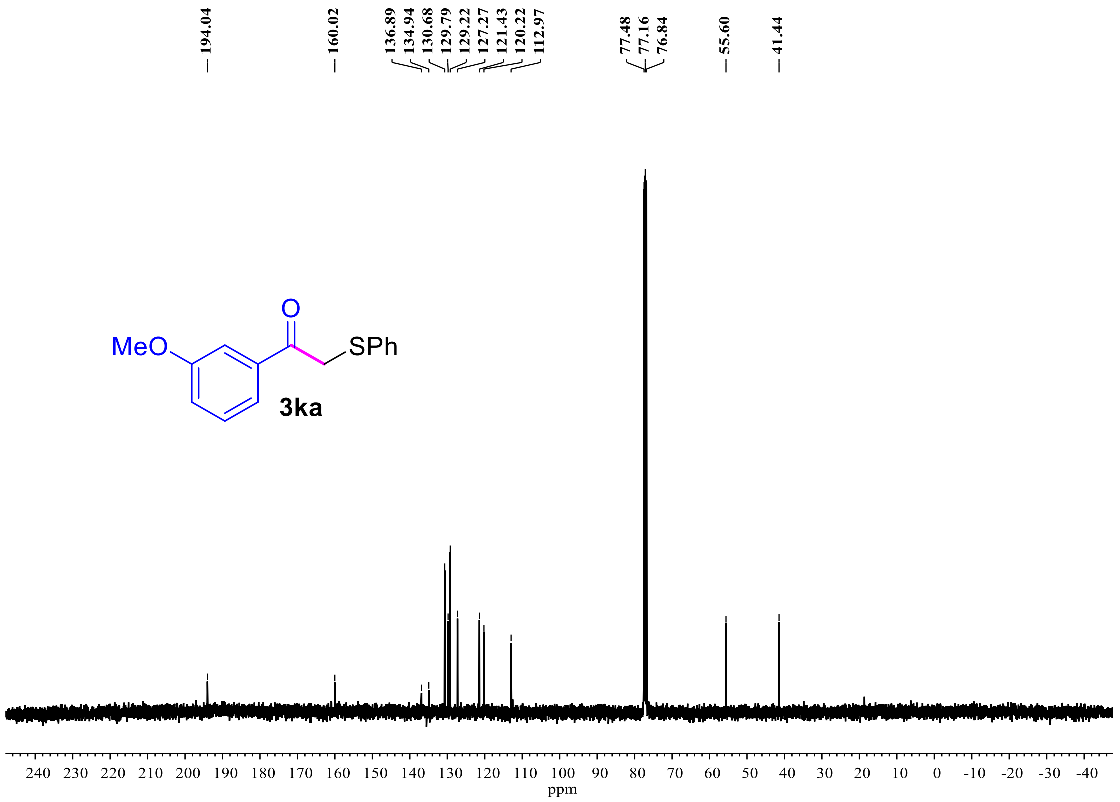

Supplementary Figure 61. ^{13}C NMR of **3ka**.

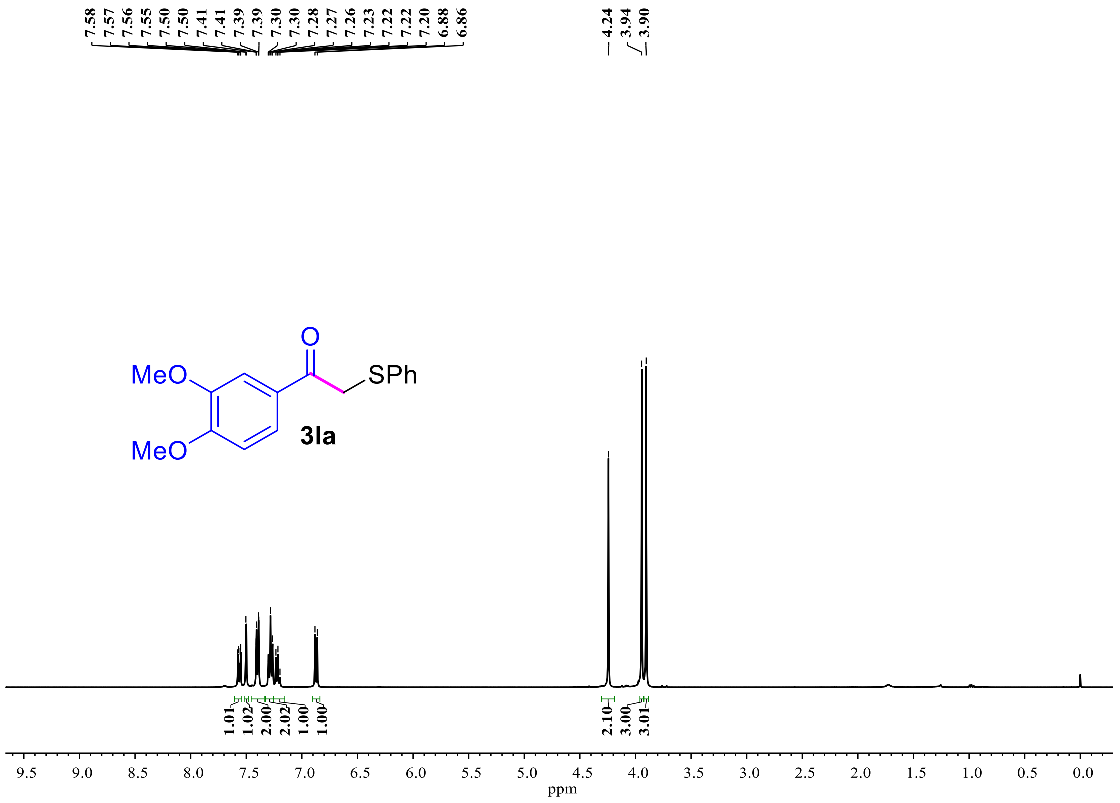

Supplementary Figure 62. ^{1}H NMR of **3la**.

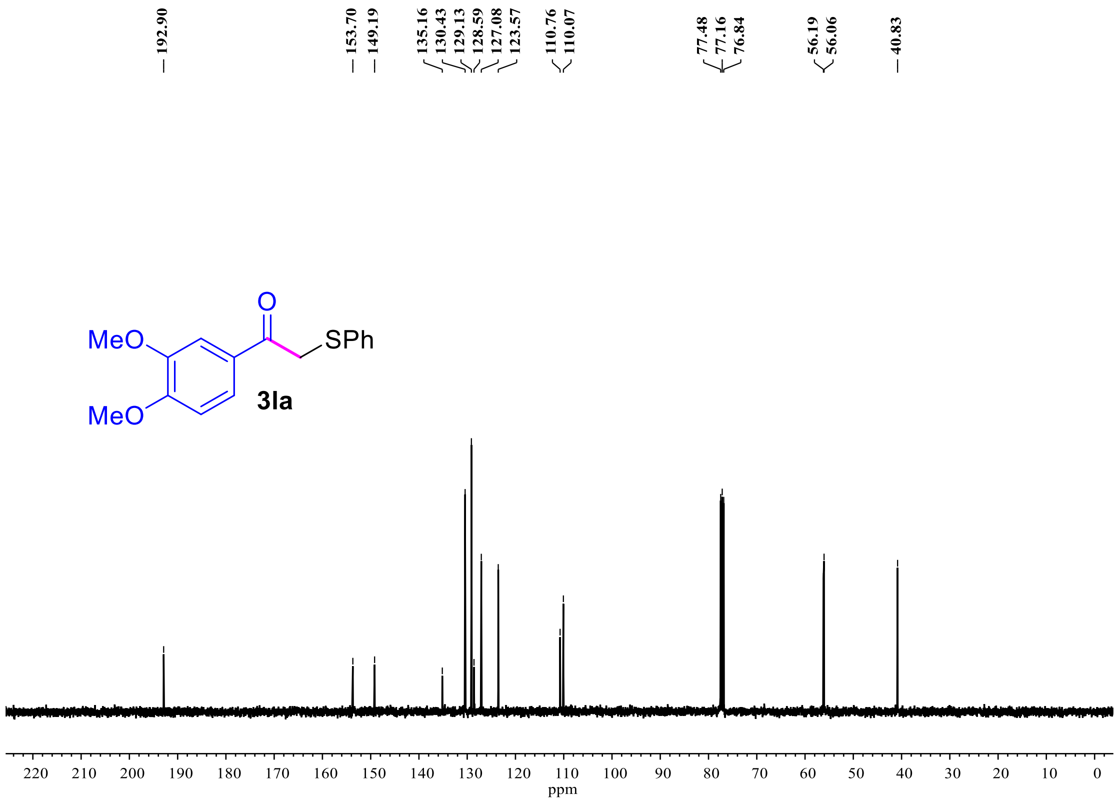

Supplementary Figure 63. ^{13}C NMR of **3la**.

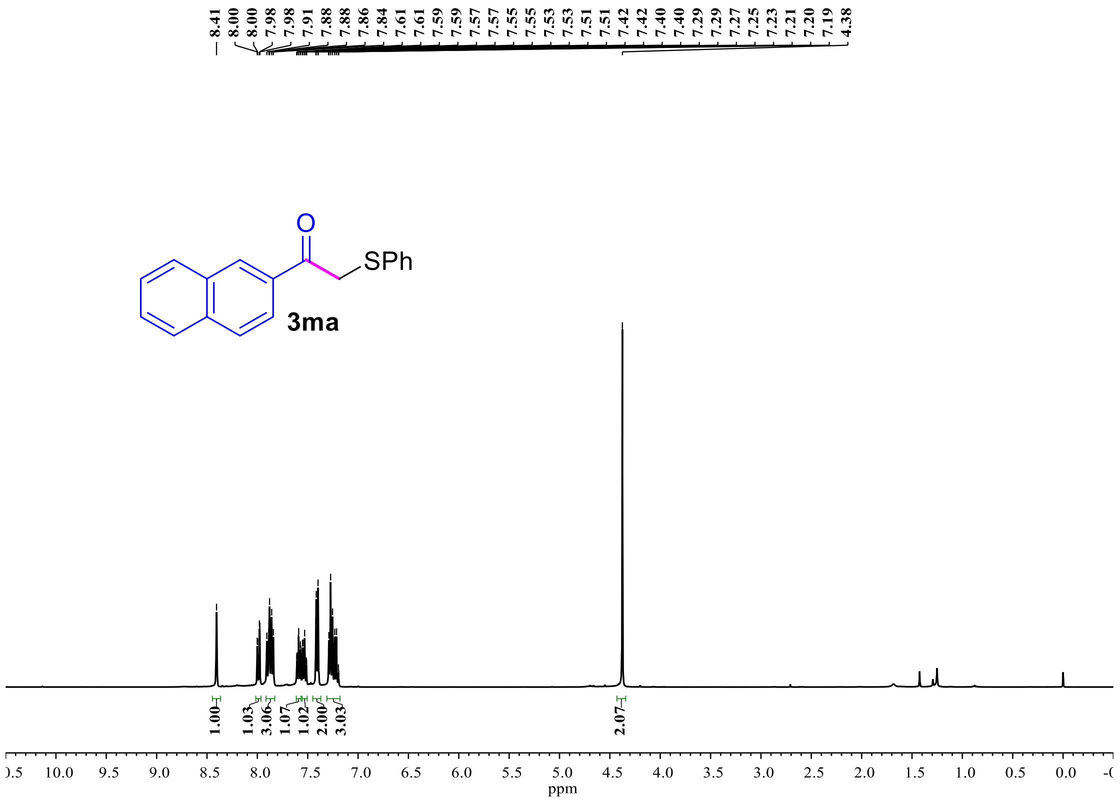

Supplementary Figure 64. ^{1}H NMR of **3ma**.

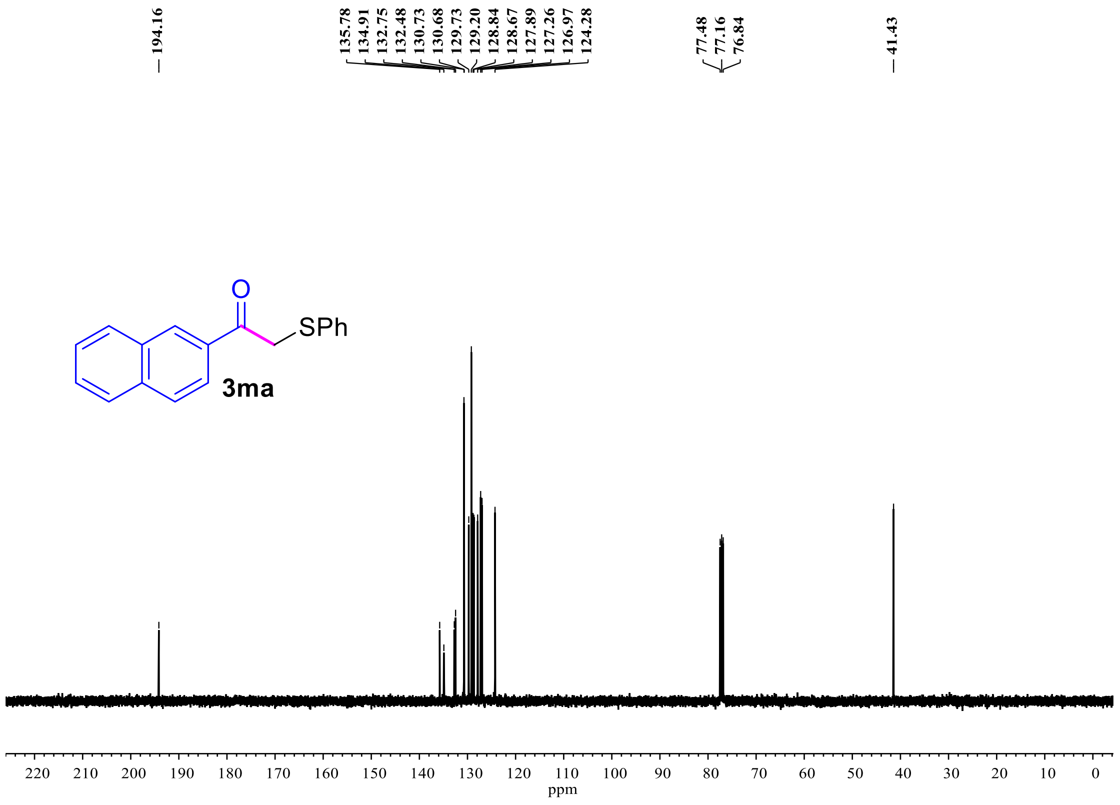

Supplementary Figure 65. ^{13}C NMR of **3ma**.

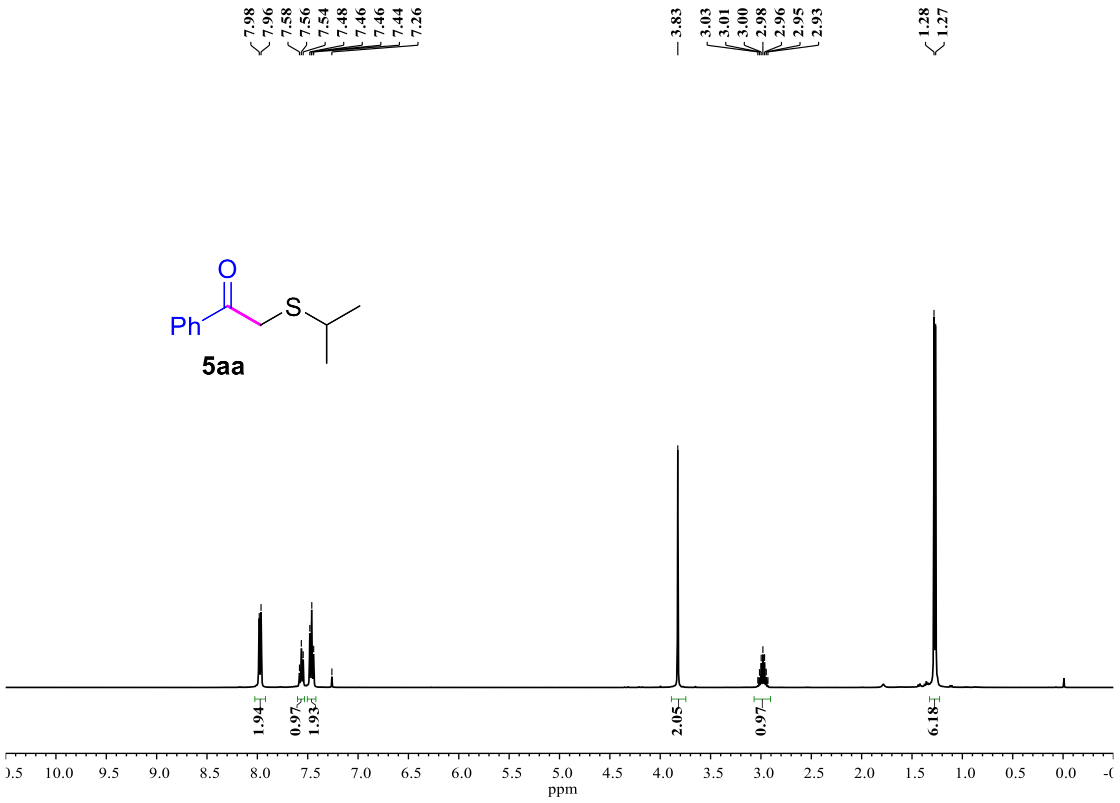

Supplementary Figure 66. ^{1}H NMR of **5aa**.

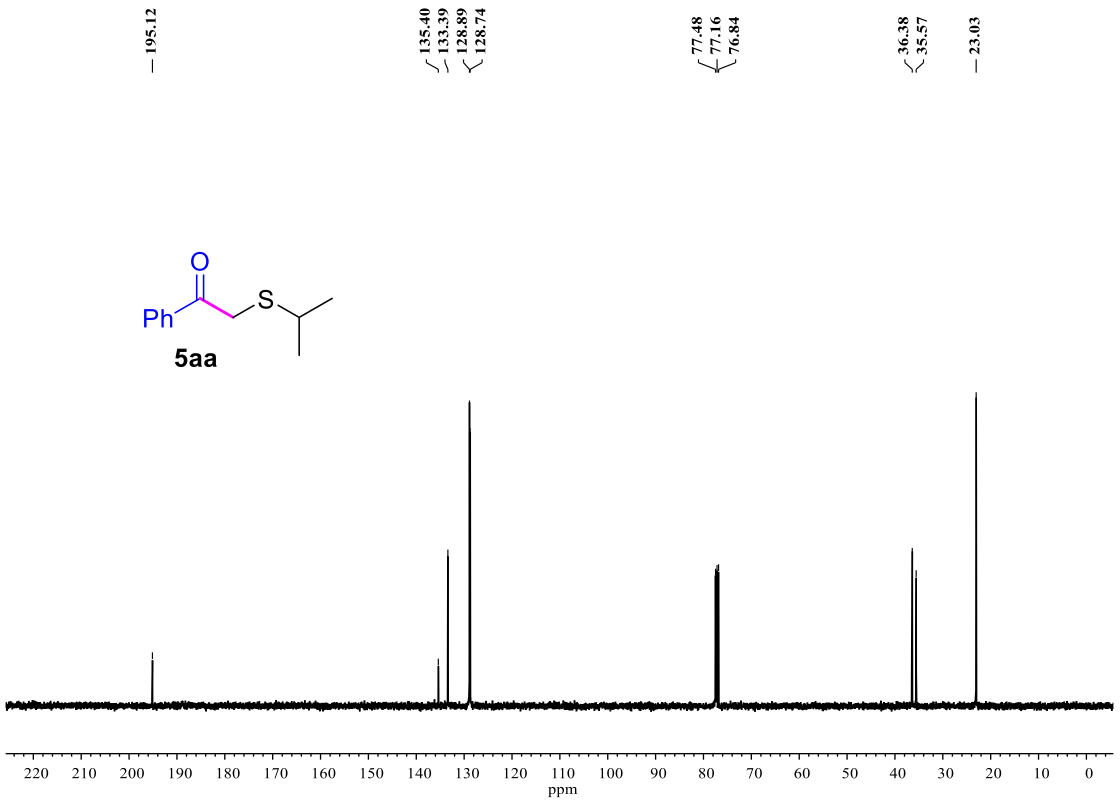

Supplementary Figure 67. ^{13}C NMR of **5aa**.

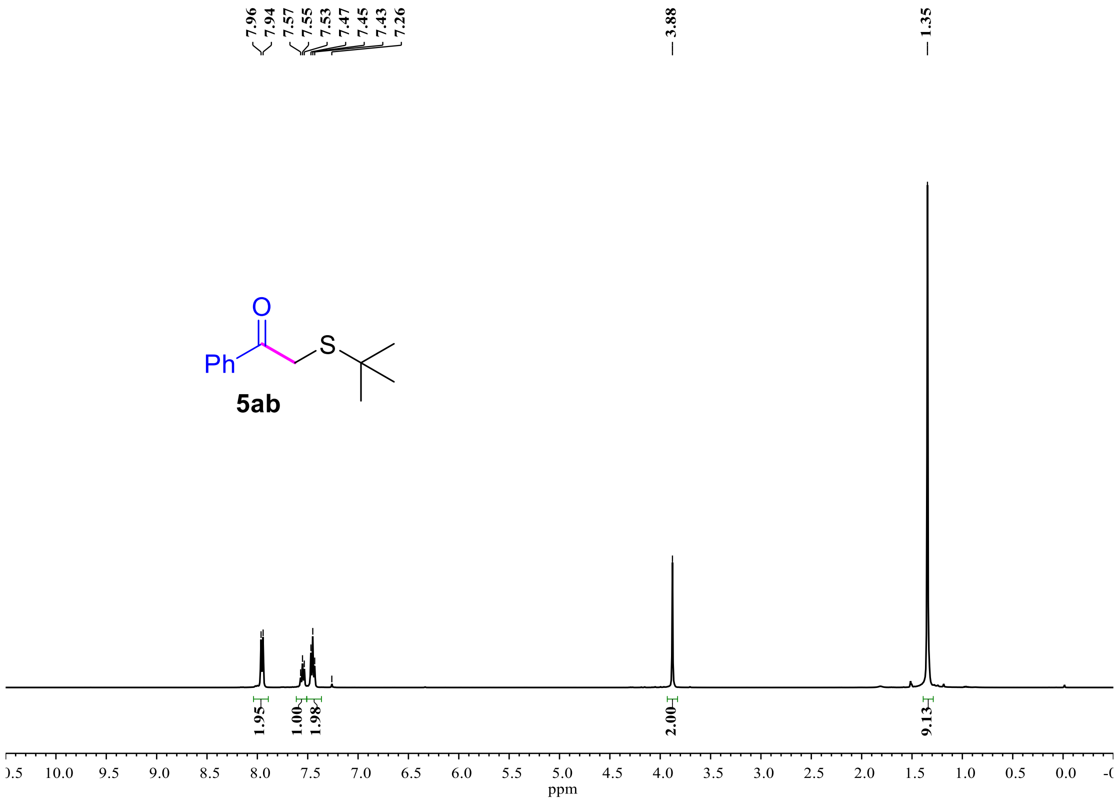

Supplementary Figure 68. ^{1}H NMR of **5ab**.

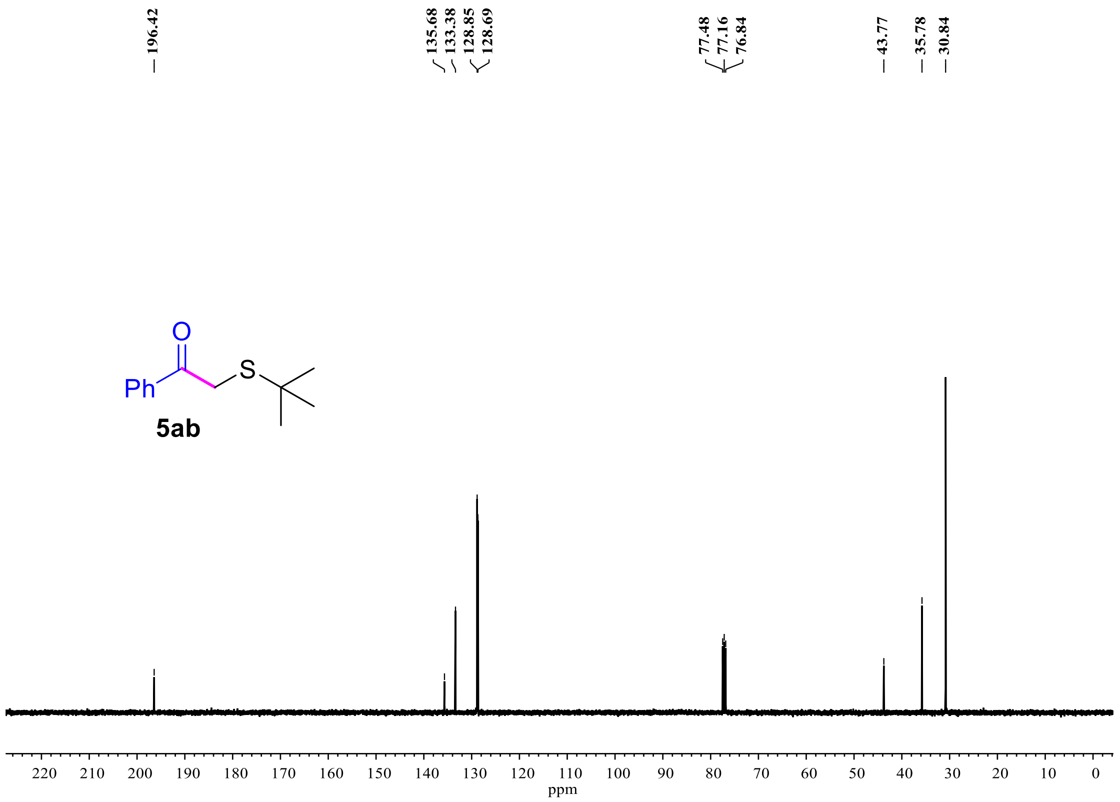

Supplementary Figure 69. ^{13}C NMR of **5ab**.

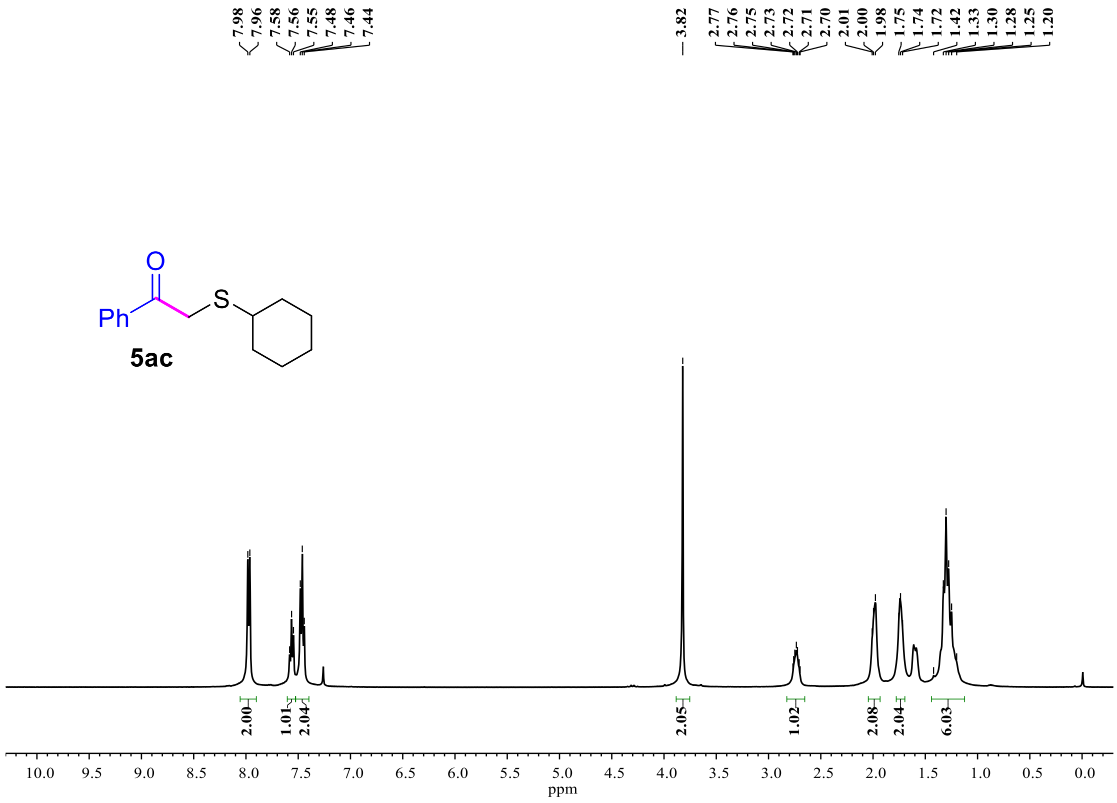

Supplementary Figure 70. ^{1}H NMR of **5ac**.

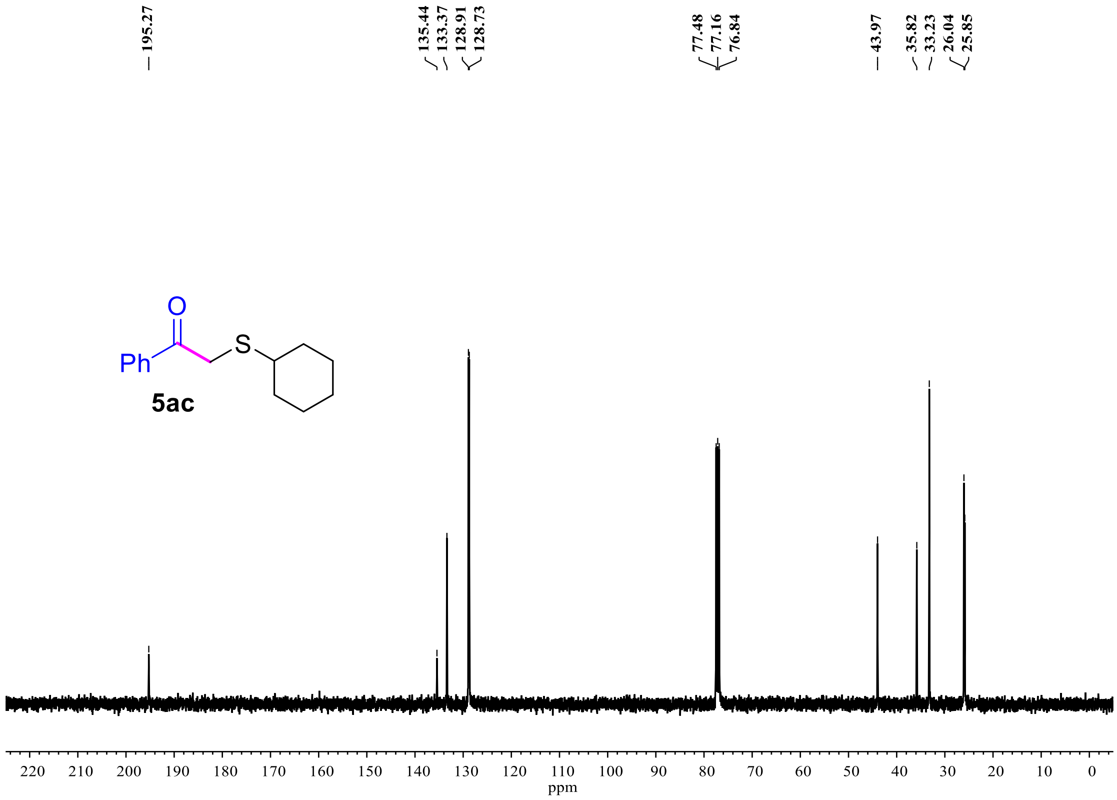

Supplementary Figure 71. ^{13}C NMR of **5ac**.

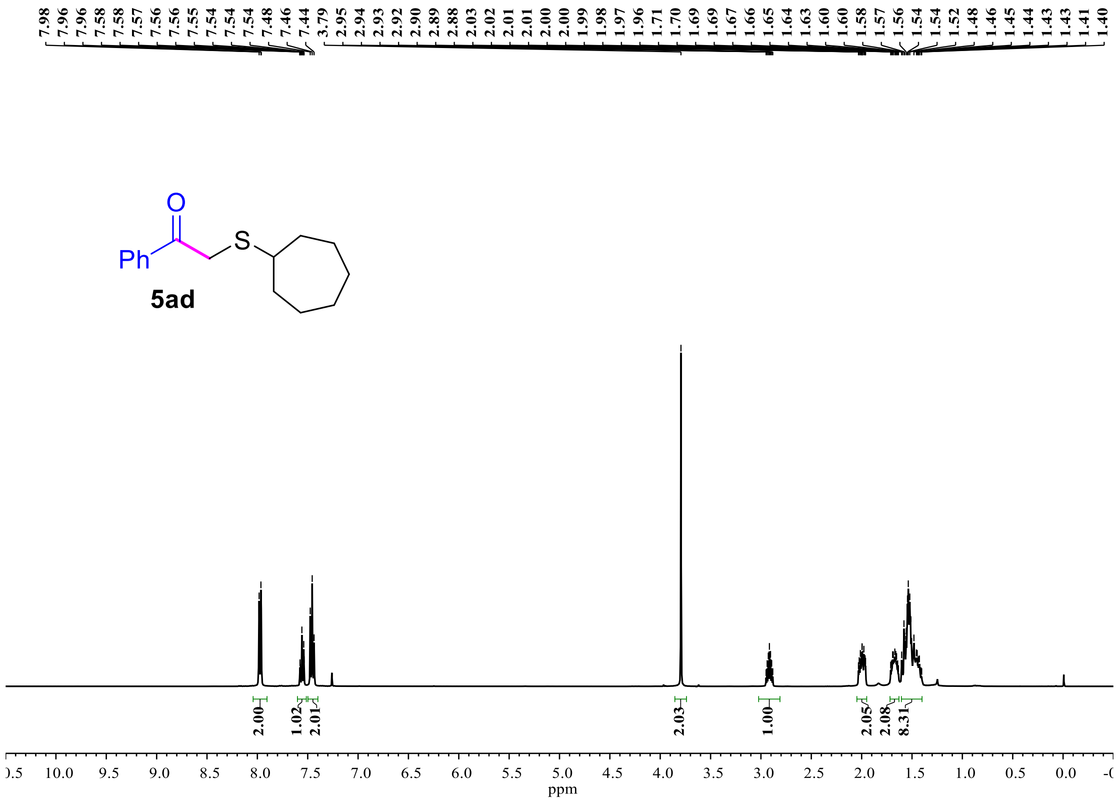

Supplementary Figure 72. ^{1}H NMR of **5ad**.

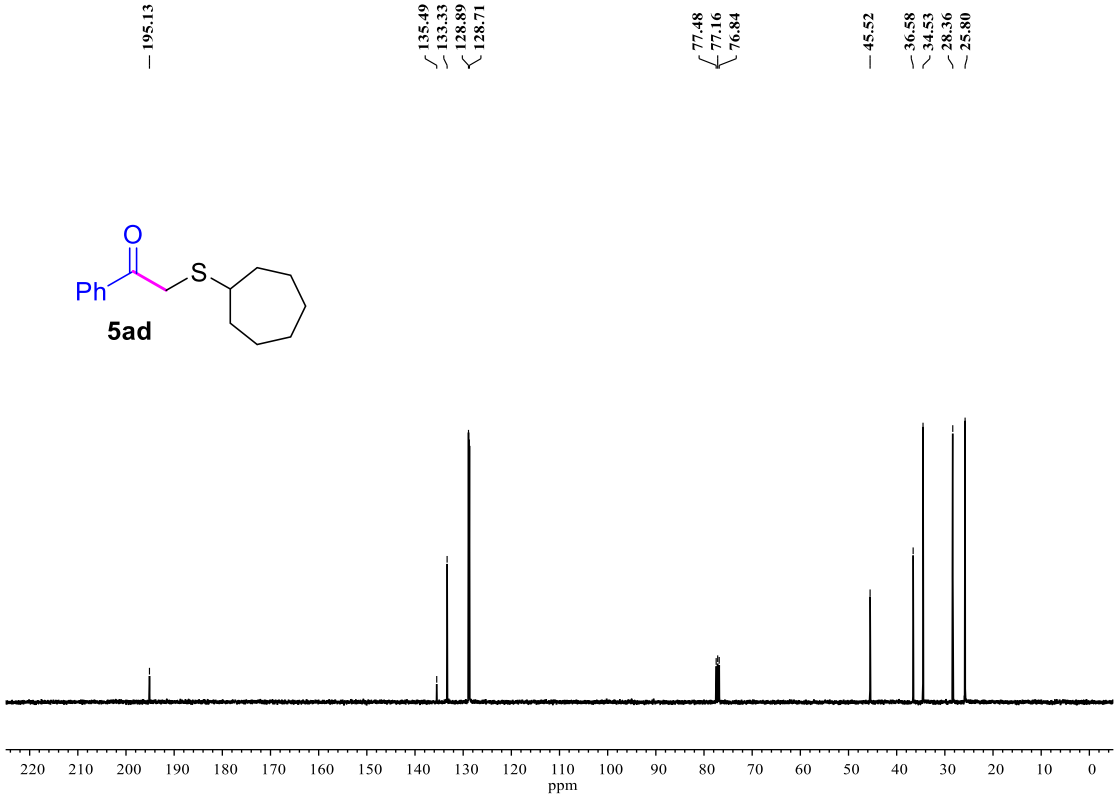

Supplementary Figure 73. ^{13}C NMR of **5ad**.

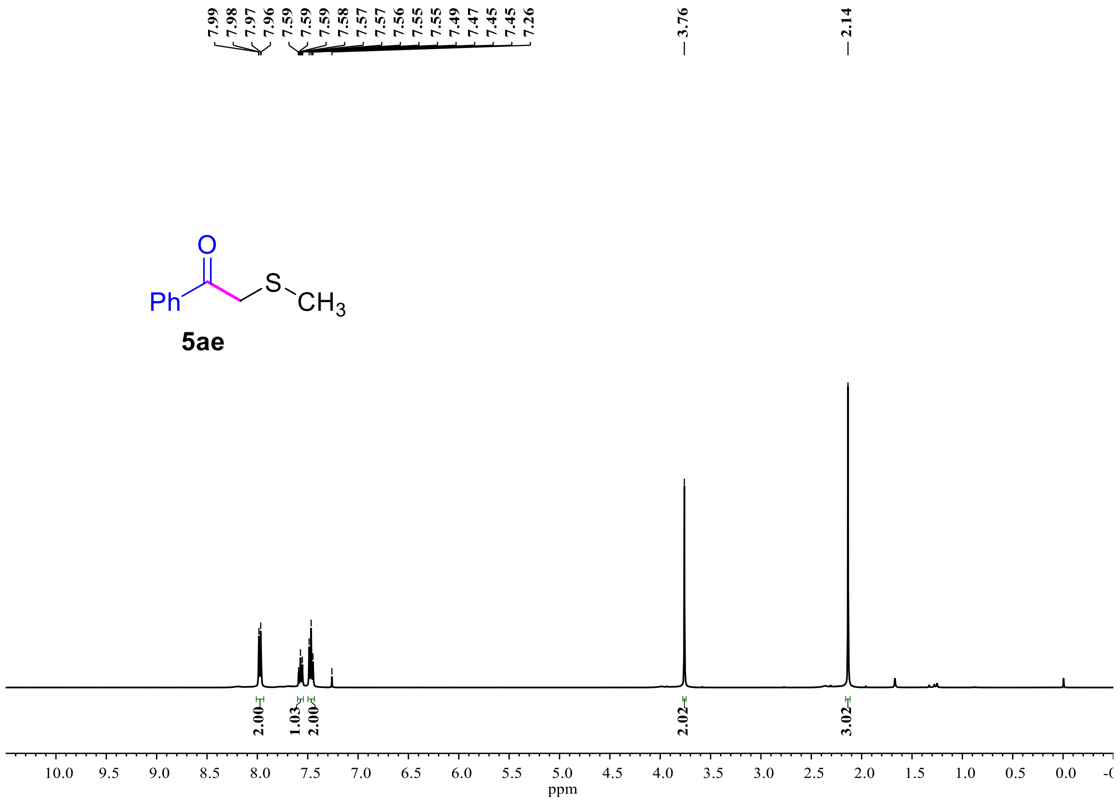

Supplementary Figure 74. ^{1}H NMR of **5ae**.

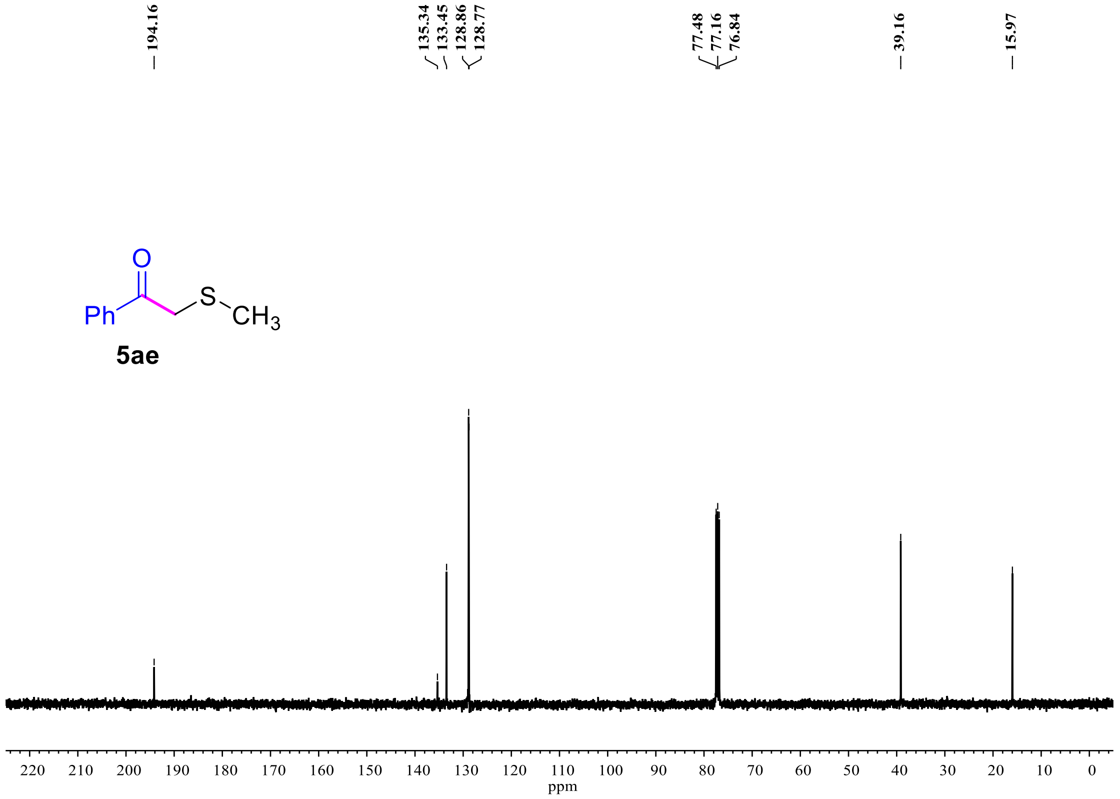

Supplementary Figure 75. ^{13}C NMR of **5ae**.

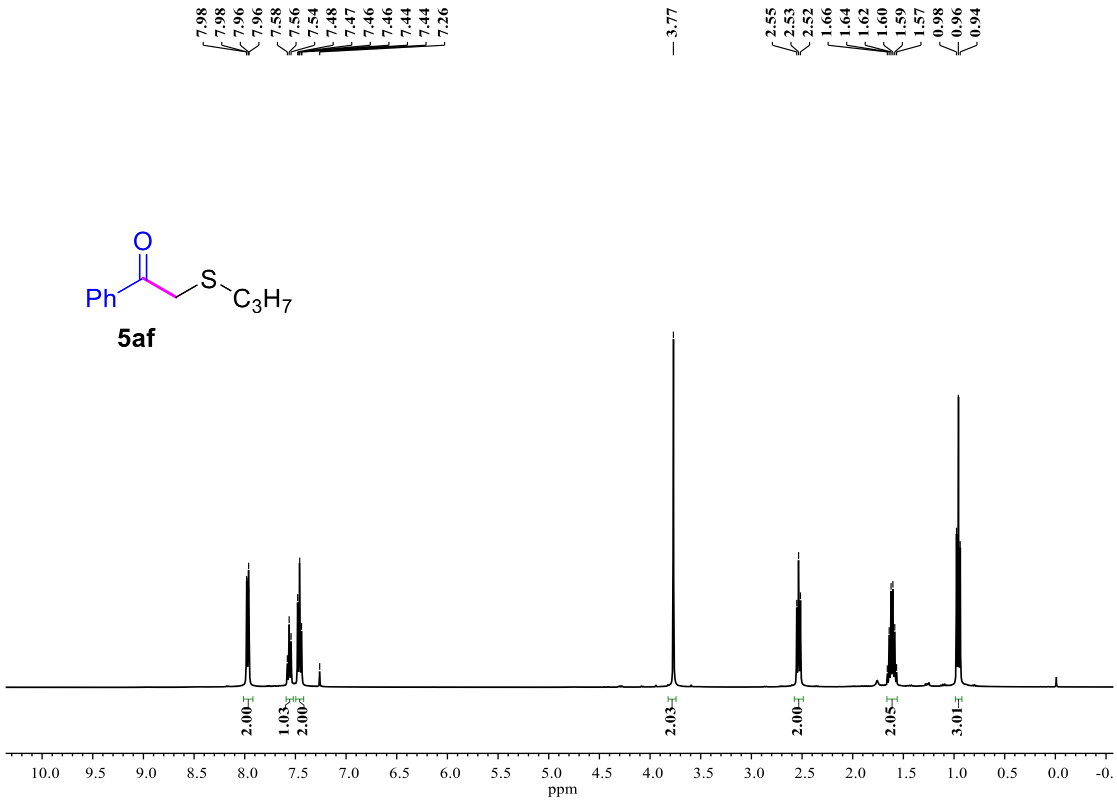

Supplementary Figure 76. ^{1}H NMR of **5af**.

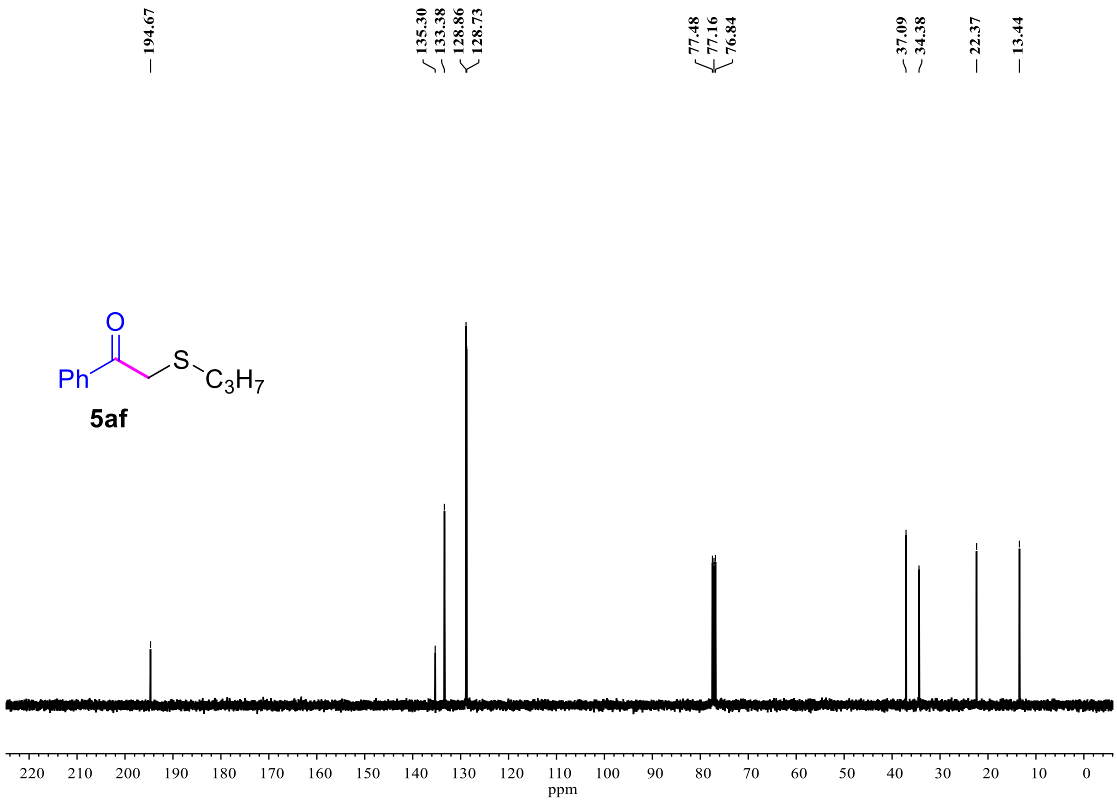

Supplementary Figure 77. ^{13}C NMR of **5af**.

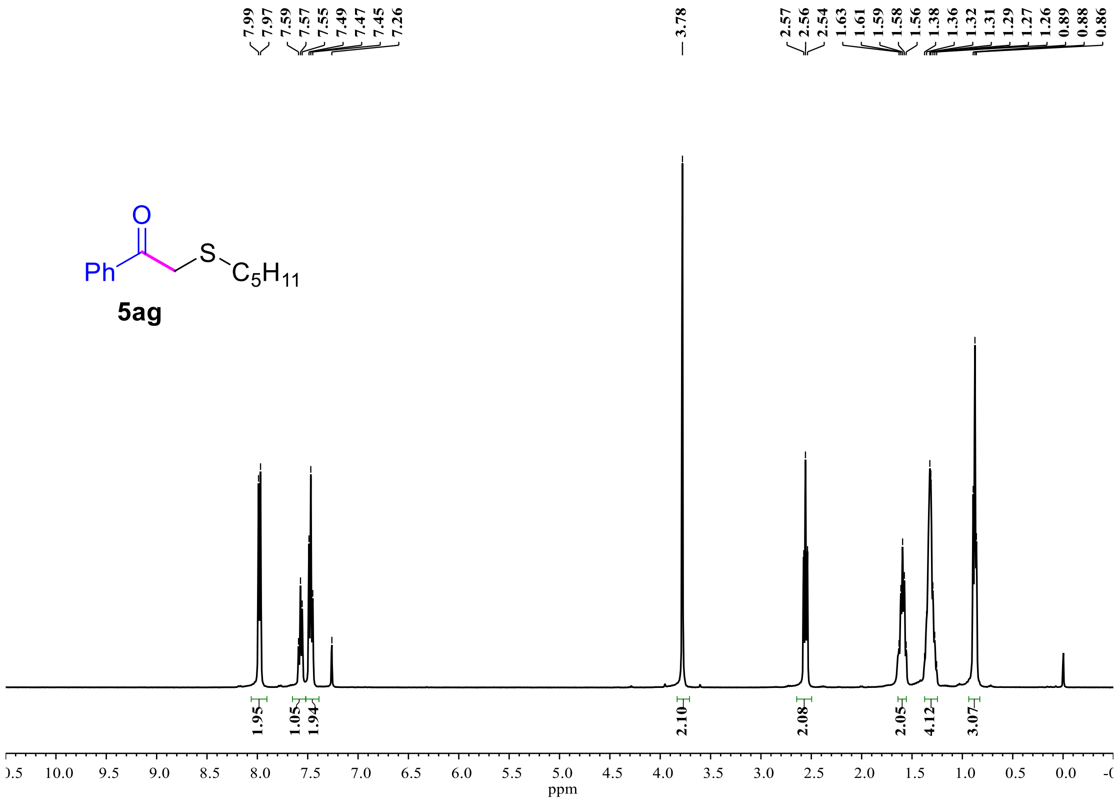

Supplementary Figure 78. ^{1}H NMR of **5ag**.

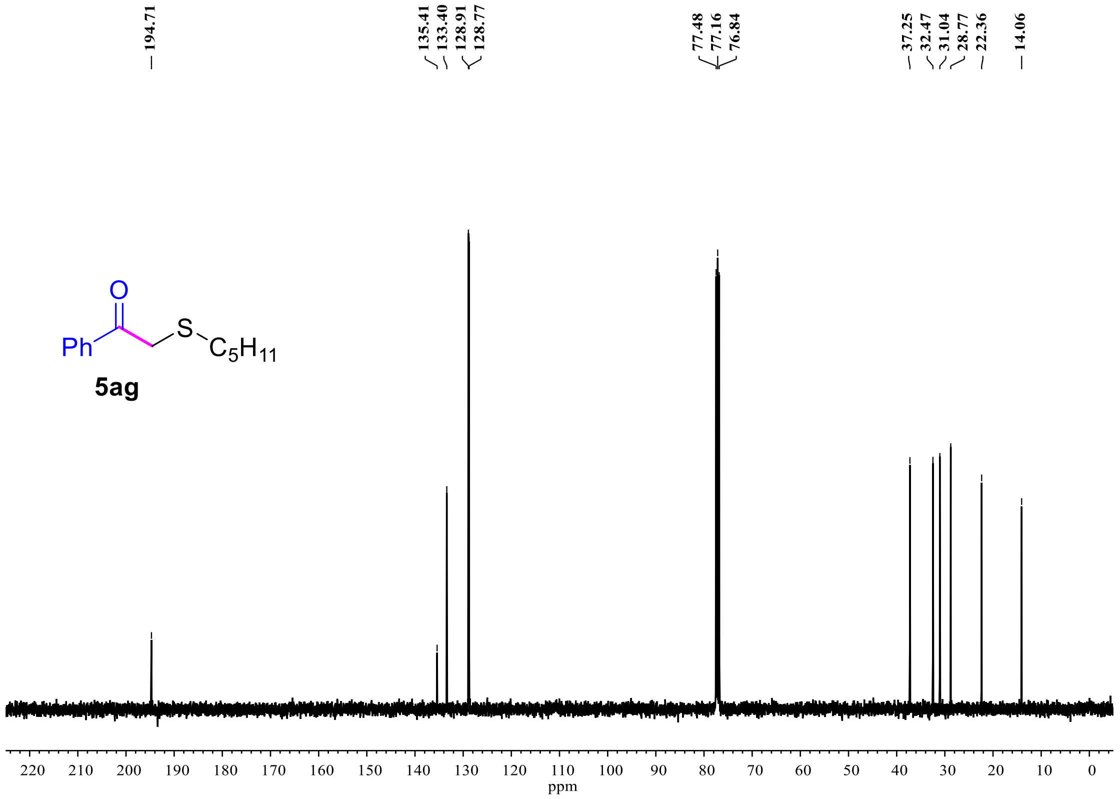

Supplementary Figure 79. ^{13}C NMR of **5ag**.

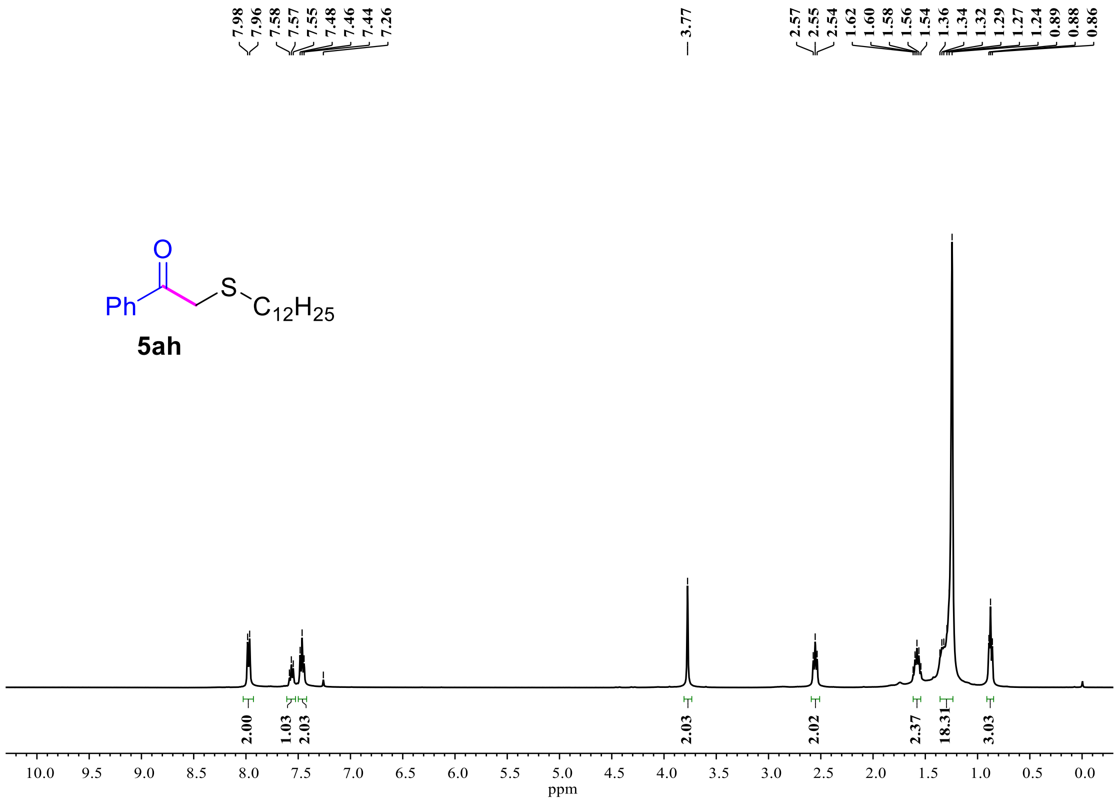

Supplementary Figure 80. ^{1}H NMR of **5ah**.

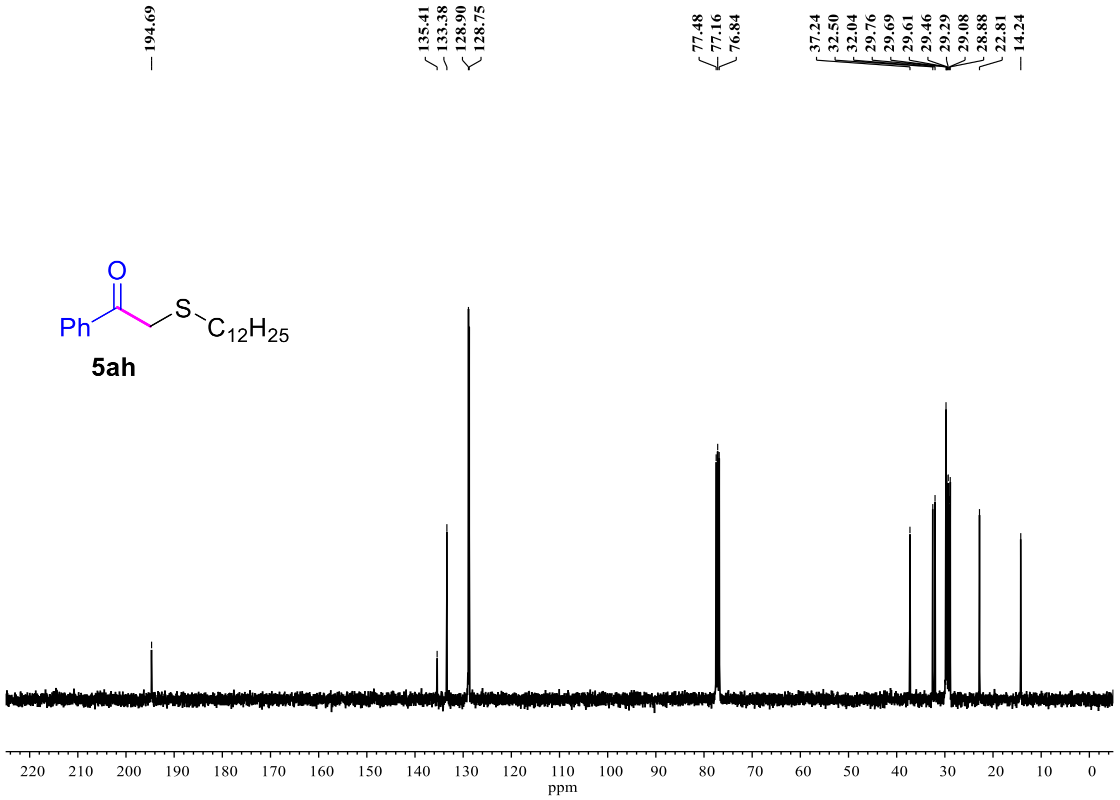

Supplementary Figure 81. ^{13}C NMR of **5ah**.

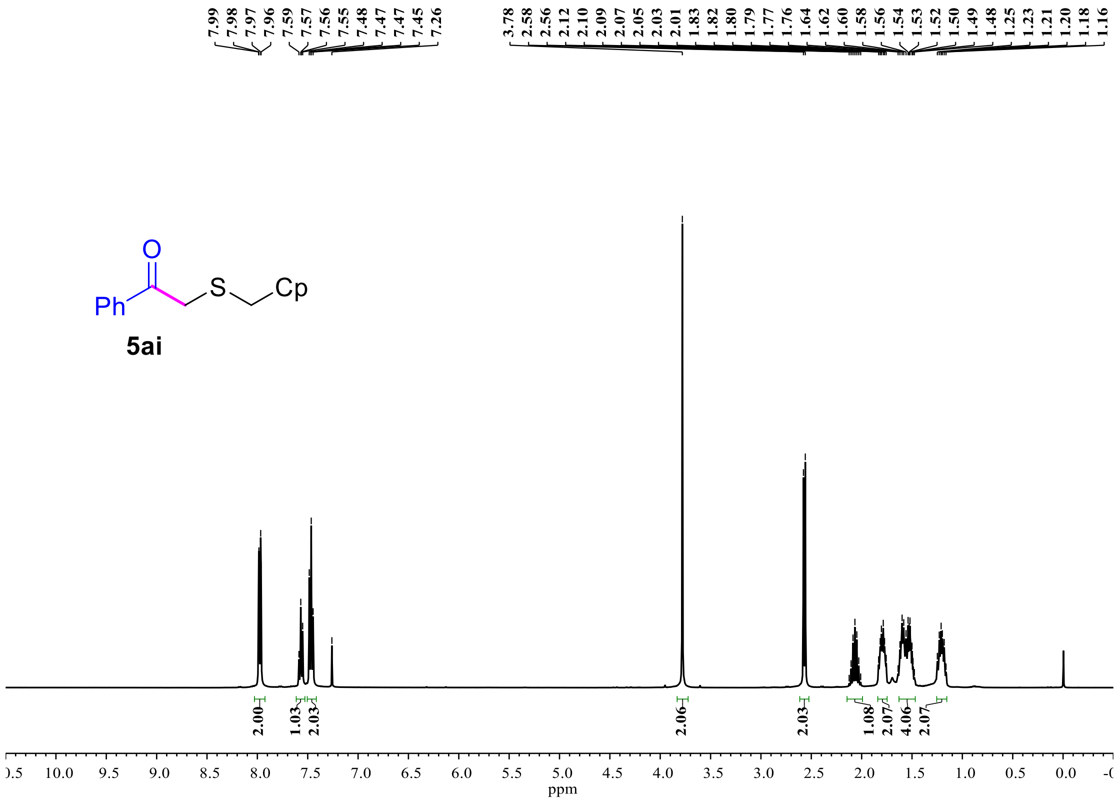

Supplementary Figure 82. ^{1}H NMR of **5ai**.

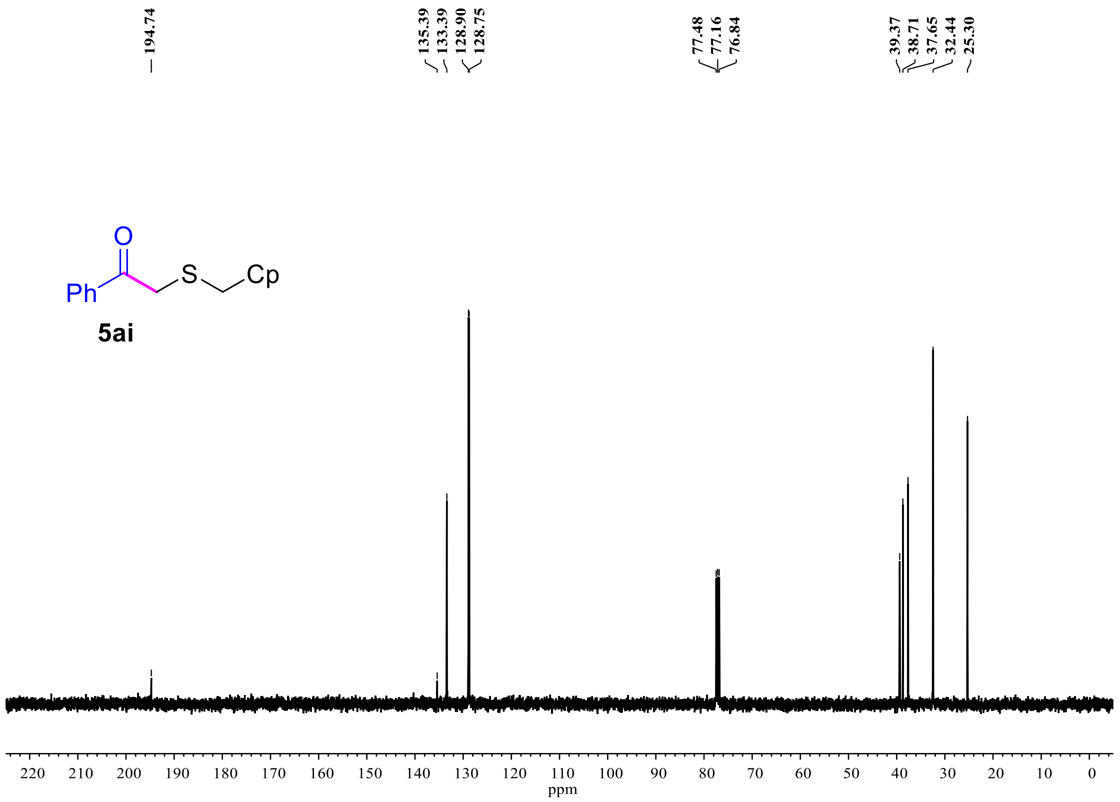

Supplementary Figure 83. ^{13}C NMR of **5ai**.

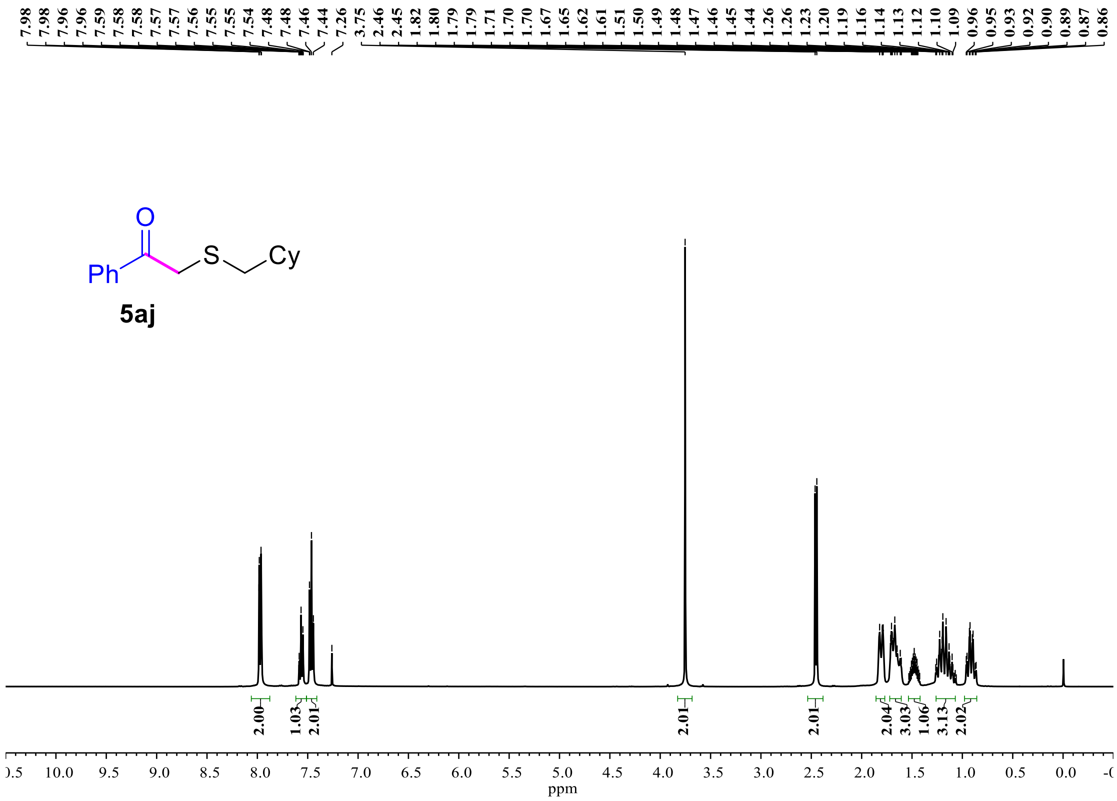

Supplementary Figure 84. ^{1}H NMR of **5aj**.

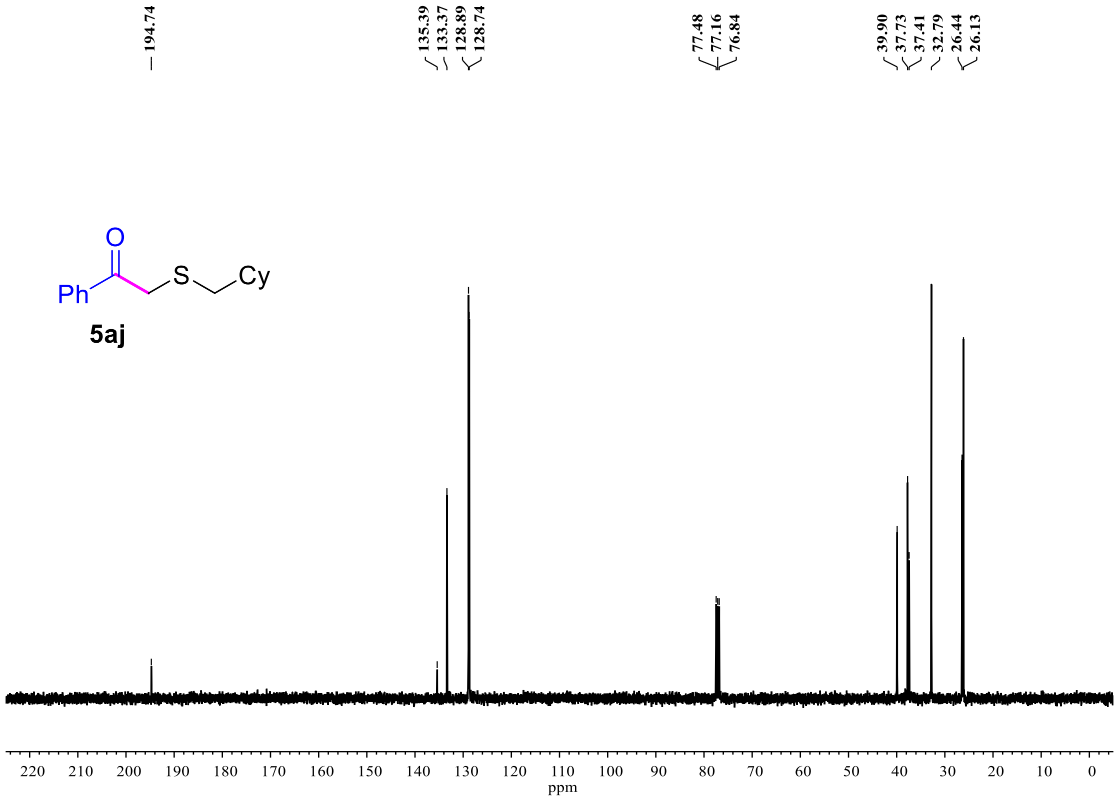

Supplementary Figure 85. ^{13}C NMR of **5aj**.

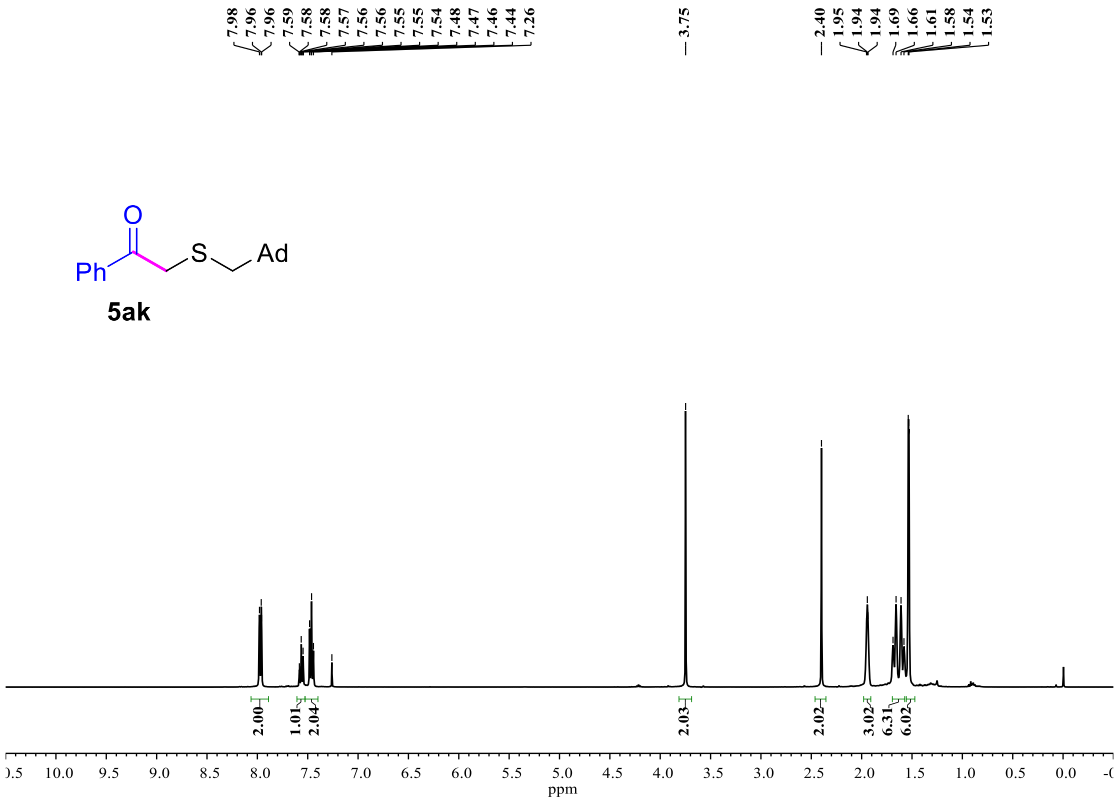

Supplementary Figure 86. ^{1}H NMR of **5ak**.

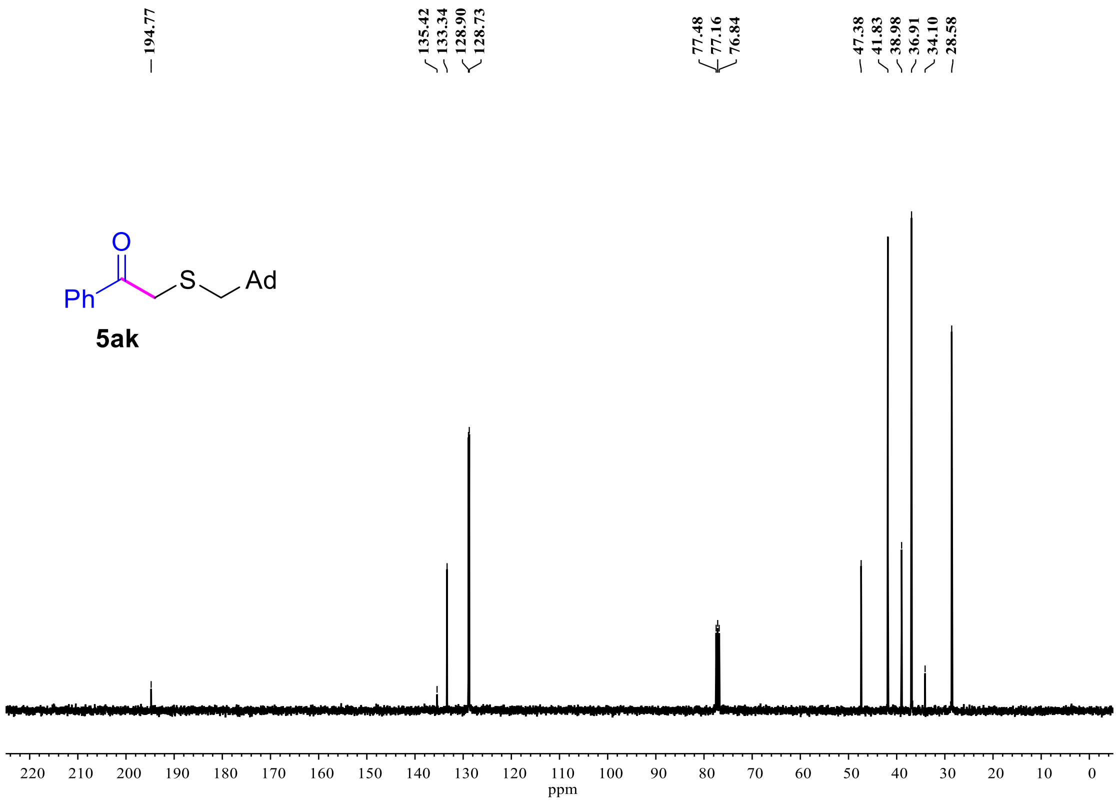

Supplementary Figure 87. ^{13}C NMR of **5ak**.

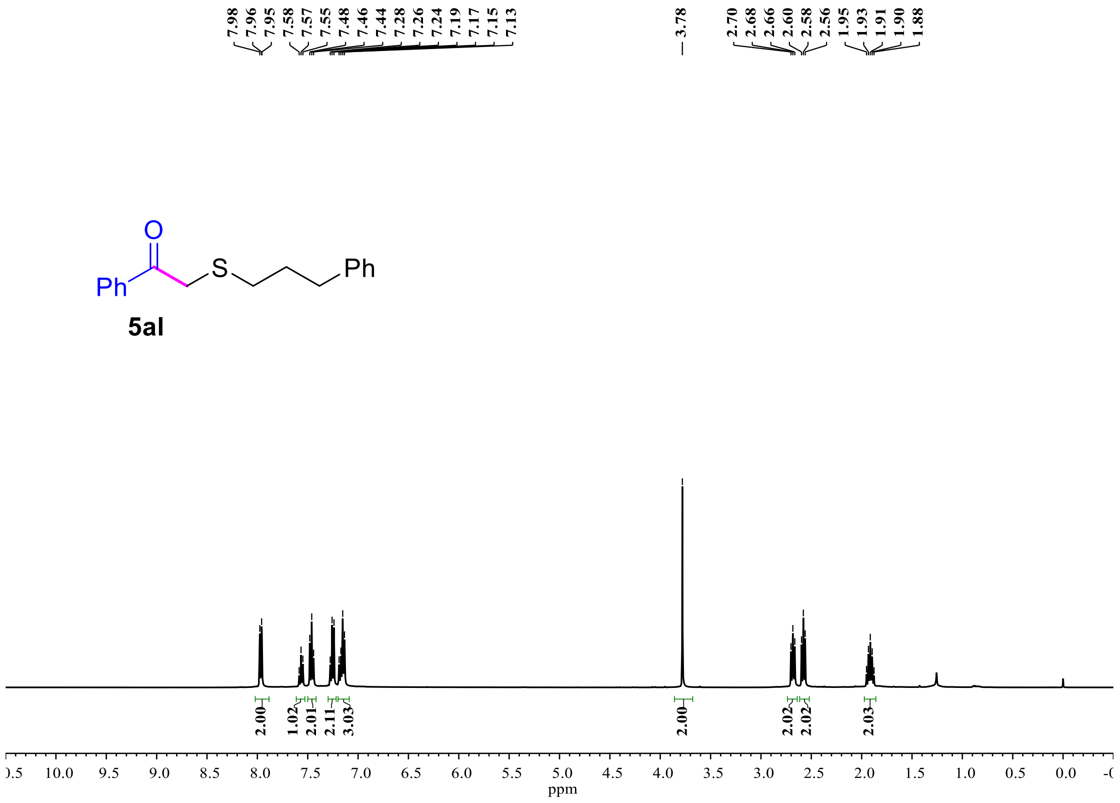

Supplementary Figure 88. ^{1}H NMR of **5al**.

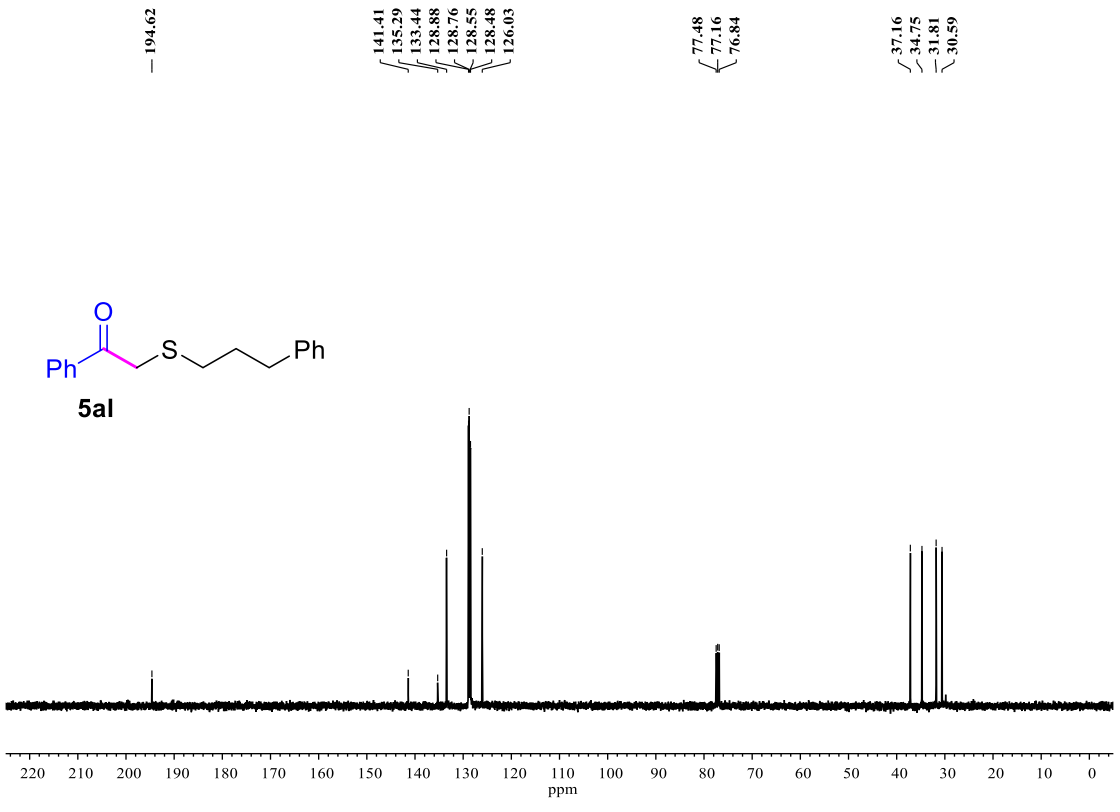

Supplementary Figure 89. ^{13}C NMR of **5al**.

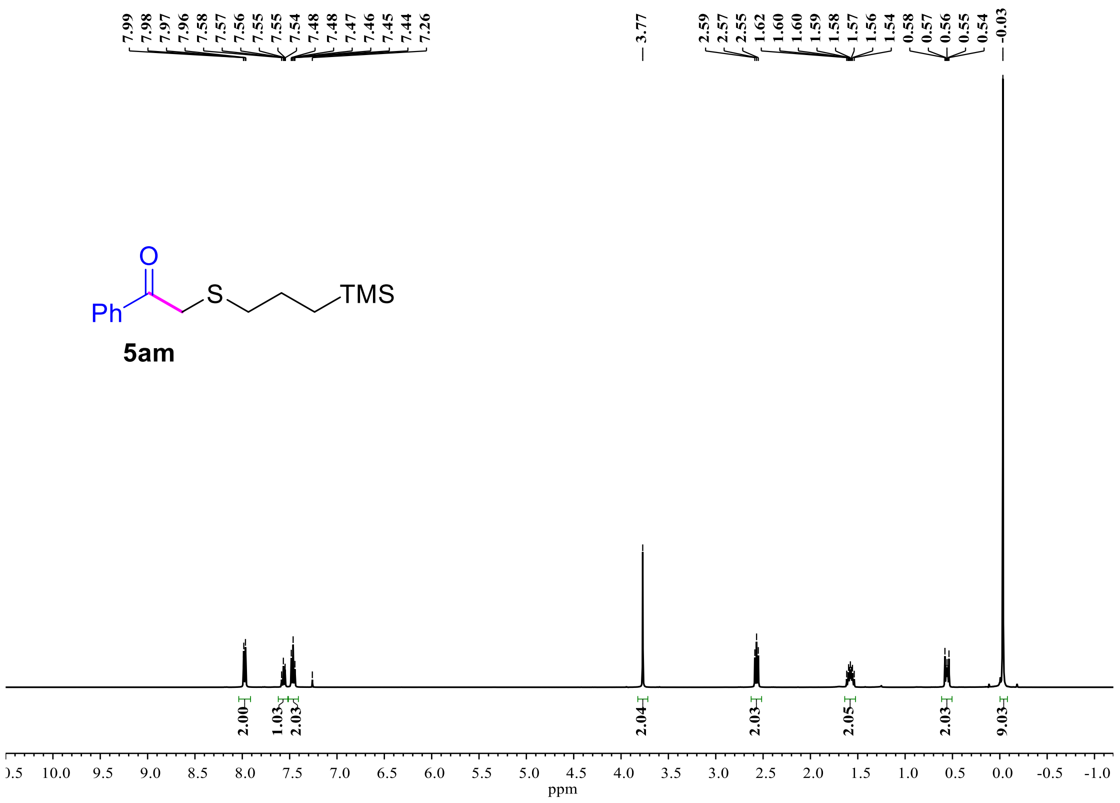

Supplementary Figure 90. ^{1}H NMR of **5am**.

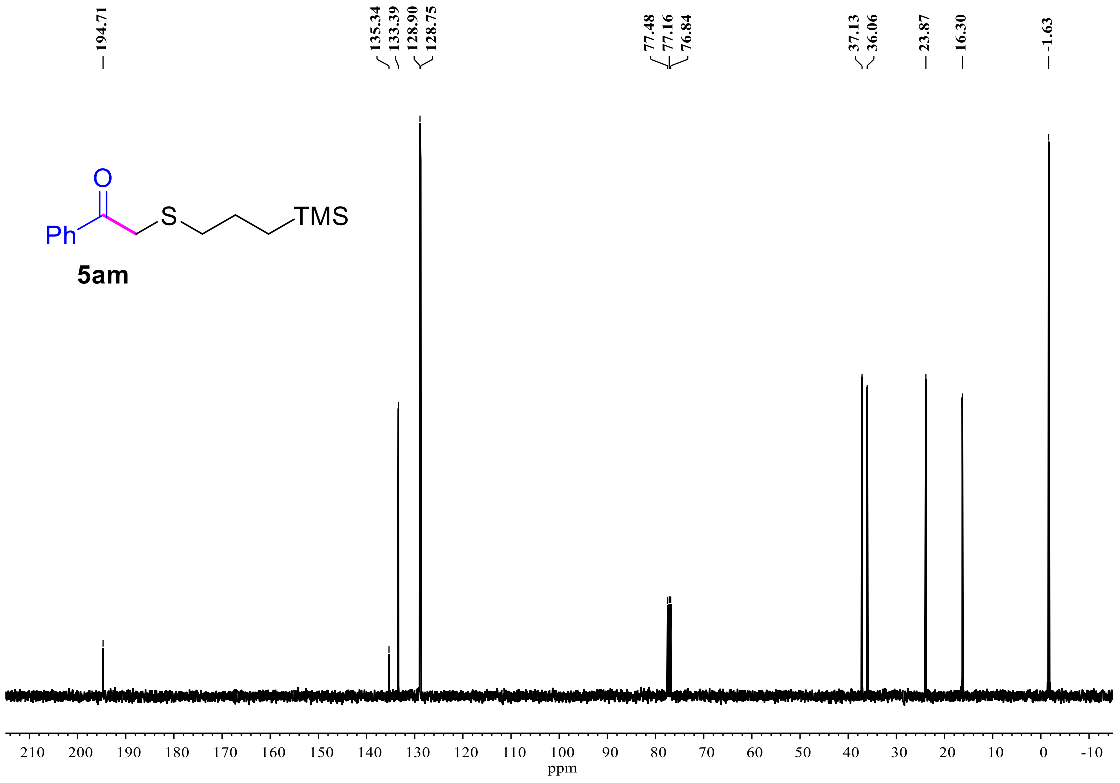

Supplementary Figure 91. ^{13}C NMR of **5am**.

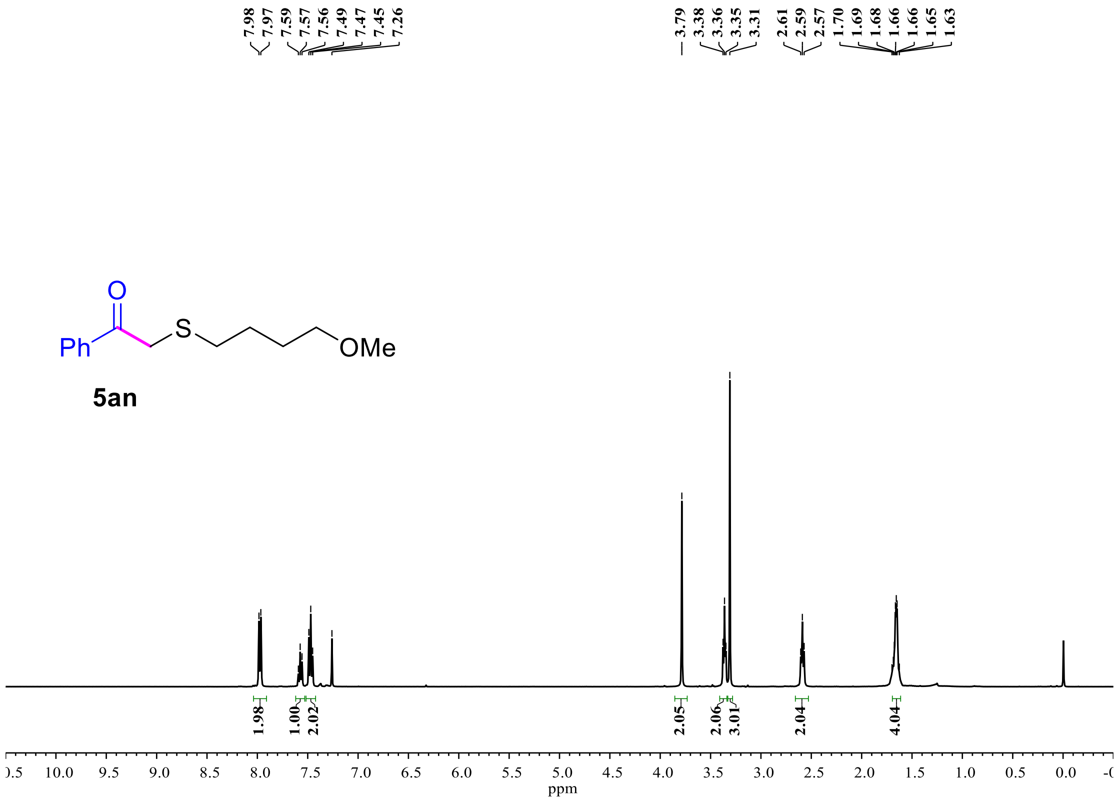

Supplementary Figure 92. ^{1}H NMR of **5an**.

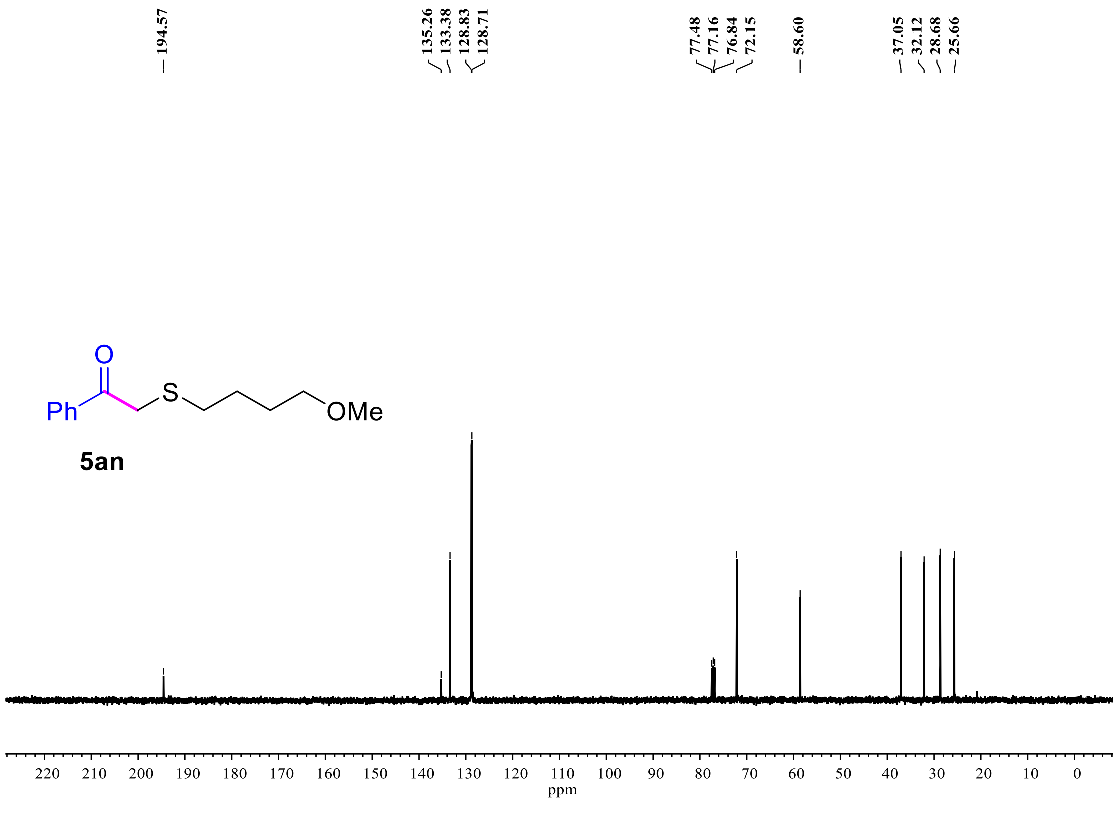

Supplementary Figure 93. ^{13}C NMR of **5an**.

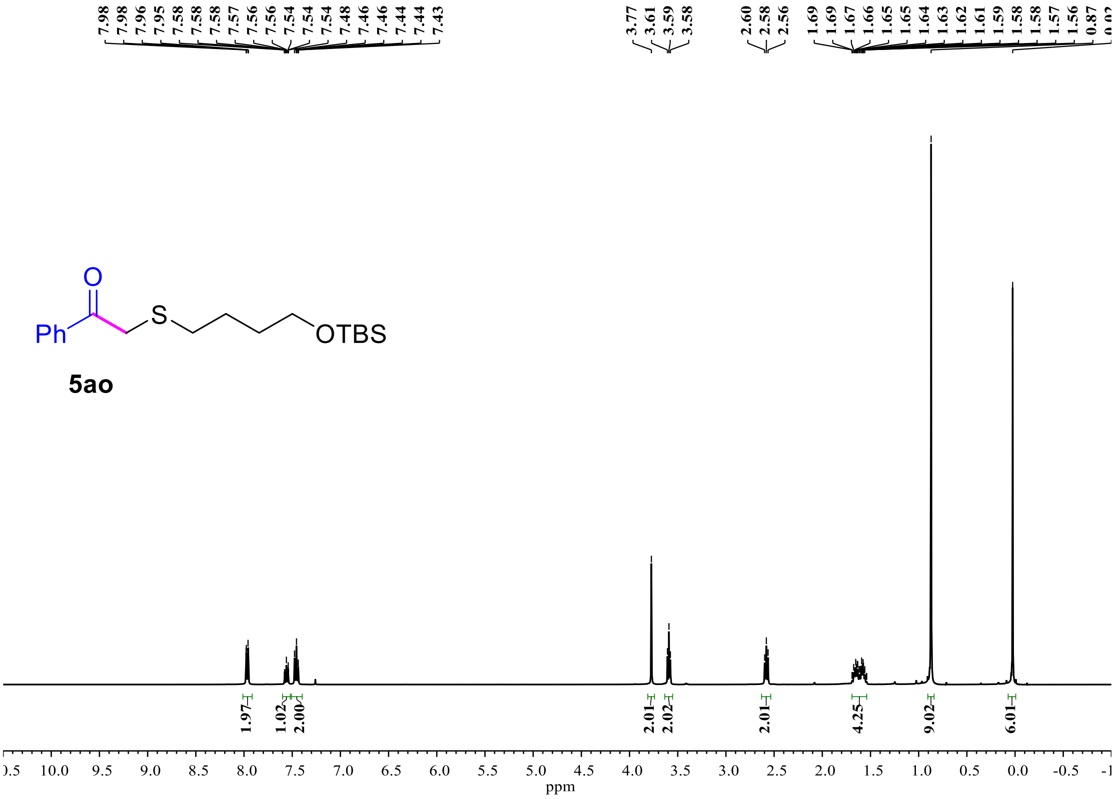

Supplementary Figure 94. ^{1}H NMR of **5ao**.

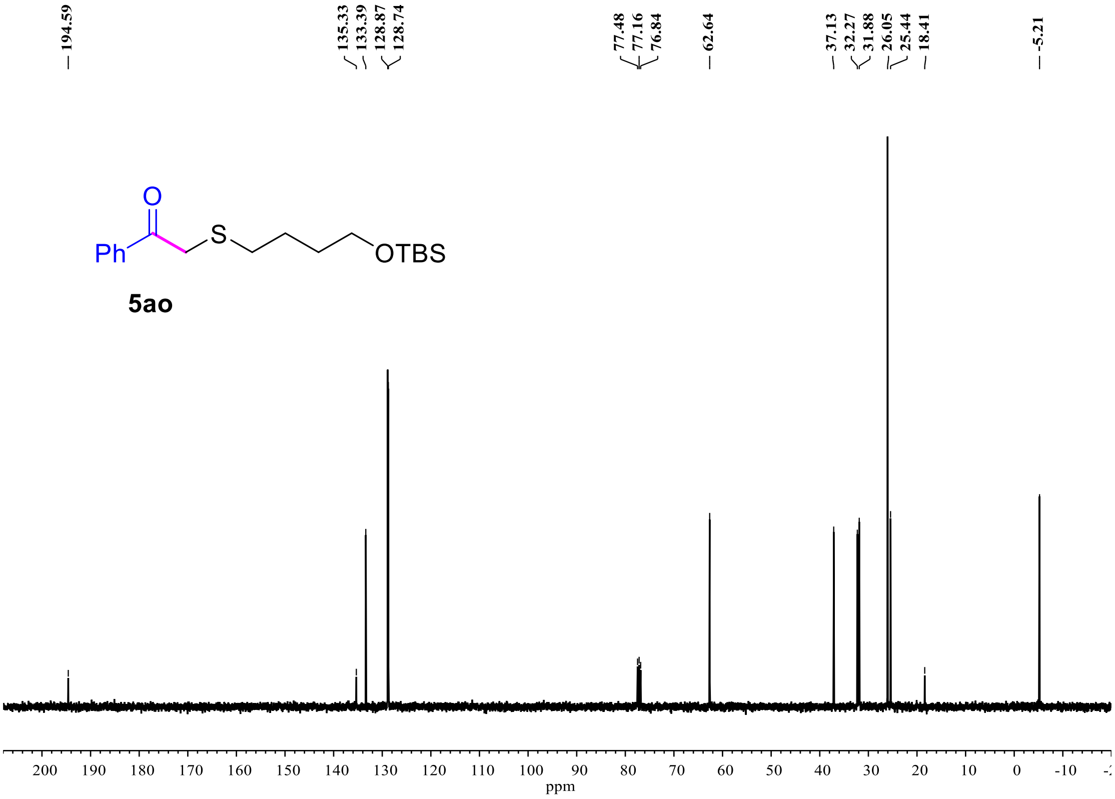

Supplementary Figure 95. ^{13}C NMR of **5ao**.

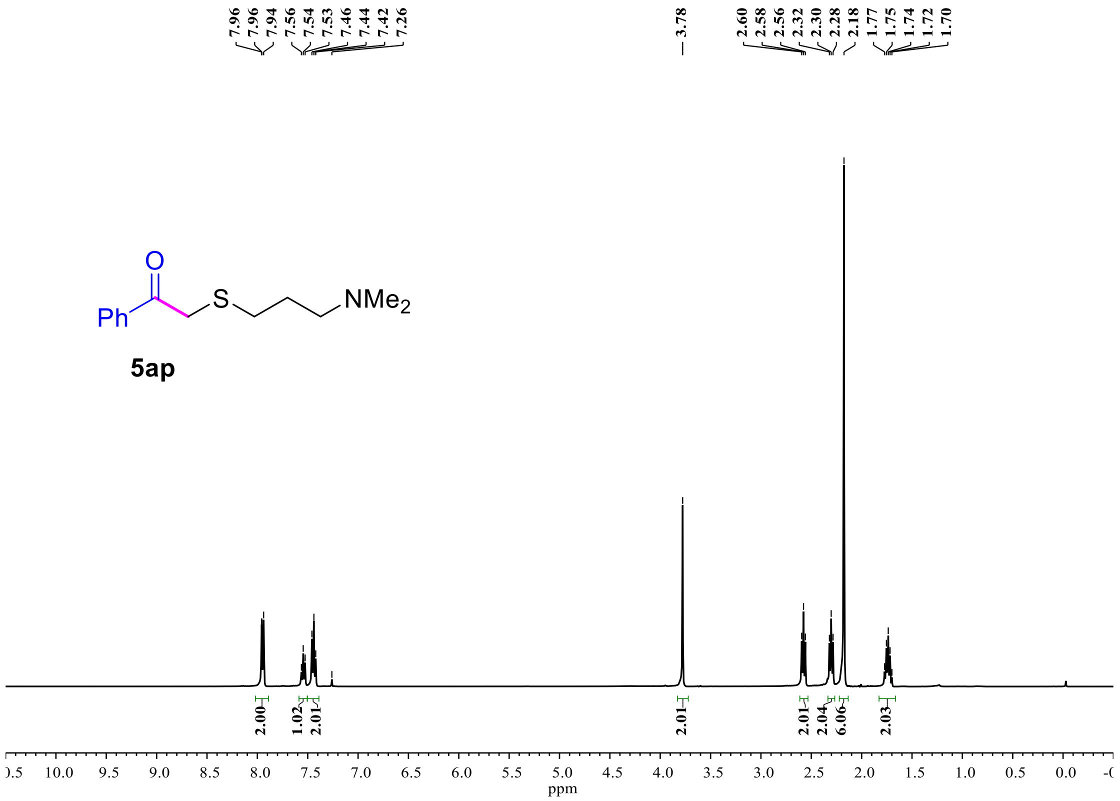

Supplementary Figure 96. ^{1}H NMR of **5ap**.

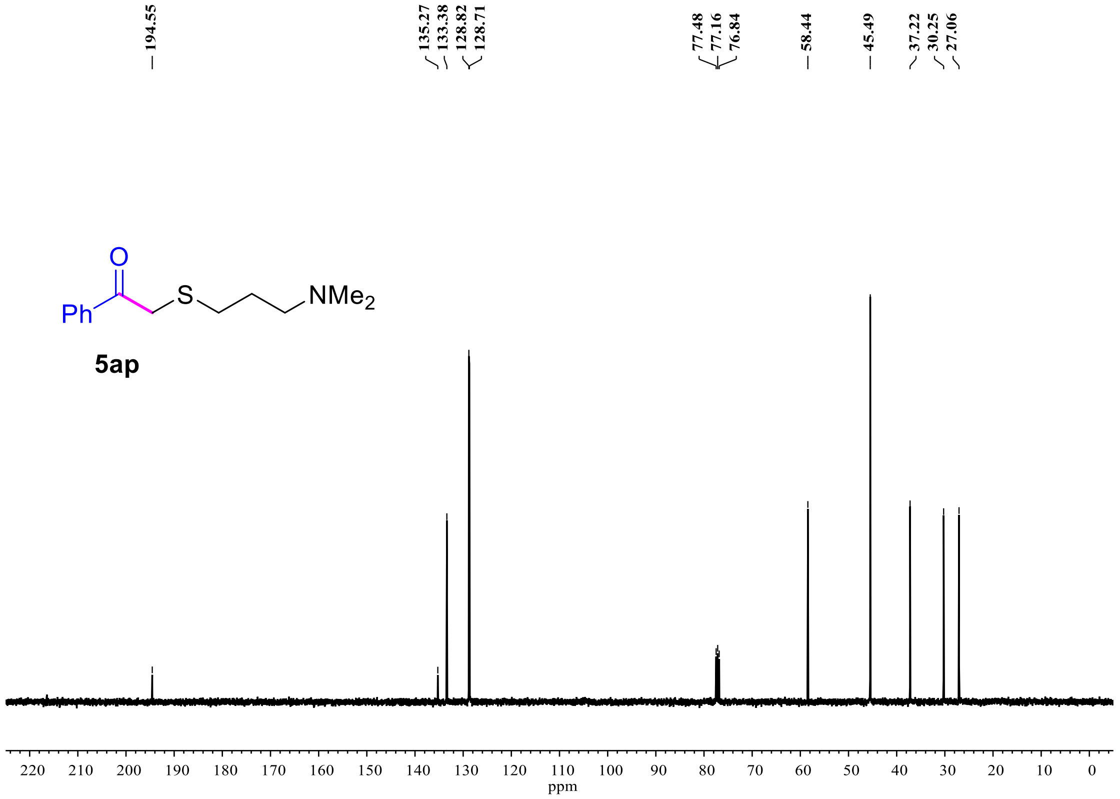

Supplementary Figure 97. ^{13}C NMR of **5ap**.

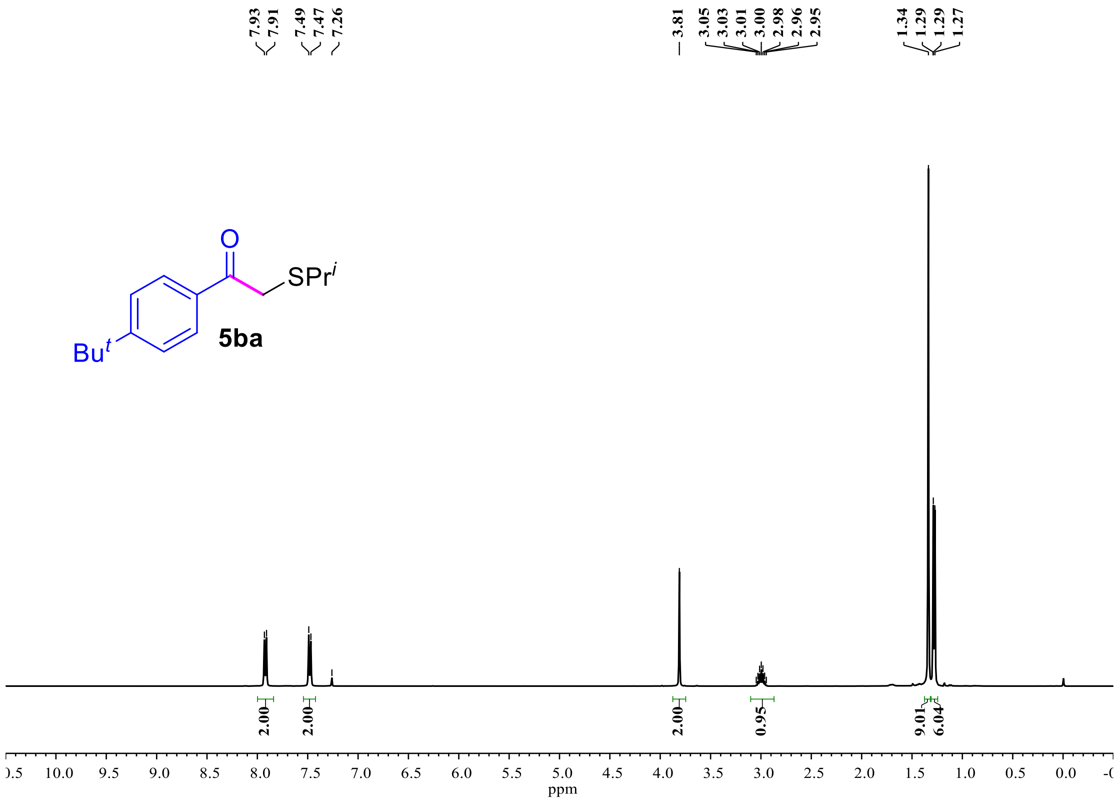

Supplementary Figure 98. ^{1}H NMR of **5ba**.

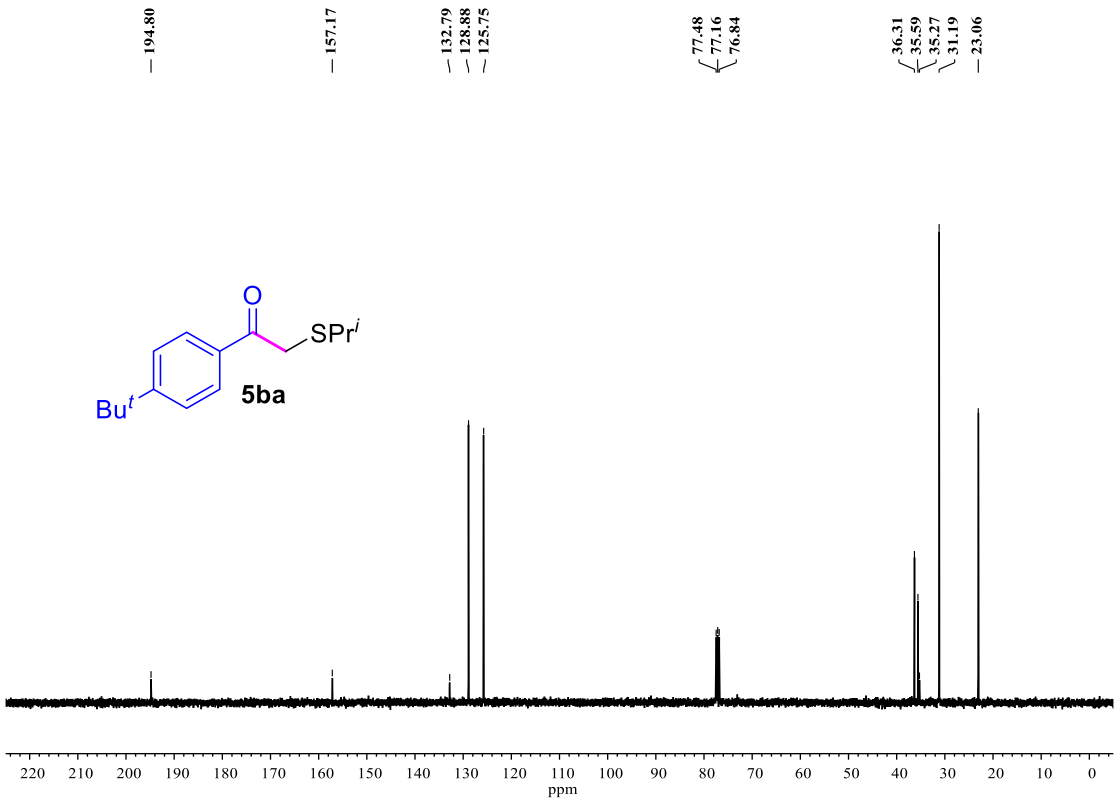

Supplementary Figure 99. ^{13}C NMR of **5ba**.

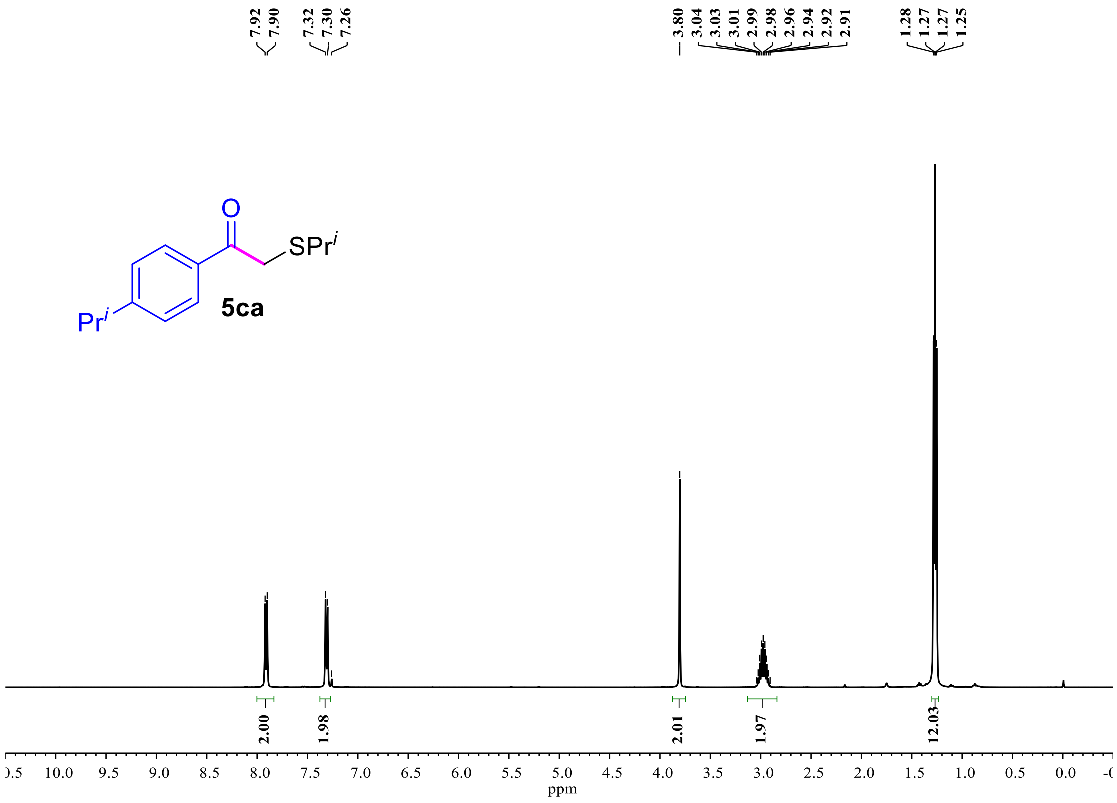

Supplementary Figure 100. ^{1}H NMR of **5ca**.

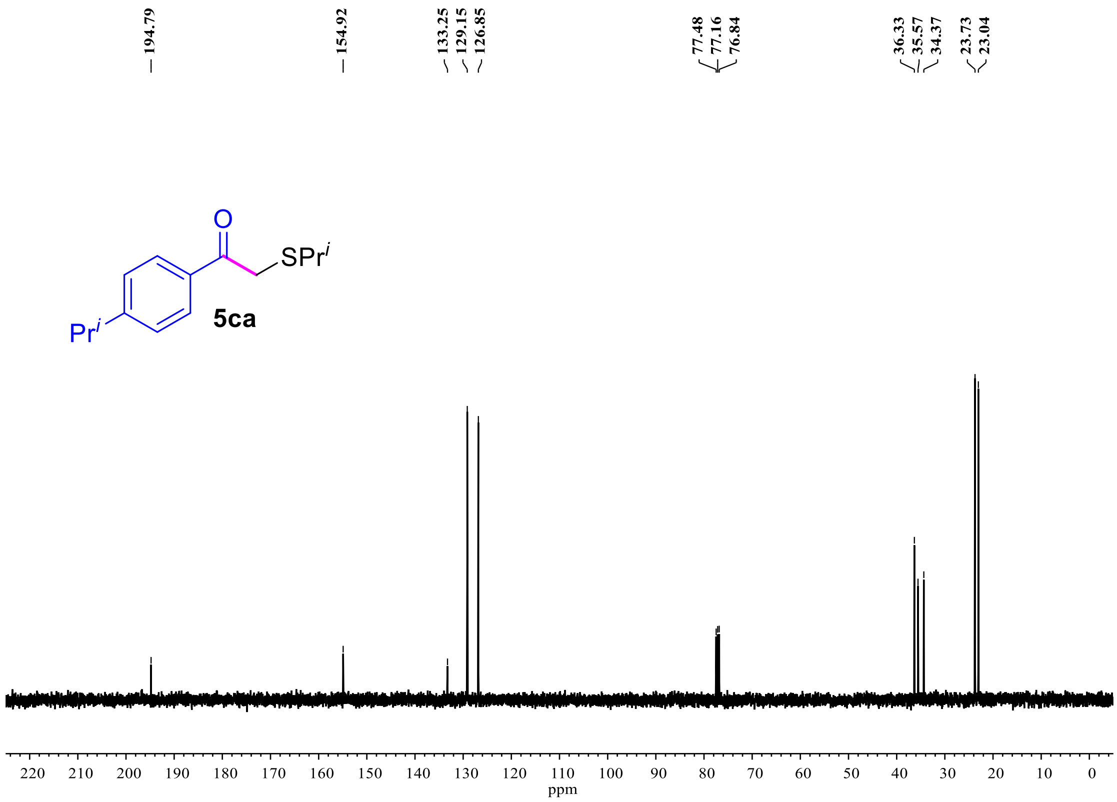

Supplementary Figure 101. ^{13}C NMR of **5ca**.

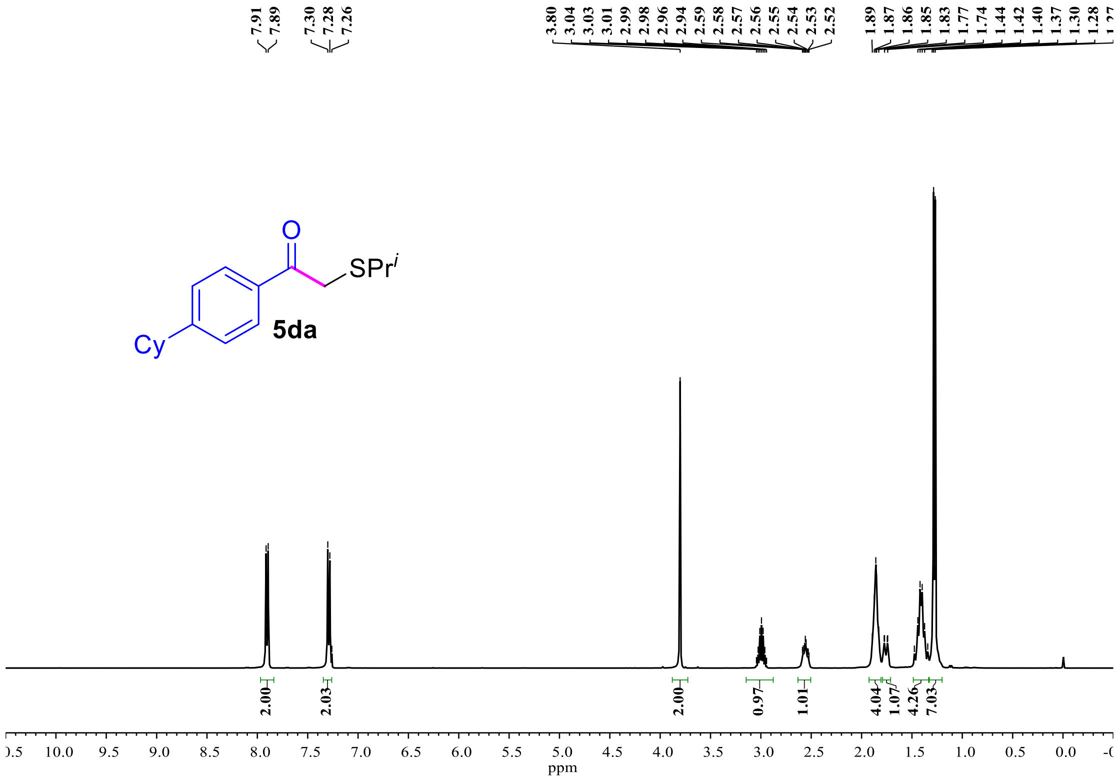

Supplementary Figure 102. ^{1}H NMR of **5da**.

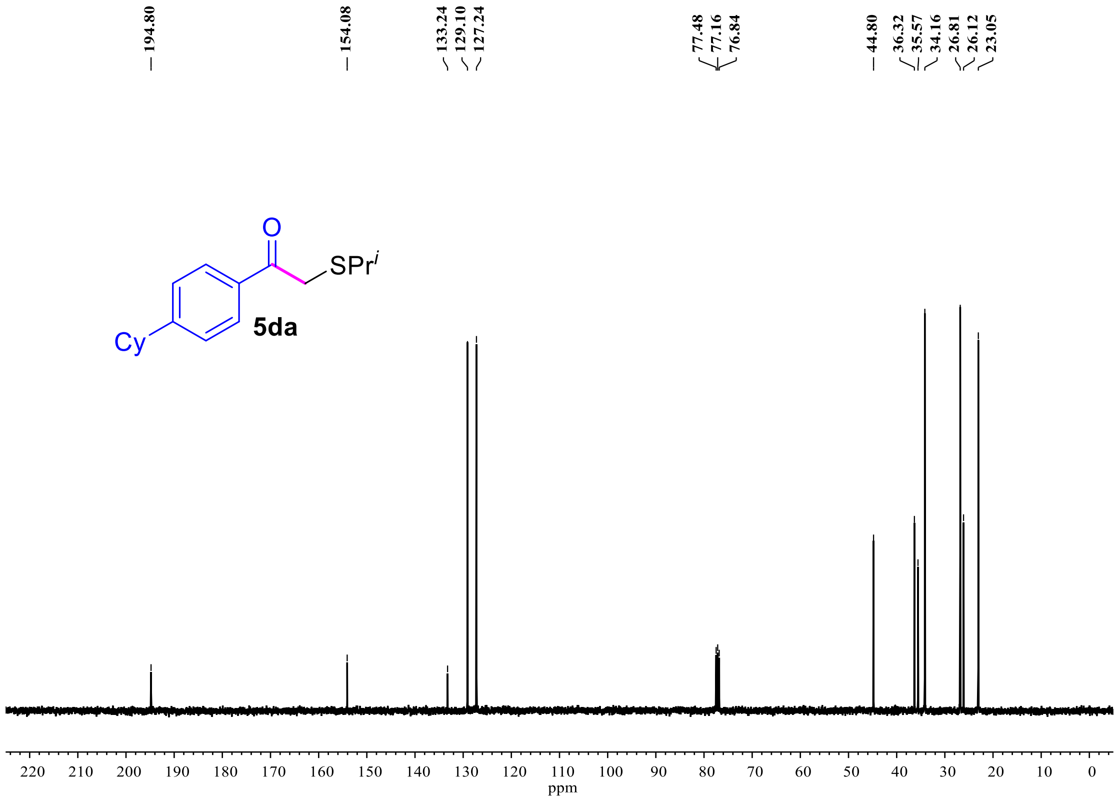

Supplementary Figure 103. ^{13}C NMR of **5da**.

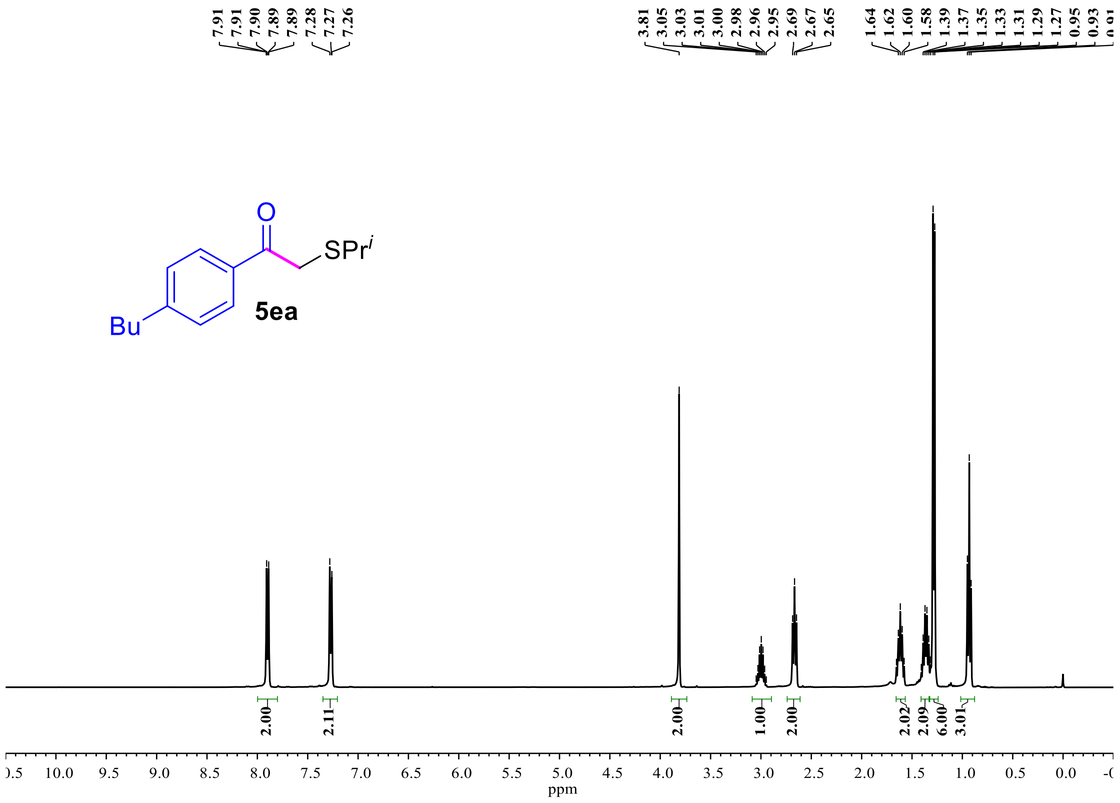

Supplementary Figure 104. ^{1}H NMR of **5ea**.

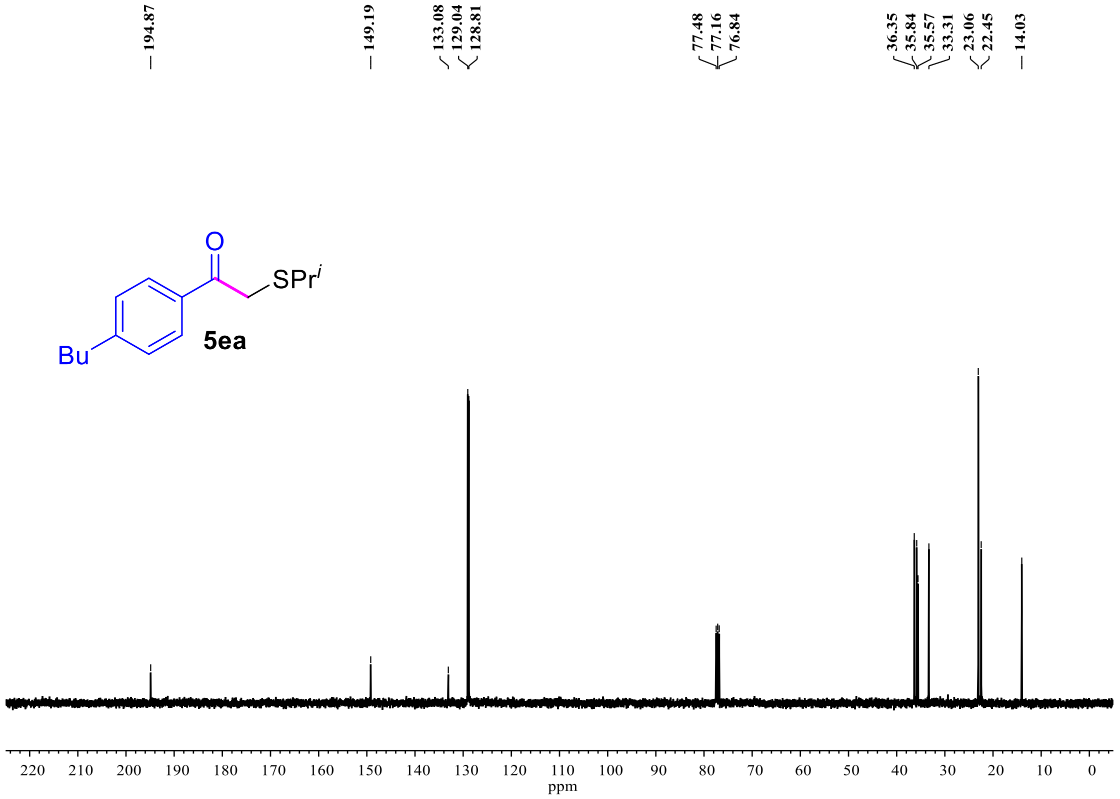

Supplementary Figure 105. ^{13}C NMR of **5ea**.

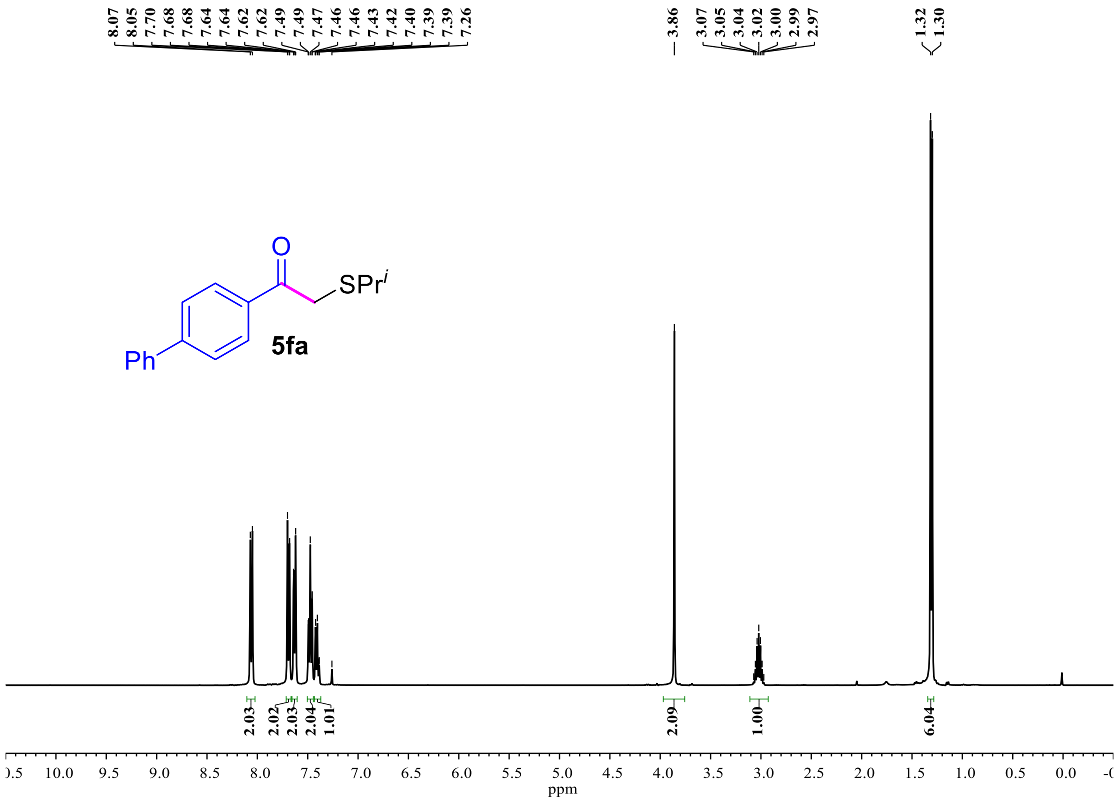

Supplementary Figure 106. ^{1}H NMR of **5fa**.

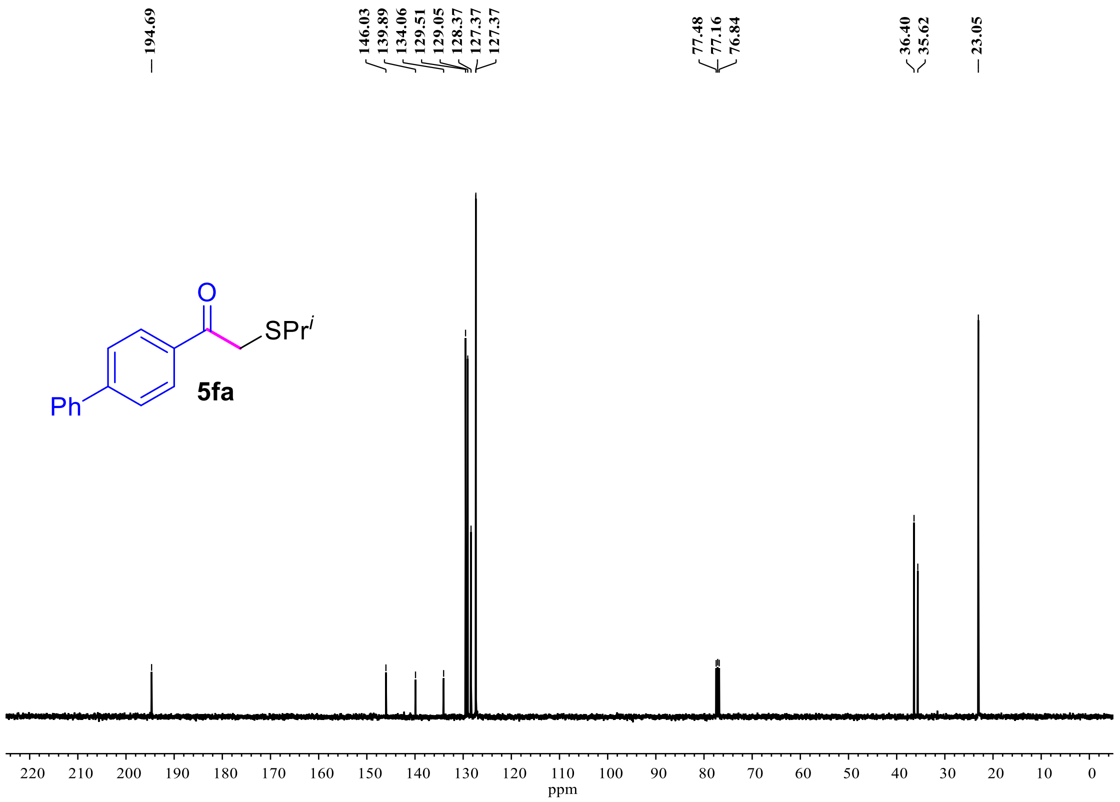

Supplementary Figure 107. ^{13}C NMR of **5fa**.

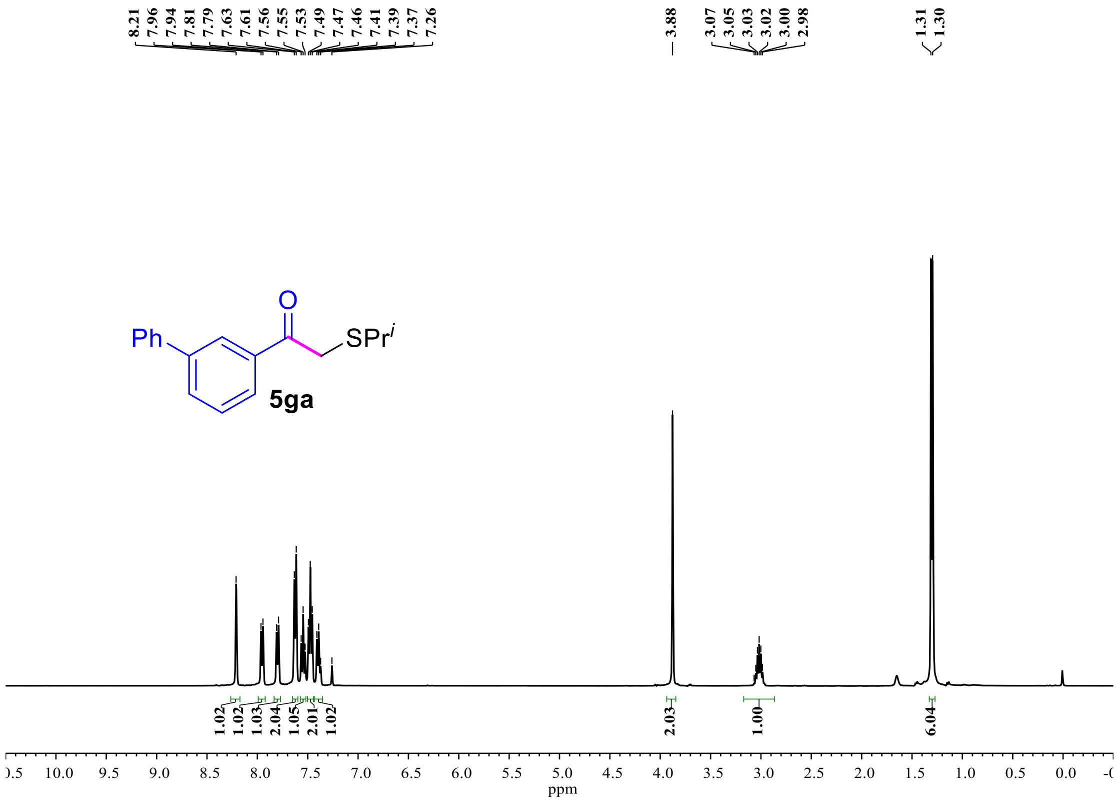

Supplementary Figure 108. ^{1}H NMR of **5ga**.

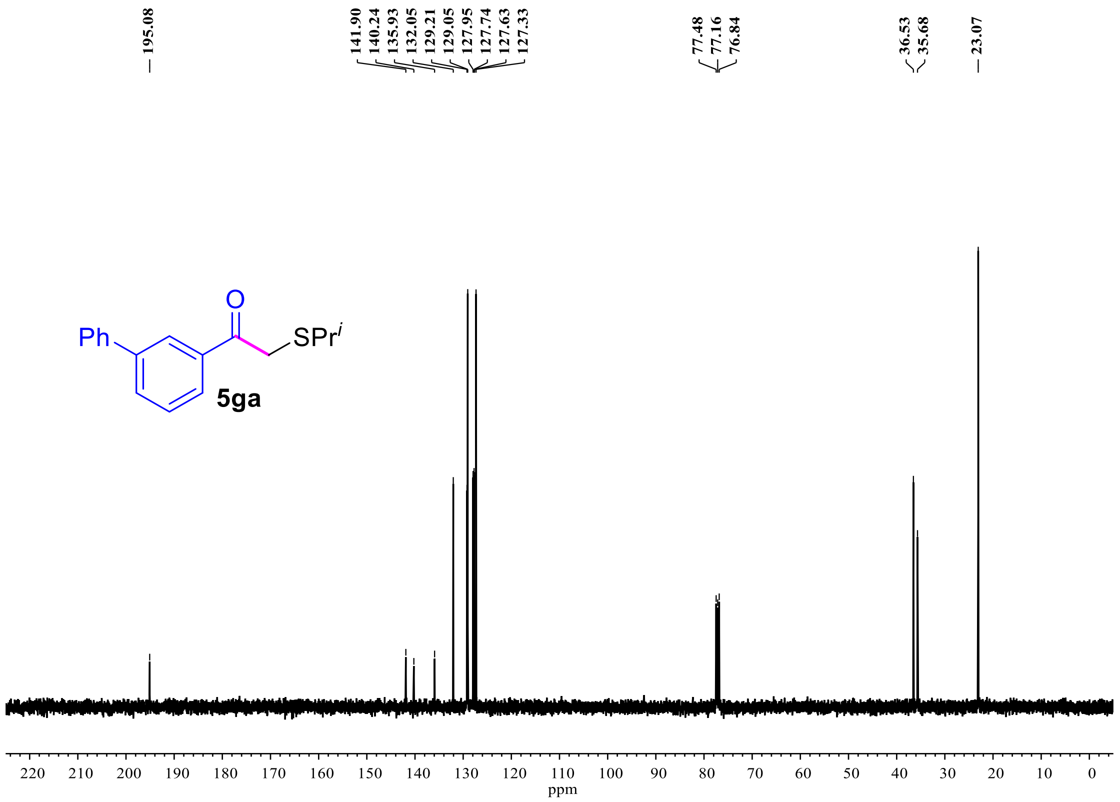

Supplementary Figure 109. ^{13}C NMR of **5ga**.

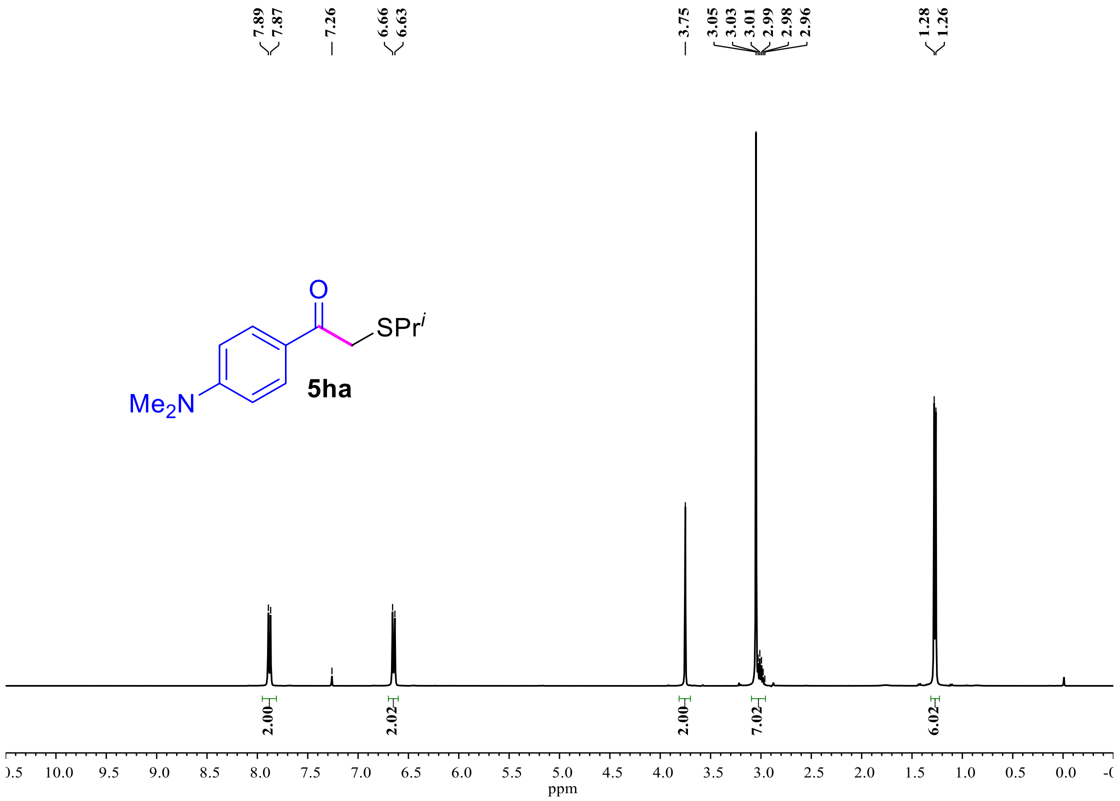

Supplementary Figure 110. ^{1}H NMR of **5ha**.

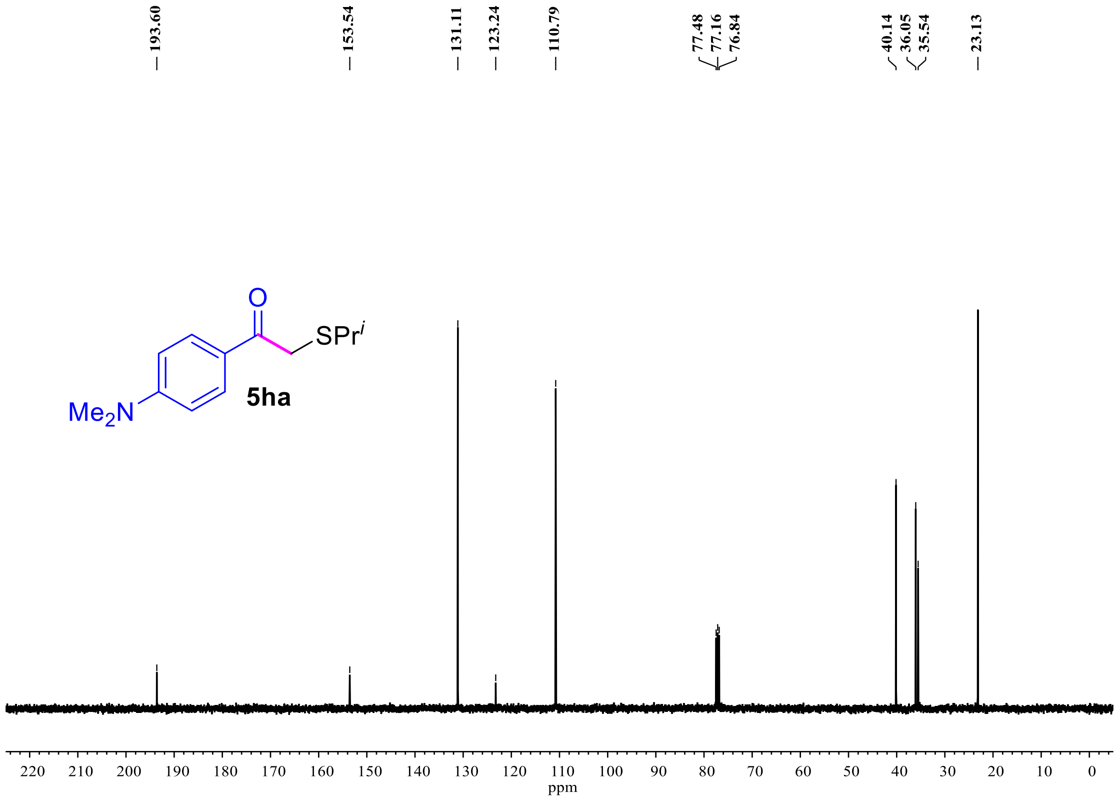

Supplementary Figure 111. ^{13}C NMR of **5ha**.

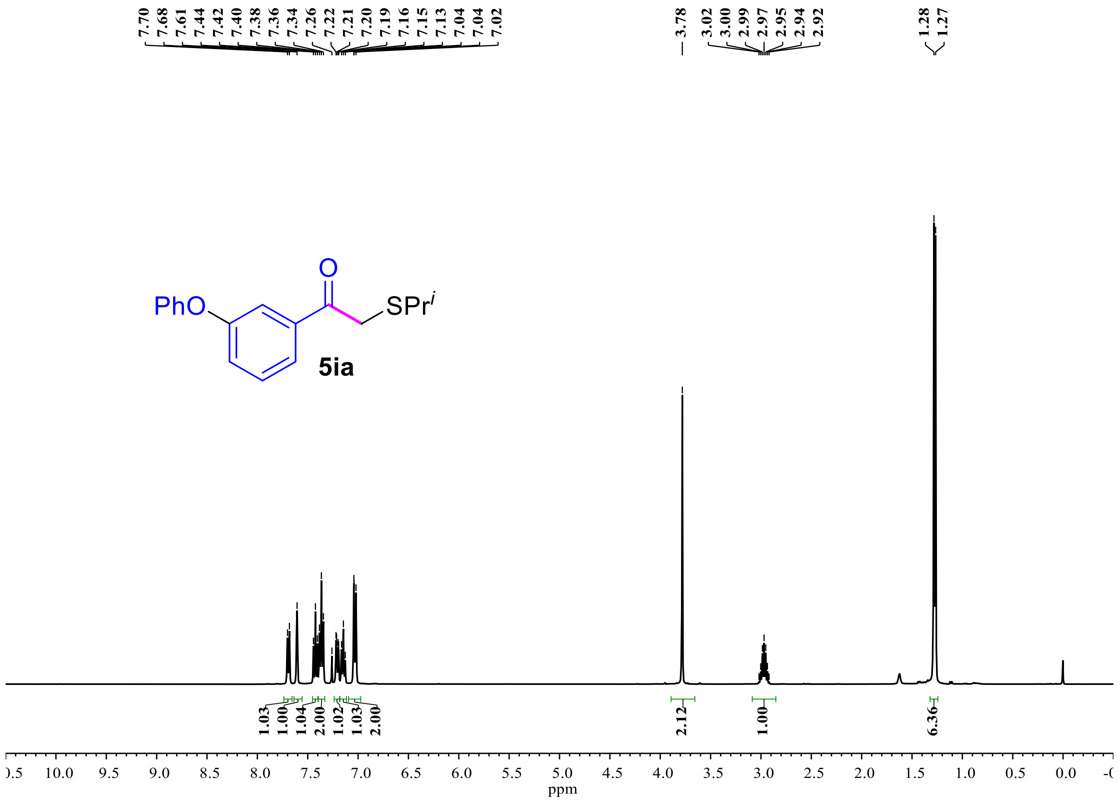

Supplementary Figure 112. ^{1}H NMR of **5ia**.

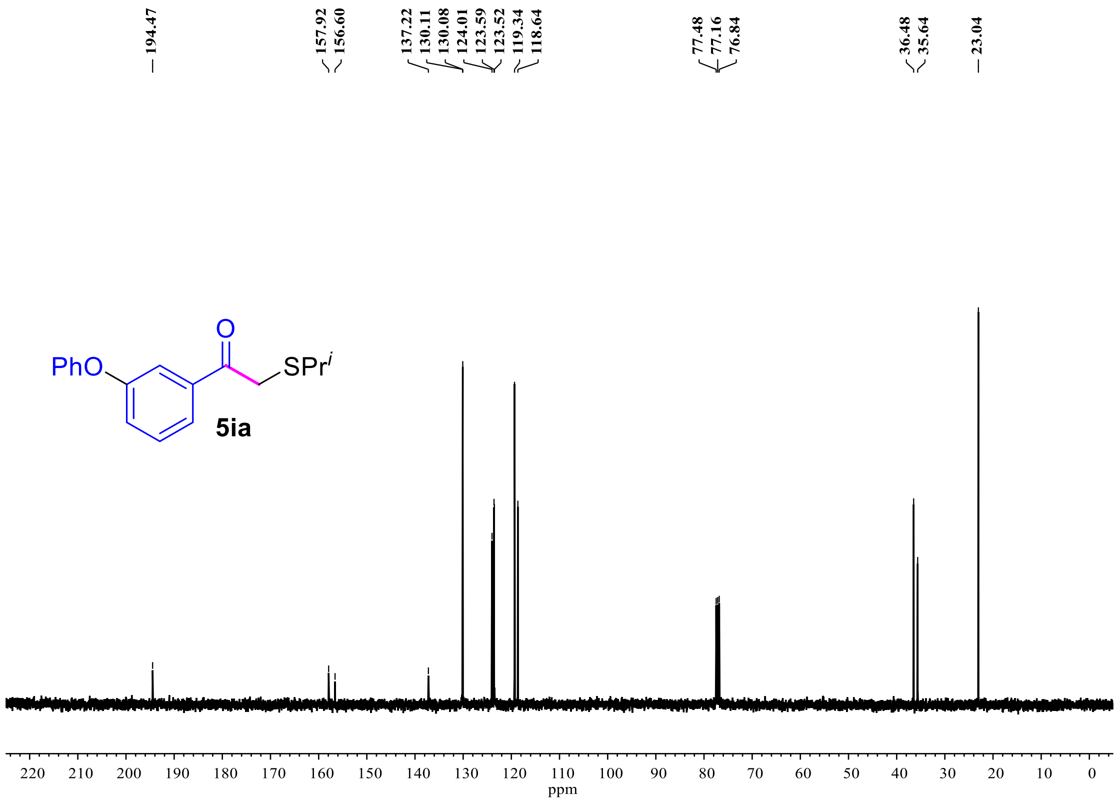

Supplementary Figure 113. ^{13}C NMR of **5ia**.

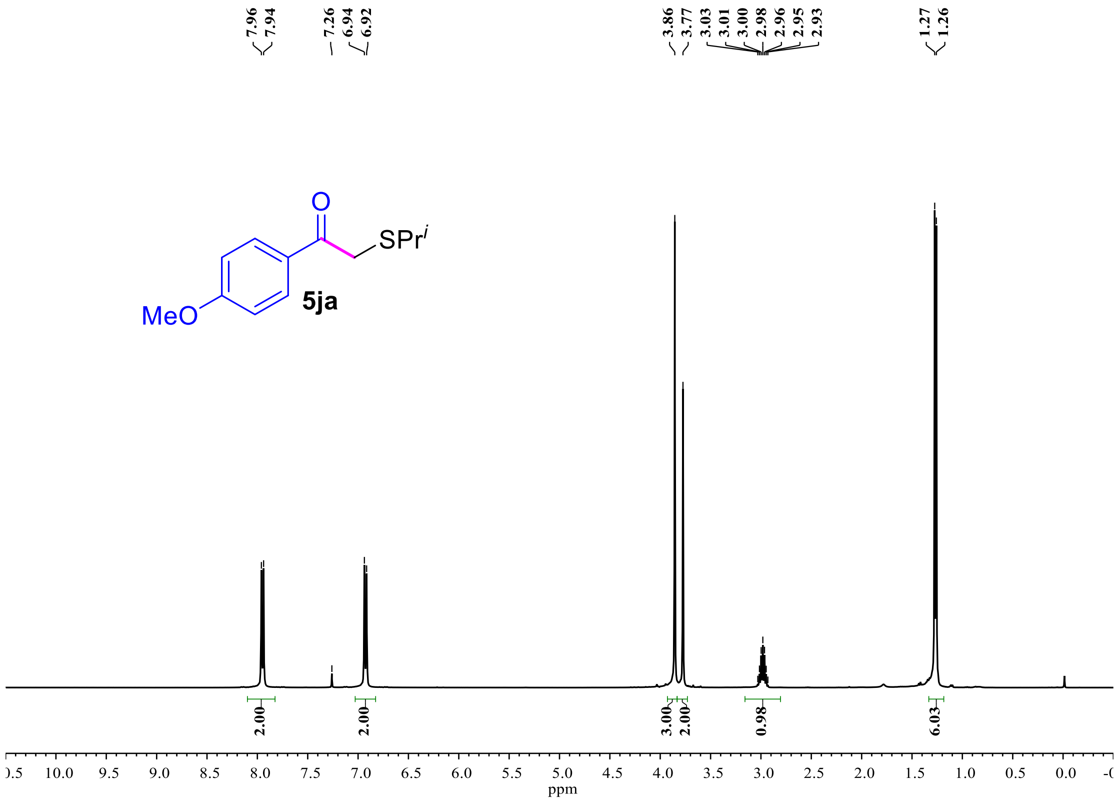

Supplementary Figure 114. ^{1}H NMR of **5ja**.

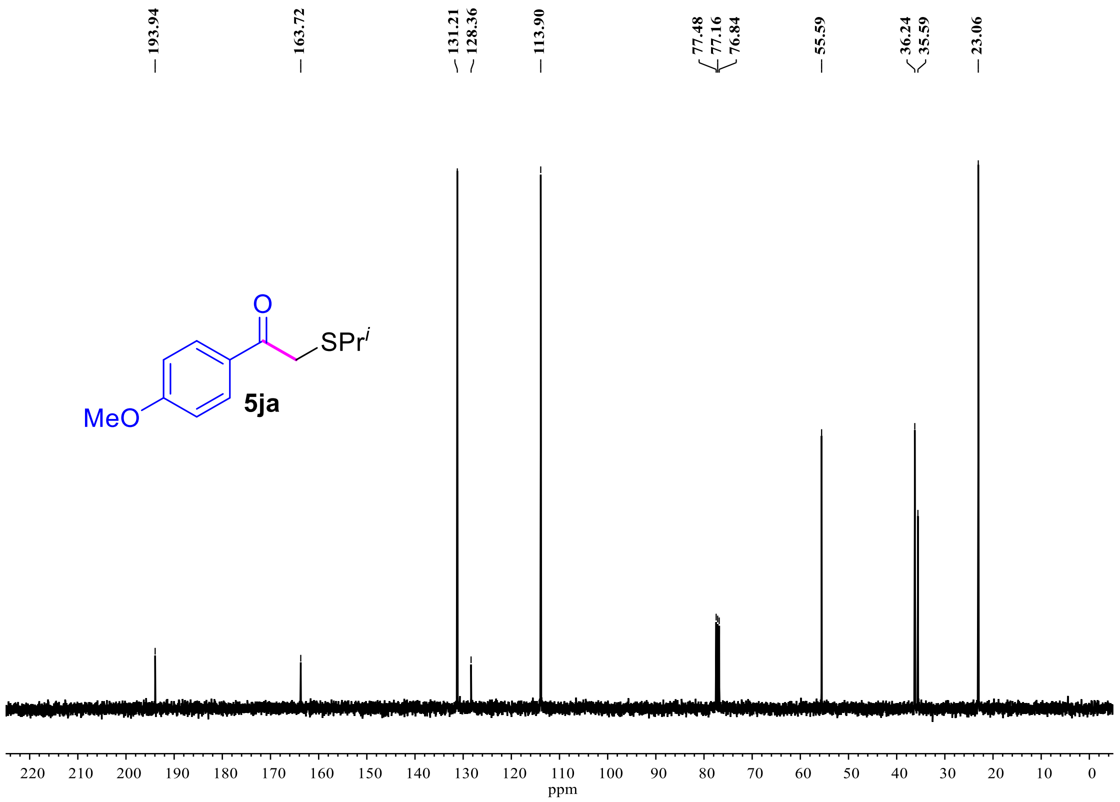

Supplementary Figure 115. ^{13}C NMR of **5ja**.

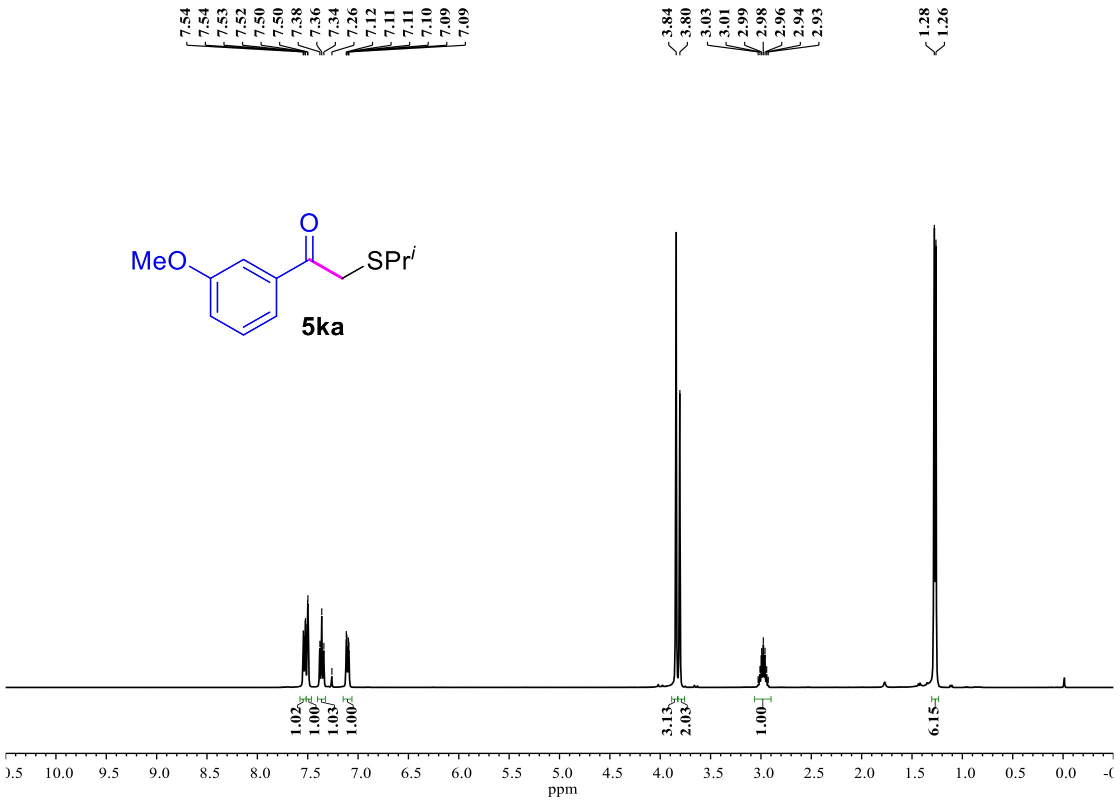

Supplementary Figure 116. ^{1}H NMR of **5ka**.

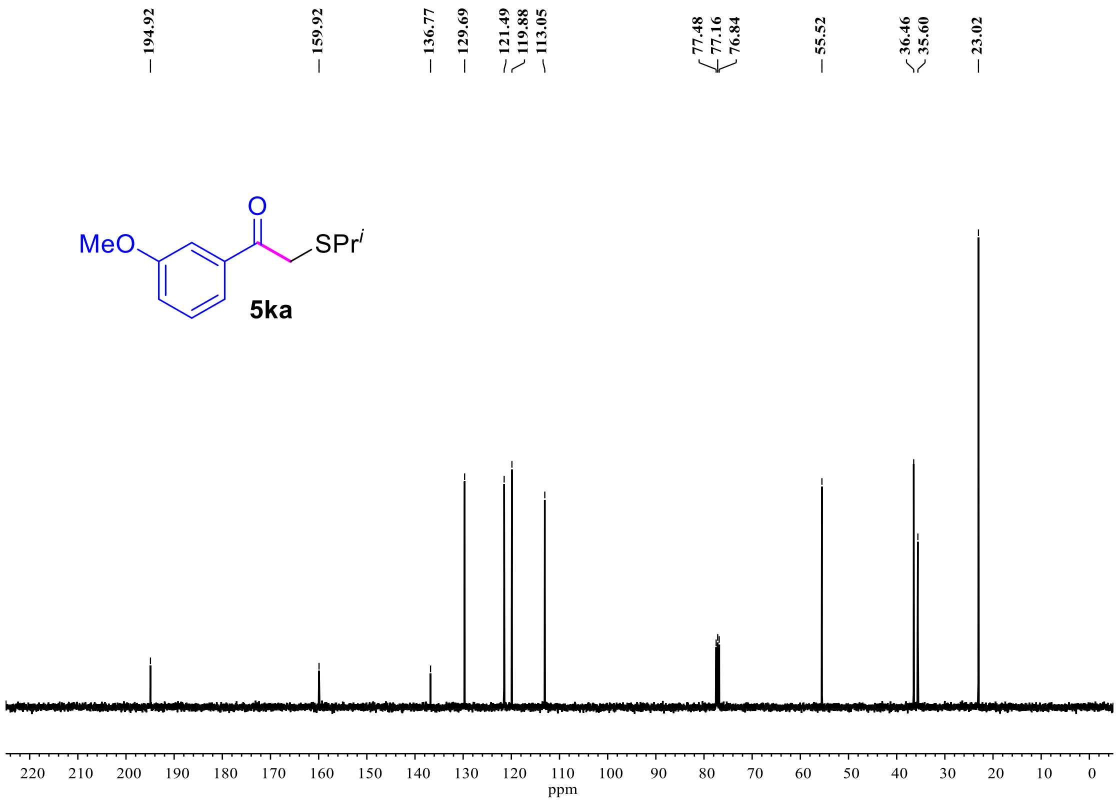

Supplementary Figure 117. ^{13}C NMR of **5ka**.

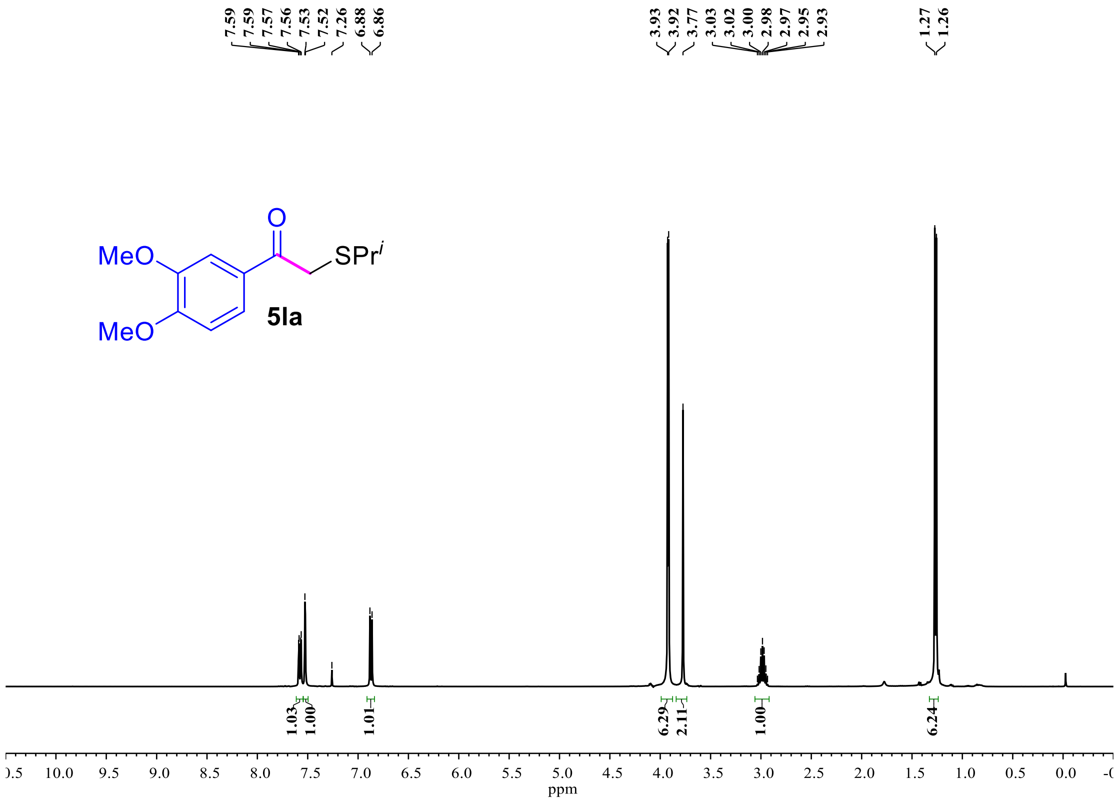

Supplementary Figure 118. ^{1}H NMR of **5la**.

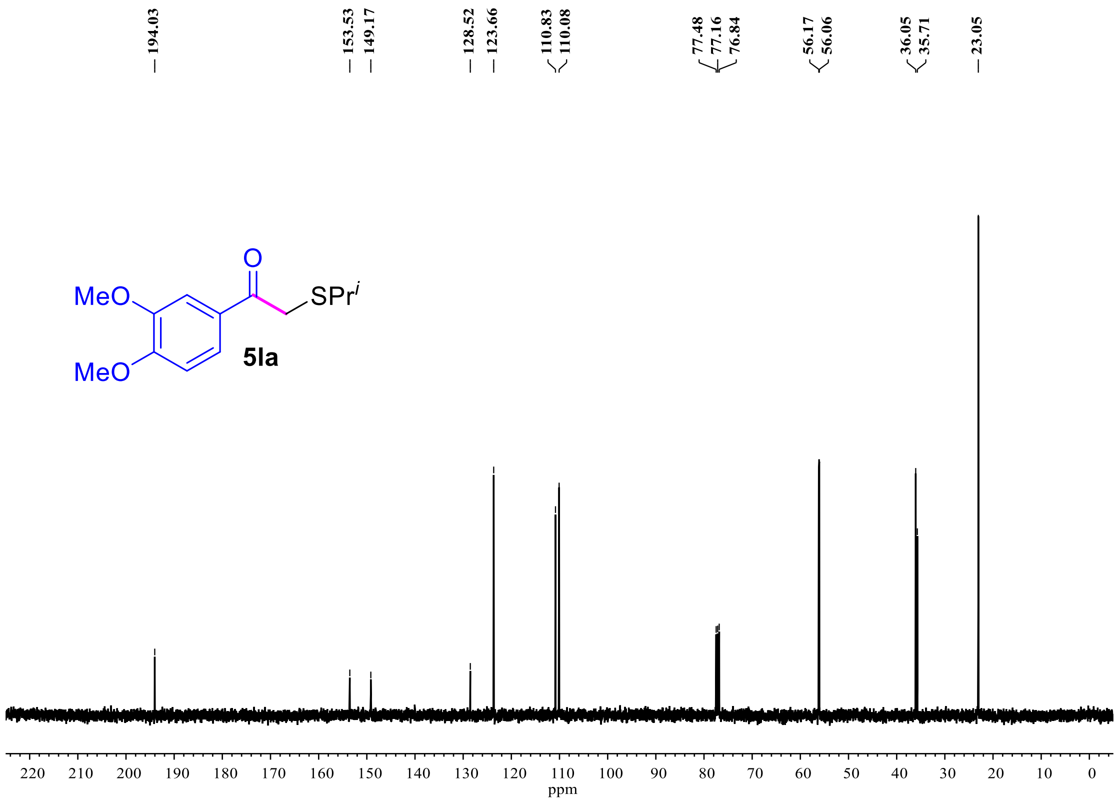

Supplementary Figure 119. ^{13}C NMR of **5la**.

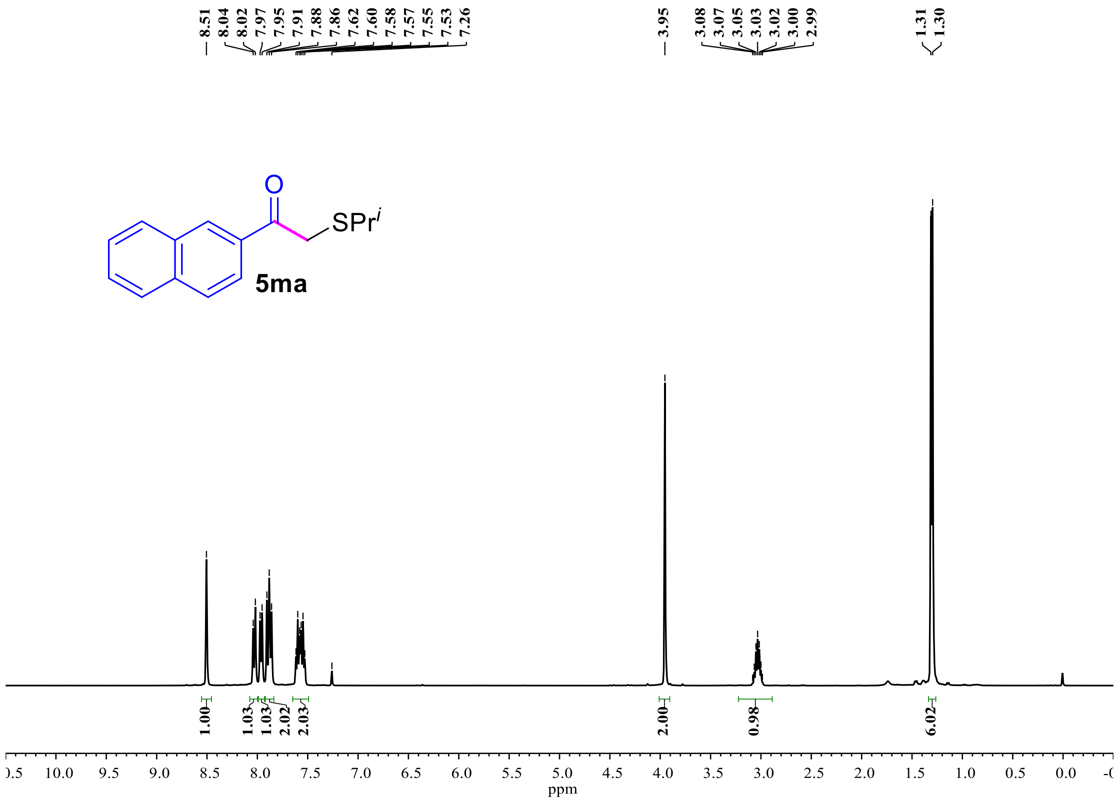

Supplementary Figure 120. ^{1}H NMR of **5ma**.

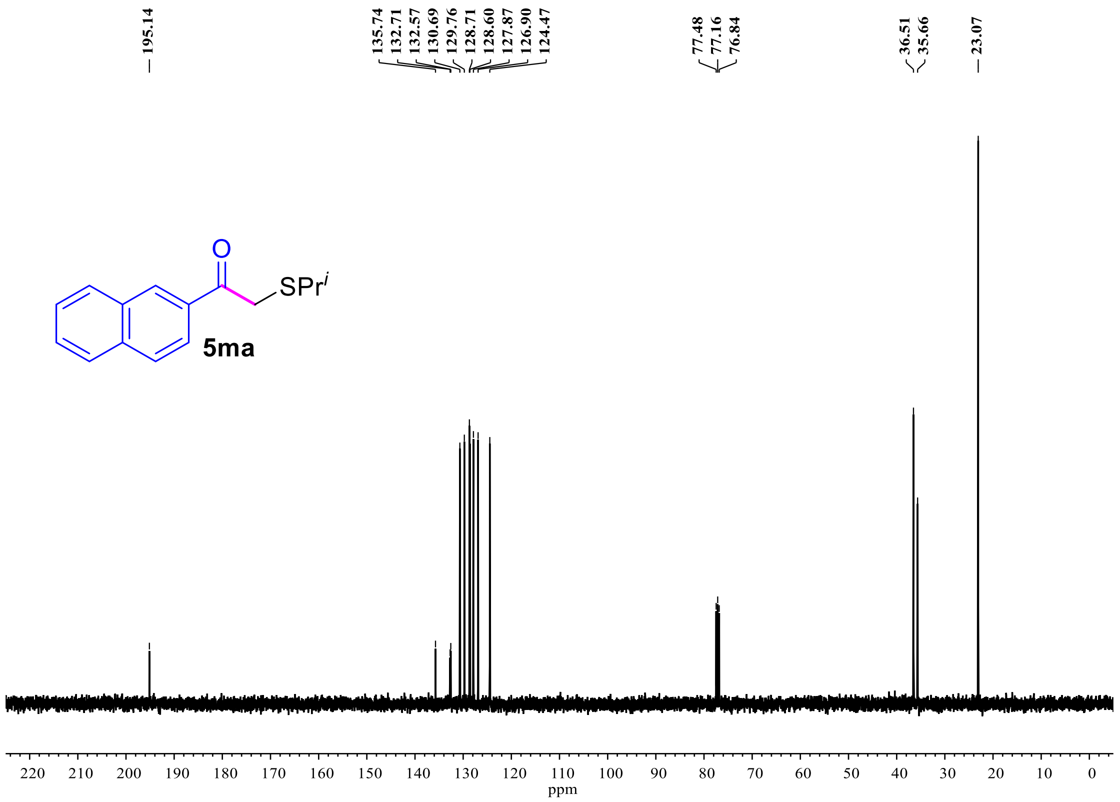

Supplementary Figure 121. ^{13}C NMR of **5ma**.

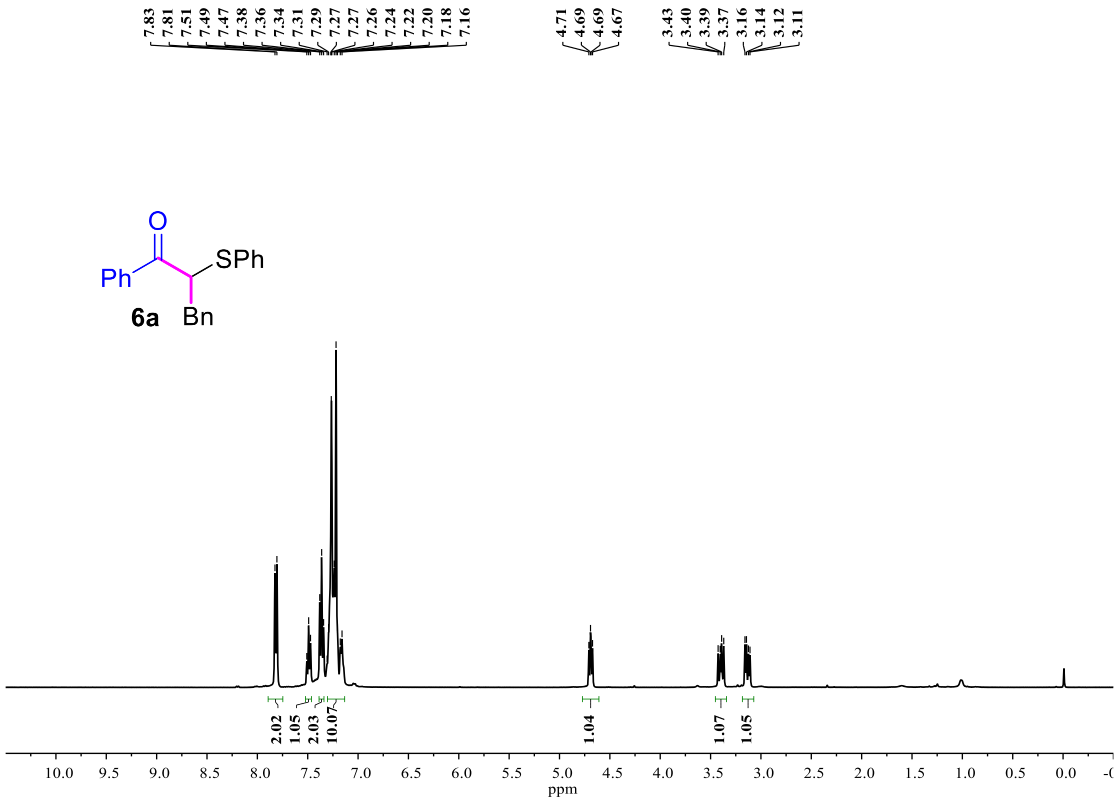

Supplementary Figure 122. ^{1}H NMR of **6a**.

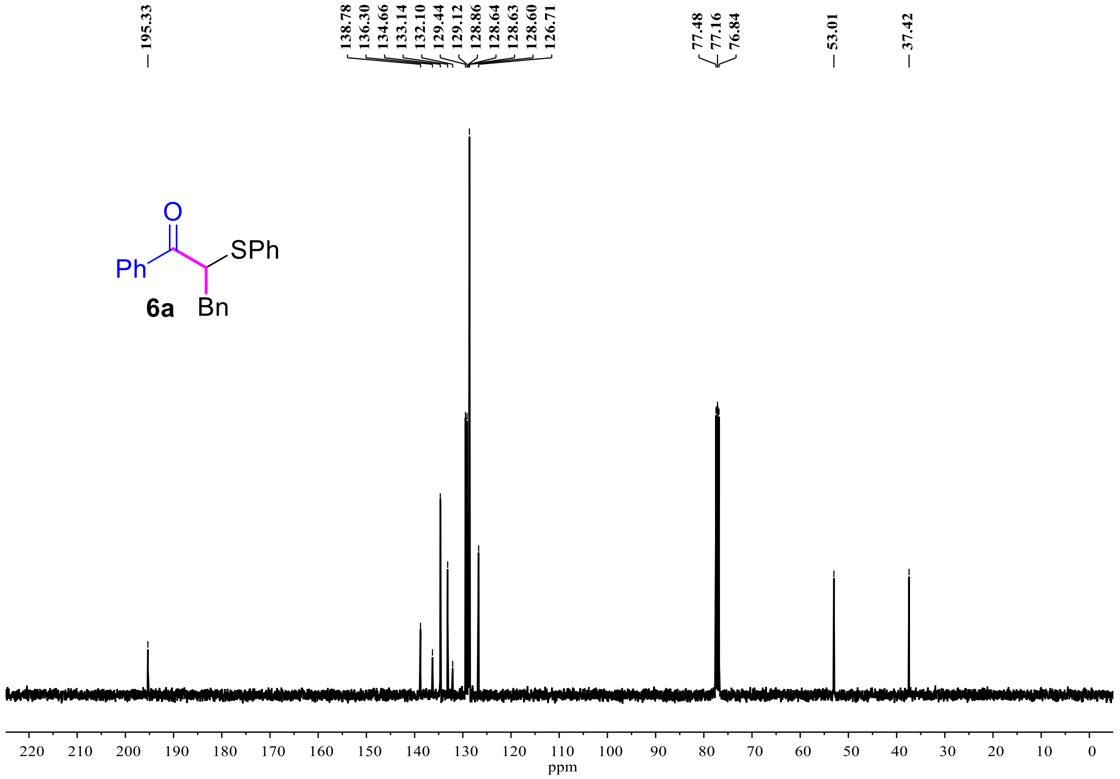

Supplementary Figure 123. ^{13}C NMR of **6a**.

6. Supplementary References

1. Denmark, S. E., Rossi, S., Webster, M. P. & Wang, H. Catalytic, Enantioselective Sulfenylation of Ketone-Derived Enoxysilanes. *J. Am. Chem. Soc.* **136**, 13016-13028 (2014).

2. Clayden, J., Davies, R. P., Hendy, M. A., Snaith, R. & Wheatley, A. E. H. The First Crystallographic Evidence for the Structures ofortho-Lithiated Aromatic Tertiary Amides. *Angew. Chem., Int. Ed.* **40**, 1238-1240 (2001).

3. Truong, T., Mesgar, M., Le, K. K. A. & Daugulis, O. General Method for Functionalized Polyaryl Synthesis via Aryne Intermediates. *J. Am. Chem. Soc.* **136**, 8568-8576 (2014).

4. Barbero, N. & Martin, R. Ligand-Free Ni-Catalyzed Reductive Cleavage of Inert Carbon–Sulfur Bonds. *Org. Lett.* **14**, 796-799 (2012).

5. Degennaro, L., Tota, A., De Angelis, S., Andresini, M., Cardellicchio, C., Capozzi, M. A., Romanazzi, G. & Luisi, R. A Convenient, Mild, and Green Synthesis of NH-Sulfoximines in Flow Reactors. *Eur. J. Org. Chem.* **2017**, 6486-6490 (2017).

6. Leiendecker, M., Chatupheeraphat, A. & Rueping, M. Metal catalyzed cross-coupling of aryl and benzyl methyl sulfides: nickel catalyzed Caryl–Csp3 and Csp3–Csp3 bond formations. *Org. Chem. Front.* **2**, 350-353 (2015).

7. Wencel-Delord, J., Nimphius, C., Patureau, F. W. & Glorius, F. [RhIIICp*]-Catalyzed Dehydrogenative Aryl-Aryl Bond Formation. *Angew. Chem., Int. Ed.* **51**, 2247-2251 (2012).

8. Pluth, M. D., Bergman, R. G. & Raymond, K. N. Acceleration of Amide Bond Rotation by Encapsulation in the Hydrophobic Interior of a Water-Soluble Supramolecular Assembly. *J. Org. Chem.* **73**, 7132-7136 (2008).

9. Dias, R. M. P. & Burtoloso, A. C. B. Catalyst-Free Insertion of Sulfoxonium Ylides into Aryl Thiols. A Direct Preparation of β-Keto Thioethers. *Org. Lett.* **18**, 3034-3037 (2016).

10. Wang, H., Wang, G., Lu, Q., Chiang, C., Peng, P., Zhou, J. & Lei, A. Catalyst-Free Difunctionalization of Activated Alkenes in Water: Efficient Synthesis of β-Keto Sulfides and Sulfones. *Chem Eur J* **22**, 14489-14493 (2016).

11. Zhang, J. J. & Schuster, G. B. Ylidions: a new reactive intermediate prepared by photosensitized one-electron oxidation of phenacyl sulfonium ylides. *J. Am. Chem. Soc.* **111**, 7149-7155 (1989).

12. Heredia, A. A., López-Vidal, M. G., Kurina-Sanz, M., Bisogno, F. R. & Peñéñory, A. B. Thiol-free chemoenzymatic synthesis of β-ketosulfides. *Beilstein J. Org. Chem.* **15**, 378-387 (2019).

13. Vedejs, E., Eberlein, T. H., Mazur, D. J., McClure, C. K., Perry, D. A., Ruggeri, R., Schwartz, E., Stults, J. S. & Varie, D. L. Thioaldehyde Diels-Alder reactions. *J. Org. Chem.* **51**, 1556-1562 (1986).
